# Oxidative [3+2]Cycloaddition of Alkynylphosphonates with Heterocyclic *N*-Imines: Synthesis of Pyrazolo[1,5-*a*]Pyridine-3-phosphonates

**DOI:** 10.3390/molecules27227913

**Published:** 2022-11-16

**Authors:** Igor Philippov, Yuriy Gatilov, Alina Sonina, Aleksey Vorob’ev

**Affiliations:** 1 Novosibirsk Institute of Organic Chemistry SB RAS, 9 Lavrentiev Avenue, 630090 Novosibirsk, Russia; 2 Department of Natural Science, Faculty of Organic Chemistry, Novosibirsk State University, 1 Pirogova Street, 630090 Novosibirsk, Russia

**Keywords:** alkynes, cycloaddition, heterocycles, oxidation, iron

## Abstract

A series of pyrazolo[1,5-*a*]pyridine-3-ylphosphonates were prepared with moderate to good yields by the oxidative [3+2]cycloaddition of 2-subtituted ethynylphosphonates with in situ generated pyridinium-N-imines and their annulated analogs. 2-Aliphatic and 2-Ph acetylenes demonstrate low activity, and the corresponding pyrazolopyridines were achieved with a moderate yield in the presence of 10 mol% Fe(NO_3_)_3_·9H_2_O. At the same time, tetraethyl ethynylbisphosphonate, diethyl 2-TMS- and 2-OPh-ethynylphosphonates possess much greater reactivity and the corresponding pyrazolo[1,5-*a*]pyridines, and their annulated derivatives were obtained with good to excellent yields without any catalyst. 2-Halogenated ethynylphosphonates also readily reacted with pyridinium-N-imines, forming complex mixtures containing poor amounts of 2-halogenated pyrazolopyridines.

## 1. Introduction

**Figure 1:**
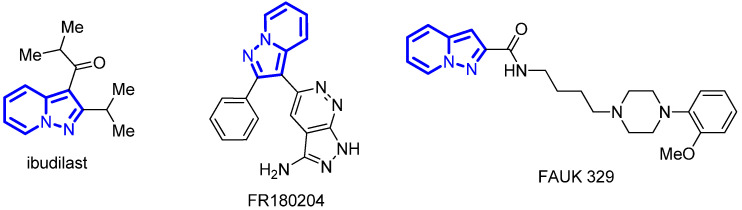
Biologically active derivatives of pyrazolo[1,5-*a*]pyridine.

Pyrazolo[1,5-*a*]pyridine is an important structural motif in modern medicinal chemistry, as it possesses high metabolic stability and acts as an isostere to indole, purine or other azaindole cores. An anti-inflammatory drug ibudilast (3-isobutyryl-2-isopropylpyrazolo[1,5-*a*]pyridine) (Figure 1) has been marketed in Japan for over 25 years and used for the treatment of asthma, post-stroke dizziness and ocular allergies [1,2]. Over the recent years, ibudilast has been paid much attention as an agent for therapy of multiple sclerosis and neurodegenerative disorders [3,4]. Recently, pyrazolo[1,5-*a*]pyridine scaffolds have been utilized in the design of DDX3X helicase [5], Pan-JAK kinase [6], C-terminal Src kinase [7], human dihydroorotate dehydrogenase [8], p110α-selective PI3 kinase [9], p38 kinase [10], ERK inhibitors [11], 5-HT4 [12], EP1 [13], D3 dopamine receptors antagonists, antitubercular [14] and antimalarial [15] agents, etc. Numerous general approaches have been reported for the construction of this heterocyclic core [16,17]. Among them, the 1,3-dipolar cycloaddition of electron-deficient alkynes and alkenes, such as unsaturated carbonyl compound [18] or nitroalkenes [19,20], with pyridinium-N-imines followed by oxidation is one of the most common methods of pyrazolo[1,5-*a*]pyridine synthesis.

**Scheme 1:**
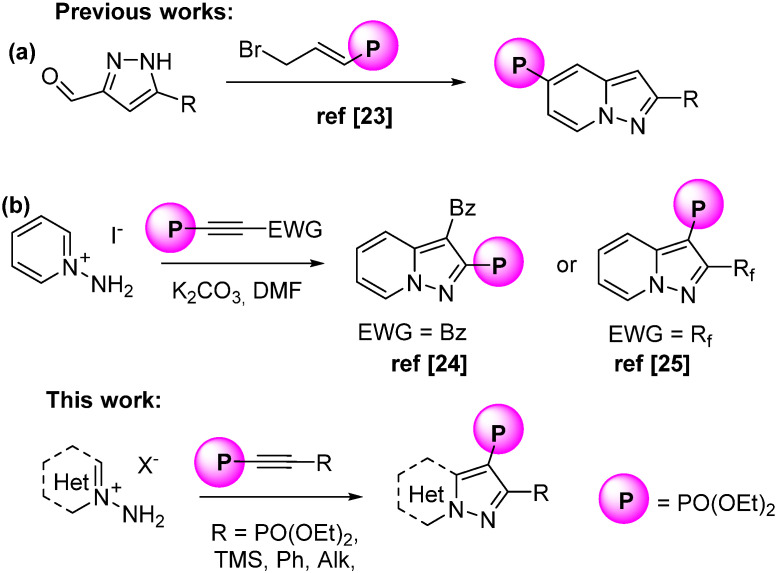
Approaches toward pyrazolo[1,5-*a*]pyridinyl phosphonate synthesis [24,25].

Due to the great importance of phosphonate groups in drug design research [21,22], the combination of a pyrazolo[1,5-*a*]pyridine moiety with a phosphonate group could be a promising building block for developing new pharmaceuticals. Previously, 2-substituted pyrazolo[1,5-*a*]pyridine-5-phosphonates were obtained by condensation of pyrazole-5-carbaldehydes with diethyl (3-bromoprop-1-en-1-yl)phosphonate [23] (Scheme 1a). The cycloaddition of EWG-substituted alkynylphosphonates with pyridinium-*N*-imines led to pyrazolo[1,5-*a*]pyridine-2-phosphonates [24] and to 3-phosphonates [25] when the EWG was a benzoyl or a perfluoroalkyl group, respectively (Scheme 1b). Since such cycloadditions represent fast and efficient routes to phosphorylated heterocylces, we decided to explore alkynylphosphonates with aliphatic, phenyl, OPh, halogen, TMS and PO(OEt)_2_-substituents at the triple bond in the reaction with pyridinium-*N*-imines. In contrast to benzoyl and perfluoroalkyl groups, aliphatic and phenyl substituents show electron donating properties and may reduce the reactivity of acetylenes in such cycloaddition reactions. At the same time, according to the Hammett parameter [26], the PO(OEt)_2_-group has a slightly stronger electron-withdrawing effect than CO_2_R. Thus, tetra-alkyl ethynylbisphosphonates have long been recognized to be active in Diels-Alder and 1,3-dipolar cycloaddition reactions [27]. Substituted dienes [28], furane [29], anthracene derivatives [30] and pyrones [31,32] were applied as diene components, and azides [33], nitrile oxides [34] and diazomethane [27] were used as 1,3-dipoles. Dialkyl ethynylphosphonates are also effective dipolarophiles and are often used for the construction of triazole [35,36], pyrazole [37,38] and β-lactam [39] units. However, only several examples of cycloaddition reactions of 2-substituted ethynylphosphonates bearing aryl-, alkyl- or sulfonamide substituent with azides [40,41] or nitrile oxides [42,43] have been reported recently.

## 2. Results

At the beginning of our work, *N*-aminopyridinium tetrafluoroborate **1** and diethyl phenylethynylphosphonate **2a** were taken as model substrates for screening optimal conditions. The reaction was followed using ^31^P NMR to estimate the phosphonate conversion and yield of products. The commonly used K_2_CO_3_/MeCN system (Table 1, entry 1) failed to reach the full conversion of **2a,** and only a 5-fold excess of salt **1** allowed for achieving the complete consumption of reagent **2a**. In the next step, various additives were tried to increase the reactivity of **2a**. Initially, we hypothesized that the electron-withdrawing character of the PO(OEt)_2_-group could be increased by the coordination of metal ions to the phosphonate group oxygen atom. However, strong Lewis acids such as AlCl_3_ or ZnCl_2_ preferably interacted with *N*-imines and, therefore, were not suitable for catalysis. Then, AgNO_3_ was used, as it was effective in the catalytic hydration of alkynylphosphonates [44]. However, no effect was found with 10 mol% AgNO_3_ (Table 1, entry 3). Next, we paid our attention to LiCl, which is known to catalyze cycloaddition reactions. The usage of 10 mol% led to notable conversion growth (Table 1, entry 4), but the increased load of LiCl resulted in lowering the conversion (Table 1, entry 5). The addition of Ni and Co salts had no significant effect on the reaction. During further experiments, redox-active additives were employed to accelerate the oxidation step of pyrazolo[1,5-*a*]pyridine synthesis (see discussion on the mechanism below). The application of chloranil or DDQ was unsuccessful, apparently due to the oxidation reaction with *N*-imines. Copper salts were previously applied for nitropyrazolo[1,5-*a*]pyridine synthesis from pyridinium-*N*-imines and nitrostyrenes [20]. However, in our case, Cu salts completely inhibited the reaction (Table 1, entries 9, 10). Fe(NO_3_)_3_ is broadly used as a catalyst in a wide scope of oxidation reactions. Recently, it has been found to be effective in the promotion of 3-acyl-1,2,4-oxadiazole synthesis from alkynes and nitriles [45]. Moreover, iron (III) nitrate mediated synthesis of acylisoxazoles from terminal alkynes was reported [46]. Therefore, we decided to try Fe(NO_3_)_3_ in nonahydrate form in our reaction. With an additive level of 10 mol% in CH_3_CN, we observed an 88% conversion of **2a** (Table 1, entry 11). Interestingly, other iron salts, such as FeCl_3_ and FeSO_4,_ were not as effective as Fe(NO_3_)_3_. Further experiments revealed that changing the solvent from CH_3_CN to the more polar DMSO led to the full conversion of **2a** (Table 1, entries 12, 13). No yield changes were observed with an increase in the additive level from 10 to 20 mol%; moreover, 5 mol% Fe(NO_3_)_3_ did not lead to the full conversion of phosphonate. Heating had a negative effect on the reaction (Table 1, entry 16). Therefore, the use of Fe(NO_3_)_3_ as an additive and DMSO as a solvent were found to be the optimal conditions.

**Scheme 2:**
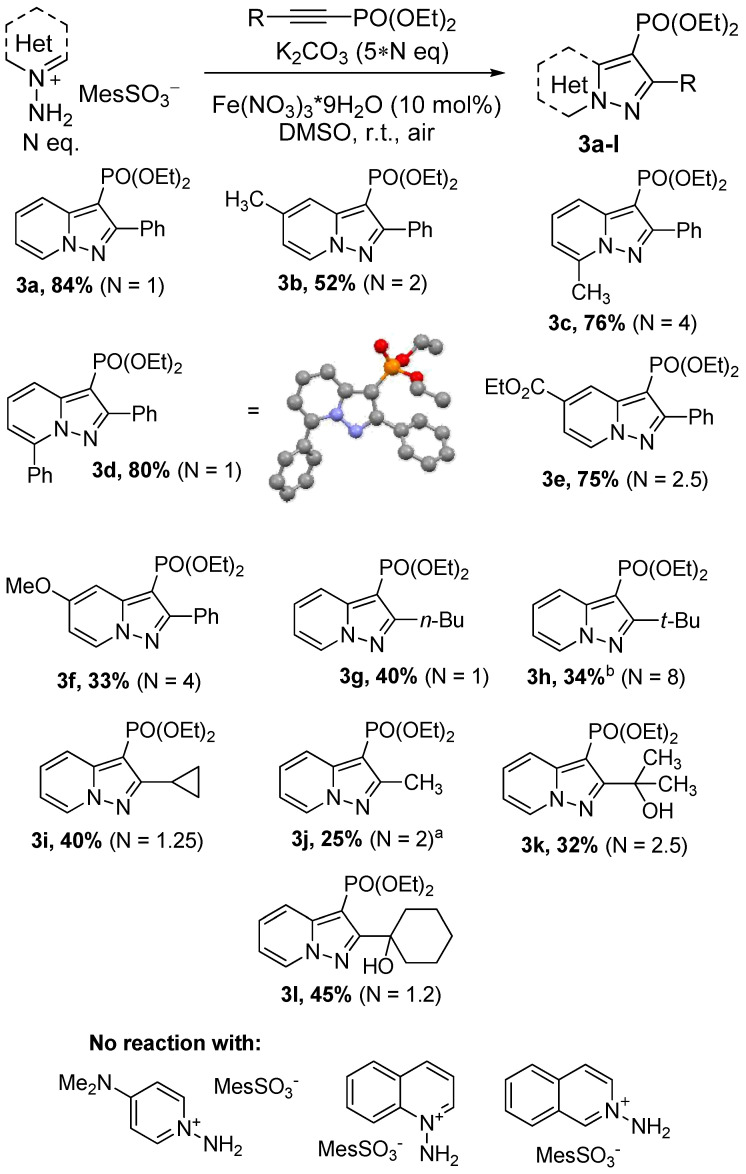
Reaction scopes for cycloaddition of N-imines with alkynylphosphonates (isolated yields). ^a^ Mixture of diethyl prop-1-yn-1-ylphosphonate and diethyl propa-1,2-diene-1-ylphosphonate was used. ^b^ Without the addition of Fe(NO_3_)_3_, 0% was observed.

With the optimized conditions in hand, the scope of pyridinium salts and alkynylphosphonates was explored (Scheme 2). First, we examined the effect of the substituents in the pyridinium ring of *N*-aminopyridinium salts on the reaction with phosphonate **2a**. The cycloaddition proceeded sluggishly in case of mild donating and moderate withdrawing substituents and required increased equivalents of starting salts. 2-Me-, 4-Me-, and 4-CO_2_Me-substituted *N*-aminopyridinium salts showed moderate to good yields of corresponding pyrazolopyridines **3b**–**e**. Despite the bulkiness of phenyl group, a good yield of 7-Ph-substituted pyrazolopyridine **3d** was obtained with only 1 eq of salt. Regioselectivity of the cycloaddition of **3d** was confirmed using X-ray analysis [47]. Strong donating groups are not favorable and significantly reduce reactivity. Thus, pyrazolopyridine **3f** was obtained only in 33% yield with 5 equivalents of 4-OMe-substituted salt. At the same time, 1-amino-4-NMe_2_-pyridinium mesitylenesulfonate was completely unreactive under used conditions. Such behavior was likely associated with the low acidity of the NH_2_-group, which could not be deprotonated by K_2_CO_3_. The use of DBU or *t*-BuOK as stronger bases also showed no positive results, apparently due to their reaction with alkyne. Quinolinium and isoquinolinium salts facilitated the dimerization of the corresponding *N*-imines [48,49] and no cycloaddition products were formed. Subsequently, we investigated the impact of the R-group in 2-R-alkynylphosphonate on the reaction with salt **1**. Pyrazolopyridines **3h**–**j** were obtained in moderate yields (30–40%), indicating that both alkyl and cycloalkyl groups led to the decrease in the corresponding phosphonate reactivity. Moreover, the phosphonate bearing the bulky *t*-Bu-group required a large excess of starting salts to obtain the desired product. α-Hydroxyalkyl substituted acetylenes were also moderately active and formed products **3k,l**.

**Scheme 3:**
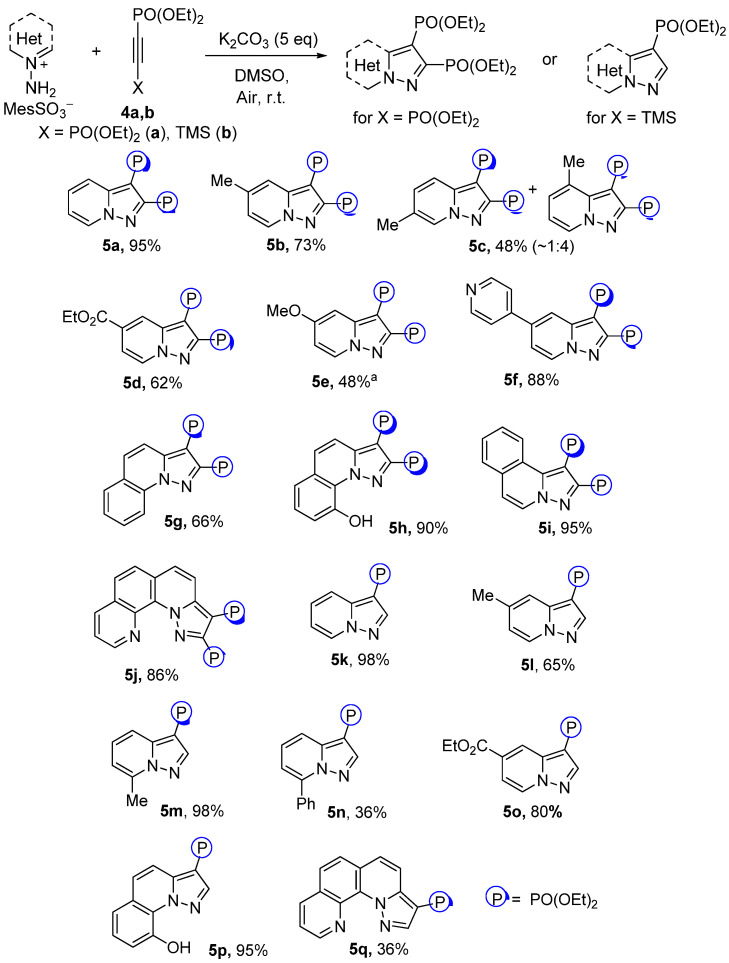
Reaction scopes for cycloaddition of N-imines with tetraethyl acetylene bisphosphonate (isolated yields). ^a^ The substance was obtained in mixture with 4-metoxypyridine and other unidentified products, yield determined using NMR with CH_2_Br_2_ as a standard.

Having succeeded in the Fe(NO_3_)_3_-catalyzed alkynylphosphonates cycloaddition, bisphosphonylated acetylene **4a** was further studied. In contrast to phosphonate **2a**, acetylene **4a** showed much greater reactivity and showed pyrazolo[1,5-*a*]pyridine-2,3-bisphosphonates **5a**–**j** with good to excellent yields without an Fe(NO_3_)_3_ additive (Scheme 3). However, pyridinium salts with strong donating groups were still much less reactive. Thus, 4-NMe_2_-substituted pyridinium salts did not undergo cycloaddition, and in the case of 4-OMe derivatives the corresponding pyrazolopyridine **5e** was obtained with a moderate yield in the mixture with the parent 4-OMe-pyridine. Enhanced activity of alkyne **4a** made it possible to obtain annulated derivatives **5g**–**j** with relatively high yields.

TMS-substituted ethynylphosphonate **4b** also possessed high reactivity toward pyridinium salts (Scheme 3). The loss of the TMS group during the process was observed and corresponding pyrazolopyridines **5k**–**o** were obtained. Annulated derivatives **5p**,**q** were prepared as well, but in the case of quinolinium and isoquinolinium salts only dimers of corresponding *N*-imines were observed. The higher activity of TMS-phosphonate **4b** in contrast to 2-alkyl- and 2-phenylalkynylphosphonates could be explained by the fast removal of the TMS group of **4b** in the K_2_CO_3_/DMSO medium with the formation of intermediate diethyl ethynylphosphonate.

**Scheme 4:**
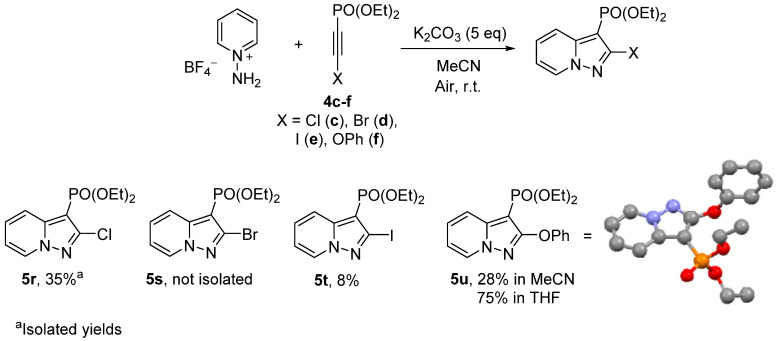
Reaction of pyridinium-N-imines with 2-halogenated diethyl acetylenephosphonate.

Next, the applicability of 2-halogenated and 2-OPh alkynylphosphonates **4c**–**f** in the cycloaddition reaction with pyridinium-*N*-imines was studied (Scheme 4). For all halogenated alkynes, the reaction resulted in the formation of complex reaction mixtures from which corresponding 2-Cl and 2-I-pyrazolopyridines **5r**,**t** were isolated with a low yield. Investigation of the reaction mixtures using NMR ^31^P and GC-MS for alkyne **4c** showed the formation of a significant amount of dehalogenated product **5k** when DMSO was used as a solvent. Various solvents were screened and the maximum yield of **5r** was reached in MeCN with K_2_CO_3_ as a base, while the nature of the inorganic base had no remarkable effect on the ratio of 5r:5k. When the excess of salt **1** was taken, formation of 2-aminopyrazolo[1,5-*a*]pyridine along with products **5k** and **5r** was also observed. In contrast to halogenated acetylenes **4c**–**e**, the cycloaddition reaction of OPh-substituted phosphonate **4f** in THF resulted in a good yield of pyrazolopyridine 5**u**. The regioselectivity for product **5u** was confirmed using X-ray analysis [50].

**Scheme 5:**
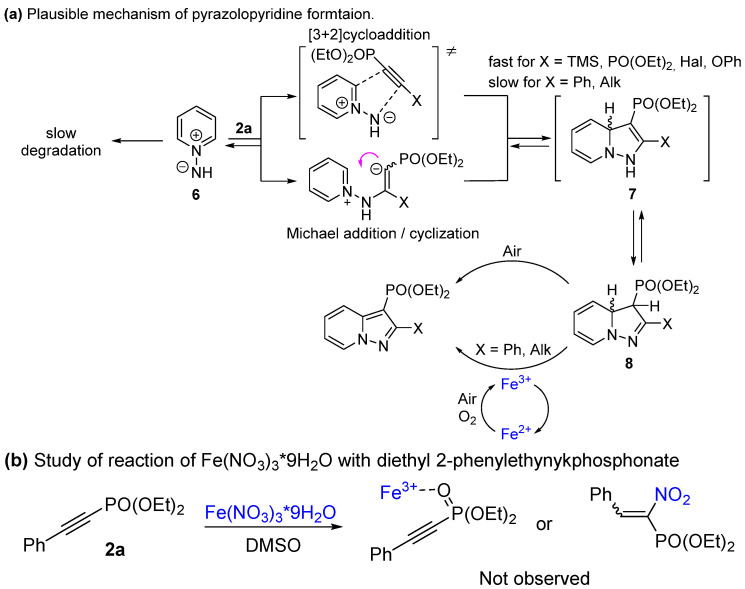
Plausible reaction mechanisms.

In terms of previous reports on *N*-imine cycloadditions [51], a plausible mechanism for the interaction of pyridinium salts with studied alkynes is shown in Scheme 5a. Ylide **6**, formed by the deprotonation of salt **1a**, reacts with alkynylphosphonates via concerted [3+2]cycloaddition or via Michael addition/intramolecular cyclization, yielding adduct **7**. Such intermediates are typically not observable due to the fast rearrangement of dihydropyrazolopyridines **8**, which lead to products **3a**–**l**, **5a**–**q** during oxidation. To shed some light on the role of iron (III) nitrate, we provided the reaction between **1a** and **2a** under an Ar atmosphere (Table 1, entries 19, 20). In the case of 10 mol% additive loading, only a poor yield of **3a** was observed, comparable to the value of the additive load. However, when 1 eq of Fe(NO_3_)_3_ was taken, a full conversion of **2a** was accomplished with a 70% isolated yield of **3a**. Additionally, Fe(NO_3_)_3_ could affect the reactivity by coordination of Fe^3+^-ion to the phosphonate group or by nitration of the triple bond (Scheme 5b). Nevertheless, no changes using ^31^P NMR were found after the addition of iron nitrate to phosphonate **2a** in the DMSO even after heating up to 100 °C. Therefore, it could be concluded that iron nitrate serves as a redox mediator in the oxidation of intermediate **8**.

Taking into account the low reactivity of 2-alkyl and 2-Ph- acetylenephosphonates, the cycloaddition step in this case is slow and probably reversible, so large amounts of *N*-imines are lost to degradation. Therefore, additional amounts of *N*-imines are required. For acetylenes **4a**–**f**, cycloaddition is fast and the oxidation of intermediate **8** is a limiting step.

## 3. Materials and Methods

Starting materials, unless otherwise noted, were obtained from commercial supplies and used without purification. O-(mesitylenesulfonyl)hydroxylamine (MSH) [52], mesitylenesulfonates of *N*-aminated heterocycles [52] and 1-aminopyridinium tetrafluoroborate [53] were obtained from previously published procedures. For the preparation of phenyl, alkyl and cycloalkyl alkynylphosphonates, Zhao’s CuSO_4_-catalysed cross-coupling procedure was applied [54]. Diethyl 2-chloroethynylphosphonate was obtained from a dichloroacetylene solution using the procedure of Svintsitskaya et al. [55] (**Caution! Dichloroacetylene is extremely explosive**). Tetraethyl acetylenebisphosphonate [56], diethyl 2-bromoethynylphosphonate [57] and diethyl 2-iodoethynylphosphonate [58] were prepared according to previously described methods.

The TLC was carried out on Sorbfil silica plates (UV 254) with further UV light visualization. Flash column chromatography was performed on silica gel (Macherey Nagel, pore size 60 E, 230–400 mesh). Spectral and analytical studies were provided at the Chemical Service Centre of Siberian Branch of the Russian Academy of Sciences. NMR spectra were recorded on Bruker Avance-300 (300.13 MHz for 1H, 121.5 MHz for 31P) and Avance-400 (400.13 MHz for 1H and 100.62 MHz for 13C) spectrometers, using the residual proton and carbon signals of CDCl3 (δH 7.24 ppm; δC 77.16 ppm) as internal standards. The 13C NMR spectra were registered with C-H spin decoupling. Copies of spectra of newly obtained compound are presented in the Supplementary Materials. The masses of molecular ions were determined by HRMS on a DFS Thermo scientific instrument (EI, 70 eV). Melting points were determined using Kofler hot-stage microscope and are uncorrected.

XRD data were obtained on a Bruker Kappa Apex II CCD diffractometer (Mo Kα radiation and a graphite monochromator) at 296K. The structures were solved by direct methods and refined by full-matrix least-squares method against all F2 in anisotropic approximation using the SHELX2014 programs set [59]. The H atoms positions were calculated geometrically and refined with the riding model. Absorption corrections were applied empirically using SADABS programs [60]. The solvent molecule is highly disordered and, therefore, this molecule was removed using the SQUEEZE procedure in the PLATON program [61]. Solvent accessible volume was calculated as 138 Å^3^ in unit cell.

**General procedure for diethyl 2-phenylpyrazolo[1,5-a]pyridine-3-phosphonates synthesis.**

*N*-aminopyridinium salt (N mmol) was dissolved in 5 mL DMSO and K_2_CO_3_ (2*N mmol) was added to produce pyridinium-*N*-imine. The resulting mixture was stirred for 5 min and alkynylphosphonate (1 mmol) was added followed with Fe(NO_3_)_3_*9H_2_O (10 mol%). The solution was kept with stirring overnight at room temperature under air atmosphere. Then, the mixture was diluted in 50 mL water and extracted three times with 20 mL of CH_2_Cl_2_. Extracts were combined, washed with water and dried over Na_2_SO_4_. Then, solvent was removed in vacuo and the resulting oil was purified by flash chromatography on silica with CHCl_3_/MeOH (50:1) as the eluent.

**Diethyl (2-phenylpyrazolo[1,5-a]pyridin-3-yl)phosphonate (3a).** Yellowish oil, 281mg (85%). NMR ^1^H (300 MHz, CDCl_3_): δ = 1.10 (t, *J* = 7.1 Hz, 6H), 3.89 (dp, *J* = 10.1 Hz, 7.3 Hz, 2H), 4.03 (dp, *J* = 10.1 Hz, 7.1 Hz, 2H), 6.89 (td, *J* = 6.9 Hz, 1.4 Hz, 1H), 7.30 (ddd, *J* = 9.2 Hz, 6.8 Hz, 1.2 Hz, 1H), 7.41 (m, 3H), 7.85 (m, 2H), 8.20 (d, *J* = 9.0 Hz, 1H), 8.50 (dd, *J* = 6.9 Hz, 1.1 Hz, 1H). NMR ^13^C (100.6 MHz, CDCl_3_): δ = 16.1 (d, *J* = 7.1 Hz), 61.9 (d, *J* = 4.8 Hz), 93.59 (d, *J* = 221.0 Hz), 113.89, 119.7, 126.6, 128.2, 128. 8, 129.1, 129.6, 132.6, 145.9 (d, *J* = 26.3 Hz), 157.6 (d, *J* = 12.9 Hz). NMR ^31^P (121.5 MHz, CDCl_3_): δ = 14.3 (s). HRMS (*m*/*z*): C_17_H_19_N_2_O_3_P^+•^, 330.1131 (found), 330.1133 (calc).

**Diethyl (5-methyl-2-phenylpyrazolo[1,5-a]pyridin-3-yl)phosphonate (3b).** Yellowish oil, 179mg (52%). NMR ^1^H (300 MHz, CDCl_3_): δ = 1.09 (t, *J* = 6.8 Hz, 6H); 2.39 (s, 3H); 3.83–3.92 (m, 2H); 3.98–4.06 (m, 2H); 6.70 (dd, *J* = 7.0 Hz, 2.0 Hz, 1H); 7.34–7.41 (m, 3H); 7.81–7.85 (m, 2H); 7.98 (m, 1H); 8.36 (dd, *J* = 7.0 Hz, 2.2 Hz, 1H). NMR ^13^C (100.6 MHz, CDCl_3_): δ = 15.8 (d, *J* = 7.2 Hz), 21.2; 61.4 (d, *J* = 4.8 Hz), 91.9 (d, *J* = 221.2 Hz), 115.9, 117.7, 127.6, 127.8, 128.7, 129.2, 132.5, 137.5, 145.9 (d, *J* = 26.4 Hz), 157.3 (d, *J* = 12.9 Hz). NMR ^31^P (121.5 MHz, CDCl_3_): δ = 14.8 (s). HRMS (*m*/*z*): C_18_H_21_N_2_O_3_P^+•^, 344.1277 (found), 344.1284 (calc).

**Diethyl (7-methyl-2-phenylpyrazolo[1,5-a]pyridin-3-yl)phosphonate (3c).** Yellowish oil, 261 mg (76%). NMR ^1^H (300 MHz, CDCl_3_): δ = 1.09 (t, *J* = 7.1 Hz, 6H), 2.75 (s, 3H), 3.81–3.94 (m, 2H), 3.96–4.04 (m, 2H), 6.74 (dd, *J* = 7.0 Hz, 1.1 Hz, 1H), 7.24 (dd, *J* = 9.1 Hz, 7.0 Hz, 1H), 7.33–7.44 (m, 3H), 7.85–7.91 (m, 2H), 8.11 (dm, *J* = 9.1 Hz, 1H). NMR ^13^C (100.6 MHz, CDCl_3_): δ = 15.8 (d, *J* = 7.2 Hz), 17.8, 61.4 (d, *J* = 4.8 Hz), 93.1 (d, *J* = 220.4 Hz), 112.8, 116.7, 126.3, 127.8, 128.6, 129.4, 132.7, 138.5, 145.8 (d, *J* = 26.5 Hz), 156.6 (d, *J* = 12.7 Hz). NMR ^31^P (121.5 MHz, CDCl_3_): δ = 14.8 (s). HRMS (*m*/*z*): C_18_H_21_N_2_O_3_P^+•^, 344.1286 (found), 344.1300 (calc).

**Diethyl (2,7-diphenylpyrazolo[1,5-a]pyridin-3-yl)phosphonate (3d).** Yellowish solid, 324 mg (80%). M.p. 122–124 °C. NMR ^1^H (300 MHz, CDCl_3_) δ = 1.14 (t, *J* = 7.1 Hz, 6H), 3.87–4.00 (m, 2H),4.01–4.16 (m, 2H), 7.00 (dd, *J* = 7.1 Hz, 1.3 Hz, 1H), 7.36–7.52 (m, 7H), 7.87–7.96 (m, 4H), 8.28 (ddd, *J* = 9.0 Hz, 1.4 Hz, 0.6 Hz, 1H). NMR ^13^C (100.6 MHz, CDCl_3_): δ = 16.1 (d, *J* = 7.2 Hz), 61.8 (d, *J* = 4.9 Hz), 93.4 (d, *J* = 220.0 Hz), 114.5, 118.3, 126.8, 128.0, 128.4, 128.9, 129.5, 129.7, 129.8, 132.8, 133.1 (d, *J* = 1 Hz), 140.9, 147.0(d, *J* = 27.1 Hz), 156.8 (d, *J* = 12.4 Hz). NMR ^31^P (121.5 MHz, CDCl_3_): δ = 14.7 (s). HRMS (*m*/*z*): C_23_H_23_O_3_N_2_P^+•^, 406.1442 (found), 406.1441 (calc).

**Ethyl 3-(diethoxyphosphoryl)-2-phenylpyrazolo[1,5-a]pyridine-5-carboxylate (3e).** Yellowish oil, 301 mg (75%). NMR ^1^H (300 MHz, CDCl_3_): δ = 1.16 (t, *J* = 7.1 Hz, 6H), 1.42 (t, *J* = 7.1 Hz, 3H), 3.89–4.02 (m, 2H), 4.03–4.15 (m, 2H), 4.42 (q, *J* = 7.1 Hz, 2H), 7.41–7.49 (m, 3H), 7.52 (dd, *J* = 7.1 Hz, 1.8 Hz, 1H), 7.86–7.94 (m, 2H), 8.55 (ddd, *J* = 7.1 Hz, 2.1 Hz, 1.0 Hz, 1H), 8.90 (m, 1H). NMR ^13^C (100.6 MHz, CDCl_3_): δ = 14.2, 15.9 (d, *J* = 7.1), 61.8, 61.9 (d, *J* = 5.3), 96.7 (d, *J* = 219.8), 112.8, 121.7, 128.1, 128.2, 128.4, 129.2, 129.4, 131.9, 144.5 (d, *J* = 25.5), 158.3 (d, *J* = 12.6), 164.6. NMR ^31^P (121.5 MHz, CDCl_3_): δ = 13.2 (s). HRMS (*m*/*z*): C_20_H_23_N_2_O_5_P^+•^, 402.1336 (found), 402.1339 (calc).

**Diethyl (5-methoxy-2-phenylpyrazolo[1,5-a]pyridin-3-yl)phosphonate (3f).** Yellowish oil, 118mg (33%). NMR ^1^H (300 MHz, CDCl_3_): δ = 1.11 (t, *J* = 7.1 Hz, 6H), 3.82–3.95 (m, 2H), 3.90 (s, 3H), 3.97–4.10 (m, 2H), 6.58 (dd, *J* = 7.5 Hz, 2.8 Hz, 1H), 7.36–7.45 (m, 3H), 7.51 (d, *J* = 2.8 Hz, 1H), 7.80–7.86 (m, 2H), 8.31 (dd, *J* = 7.5 Hz, 2.0 Hz, 1H). NMR ^13^C (100.6 MHz, CDCl_3_): 15.9 (d, *J* = 7.2 Hz), 55.7, 61.5 (d, *J* = 5.0 Hz), 91.8 (d, *J* = 222.4 Hz), 96.5, 108.1, 127.9, 128.8, 129.20, 129.22, 132. 6, 147.6 (d, *J* = 26.1 Hz), 157.8 (d, *J* = 12.8 Hz), 158.6. NMR ^31^P (121.5 MHz, CDCl_3_): δ = 15.2 (s). HRMS (*m*/*z*): C_18_H_21_N_2_O_4_P^+•^, 360.1235 (found), 360.1234 (calc).

**Diethyl (2-butylpyrazolo[1,5-a]pyridin-3-yl)phosphonate (3g).** Yellow liquid, 124 mg (40%). NMR ^1^H (300 MHz, CDCl_3_): δ = 0.92 (t, *J* = 7.3 Hz, 3H), 1.28 (t, *J* = 7.1 Hz, 6H), 1.41 (m, 2H), 1.74 (m, 2H), 2.93 (m, 2H), 3.91–4.02 (m, 2H), 4.03–4.19 (m, 2H), 6.82 (td, *J* = 6.9 Hz, 1.4 Hz, 1H), 7.24 (ddd, *J* = 8.9 Hz, 6.8 Hz, 1.2 Hz, 1H), 7.95 (dt, *J* = 8.9 Hz, 1.3 Hz, 1H), 8.40 (dm, *J* = 6.8 Hz, 1H). NMR ^13^C (100.6 MHz, CDCl_3_): δ = 13.8, 16.2 (d, *J* = 6.9 Hz), 22.6, 27.6, 31.5, 61.4 (d, *J* = 5.0 Hz), 93.4 (d, *J* = 223.3 Hz), 112.7, 118.4, 126.0, 128.4, 144.5 (d, *J* = 26.8 Hz), 160.3 (d, *J* = 14.7 Hz). NMR ^31^P (121.5 MHz, CDCl_3_): δ = 16.3 (s). HRMS (*m*/*z*): C_15_H_23_N_2_O_3_P^+•^, 310.1441 (found), 310.1445 (calc).

**Diethyl (2-tert-butylpyrazolo[1,5-a]pyridin-3-yl)phosphonate (3h).** Yellow oil, 105 mg (34%). NMR ^1^H (300 MHz, CDCl_3_): δ = 1.25 (t, *J* = 7.0 Hz, 6H), 1.47 (s, 9H), 3.91–4.17 (m, 4H), 6.79 (td, *J* = 7.0 Hz, 1.2 Hz, 1H), 7.19 (ddd, *J* = 9.1 Hz, 6.8 Hz, 1.2 Hz, 1H), 8.01 (dt, *J* = 9.1 Hz, 1.2 Hz, 1H), 8.38 (ddt, *J* = 6.9 Hz, 2.1 Hz, 1.1 Hz). NMR ^13^C (100.6 MHz, CDCl_3_): δ = 16.2 (d, *J* = 7.1 Hz), 30.2, 34.4, 61.6 (d, *J* = 5.0 Hz), 91.9 (d, *J* = 221.7 Hz), 112.7, 118.9, 126.2, 128.7, 145.7 (d, *J* = 25.4 Hz), 167.5 (d, *J* = 15.4 Hz), 173.9. NMR ^31^P (121.5 MHz, CDCl_3_): δ = 16.1 (s). HRMS (*m*/*z*): C_15_H_23_N_2_O_3_P^+•^, 310.1442 (found), 310.1445 (calc).

**Diethyl (2-cyclopropylpyrazolo[1,5-a]pyridin-3-yl)phosphonate (3i).** Yellowish liquid, 118 mg (40%). NMR ^1^H (300 MHz, CDCl_3_): δ = 0.98–1.09 (m, 4H), 1.28 (t, *J* = 7.0 Hz, 6H), 2.35–2.45 (m, 1H), 3.95–4.06 (m, 2H), 4.08–4.19 (m, 2H), 6.79 (td, *J* = 6.9 Hz, 1.4 Hz, 1H), 7.23 (ddd, *J* = 8.9 Hz, 6.9 Hz, 1.2 Hz, 1H), 7.92 (dt, *J* = 8.9 Hz, 1.2 Hz, 1H), 8.35 (dm, *J* = 6.9 Hz, 1H). NMR ^13^C (100.6 MHz, CDCl_3_): δ = 8.3, 9.4, 16.1 (d, *J* = 6.9 Hz), 61.4 (d, *J* = 4.7 Hz), 93.7 (d, *J* = 222.2), 112.6, 117.9, 125.9, 128.3, 144.6 (d, *J* = 26.3 Hz), 161.3 (d, *J* = 14.1 Hz). NMR ^31^P (121.5 MHz, CDCl_3_): δ = 15.5 (s). HRMS (*m*/*z*): C_14_H_19_N_2_O_3_P^+•^, 294.1126 (found), 294.1128 (calc).

**Diethyl (2-methyl[1,5-a]pyridin-3-yl)phosphonate (3j).** Orange oil, 76.0 mg (25 %). NMR ^1^H (300 MHz, CDCl_3_): δ = 1.26 (t, *J* = 7.2Hz, 6H), 2.23 (s, 3H), 3.90–4.04 (m, 2H), 4.04–4.17 (m, 2H), 6.80 (t, *J* = 6.7Hz, 1H), 7.24 (t, *J* = 7.8Hz, 1H), 7.92 (d, *J* = 9.1Hz, 1H), 8.37 (d, *J* = 6.8Hz, 1H). NMR ^13^C (100.6 MHz, CDCl_3_): δ = 13.7, 16.4 (d, *J* = 7Hz), 61.6 (d, *J* = 5Hz), 94.2 (d, *J* = 223Hz), 112.9, 118.4, 126.4, 128.5, 144.8 (d, *J* = 26.6 Hz), 156.2 (d, *J* = 14.0 Hz). NMR ^31^P (121.5 MHz, CDCl3): δ = 15.2 (s). HRMS (*m*/*z*): C_12_H_17_O_3_N_2_P^+•^, 268.0968 (found), 268.0971 (calc).

**Diethyl (2-(2-hydroxypropan-2-yl)pyrazolo[1,5-a]pyridin-3-yl)phosphonate (3k).** Recrystallization from ethanol. Yellowish solid, 99.9 mg (32%). M.p. 82–84 °C. NMR ^1^H (300 MHz, CDCl_3_): δ = 1.29 (t, *J* = 7.1 Hz, 6H), 1.72 (s, 6H), 6.95–4.09 (m, 2H), 4.11–4.24 (m, 2H), 6.45 (s, 1H), 6.89 (td, *J* = 6.9 Hz, 1.4 Hz, 1H), 7.31 (ddd, *J* = 8.9 Hz, 6.9 Hz, 1.2 Hz, 1H), 7.71 (dt, *J* = 8.9 Hz, 1.2 Hz, 1H), 8.46 (dm, *J* = 6.9 Hz, 1H). NMR ^13^C (100.6 MHz, CDCl_3_): δ = 16.1 (d, *J* = 6.9 Hz), 29.9, 62.1 (d, *J* = 4.9 Hz), 69.8, 90.9 (d, *J* = 218.7 Hz), 113.1, 117.8, 126.6, 129.0, 143.9 (d, *J* = 22.2 Hz), 167.9 (d, *J* = 16.1 Hz). NMR ^31^P (121.5 MHz, CDCl_3_): δ = 18.3 (s). HRMS (*m*/*z*): C_14_H_21_N_2_O_4_P^+•^, 312.1241 (found), 312.1243 (calc).

**Diethyl (2-(1-hydroxycyclohexyl)pyrazolo[1,5-a]pyridin-3-yl)phosphonate (3l).** Recrystallization from ethanol. Orange solid, 158 mg (45%). NMR ^1^H (300 MHz, CDCl_3_): δ = 1.25 (t, *J* = 7.0 Hz, 6H), 1.62 (m, 4H), 1.90 (m, 6H), 2.11 (d, *J* = 11.0 Hz, 3H), 3.98 (m, 2H), 4.13 (dp, *J* = 10.1, 7.2, 2H), 6.08 (s, 1H), 6.86 (td, *J* = 6.9 Hz, 1.4 Hz, 1H), 7.27 (m, 1H), 7.68 (d, *J* = 8.9 Hz, 1H), 8.43 (d, *J* = 6.9 Hz, 1H). NMR ^13^C (100.6 MHz, CDCl_3_): δ = 16.0 (d, *J* = 7.0 Hz), 21.6, 25.6, 37.3, 62.0 (d, *J* = 4.8 Hz), 70.7, 90.9 (d, *J* = 218.2 Hz), 113.0, 117.8, 126.5, 128.9, 143.8 (d, *J* = 22.1 Hz), 168.7 (d, *J* = 16.1 Hz). NMR ^31^P (121.5 MHz, CDCl_3_): δ = 18.6 (s). HRMS (*m*/*z*): C_17_H_25_N_2_O_4_P^+•^, 352.1545 (found), 352.1547 (calc).

**General experimental procedure for synthesis of pyrazolopyridines 5a-r, 5t,u.***N*-aminopyridinium salt (1 mmol) was dissolved in 5 mL DMSO and K_2_CO_3_ (5 mmol) was added to produce pyridinium-*N*-imine. The resulting mixture was stirred for 5 min and alkynylphosphonate (1 mmol) was added. The solution was kept with stirring overnight at RT under air atmosphere. Then, the mixture was diluted in water and extracted three times with 20 mL of CH_2_Cl_2_. The extracts were combined and washed with water and dried over Na_2_SO_4_. Then, the solvent was removed in vacuo and the resulting oil was purified by flash chromatography on silica with CHCl_3_/MeOH (50:1).

**Tetraethyl pyrazolo[1,5-a]pyridine-2,3-diylbis(phosphonate) (5a).** Yellow oil, 370 mg (95%). NMR ^1^H (300 MHz, CDCl_3_): δ = 1.17 (t, *J* = 7.0 Hz, 6H), 1.22 (t *J* = 7.0 Hz, 6H), 3.86–420 (m, 8H), 6.86 (tdd, *J* = 6.9 Hz, 1.4 Hz, 0.7 Hz, 1H), 7.22 (ddd, 9.1 Hz, 6.8 Hz, 1.2 Hz, 1H), 8.13 (dm, *J* = 9.1, 1H), 8.42 (dm, *J* = 6.8, 1H). NMR ^13^C (100.6 MHz, CDCl_3_): δ = 15.6 (d, *J* = 7.0 Hz), 15.7 (d, *J* = 7.0 Hz), 61.5 (d, *J* = 5.1 Hz), 62.4 (d, *J* = 5.8 Hz), 99.95 (dd, *J* = 221.9 Hz, 24.2 Hz), 114.34, 119.58, 126.41, 128.42, 144.30 (dd, *J* = 25.6 Hz, 9.9 Hz), 146.84 (dd, *J* = 227.7 Hz, 14.1 Hz). NMR ^31^P (121.5 MHz, CDCl_3_): δ = 7.8 (s), 11.3 (s). HRMS (*m*/*z*): C_15_H_24_N_2_O_6_P_2_^+•^, 390.1104 (found), 390.1106 (calc).

**Tetraethyl (5-methylpyrazolo[1,5-a]pyridine-2,3-diyl)bis(phosphonate) (5b).** Orange oil, 194 mg (48%). NMR ^1^H (300 MHz, CDCl_3_): δ = 1.28 (t, 7.1 Hz, 6H), 1.32 (t, 7.1 Hz, 6H), 2.38 (s, 3H), 3.98–4.14 (m, 4H), 4.15–4.30 (m, 4H), 6.77 (dd, *J* = 7.0 Hz, 2.0 Hz, 1H), 8.02 (s, 1H), 8.39 (dd, *J* = 7.0 Hz, 2.1 Hz, 1H). NMR ^13^C (100.6 MHz, CDCl_3_): δ = 16.0 (d, *J* = 7.1 Hz), 16.1 (d, *J* = 7.1 Hz), 21.2, 61.9 (d, *J* = 5.1 Hz), 62.9 (d, *J* = 5.9 Hz), 98.8 (dd, *J* = 222.5 Hz, 24.4 Hz), 117.3, 118.3, 127.9, 138.2, 145.1 (dd, *J* = 25.9 Hz, 9.8 Hz), 147.3 (dd, *J* = 228.1, 14.1 Hz). NMR ^31^P (121.5 MHz, CDCl_3_): δ = 8.2 (s), 12.0 (s). HRMS (*m*/*z*): C_16_H_26_N_2_O_6_P_2_^+•^, 404.1261 (found), 404.1264 (calc).

**Tetraethyl (6-methylpyrazolo[1,5-a]pyridine-2,3-diyl)bis(phosphonate) and tetraethyl (4-methylpyrazolo[1,5-a]pyridine-2,3-diyl)bis(phosphonate) (5c).** An inseparable mixture of isomers was obtained. Orange oil, 194 mg (48%). Signals of the heterocyclic part were as follows. For tetraethyl (6-methylpyrazolo[1,5-a]pyridine-2,3-diyl)bis(phosphonate) NMR ^1^H (300 MHz, CDCl_3_): δ = 2.34 (s, 3H), 7.19 (dd, *J* = 9.2 Hz, 1.2 Hz, 1H), 8.16 (d, *J* = 9.2 Hz, 1H), 8.33 (s). For tetraethyl (4-methylpyrazolo[1,5-a]pyridine-2,3-diyl)bis(phosphonate) NMR ^1^H (300 MHz, CDCl_3_): δ = 2.80.

**Ethyl 2,3-bis(diethoxyphosphoryl)pyrazolo[1,5-a]pyridine-5-carboxylate (5d).** Orange oil, 286 mg (62%). NMR ^1^H (300 MHz, CDCl_3_): δ = 1.35 (t, *J* = 7.1 Hz, 6H), 1.38 (t, *J* = 7.1 Hz, 6H), 1.40 (t, *J* = 7.2 Hz, 3H), 4.10–4.36 (m, 8H), 4.41 (q, *J* = 7.2 Hz, 2H), 7.57 (ddd, *J* = 7.3 Hz, 1.9 Hz, 0.8 Hz, 1H), 8.58 (ddd, *J* = 7.3 Hz, 2.1 Hz, 1.0 Hz, 1H), 8.96 (s, 1H). NMR ^13^C (100.6 MHz, CDCl_3_): δ = 14.1, 16.1 (d, *J* = 6.9 Hz), 16.2 (d, *J* = 6.9 Hz), 61.9, 62.4 (d, *J* = 5.4 Hz), 63.3 (d, *J* = 5.9 Hz), 103.9 (dd, *J* = 220.8 Hz, 23.8 Hz), 114.0, 122.6, 128.8, 143.8 (dd, *J* = 24.9 Hz, 10.1 Hz), 148.5 (dd, *J* = 227.8 Hz, 14.0 Hz), 164.2. NMR ^31^P (121.5 MHz, CDCl_3_): δ = 7.2 (s), 10.4 (s). HRMS (*m*/*z*): C_18_H_28_N_2_O_8_P_2_^+•^, 462.1312 (found), 462.1315 (calc).

**Tetraethyl (5-methoxypyrazolo[1,5-a]pyridine-2,3-diyl)bis(phosphonate) (5e).** Yellow liquid, 202mg. Mixture of 53e and 4-methoxypyridine. NMR ^1^H (300 MHz, CDCl_3_): δ = 1.26 (t, *J* = 7.1 Hz, 6H), 1.30 (t, *J* = 7.1 Hz, 6H), 3.83 (s, 3H), 3.96–4.28 (m, 8H), 6.59 (dd, *J* = 7.6, 2.8, 1.0, 1H), 7.50 (d, *J* = 2.8, 1H), 8.29 (dd, *J* = 7.6, 2.0, 1H). NMR ^13^C (100.6 MHz, CDCl_3_): NMR ^31^P (121.5 MHz, CDCl_3_): δ = 8.1 (s), 12.3 (s). HRMS (m/z): C_16_H_26_N_2_O_7_P_2_^+•^, 420.1210 (found), 420.1215 (calc).

**Tetraethyl 5-(pyridin-4-yl)pyrazolo[1,5-a]pyridine-2,3-diyldiphosphonate (5f).** Brown oil, 411 mg (88 %). NMR ^1^H (300 MHz, CDCl_3_): δ = 1.30 (t, *J* = 7.1 Hz, 6H), 1.35 (t, *J* = 7.1 Hz, 6H), 4.04–4.33 (m, 8H), 7.25 (d, *J* = 7.2 Hz, 2.0 Hz, 1H), 7.56 (m, 2H), 8.59–8.68 (m, 4H). NMR ^13^C (100.6 MHz, CDCl_3_): δ = 16.0 (d, *J* = 6.0 Hz), 16.1 (d, *J* = 6.0 Hz), 62.1 (d, *J* = 5.1 Hz), 62.9 (d, *J* = 5.9 Hz), 101.5 (dd, *J* = 221.4 Hz, 24.0 Hz), 113.5, 117.8, 121.1, 129.2, 136.7, 144.7, 144.8 (dd, *J* = 25.6 Hz, 10.1 Hz), 148.1 (dd, *J* =227.6 Hz, 13.9 Hz), 150.3. NMR ^31^P (121.5 MHz, CDCl_3_): δ = 5.7 (s), 9.5 (s). HRMS (*m*/*z*): C_20_H_27_N_3_O_6_P_2_^+•^, 467.1366 (found), 467.1370 (calc).

**Tetraethyl pyrazolo[1,5-a]quinoline-2,3-diyldiphosphonate (5g).** Yellow oil, 290 mg (66 %). NMR ^1^H (300 MHz, CDCl_3_): δ = 1.32 (t, *J* = 7.0 Hz, 6H), 1.41 (t, *J* = 7.1 Hz, 6H), 4.10-4.22 (m, 4H), 4.27-4.38 (m, 4H), 7.52 (ddd, *J* = 8.3 Hz, 7.2 Hz, 1.2 Hz, 1H), 7.64 (d, *J* = 9.4 Hz, 1H), 7.70 (ddd, *J* = 8.4 Hz, 7.2 Hz, 1.4 Hz, 1H), 7.80 (dd, *J* = 7.8 Hz, 1.4 Hz, 1H), 8.18 (d, *J* = 9.4 Hz, 1H), 8.64 (dm, *J* = 8.4 Hz, 1H). NMR ^13^C (100.6 MHz, CDCl_3_): δ = 16.0 (d, *J* = 6.9 Hz), 16.2 (d, *J* = 6.6 Hz), 62.3 (d, *J* = 5.2 Hz), 63.2 (d, *J* = 6.0 Hz), 102.9 (dd, *J* = 220.5, 24.7 Hz), 116.2 (d, *J* = 1.3 Hz), 117.1, 123.7, 126.3, 128.1, 128.3, 129.9, 133.87, 143.21 (dd, *J* = 25.5 Hz, 9.4 Hz), 146.10 (dd, *J* = 230.8 Hz, 14.1 Hz). NMR ^31^P (121.5 MHz, CDCl_3_): δ = 8.6 (s), 12.1 (s). HRMS (*m*/*z*): C_19_H_26_N_2_O_6_P_2_^+•^, 440.1258 (found), 440.1261 (calc).

**Tetraethyl (9-hydroxypyrazolo[1,5-a]quinoline-2,3-diyl)bis(phosphonate) (5h).** Purified by chromatography on Al_2_O_3_, with CHCl3 as eluent. Yellow oil, 410 mg (90%). NMR ^1^H (300 MHz, CDCl_3_): δ = 1.33 (t, *J* = 7.1 Hz, 6H), 1.40 (t, *J* = 7.1 Hz, 6H), 4.07–4.38 (m, 8H), 7.23 (dd, *J* = 7.9 Hz, 1.2 Hz, 1H), 7.29 (dd, *J* = 7.9 Hz, 1.2 Hz, 1H), 7.41 (t, *J* = 7.9 Hz, 1H), 7.65 (d, *J* = 9.6 Hz, 1H), 8.12 (d, *J* = 9.6 Hz, 1H), 11.36 (s, 1H). NMR ^13^C (100.6 MHz, CDCl_3_): δ = 16.1 (d, *J* = 6.8 Hz), 16.2 (d, *J* = 6.5 Hz), 62.4 (d, *J* = 5.2 Hz), 63.3 (d, *J* = 6.0 Hz), 102.8 (dd, *J* = 219.5 Hz, 23.1 Hz), 116.6, 116.7, 118.6, 121.6, 125.5, 127.4, 129.4, 142.9 (dd, *J* = 25.2 Hz, 9.8 Hz), 144.1 (dd, *J* = 228.3 Hz, 14.8 Hz), 148.4. NMR ^31^P (121.5 MHz, CDCl_3_): δ = 6.1 (s), 10.7 (s). HRMS (*m*/*z*): C_19_H_26_N_2_O_7_P_2_^+•^, 456.1205 (found), 456.1210 (calc).

**Tetraethyl pyrazolo[5,1-a]isoquinoline-1,2-diylbis(phosphonate) (5i).** Yellow oil, 418 mg (95 %). NMR ^1^H (300 MHz, CDCl_3_): δ = 1.32 (t, *J* = 7.1 Hz, 6H), 1.40 (t, *J* = 7.0 Hz, 6H), 4.15–4.38 (m, 8H), 7.21–7.30 (m, 1H), 7.68 (td, *J* = 7.3 Hz, 2.0 Hz, 2H), 7.77 (dd, *J* = 7.3 Hz, 2.1 Hz, 1H), 8.34 (dd, *J* = 7.3 Hz, 2.0 Hz, 1H), 9.33 (dm, *J* = 7.3 Hz, 1H). NMR ^13^C (100.6 MHz, CDCl_3_): δ = 16.3 (d, *J* = 6.9 Hz), 16.4 (d, *J* = 6.9 Hz), 62.7 (d, *J* = 5.6 Hz), 63.3 (d, *J* = 6.0 Hz), 103.9 (dd, *J* = 218.5 Hz, 24.5 Hz), 116.2, 124.1, 126.2, 127.5, 127.8, 128.6, 129.7, 130.4, 141.9 (dd, *J* = 24.9 Hz, 9.1 Hz), 148.3 (dd, *J* = 229.7 Hz, 13.9 Hz). NMR ^31^P (121.5 MHz, CDCl_3_): δ = 8.6 (s), 12.7 (s). HRMS (*m*/*z*): C_19_H_26_N_2_O_6_P_2_^+•^, 440.1261 (found), 440.1265 (calc).

**Tetraethyl pyrazolo[1,5-a][1,10]phenanthroline-2,3-diylbis(phosphonate) (5j).** Purified by chromatography on Al_2_O_3_, with CHCl_3_ as eluent. Yellow oil, 422 mg (86 %). NMR ^1^H (300 MHz, CDCl_3_): δ = 1.27 (t, *J* = 7.1 Hz, 6H), 1.43 (t, *J* = 7.1 Hz, 6H), 4.05–4.22 (m, 4H), 4.31–4.55 (m, 4H), 7.49 (dd, *J* = 8.1 Hz, 4.1 Hz, 1H), 7.68 (d, *J* = 9.3 Hz, 1H), 7.71 (s, 2H), 8.16 (dd, *J* = 8.2, 1.9, 1H), 8.47 (d, *J* = 9.3 Hz, 1H), 9.10 (dd, *J* = 4.2 Hz, 1.9 Hz, 1H). NMR ^13^C (100.6 MHz, CDCl_3_): δ = 15.9 (d, *J* = 6.9 Hz), 16.1 (d, *J* = 6.9 Hz), 61.9 (d, *J* = 5.0 Hz), 63.3 (d, *J* = 6.0 Hz), 101.1 (dd, *J* = 218.4, 24.3), 119.0, 121.9, 125.0, 126.1, 126.3, 127.8, 128.7, 129.9, 135.8, 139.3, 145.6 (dd, *J* = 27.3, 9.1), 147.3 (dd, *J* = 232.1, 12.8), 149.8. NMR ^31^P (121.5 MHz, CDCl_3_): δ = 8.0 (s), 11.7 (s). HRMS (*m*/*z*): C_22_H_27_N_3_O_6_P_2_^+•^, 491.1366 (found), 491.1370 (calc).

**Diethyl (pyrazolo[1,5-a]pyridin-3-yl)phosphonate (5k).** Without purification. Yellow oil, 110 mg (98%). NMR ^1^H (300 MHz, CDCl_3_): δ = 1.34 (t, *J* = 7.2 Hz, 6H), 4.02–4.25 (m, 4H), 6.97 (td, *J* = 6.9 Hz, *J* = 1.2 Hz, 1H), 7.38 (ddd, *J* = 8.9 Hz, *J* = 6.9 Hz, *J* = 1.0 Hz, 1H), 7.98 (dt, *J* = 8.9 Hz, *J* = 1.0 Hz, 1H), 8.23 (d, *J* = 1.9 Hz, 1H), 8.57 (ddt, *J* = 7.0 Hz, *J* = 2.1 Hz, *J* = 1.0 Hz, 1H). NMR ^13^C (100.6 MHz, CDCl_3_): δ = 16.4 (d, *J* = 7 Hz), 62.0 (d, *J* = 5 Hz), 96.4 (d, *J* = 224 Hz), 113.7, 118.6, 126.7, 129.3, 142.8 (d, *J* = 25.0 Hz), 146.4 (d, *J* = 14.0 Hz). NMR ^31^P (121.5 MHz, CDCl_3_): δ = 14.3 (s). HRMS (*m*/*z*): C_11_H_15_N_2_O_3_P^+•^, 254.0822 (found), 254.0820 (calc).

**Diethyl (5-methylpyrazolo[1,5-a]pyridin-3-yl)phosphonate (5l).** Without purification. Brown oil, 75 mg (65%). NMR ^1^H (300 MHz, CDCl_3_): δ = 1.30 (t, *J* = 7.1 Hz, 6H), 2.41 (s, 3H), 3.96–4.20 (m, 4H), 6.74 (dd, *J* = 7.1 Hz, *J* = 1.5 Hz, 1H), 7.69 (s, 1H), 8.11 (d, *J* = 1.6 Hz, 1H), 8.39 (dd, *J* = 7.15 Hz, *J* = 1.4 Hz). NMR ^13^C (100.6 MHz, CDCl_3_): δ = 16.2 (d, *J* = 7 Hz), 21.2, 61.8 (d, *J* = 5 Hz), 94.8 (d, *J* = 224 Hz), 116.1, 116.8, 128.2, 137.9, 142.9 (d, *J* = 25 Hz), 146.2 (d, *J* = 14 Hz). NMR ^31^P (121.5 MHz, CDCl_3_): δ = 14.8 (s). HRMS (*m*/*z*): C_12_H_17_N_2_O_3_P^+•^, 268.0975 (found), 268.0971 (calc).

**Diethyl (7-methylpyrazolo[1,5-a]pyridin-3-yl)phosphonate (5m).** Without purification. Brown oil, 125 mg (98%). NMR ^1^H (300 MHz, CDCl_3_): δ = 1.29 (t, *J* = 7 Hz, 6H), 2.77 (s, 3H), 3.96–4.21 (m, 4H), 6.79 (d, *J* = 7 Hz, 1H), 7.25–7.32 (m, 1H), 7.82 (d, *J* = 8.8 Hz, 1H), 8.24 (d, *J* = 1.6 Hz, 1H). NMR ^13^C (100.6 MHz, CDCl_3_): δ = 16.2 (d, *J* = 7 Hz), 17.9, 61.7 (d, *J* = 5 Hz), 96.3 (d, *J* = 224 Hz), 112.8, 115.9, 126.5, 138.9, 145.6 (d, *J* = 14 Hz). NMR ^31^P (121.5 MHz, CDCl_3_): δ = 14.7 (s). HRMS (*m*/*z*): C_12_H_17_N_2_O_3_P^+•^, 268.0974 (found), 268.0971 (calc).

**Diethyl (7phenylpyrazolo[1,5-a]pyridin-3-yl)phosphonate (5m).** Purified by chromatography on SiO_2_, with EtOAc:hexane (1:1) as eluent. Yellow solid, 74mg (36 %). NMR ^1^H (300 MHz, CDCl_3_): δ = 1.32 (t, *J* = 7.1 Hz, 6H), 3.99–4.24 (m, 4H), 6.99 (d, *J* = 6.9 Hz, 1H), 7.39–7.47 (m, 1H), 7.48–7.57 (m, 3H), 7.79–7.85 (m, 2H), 7.95 (d, *J* = 8.9 Hz, 1H), 8.22 (s, 1H). NMR ^13^C (100.6 MHz, CDCl_3_): δ = 16.2 (d, *J* = 7.2 Hz), 61.8 (d, *J* = 5.2 Hz), 96.4 (dd, *J* = 227 Hz), 114.2, 117.2, 126.7, 128.4, 129.1, 129.7, 132.8, 141.4, 143.4, 143.8, 145.7, 145.9. NMR ^31^P (121.5 MHz, CDCl_3_): δ = 14.5 (s). HRMS (*m*/*z*): C_17_H_19_N_2_O_3_P^+•^, 330.1125 (found), 330.1133 (calc).

**Methyl 3-dietoxyphosphorylpyrazolo[1,5-a]pyridine-5-carboxylate (5o).** Without purification. Brown oil, 110 mg (80%). NMR ^1^H (300 MHz, CDCl_3_): δ = 1.33 (t, *J* = 7.1 Hz, 6H), 3.96 (s, 3H), 4.01–4.24 (m, 4H), 7.51 (dd, *J* = 7 Hz, *J* = 1 Hz, 1H), 8.27 (d, *J* = 1.7 Hz, 1H), 8.56 (dm, *J* = 6.5 Hz, 1H), 8.64 (s, 1H). NMR ^13^C (100.6 MHz, CDCl_3_): δ = 16.2 (d, *J* = 7 Hz), 52.7, 62.1 (d, *J* = 5 Hz), 99.9 (d, *J* =222 Hz), 112.7, 120.9, 128.1, 128.9, 141.6 (d, *J* = 25 Hz), 147.0 (d, *J* = 12 Hz), 164.9. NMR ^31^P (121.5 MHz, CDCl_3_): δ = 12.8 (s). HRMS (*m*/*z*): C_13_H_17_N_2_O_5_P^+•^, 312.0864 (found), 312.0871 (calc).

**Diethyl (9-hydroxypyrazolo[1,5-a]quinoline-3-yl)phosphonate (5p).** Without purification. Colorless crystals, 130 mg (95%). NMR ^1^H (300 MHz, CDCl_3_): δ = 1.33 (t, *J* = 7.2 Hz, 6H), 4.00–4.28 (m, 4H), 7.26 (d, *J* = 7.8Hz, 1H), 7.31 (d, *J* = 7.8 Hz, 1H), 7.41 (t, *J* = 7.9 Hz, 1H), 7.66 (d, *J* = 9.4 Hz, 1H), 7.81 (d, *J* = 9.4 Hz, 1H), 8.21 (d, *J* = 1.6 Hz, 1H), 11.68 (s, 1H). NMR ^13^C (100.6 MHz, CDCl_3_): δ = 16.2 (d, *J* = 7 Hz), 62.0 (d, *J* = 5 Hz), 98.8 (d, *J* = 222 Hz), 115.7, 116.4, 118.4, 122.3, 125.2, 126.8, 129.0, 140.5 (d, *J* = 25 Hz), 142.5 (d, *J* = 14 Hz), 148.5. NMR ^31^P (121.5 MHz, CDCl_3_): δ = 14.1 (s). HRMS (*m*/*z*): C_15_H_17_N_2_O_4_P^+•^, 320.0922 (found), 320.0921 (calc).

**Diethyl (pyrazolo[1,5-a][1,10]phenantroline-3-yl)phosphonate (5q).** Without purification. Brown oil, 115 mg (75%). NMR _1_H (300 MHz, CDCl_3_): δ = 1.28 (t, *J* = 7 Hz, 6H), 3.98–4.22 (m, 4H), 7.61 (dd, *J* = 8.2 Hz, *J* = 4.3 Hz, 1H), 7.75–7.86 (m, 3H), 8.24 (d, *J* = 9.1 Hz, 1H), 8.29 (dd, *J* = 8.2 Hz, *J* = 1.3 Hz, 1H), 9.38 (dd, *J* = 4.3 Hz, *J* = 1.4 Hz). NMR ^13^C (100.6 MHz, CDCl_3_): δ = 16.2 (d, *J* = 7.5 Hz), 62.0 (d, *J* = 5 Hz), 97.9 (d, *J* = 222 Hz), 118.2, 122.1, 124.9, 126, 126.7, 127.7, 129.2, 130.6, 136.3, 139.8, 143.6, 143.8, 146.4, 146.5, 150.5. NMR ^31^P (121.5 MHz, CDCl_3_): δ = 14.8 (s). HRMS (*m*/*z*): C_18_H_18_N_3_O_3_P^+•^, 355.1078 (found), 355.1080 (calc).

**Diethyl (2-chloro[1,5-a]pyridin-3-yl)phosphonate (5r).** Purified by chromatography on SiO_2_, with CHCl_3_/MeOH (50:1) as the eluent. Yellow oil, 92.4 mg (32%). NMR ^1^H (300 MHz, CDCl_3_): δ = 1.33 (t, *J* = 7.1 Hz, 6H), 3.99-4.24 (m, 4H), 6.95 (t, *J* = 6.7 Hz, 1H), 7.37 (t, *J* = 7.5 Hz, 1H), 8.09 (d, *J* = 8.7 Hz, 1H), 8.41 (d, *J* = 6.7 Hz, 1H). NMR ^13^C (100.6 MHz, CDCl_3_): δ = 16.1 (d, *J* = 6.9 Hz), 62.1 (d, *J* = 6.0 Hz), 94.1 (d, *J* = 225 Hz), 113.9, 118.7, 127.3, 128.4, 145.2 (d, *J* = 24 Hz), 145.2 (d, *J* = 25 Hz), 146.7 (d, *J* = 9 Hz). NMR ^31^P (121.5 MHz, CDCl_3_): δ = 10.4 (s). HRMS (*m*/*z*): C_11_H_14_O_3_N_2_^35^ClP^+•^, 288.0422 (found), 288.0425 (calc).

**Diethyl (2-iodo[1,5-a]pyridin-3-yl)phosphonate (5t).** Purified by chromatography on SiO2, with CHCl_3_/MeOH (50:1) as the eluent. Yellow oil, 57.0 mg (15%). NMR ^1^H (300 MHz, CDCl_3_): δ = 1.34 (t, *J* = 7.1 Hz, 6H), 3.97–4.10 (m, 2H), 4.11–4.24 (m, 2H), 6.88 (td, *J* = 6.8 Hz, *J* = 1 Hz, 1H), 7.32 (t, *J* = 8.5 Hz, 1H), 8.15 (d, *J* = 8.9 Hz, 1H), 8.46 (d, *J* = 6.9 Hz, 1H). NMR ^13^C (100.6 MHz, CDCl_3_): δ = 16.1 (d, *J* = 6.9 Hz), 62.1 (d, *J* = 6.0 Hz), 101.8 (d, *J* = 227 Hz), 106.4 (d, *J* = 12 Hz), 113.6, 118.4, 127.0, 128.1, 145.2 (d, *J* = 25 Hz). NMR ^31^P (121.5 MHz, CDCl_3_): δ = 11.1 (s). HRMS (*m*/*z*): C_11_H_14_O_3_N_2_IP^+•^, 379.9783 (found), 379.9781 (calc).

**Diethyl (2-phenoxy[1,5-a]pyridin-3-yl)phosphonate (5u).** THF was used as the solvent instead of MeCN. Purified by chromatography on SiO_2_, with CH_2_Cl_2_/NEt_3_ (100:1) as the eluent. Yellow crystals, 260.0 mg (75%). NMR ^1^H (300 MHz, CDCl_3_): δ = 1.31 (t, *J* = 7.1 Hz, 6H), 4.02–4.25 (m, 4H), 6.83 (td, *J* = 7.1 Hz, *J* = 1.4 Hz, 1H), 7.12–7.30 (m, 4H), 7.30–7.42 (m, 3H), 8.01 (d, *J* = 9 Hz, 1H), 8.27 (dt, *J* = 6.9 Hz, *J* = 1 Hz, 1H). NMR ^13^C (100.6 MHz, CDCl_3_): δ = 16.1 (d, *J* = 6.9 Hz), 62.1 (d, *J* = 6.0 Hz), 82.3 (d, *J* = 227 Hz), 112.7, 118.0, 119.5, 124.6, 126.9, 128.8, 129.4, 145.0 (d, *J* = 24 Hz), 155.2, 164.4 (d, *J* = 7 Hz). HRMS (*m*/*z*): C_17_H_19_O_4_N_2_P^+•^, 346.1086 (found), 346.1082 (calc).

## 4. Conclusions

In summary, an operationally simple procedure toward 3-phosphonylated pyrazolo[1,5-*a*]pyridines based on the cycloaddition of diethyl 2-alkyl-, 2-cycloalkyl-, phenylethynylphosphonates and *N*-aminopyridinium salts with catalysis by cheap Fe(NO_3_)_3_·9H_2_O was achieved. The reaction is applicable for mild donating and withdrawing functional groups in the pyridinium ring and for various 2-alkyl and α-hydroxyalkyl acetylenephosphonates. Tetraethyl ethylene-1,2-bis(phosphonate), diethyl 2-TMS- and 2-OPh-ethynylphosphonate reacted smoothly without any catalyst to yield the corresponding 2,3-bisphosphonylated and 3-phosphonylated pyrazolo[1,5-*a*]pyridines and their annulated analogs.

## Figures and Tables

**Table 1:**
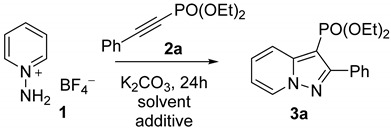
Optimization of the reaction conditions.

Entry	Solvent/Additive ^1^	Conversion of 2a, % ^2^
1	CH_3_CN/–	63
2	CH_3_CN/– ^3^	100 (64 ^4^)
3	CH_3_CN/AgNO_3_ (10 mol%)	63
4	CH_3_CN/LiCl (10 mol%)	80
5	CH_3_CN/LiCl (20 mol%)	56
6	CH_3_CN/Ni(OAc)_2_ (10 mol%)	64
7	CH_3_CN/Co(NO_3_)_2_ (10 mol%)	60
8	CH_3_CN/chloranil or DDQ (1 eq)	0
9	CH_3_CN/CuSO_4_ (10 mol%)	0
10	CH_3_CN/CuI (10 mol%)	0
11	CH_3_CN/Fe(NO_3_)_3_·9H_2_O (10 mol%)	88
12	DMF/Fe(NO_3_)_3_·9H_2_O (10 mol%)	82
13	DMSO/Fe(NO_3_)_3_·9H_2_O (10 mol%)	100 (84 ^4^)
14	DMSO/Fe(NO_3_)_3_·9H_2_O (20 mol%)	100 (84 ^4^)
15	DMSO/Fe(NO_3_)_3_·9H_2_O (5 mol%)	89
16	DMSO/Fe(NO_3_)_3_·9H_2_O (10 mol%), 60 °C	67
17	DMSO/FeCl_3_	47
18	DMSO/FeSO_4_·5H_2_O (10 mol%)	66
19	DMSO/Fe(NO_3_)_3_·9H_2_O (10 mol%) under Ar	15
20	DMSO/Fe(NO_3_)_3_·9H_2_O (1 eq) under Ar	100 (70 ^4^)

^1^ If otherwise noted, reactions were carried out under air. ^2^ Yield is determined using ^31^P NMR without purification. ^3^ Five equivalents of salt **1** were used. ^4^ Isolated yield of **3a.**

## Data Availability

Not applicable.

## Supplementary Materials

The following supporting information can be downloaded at: https://www.mdpi.com/article/10.3390/molecules27227913/s1, Figure S1: Crystal structures of pyrazolopyridines **3d** (A) and **5u** (B). Copies of ^1^H, ^13^C and ^31^P NMR spectra of the obtained compounds.

## Sample Availability

Samples of the compounds are available from the authors.

## References

[1] Rolan P., Hutchinson M., Johnson K. (2009). Ibudilast: A Review of Its Pharmacology, Efficacy and Safety in Respiratory and Neurological Disease. Expert Opin. Pharmacother..

[2] Ledeboer A., Hutchinson M.R., Watkins L.R., Johnson K.W. (2007). Ibudilast (AV-411): A New Class Therapeutic Candidate for Neuropathic Pain and Opioid Withdrawal Syndromes. Expert Opin. Investig. Drugs.

[3] Fox R.J., Coffey C.S., Conwit R., Cudkowicz M.E., Gleason T., Goodman A., Klawiter E.C., Matsuda K., McGovern M., Naismith R.T. (2018). Phase 2 Trial of Ibudilast in Progressive Multiple Sclerosis. N. Engl. J. Med..

[4] Goodman A.D., Gyang T., Smith A.D. (2016). Ibudilast for the Treatment of Multiple Sclerosis. Expert Opin. Investig. Drugs.

[5] Nakao S., Nogami M., Iwatani M., Imaeda T., Ito M., Tanaka T., Tawada M., Endo S., Cary D.R., Ohori M. (2020). Identification of a Selective DDX3X Inhibitor with Newly Developed Quantitative High-Throughput RNA Helicase Assays. Biochem. Biophys. Res. Commun..

[6] Calbet M., Ramis I., Calama E., Carreño C., Paris S., Maldonado M., Orellana A., Calaf E., Pauta M., De Alba J. (2019). Novel Inhaled Pan-JAK Inhibitor, LAS194046, Reduces Allergen-Induced Airway Inflammation, Late Asthmatic Response, and PSTAT Activation in Brown Norway Rats. J. Pharmacol. Exp. Ther..

[7] O’Malley D.P., Ahuja V., Fink B., Cao C., Wang C., Swanson J., Wee S., Gavai A.V., Tokarski J., Critton D. (2019). Discovery of Pyridazinone and Pyrazolo[1,5-*a*]Pyridine Inhibitors of C-Terminal Src Kinase. ACS Med. Chem. Lett..

[8] Sainas S., Pippione A.C., Lupino E., Giorgis M., Circosta P., Gaidano V., Goyal P., Bonanni D., Rolando B., Cignetti A. (2018). Targeting Myeloid Differentiation Using Potent 2-Hydroxypyrazolo[1,5-*a*]Pyridine Scaffold-Based Human Dihydroorotate Dehydrogenase Inhibitors. J. Med. Chem..

[9] Kendall J.D., Giddens A.C., Tsang K.Y., Marshall E.S., Lill C.L., Lee W.-J., Kolekar S., Chao M., Malik A., Yu S. (2017). Novel Pyrazolo[1,5-a]Pyridines with Improved Aqueous Solubility as P110α-Selective PI3 Kinase Inhibitors. Bioorganic Med. Chem. Lett..

[10] Wu H.-C., Chu J.-H., Li C.-W., Hwang L.-C., Wu M.-J. (2016). Palladium-Catalyzed Regioselective Arylation of Pyrazolo[1,5-*a*]Pyridines via C–H Activation and Synthetic Applications on P38 Kinase Inhibitors. Organometallics.

[11] Lechtenberg B.C., Mace P.D., Sessions E.H., Williamson R., Stalder R., Wallez Y., Roth G.P., Riedl S.J., Pasquale E.B. (2017). Structure-Guided Strategy for the Development of Potent Bivalent ERK Inhibitors. ACS Med. Chem. Lett..

[12] Nirogi R., Mohammed A.R., Shinde A.K., Gagginapally S.R., Kancharla D.M., Middekadi V.R., Bogaraju N., Ravella S.R., Singh P., Birangal S.R. (2018). Synthesis, Structure–Activity Relationships, and Preclinical Evaluation of Heteroaromatic Amides and 1,3,4-Oxadiazole Derivatives as 5-HT_4_ Receptor Partial Agonists. J. Med. Chem..

[13] Umei K., Nishigaya Y., Kondo A., Tatani K., Tanaka N., Kohno Y., Seto S. (2017). Novel Pyrazolo[1,5-a]Pyridines as Orally Active EP 1 Receptor Antagonists: Synthesis, Structure-Activity Relationship Studies, and Biological Evaluation. Bioorganic Med. Chem..

[14] Lu X., Williams Z., Hards K., Tang J., Cheung C.-Y., Aung H.L., Wang B., Liu Z., Hu X., Lenaerts A. (2019). Pyrazolo[1,5-*a*]Pyridine Inhibitor of the Respiratory Cytochrome *Bcc* Complex for the Treatment of Drug-Resistant Tuberculosis. ACS Infect. Dis..

[15] Large J.M., Birchall K., Bouloc N.S., Merritt A.T., Smiljanic-Hurley E., Tsagris D.J., Wheldon M.C., Ansell K.H., Coombs P.J., Kettleborough C.A. (2019). Potent Inhibitors of Malarial P. Falciparum Protein Kinase G: Improving the Cell Activity of a Series of Imidazopyridines. Bioorganic Med. Chem. Lett..

[16] Kendall J.D. (2011). Synthesis and Reactions of Pyrazolo[1,5-a]Pyridines and Related Heterocycles. Curr. Org. Chem..

[17] Mohan D.C., Ravi C., Rao S.N., Adimurthy S. (2015). Copper-Mediated Synthesis of Pyrazolo[1,5-a]Pyridines through Oxidative Linkage of C–C/N–N Bonds. Org. Biomol. Chem..

[18] Ravi C., Samanta S., Mohan D., Reddy N., Adimurthy S. (2017). Synthesis of Functionalized Pyrazolo[1,5-a]Pyridines: [3+2] Cycloaddition of N-Aminopyridines and α,β-Unsaturated Carbonyl Compounds/Alkenes at Room Temperature. Synthesis.

[19] Ravi C., Chandra Mohan D., Naresh Kumar Reddy N., Adimurthy S. (2015). Substrate Selective Synthesis of Pyrazolo[1,5-a]Pyridines through [3+2] Cycloaddition of N-Aminopyridines and β-Nitro Styrenes. RSC Adv..

[20] Motornov V.A., Tabolin A.A., Nelyubina Y.V., Nenajdenko V.G., Ioffe S.L. (2020). Copper-Mediated Oxidative [3+2]-Annulation of Nitroalkenes and Pyridinium Imines: Efficient Synthesis of 3-Fluoro- and 3-Nitro-Pyrazolo[1,5-*a*]Pyridines. Org. Biomol. Chem..

[21] Rodriguez J.B., Gallo-Rodriguez C. (2018). The Role of the Phosphorus Atom in Drug Design. ChemMedChem.

[22] Demmer C.S., Krogsgaard-Larsen N., Bunch L. (2011). Review on Modern Advances of Chemical Methods for the Introduction of a Phosphonic Acid Group. Chem. Rev..

[23] Wang J.-W., Jia J., Xie Y.-F., Feng L., Xu H.-Q., Meng S., Zhao G.-L., Xu W.-R., Ge Y.-Q. (2013). Synthesis of Nitrogen Bridgehead Heterocycles with Phosphonates via a Novel Tandem Process. Heterocycles.

[24] Liao L., Zhang H., Zhao X. (2018). Selenium-π-Acid Catalyzed Oxidative Functionalization of Alkynes: Facile Access to Ynones and Multisubstituted Oxazoles. ACS Catal..

[25] Huang Q., He D., Han J., Chen J., He W., Deng H., Shao M., Zhang H., Cao W. (2018). [3+2] Cycloaddition of N-Aminopyridines and Perfluoroalkynylphosphonates: Facile Synthesis of Perfluoroalkylated Pyrazolo[1,5-a]Pyridines Containing a Phosphonate Moiety. Synthesis.

[26] Hansch C., Leo A., Taft R.W. (1991). A Survey of Hammett Substituent Constants and Resonance and Field Parameters. Chem. Rev..

[27] Seyferth D., Paetsch J. (1969). Diels-Alder Reaction in Organometallic Chemistry. V. Tetramethyl Acetylenediphosphonate and Dimethyl Chloroacetylenephosphonate and Their Reactions with Cyclopentadiene, 1,3-Cyclohexadiene, and Diazomethane. J. Org. Chem..

[28] Tverdomed S.N., Röschenthaler G.-V., Kalinovich N., Lork E., Dogadina A.V., Ionin B.I. (2008). New α-Substituted Alkylbenzene- and Dialkylbenzene-1,2-Diphosphonates: Side-Chain Metalation of Tetraethyl 4-Methyl- and 4,5-Dimethylbenzene-1,2-Diphosphonates. Tetrahedron.

[29] Mahajna M., Quistad G.B., Casida J.E. (1996). Retro-Diels−Alder Reaction: Possible Involvement in the Metabolic Activation of 7-Oxabicyclo[2.2.1]Hepta-2(3),5(6)-Diene-2,3-Dicarboxylates and a Phosphonate Analog. Chem. Res. Toxicol..

[30] Selmani S., Schipper D.J. (2018). Orientation Control of Molecularly Functionalized Surfaces Applied to the Simultaneous Alignment and Sorting of Carbon Nanotubes. Angew. Chem..

[31] Kyba E.P., Rines S.P., Owens P.W., Chou S.-S.P. (1981). A Novel Synthesis of 1,2-Diphosphorylbenzenes. Tetrahedron Lett..

[32] Ziegler T., Layh M., Effenberger F. (1987). Darstellung Hochsubstituierter Aromaten Über Diels-Alder-Reaktionen Mit 2*H*-Pyran-2-onen. Chem. Ber..

[33] Artyushin O.I., Matveeva E.V., Bushmarinov I.S., Odinets I.L. (2012). Water as a Promoting Media for 1,3-Dipolar Cycloaddition of Phosphorylated Azides to Internal Alkynes. Arkivoc.

[34] Vereshchagina Y.A., Alimova A.Z., Sharova E.V., Artyushin O.I., Chachkov D.V., Ishmaeva E.A. (2013). Polarity and Structure of Diphosphorus-Substituted Isoxazole and 1,2,3-Triazole. Russ. J. Org. Chem..

[35] Mukai S., Flematti G.R., Byrne L.T., Besant P.G., Attwood P.V., Piggott M.J. (2012). Stable Triazolylphosphonate Analogues of Phosphohistidine. Amino Acids.

[36] Lukáč M., Hocková D., Keough D.T., Guddat L.W., Janeba Z. (2017). Novel Nucleotide Analogues Bearing (1H-1,2,3-Triazol-4-Yl)Phosphonic Acid Moiety as Inhibitors of Plasmodium and Human 6-Oxopurine Phosphoribosyltransferases. Tetrahedron.

[37] Matoba K., Yonemoto H., Fukui M., Yamazaki T. (1984). Structural modification of bioactive compounds. II. Syntheses of aminophosphonoic acids. Chem. Pharm. Bull..

[38] Heimgartner H., Mlostoń G., Pipiak P. (2017). [3+2] Cycloadditions of N-Protected ‘(S)-Diazoproline’ with Selected Acetylenes. Heterocycles.

[39] Kowalski M.K., Mlostoń G., Obijalska E., Heimgartner H. (2016). Application of Diethyl Ethynephosphonate for the Synthesis of 3-Phosphonylated β-Lactams via Kinugasa Reaction. Arkivoc.

[40] Zhu S., Zhang Y., Li P., Bi W., Chen X., Zhao Y. (2017). Synthesis of Novel Phosphorylated Chrysin Derivatives by 1,3-Dipolar Cycloaddition Reaction. Phosphorus Sulfur Silicon Relat. Elem..

[41] Song W., Zheng N., Li M., Ullah K., Zheng Y. (2018). Rhodium(I)-Catalyzed Azide-Alkyne Cycloaddition (RhAAC) of Internal Alkynylphosphonates with High Regioselectivities under Mild Conditions. Adv. Synth. Catal..

[42] Perez V., Fadel A., Rabasso N. (2017). Synthesis of N-Sulfonyl Ynamido-Phosphonates: Valuable Partners for Cycloadditions. Synthesis.

[43] Feng Q., Huang H., Sun J. (2021). Ru-Catalyzed [3+2] Cycloaddition of Nitrile Oxides and Electron-Rich Alkynes with Reversed Regioselectivity. Org. Lett..

[44] Xiang J., Yi N., Wang R., Lu L., Zou H., Pan Y., He W. (2015). Synthesis of β-Ketophosphonates via AgNO_3_-Catalyzed Hydration of Alkynylphosphonates: A Rate-Enhancement Effect of Methanol. Tetrahedron.

[45] Bian Q., Wu C., Yuan J., Shi Z., Ding T., Huang Y., Xu H., Xu Y. (2020). Iron Nitrate-Mediated Selective Synthesis of 3-Acyl-1,2,4-Oxadiazoles from Alkynes and Nitriles: The Dual Roles of Iron Nitrate. J. Org. Chem..

[46] Lai Z., Li Z., Liu Y., Yang P., Fang X., Zhang W., Liu B., Chang H., Xu H., Xu Y. (2018). Iron-Mediated Synthesis of Isoxazoles from Alkynes: Using Iron(III) Nitrate as a Nitration and Cyclization Reagent. J. Org. Chem..

[47] The Crystallographic Data Was Deposited at the Cambridge Crystallographic Data Center, CCDC 200118. https://www.ccdc.cam.ac.uk/.

[48] Tsuchiya T., Kurita J., Snieckus V. (1977). General Photochemical Synthesis of 1H-1,2-Benzodiazepines from N-Iminoquinolinium Ylide Dimers. J. Org. Chem..

[49] Huisgen R., Grashey R., Krischke R. (1977). 1,3-Dipolare Cycloadditionen, 84. Additionen mit Chinolinium-, Isochinolinium- und Phenanthridinium-N-imid2). Justus Liebigs Ann. Chem..

[50] The Crystallographic Data Was Deposited at the Cambridge Crystallographic Data Center, CCDC 2122366. https://www.ccdc.cam.ac.uk/.

[51] Supranovich V.I., Vorob’ev A.Y., Borodkin G.I., Gatilov Y.V., Shubin V.G. (2016). Study on Selectivity in the Reaction of 2-Substituted Pyridinium- *N*-Imines with Dimethyl Acetylenedicarboxylate. Tetrahedron Lett..

[52] Tamura Y., Minamikawa J., Ikeda M. (1977). *O*-Mesitylenesulfonylhydroxylamine and Related Compounds-Powerful Aminating Reagents. Synthesis.

[53] Vorob’ev A.Y., Supranovich V.I., Borodkin G.I., Shubin V.G. (2017). New approach toward the synthesis of deuterated pyrazolo[1,5-*a*]pyridines and 1,2,4-triazolo[1,5-*a*]pyridines. Beilstein J. Org. Chem..

[54] Qu Z., Chen X., Yuan J., Qu L., Li X., Wang F., Ding X., Zhao Y. (2012). CuSO_4_·5H_2_O-catalyzed alkynylphosphonates formation—An efficient coupling reaction of terminal alkynes with *H*-phosphonates. Can. J. Chem..

[55] Egorova A.V., Viktorov N.B., Starova G.L., Svintsitskaya N.I., Garabadziu A.V., Dogadina A.V. (2017). BF_3_·Et_2_O catalyzed intramolecular cyclization of diethyl 2-(dialkoxyphosphorylethynyl)-2-arylaminomalonates to 3-phosphonylated indoles. Tetrahedron Lett..

[56] Kruglov S.V., Ignat’ev V.M., Ionin B.I., Petrov A.A. (1973). Synthesis of Symmetrical and Mixed Diphosphonic Esters. J. General Chem. USSR.

[57] Oakdale J.S., Sit R.K., Fokin V.V. (2014). Ruthenium-Catalyzed Cycloadditions of 1-Haloalkynes with Nitrile Oxides and Organic Azides: Synthesis of 4-Haloisoxazoles and 5-Halotriazoles. Chem.-Eur. J..

[58] Marian A., Maas G. (2020). Diethyl (iodoethynyl)phosphonate and (iodoethynyl)diphenylphosphane oxide: Crystal structures and some cycloaddition reactions. Z. Nat. B.

[59] Sheldrick G.M. (2015). Crystal structure refinement with SHELXL. Acta Cryst..

[60] Sheldrick G.M. SADABS 1996, Program for Empirical Adsorption Correction. https://www.scienceopen.com.

[61] Sheldrick G.M. (2015). SHELXT-Integrated space-group and crystal-structure determination. Acta Cryst. Sect. A Found. Cryst..

