# Supplementary material for: Oxidative [3+2]Cycloaddition of Alkynylphosphonates with Heterocyclic N-Imines: Synthesis of Pyrazolo[1,5-a]Pyridine-3-phosphonates
Source: Molecules. 2022 Nov 16;27(22):7913. doi: 10.3390/molecules27227913 (PMC9694626; doi:10.3390/molecules27227913)

Supporting information

Oxidative [3+2]cycloaddition of alkynylphosphonates to heterocyclic *N*-imines: synthesis of pyrazolo[1,5-*a*]pyridine-3-phosphonates

Igor R. Philippov[1], Yuriy V. Gatilov[1], Alina A. Sonina[1], Aleksey Yu. Vorob'ev[1,2*]

[1]Novosibirsk Institute of Organic Chemistry SB RAS, 630090, Novosibirsk, Russian Federation, 9 Lavrentiev avenue

[2]Novosibirsk State University, 630090, Novosibirsk, Russian Federation, 1 Pirogova street

e-mail: vor@nioch.nsc.ru

Table of content:

**Crystallographic data**	**S2**
**Copies of NMR spectra.**	**S3**

Crystallographic data

Crystallographic data for diethyl (2,7-diphenylpyrazolo[1,5-a]pyridin-3-yl)phosphonate (3d). Formula $C_{23}H_{19}N_2O_3P$ (without solvent), *M* = 402.37, triclinic, space group *P*-1, *a* = 7.7535(4), *b* = 10.9029(7), *c* = 14.0556(9) Å, α = 94.892(3), *β* = 97.428(3), γ = 103.153(3)°, *V* = 1139.19(12) $Å^3$, *Z* = 2, D_{calc} = 1.173 $g{\cdot}cm^{-3}$, μ = 0.144 mm^{-1}, min/max transmission = 0.8816/0.9391, θ_{max} = 26.1°, measured 21669, unique 4514 (R_{int} = 0.0467), observed [$I_o > 2\sigma(I)$] 3004 reflections, R_1 = 0.0481 (I_o), wR_2 = 0.1339 (all), Gof = 1.023, $\Delta\rho_{max}$ = 0.32, $\Delta\rho_{min}$ = -0.31 $e{\cdot}Å^{-3}$. CCDC 2001188.

Crystallographic data for diethyl (2-phenoxy[1,5-a]pyridin-3-yl)phosphonate (5u). Formula C17H19N2O4P, M = 346.31,monoclinic space group P21/n, a = 9.9416(5), b = 12.9865(5), c = 13.7882(8) Å, β = 92.659(2), V = 1778.23(15) Å3, Z = 4, Dcalc = 1.294 g·cm-3, μ = 0.177 mm-1, min/max transmission = 0.756/0.801, θmax = 27.2°, measured 23274, unique 3933 (Rint = 0.047), observed [Io > 2σ(I)] 3185 reflections, R1 = 0.071 (Io), wR2 = 0.211 (all), Gof = 1.05, Δρmax = 0.51, Δρmin = -0.50 e·Å-3. CCDC 2122366.

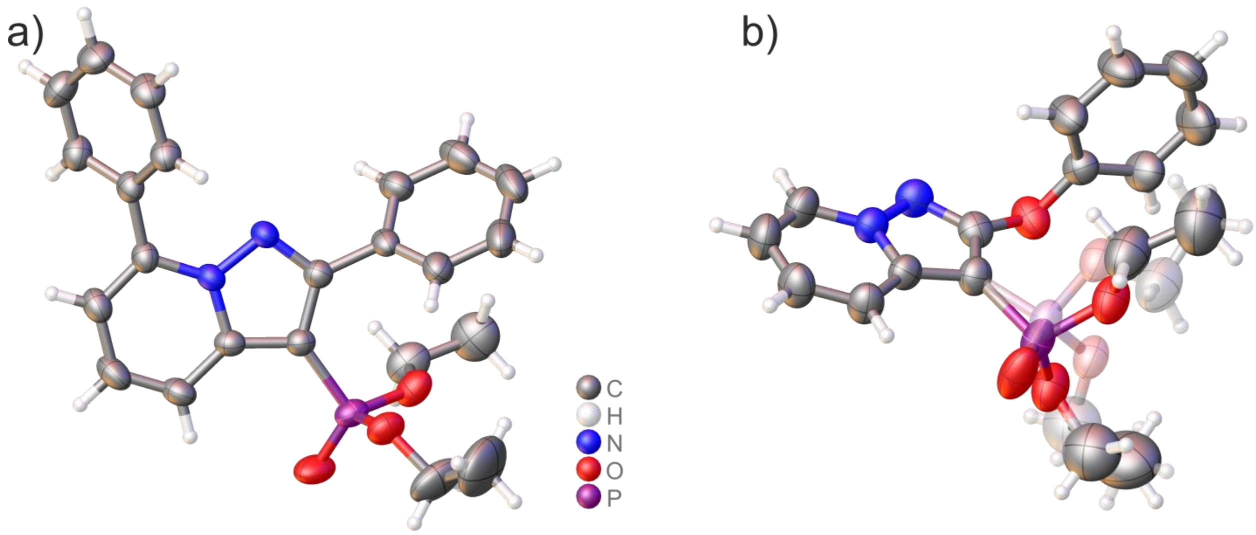

Figure S1. Crystal structures of pyrazolopyridines **3d** (A) and **5u** (B)

Copies of NMR spectra.

Diethyl (2-phenylpyrazolo[1,5-*a*]pyridin-3-yl)phosphonate (3a) ^{1}H NMR.

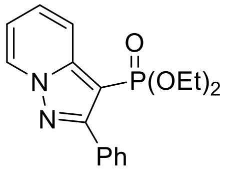

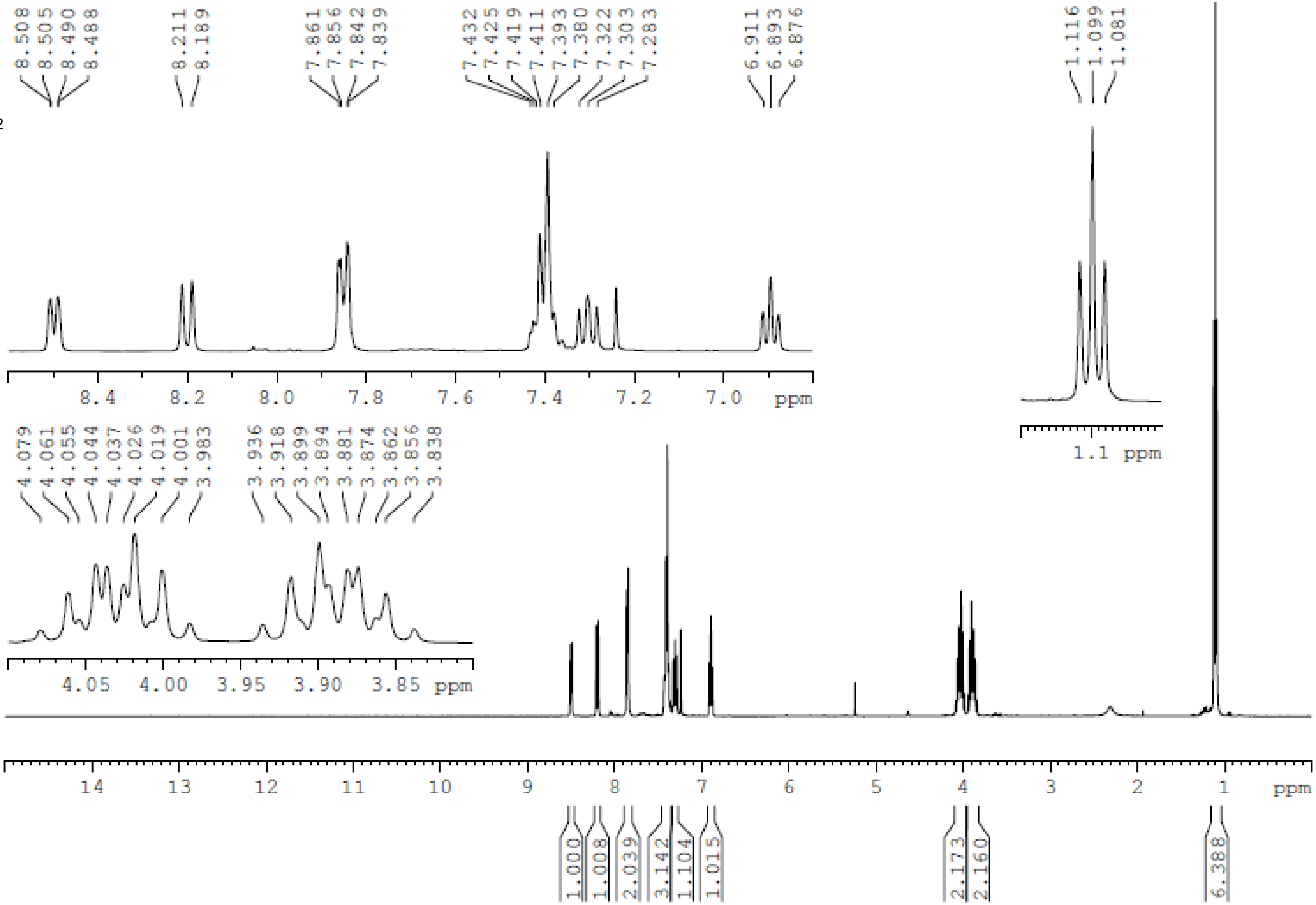

Diethyl (2-phenylpyrazolo[1,5-*a*]pyridin-3-yl)phosphonate (3a) ^{13}C NMR.

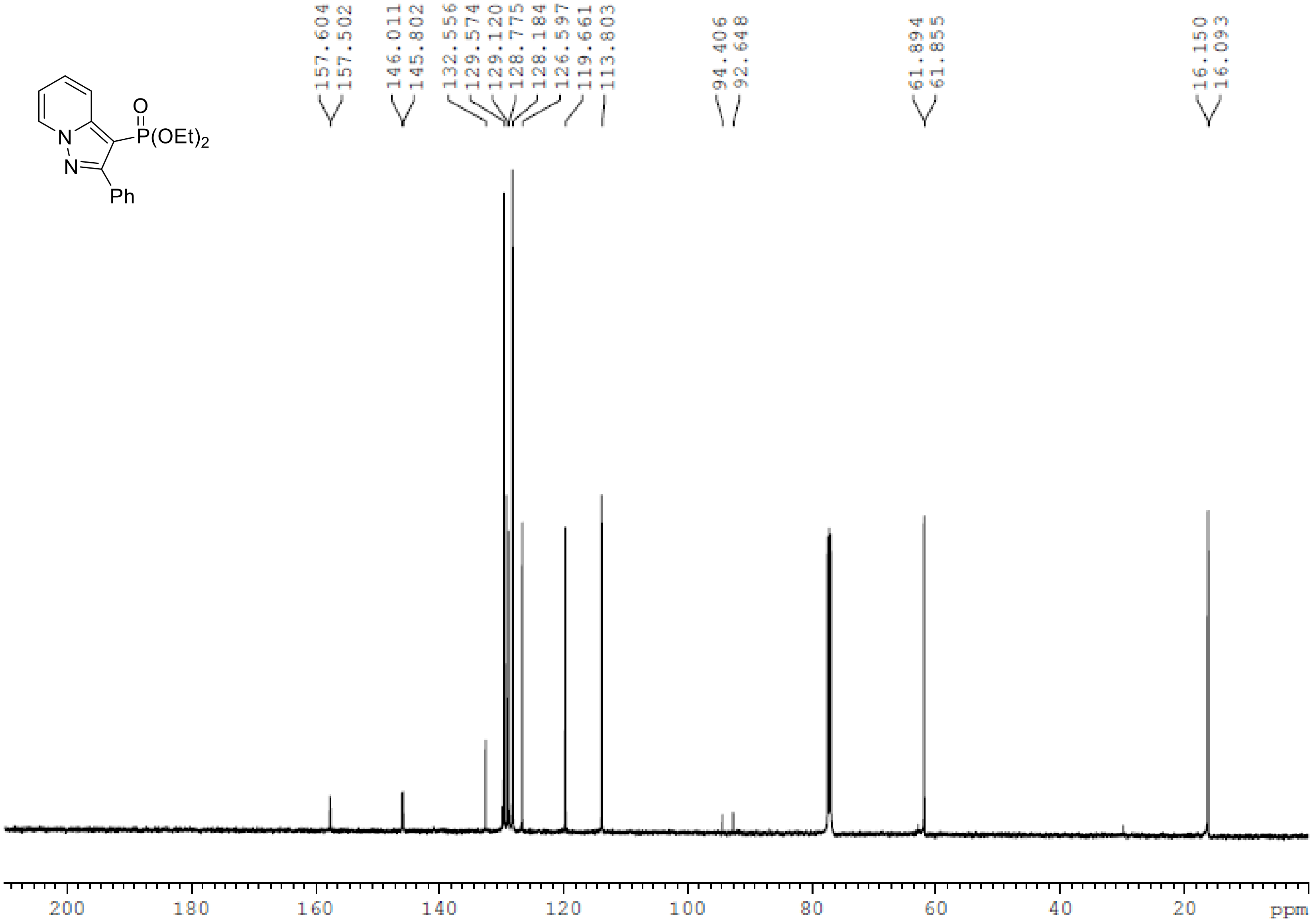

Diethyl (2-phenylpyrazolo[1,5-*a*]pyridin-3-yl)phosphonate (3a) ^{31}P NMR.

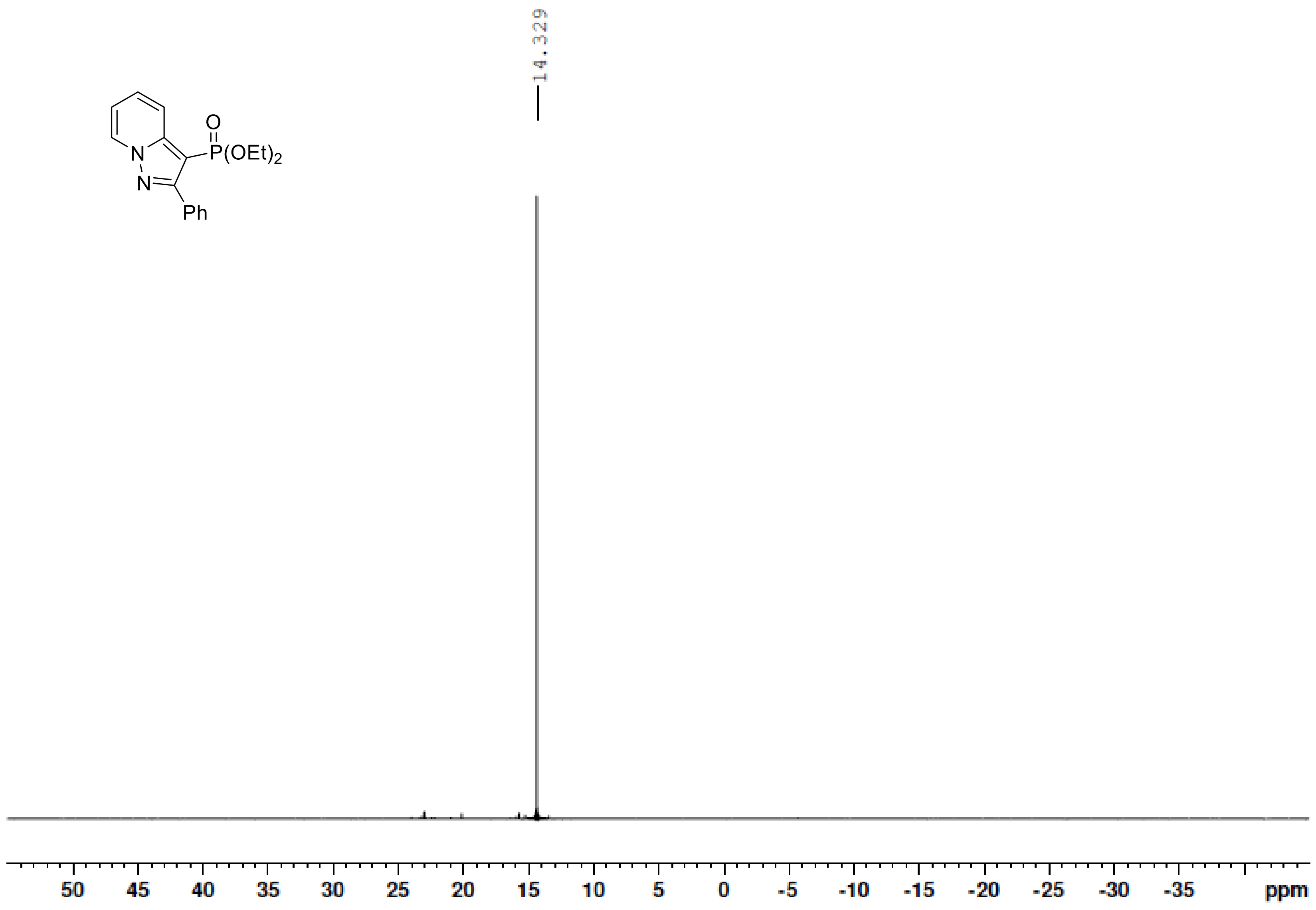

Diethyl (5-methyl-2-phenylpyrazolo[1,5-a]pyridin-3-yl)phosphonate (3b) ^{1}H NMR.

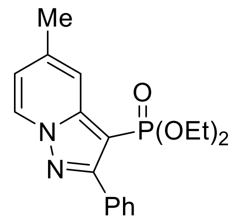

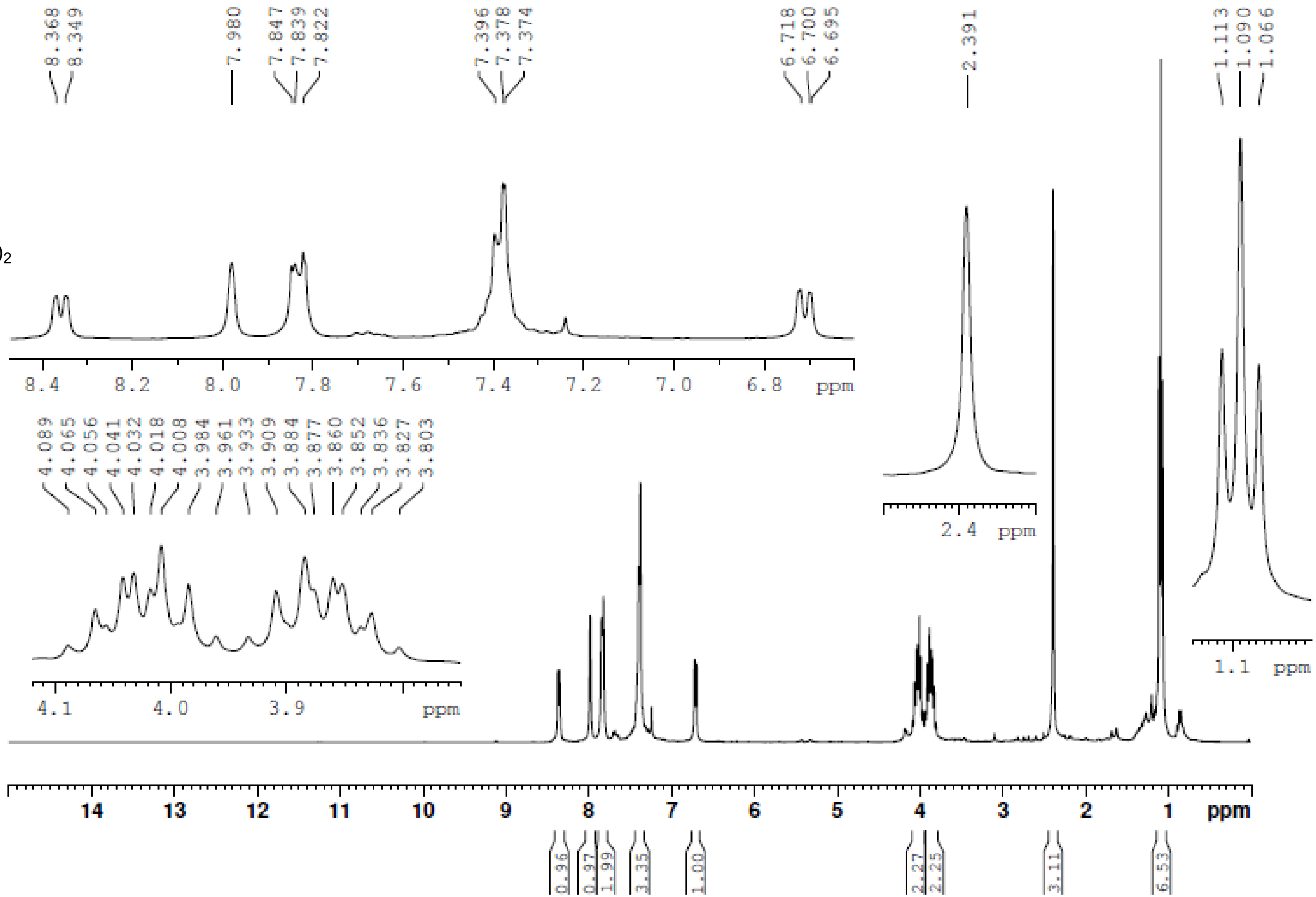

Diethyl (5-methyl-2-phenylpyrazolo[1,5-a]pyridin-3-yl)phosphonate (3b) ^{13}C NMR.

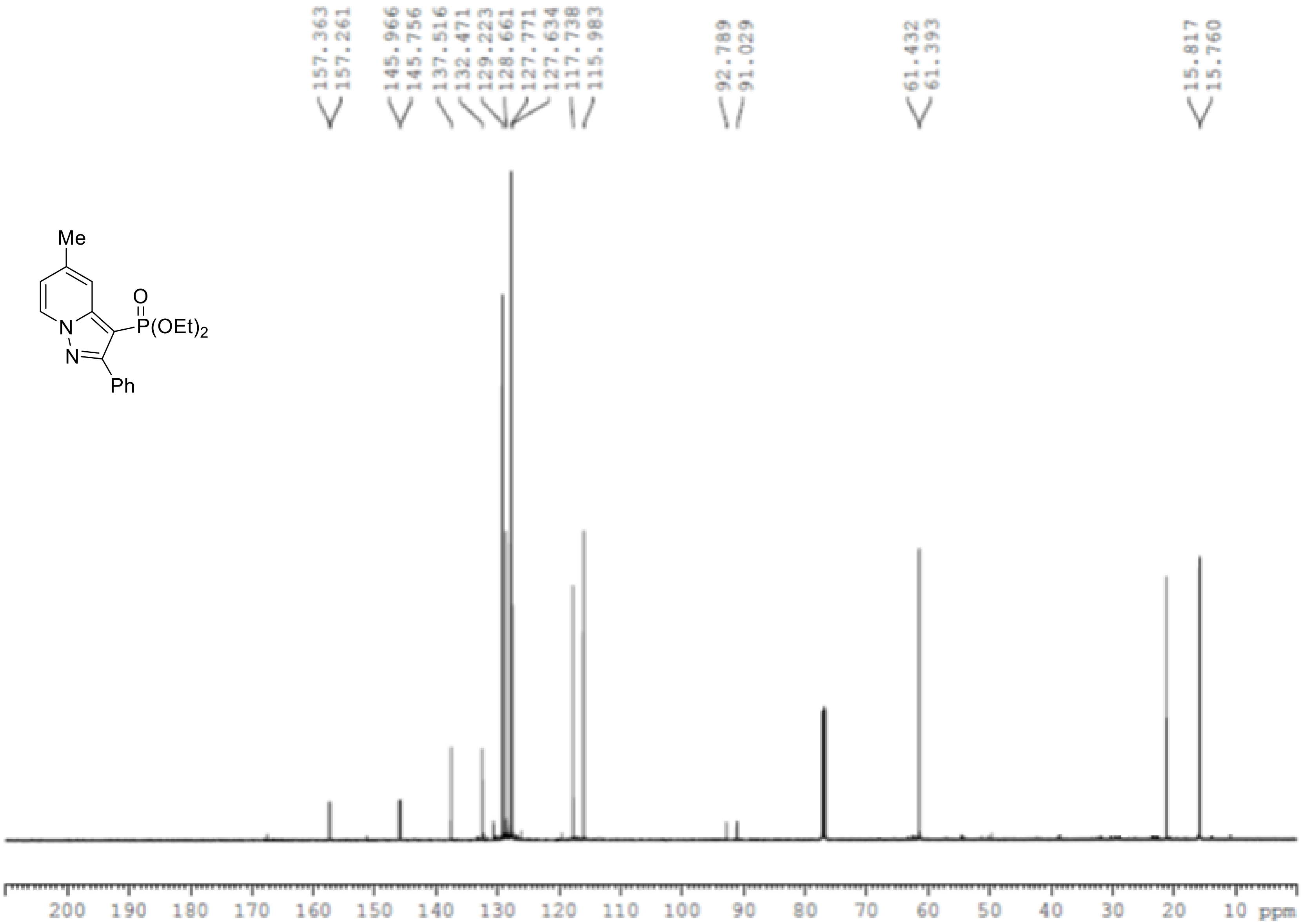

Diethyl (5-methyl-2-phenylpyrazolo[1,5-a]pyridin-3-yl)phosphonate (3b) ^{1}H NMR.

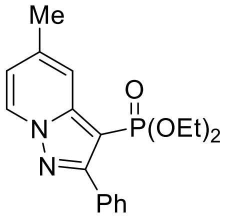

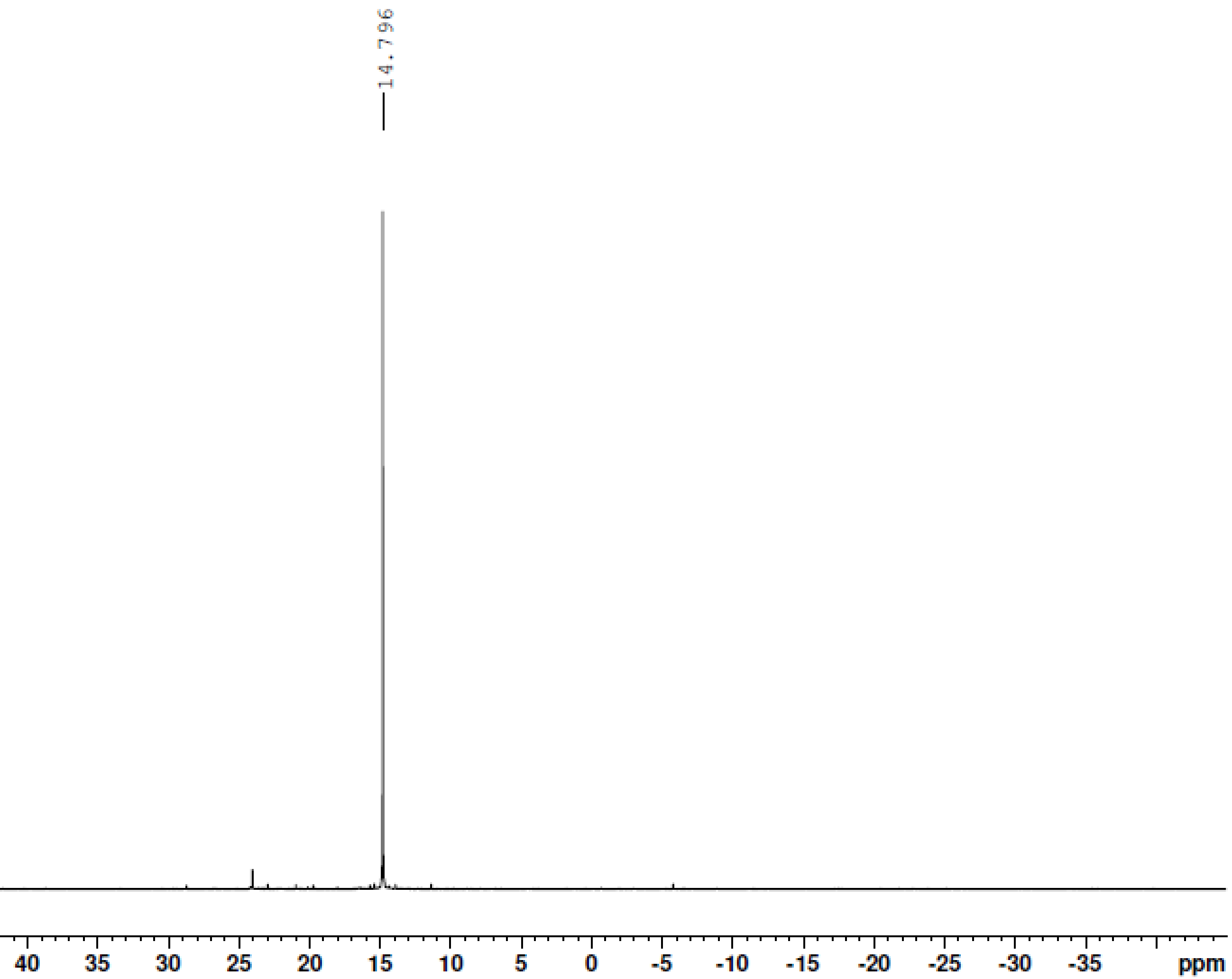

Diethyl (7-methyl-2-phenylpyrazolo[1,5-a]pyridin-3-yl)phosphonate (3c) ^{1}H NMR

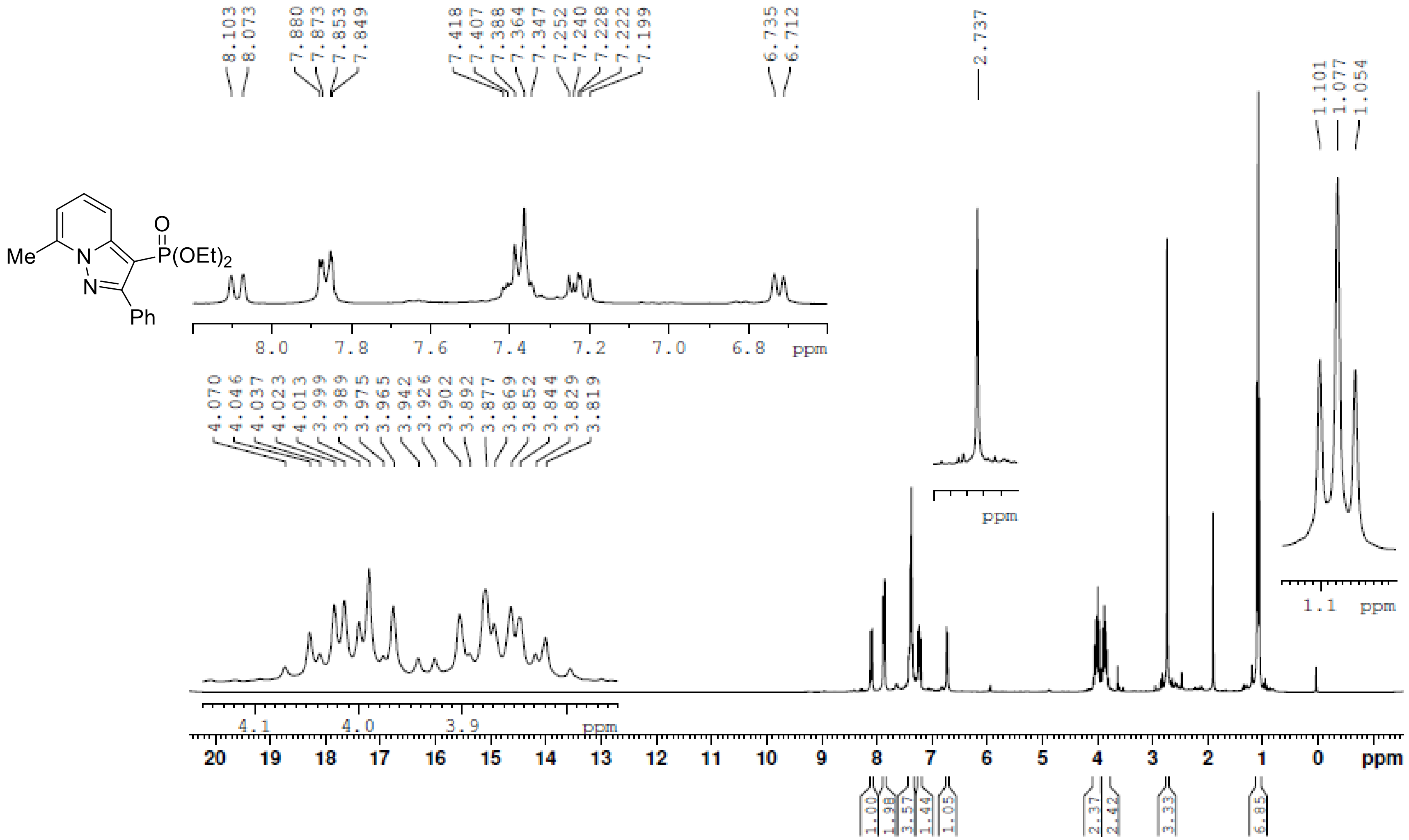

Diethyl (7-methyl-2-phenylpyrazolo[1,5-a]pyridin-3-yl)phosphonate (3c) ^{13}C NMR

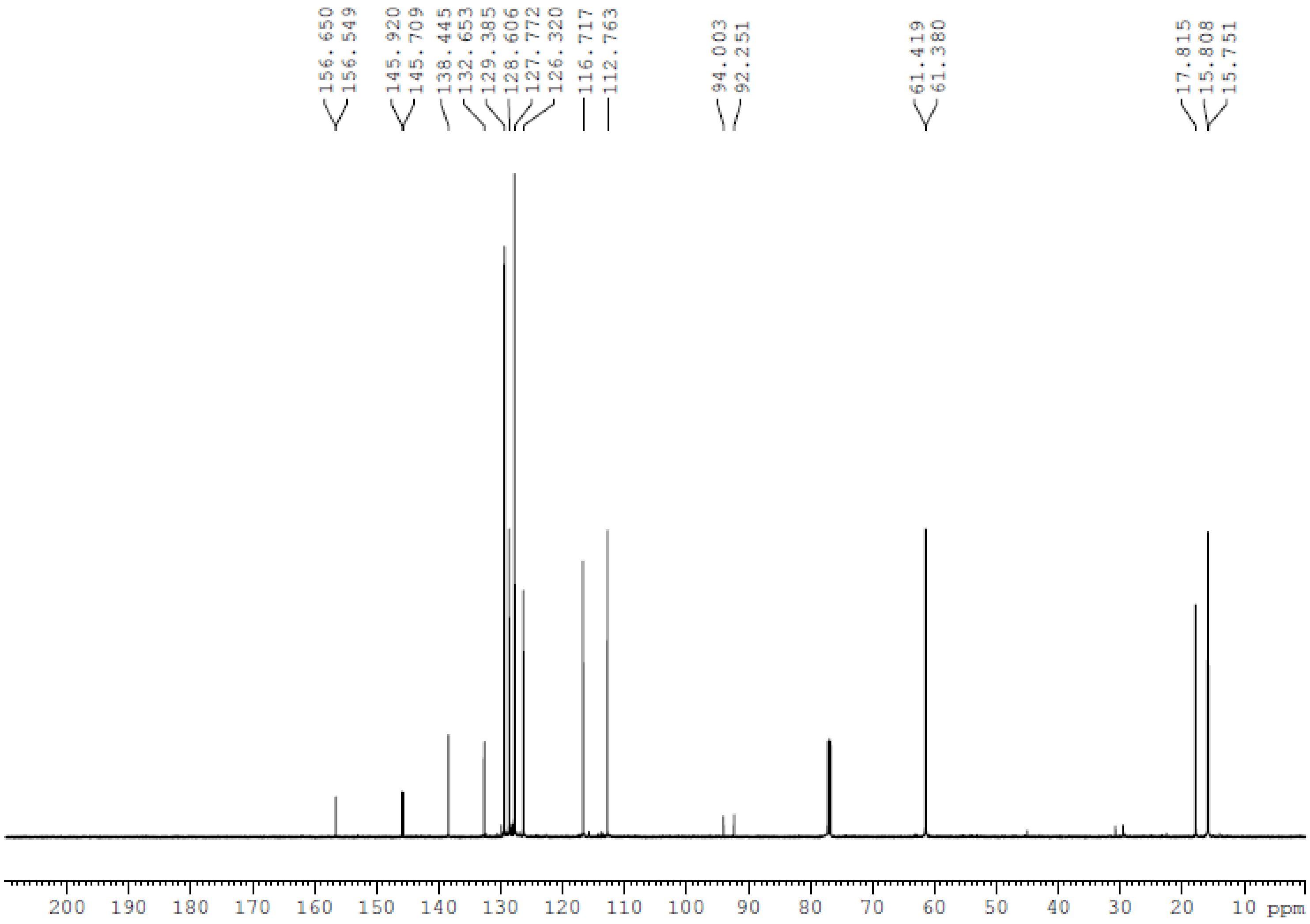

Diethyl (7-methyl-2-phenylpyrazolo[1,5-a]pyridin-3-yl)phosphonate (3c) ^{31}P NMR

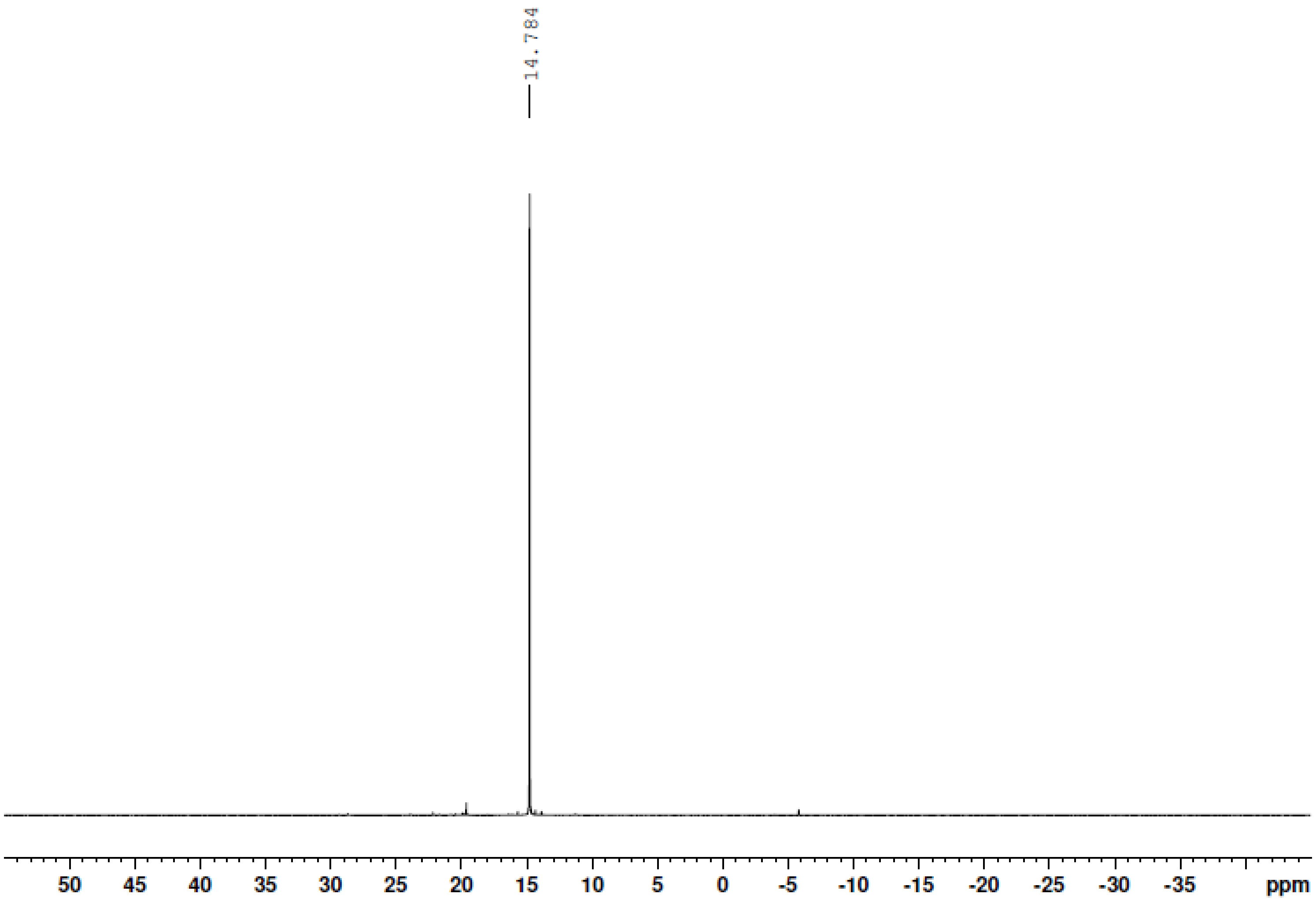

Diethyl (2,7-diphenylpyrazolo[1,5-a]pyridin-3-yl)phosphonate (3d) ^{1}H NMR.

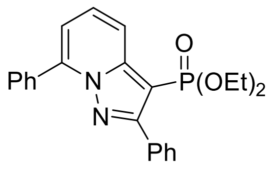

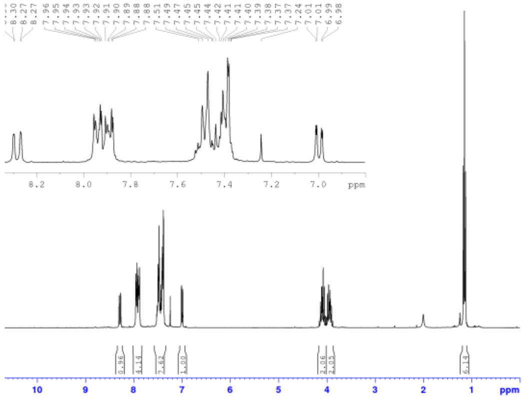

Diethyl (2,7-diphenylpyrazolo[1,5-a]pyridin-3-yl)phosphonate (3d) ^{13}C NMR.

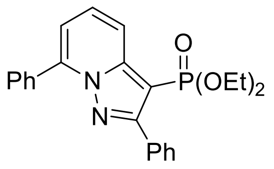

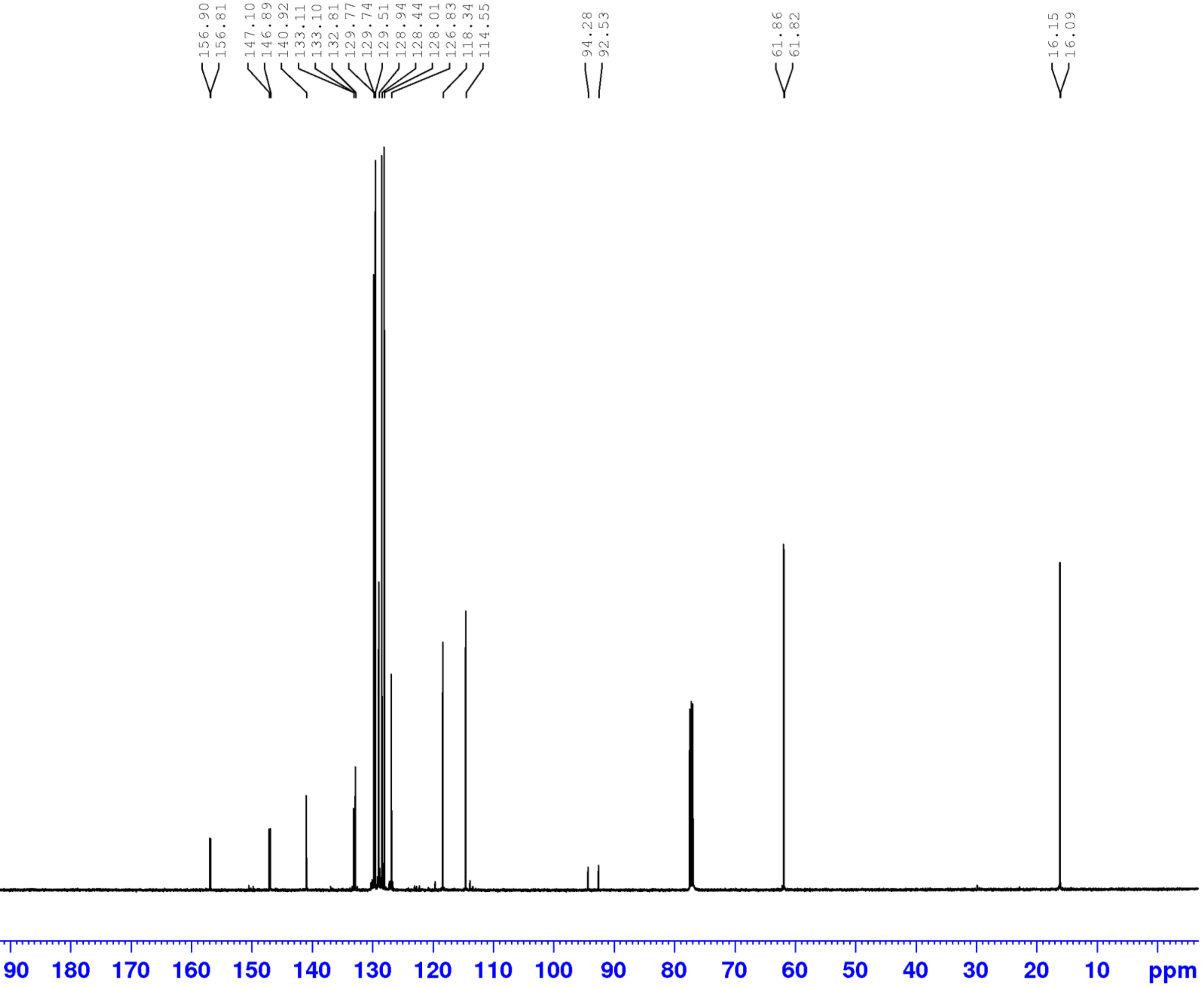

Diethyl (2,7-diphenylpyrazolo[1,5-a]pyridin-3-yl)phosphonate (3d) ^{31}P NMR.

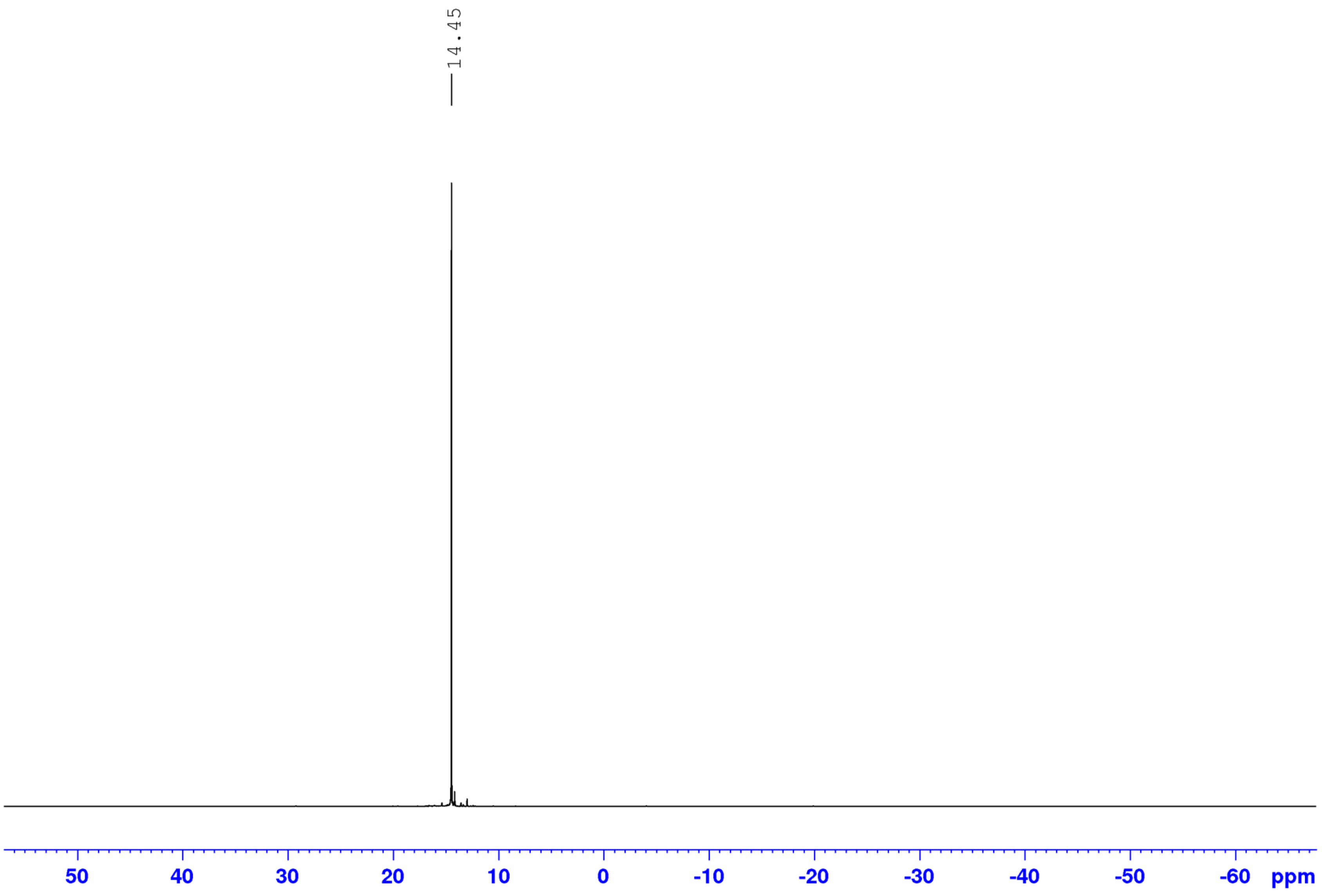

Ethyl 3-(diethoxyphosphoryl)-2-phenylpyrazolo[1,5-a]pyridine-5-carboxylate ^{1}H NMR (3e).

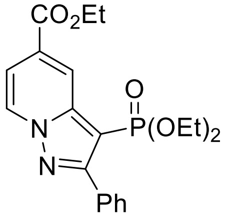

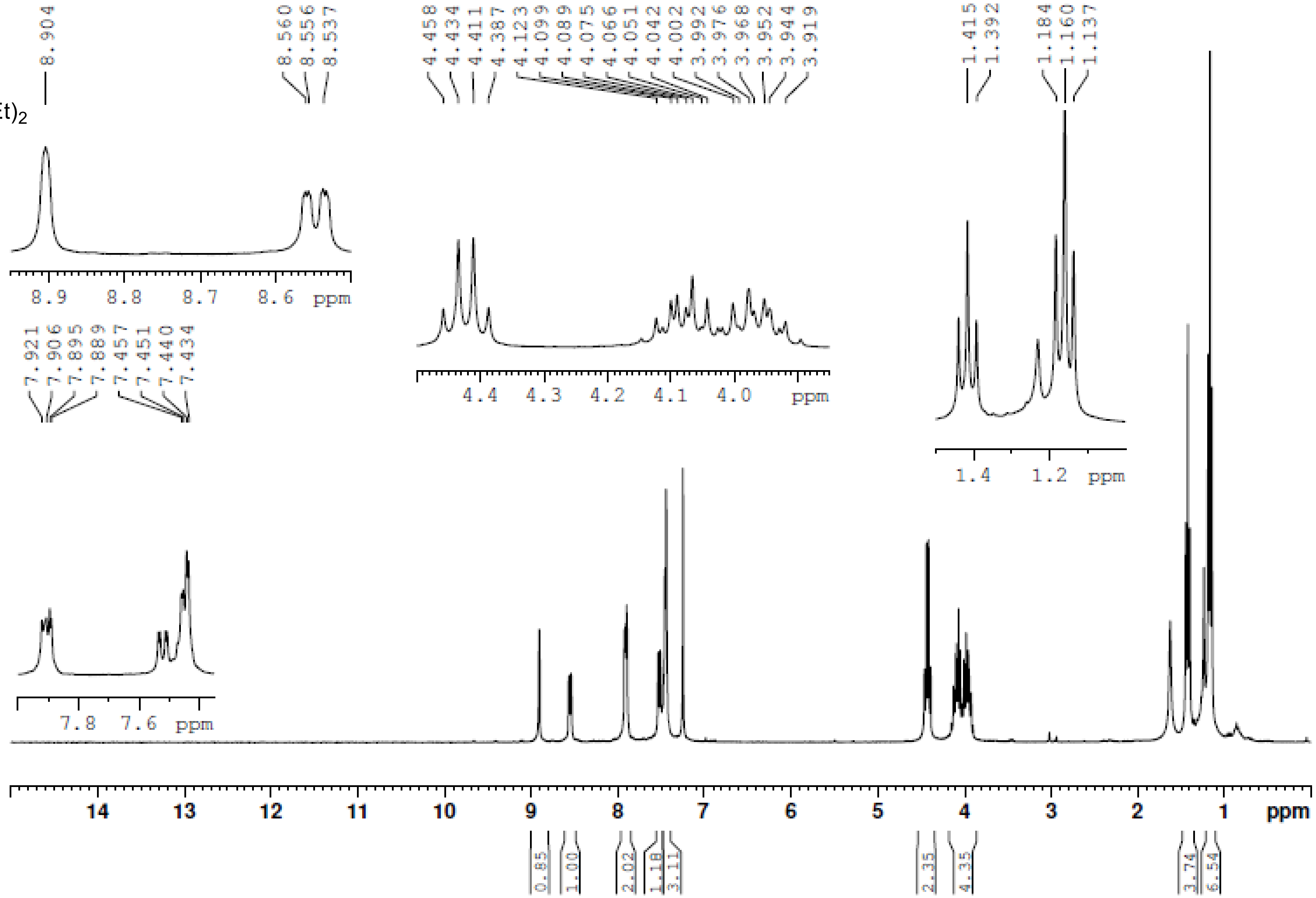

Ethyl 3-(diethoxyphosphoryl)-2-phenylpyrazolo[1,5-a]pyridine-5-carboxylate ^{13}C NMR (3e).

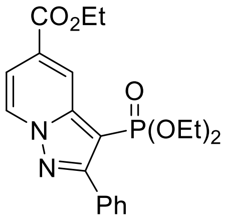

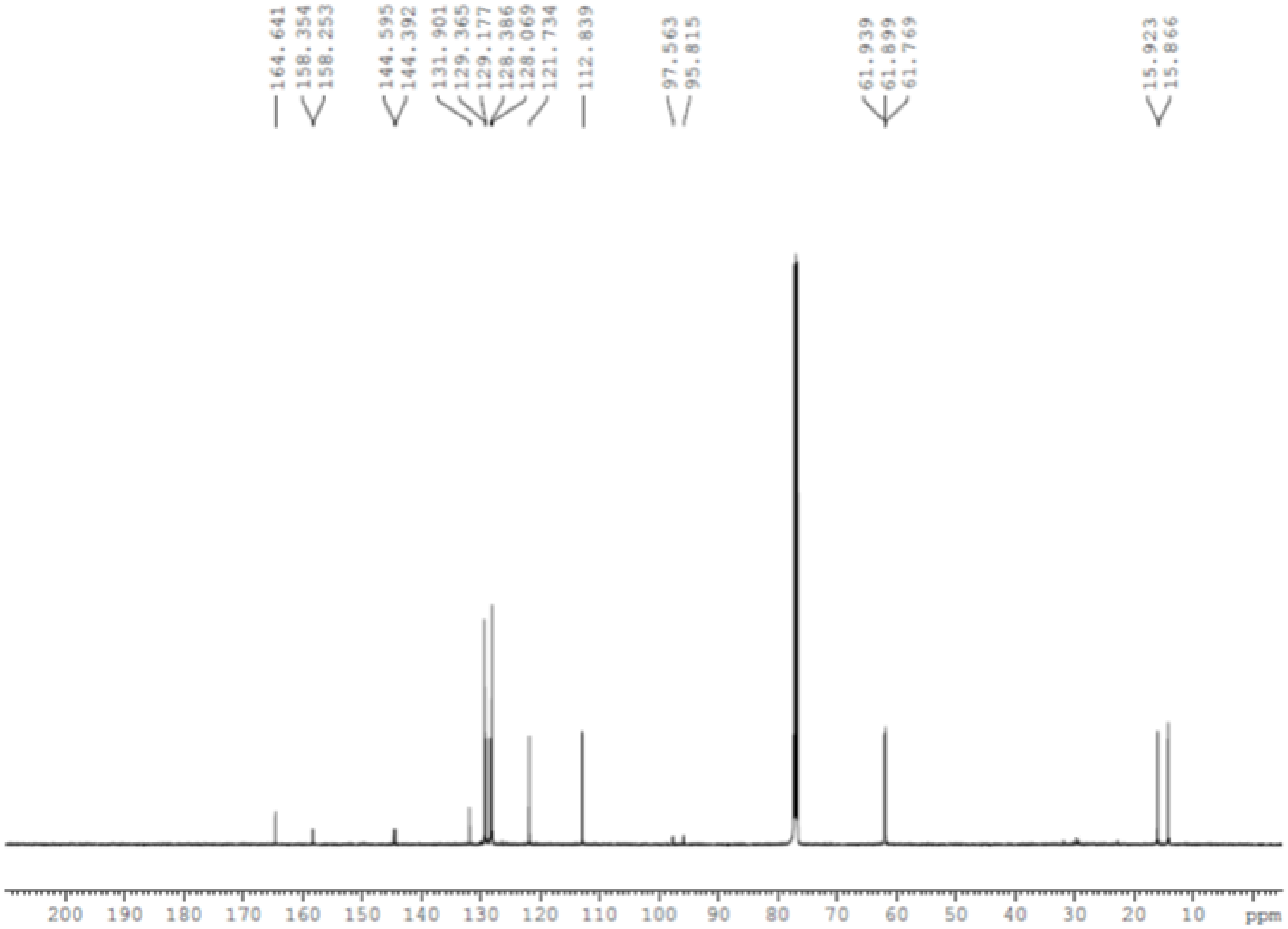

Ethyl 3-(diethoxyphosphoryl)-2-phenylpyrazolo[1,5-a]pyridine-5-carboxylate ^{31}P NMR (3e).

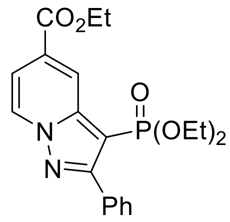

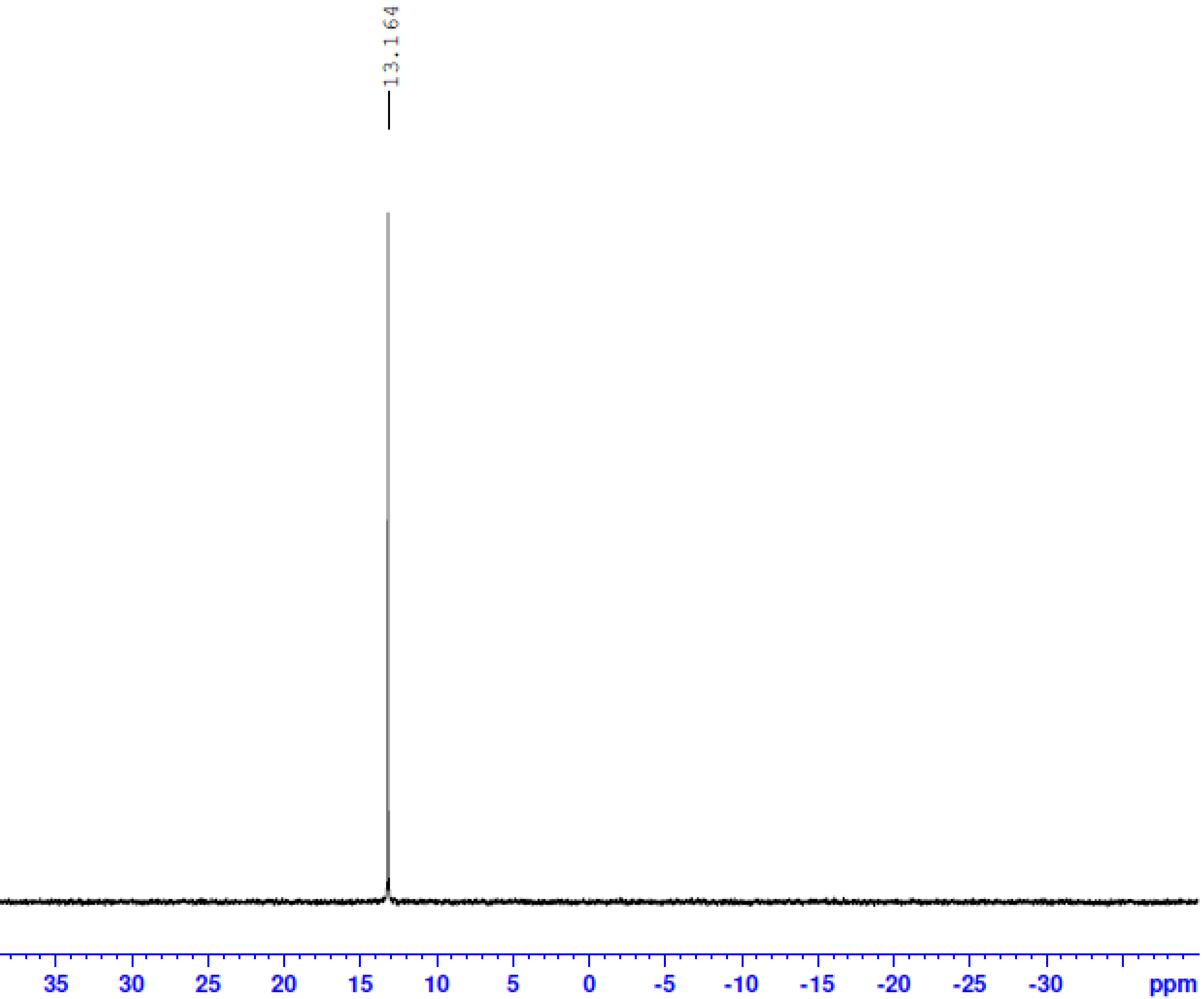

Diethyl (5-methoxy-2-phenylpyrazolo[1,5-a]pyridin-3-yl)phosphonate (3f) ^{1}H NMR.

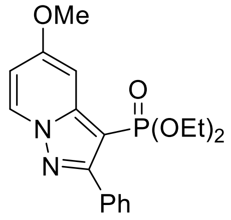

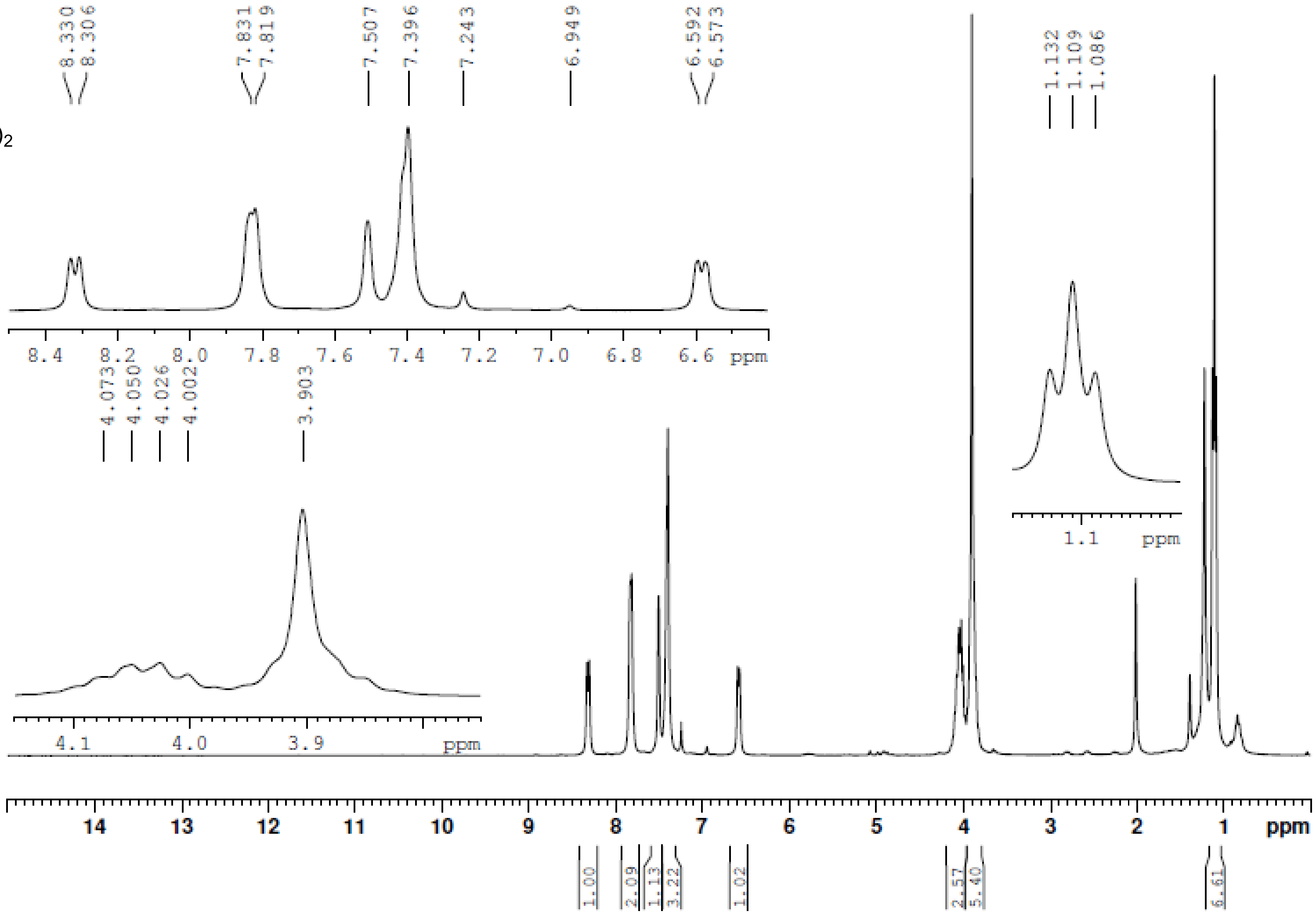

Diethyl (5-methoxy-2-phenylpyrazolo[1,5-a]pyridin-3-yl)phosphonate (3f) ^{13}C NMR.

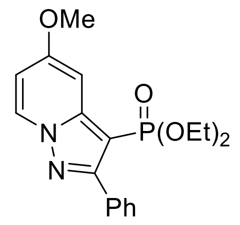

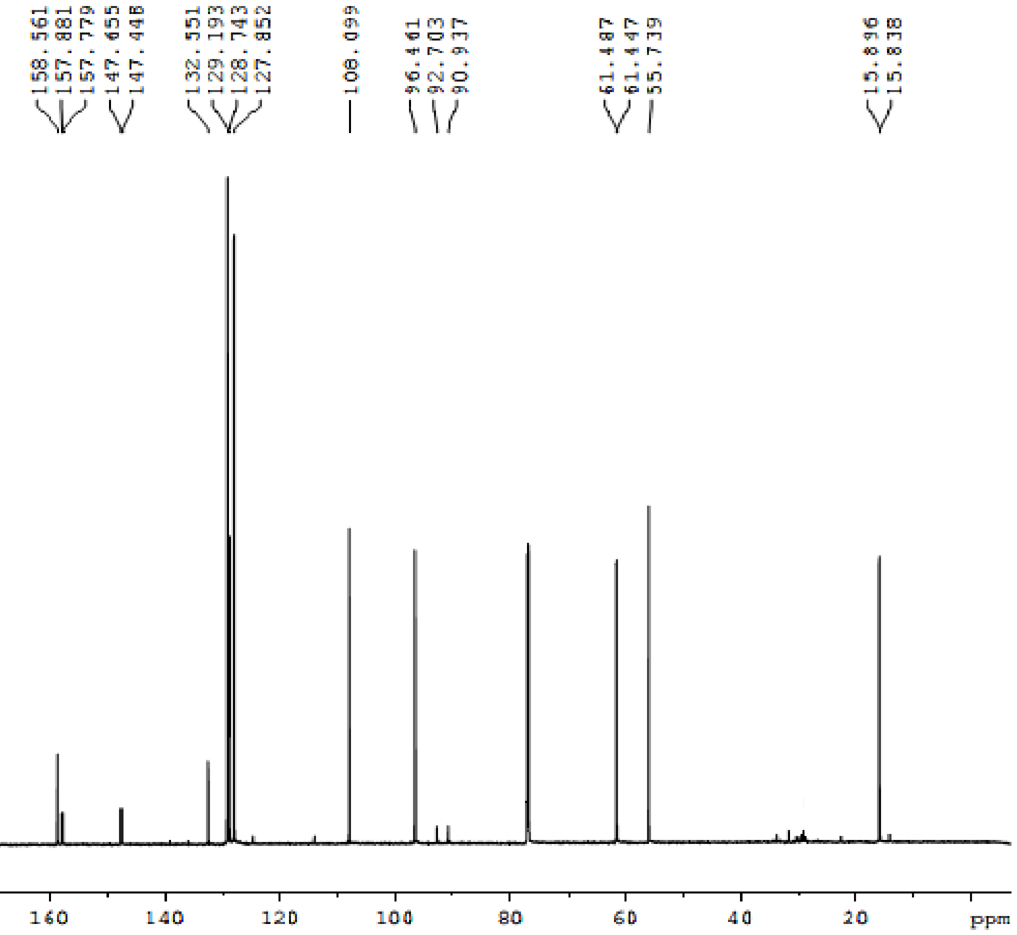

Diethyl (5-methoxy-2-phenylpyrazolo[1,5-a]pyridin-3-yl)phosphonate (3f) ^{31}P NMR.

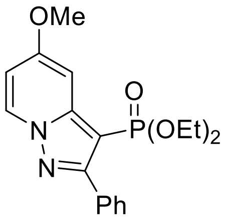

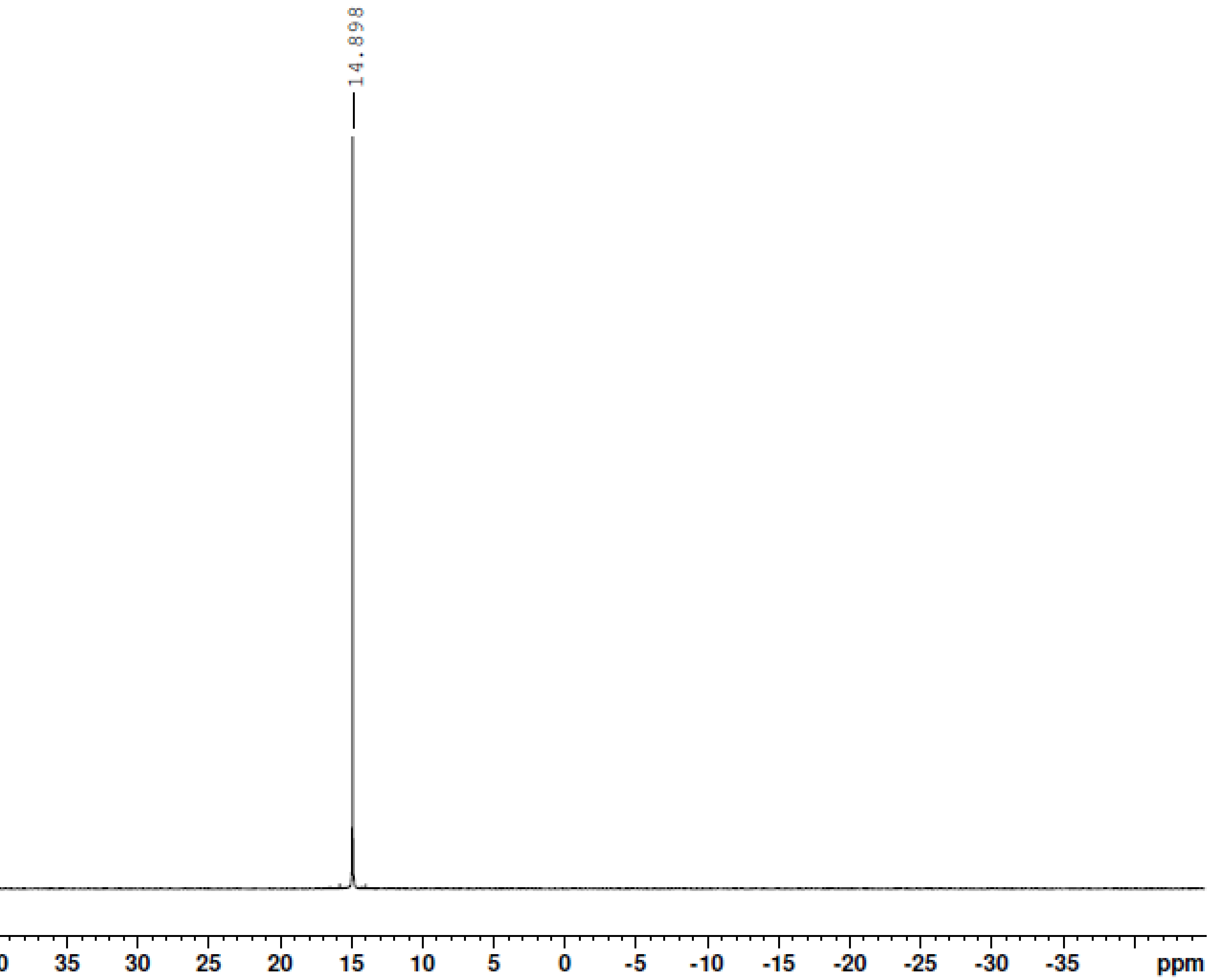

Diethyl (2-butylpyrazolo[1,5-a]pyridin-3-yl)phosphonate (3g) ^{1}H NMR.

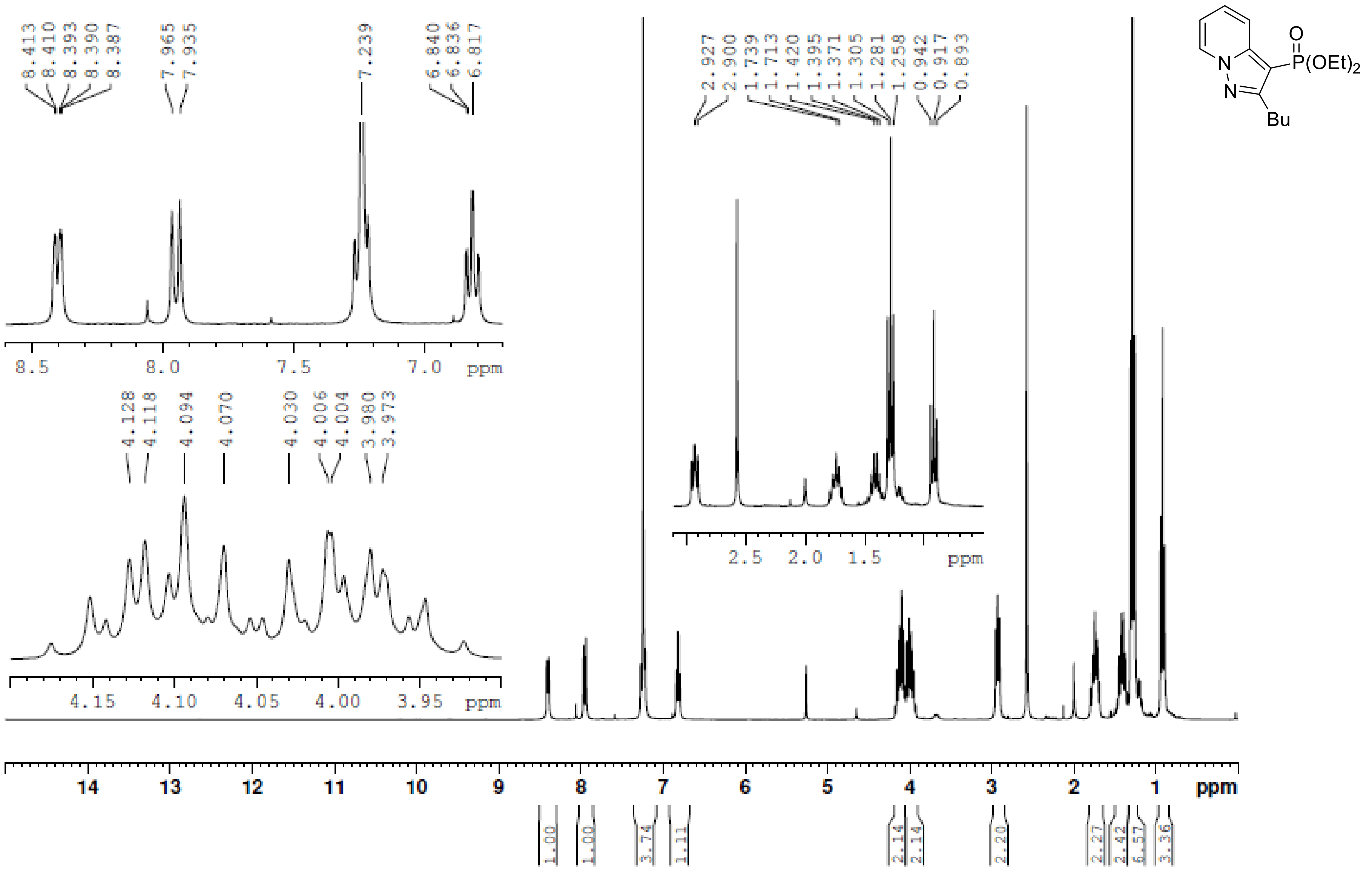

Diethyl (2-butylpyrazolo[1,5-a]pyridin-3-yl)phosphonate (3g) ^{13}C NMR.

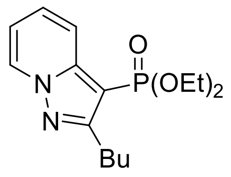

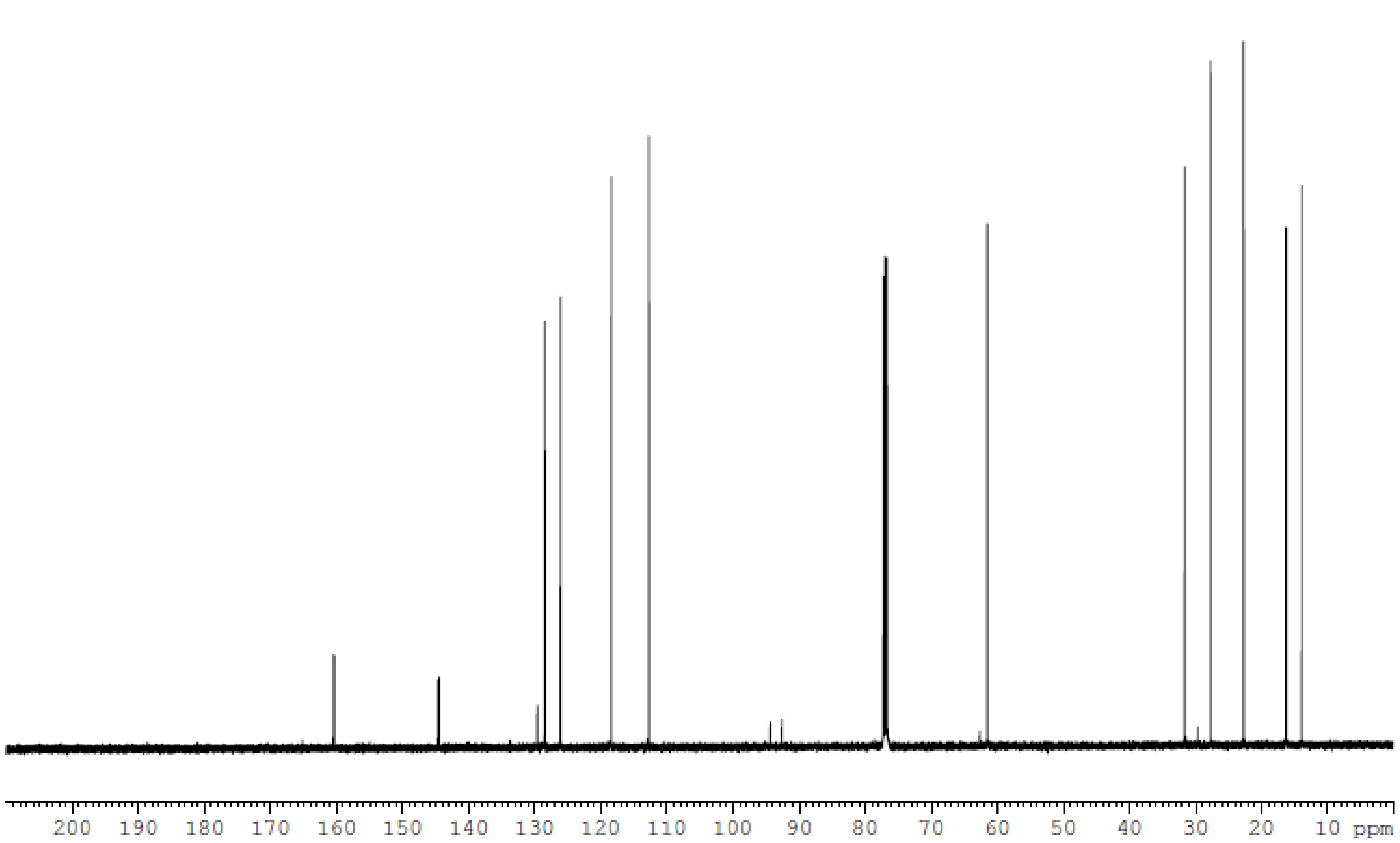

Diethyl (2-butylpyrazolo[1,5-a]pyridin-3-yl)phosphonate (3g) ^{31}P NMR.

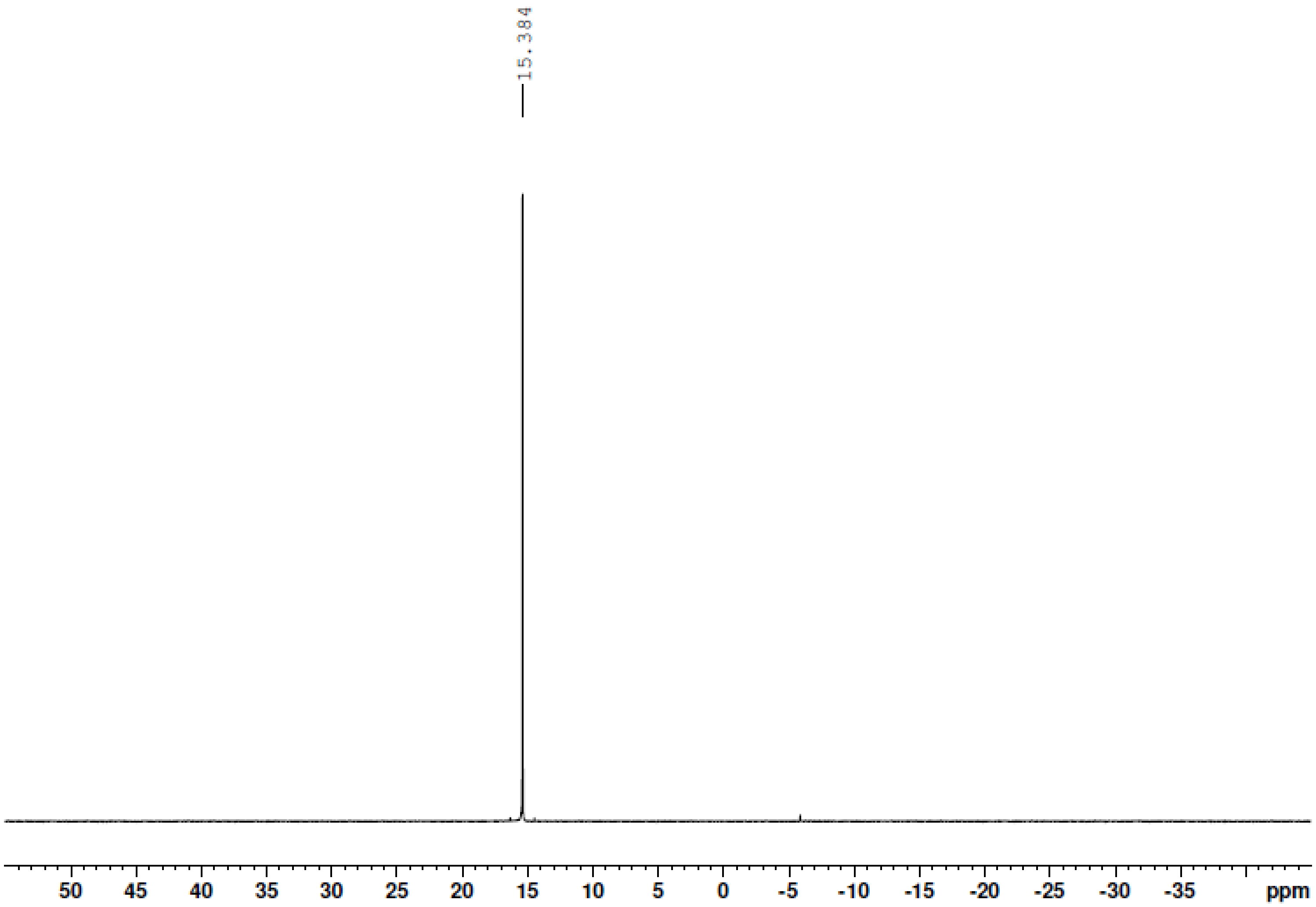

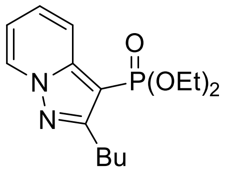

Diethyl (2-*tert*-butylpyrazolo[1,5-a]pyridin-3-yl)phosphonate (3h) ^{1}H NMR.

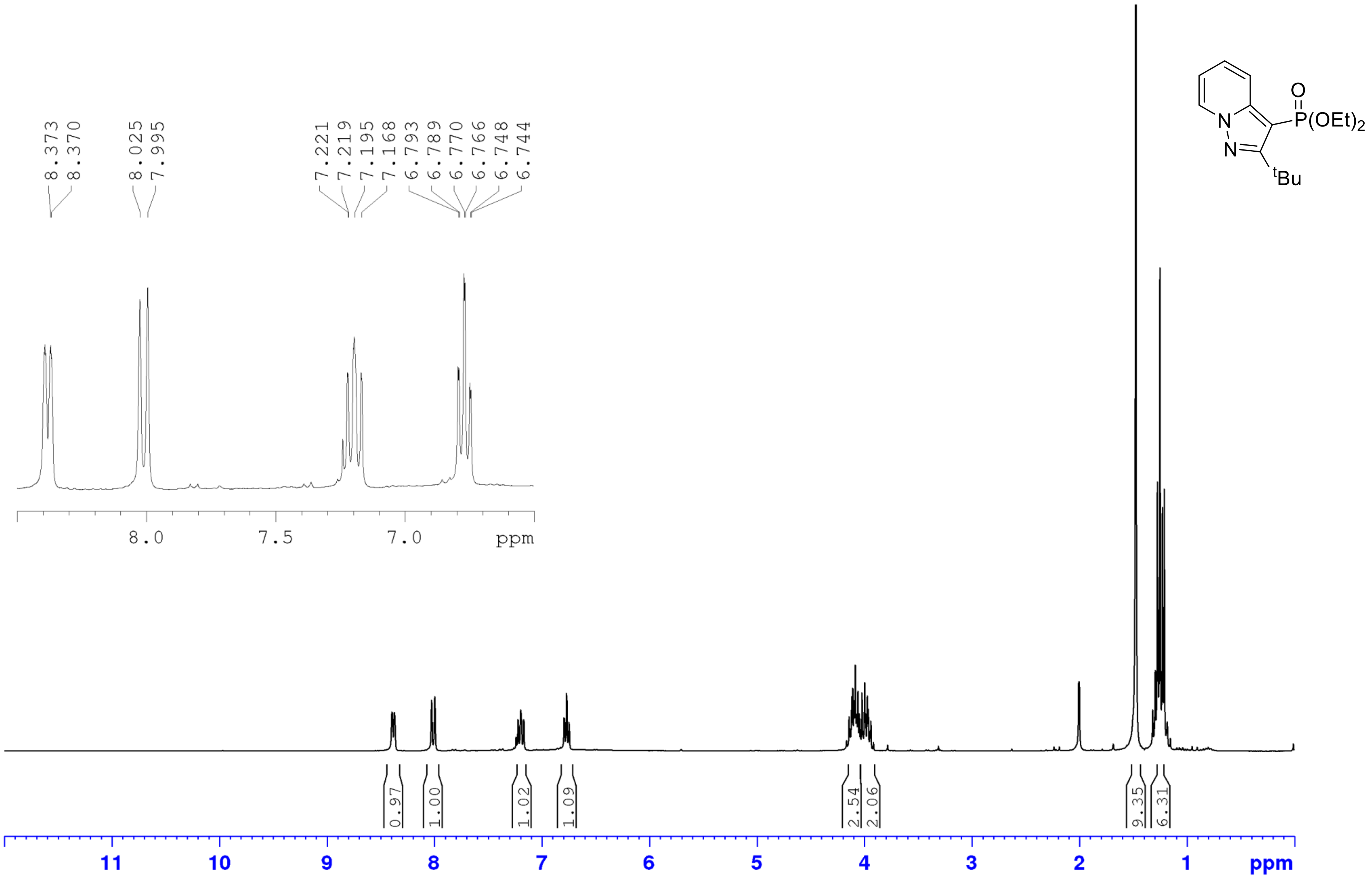

Diethyl (2-*tert*-butylpyrazolo[1,5-a]pyridin-3-yl)phosphonate (3h) ^{13}C NMR.

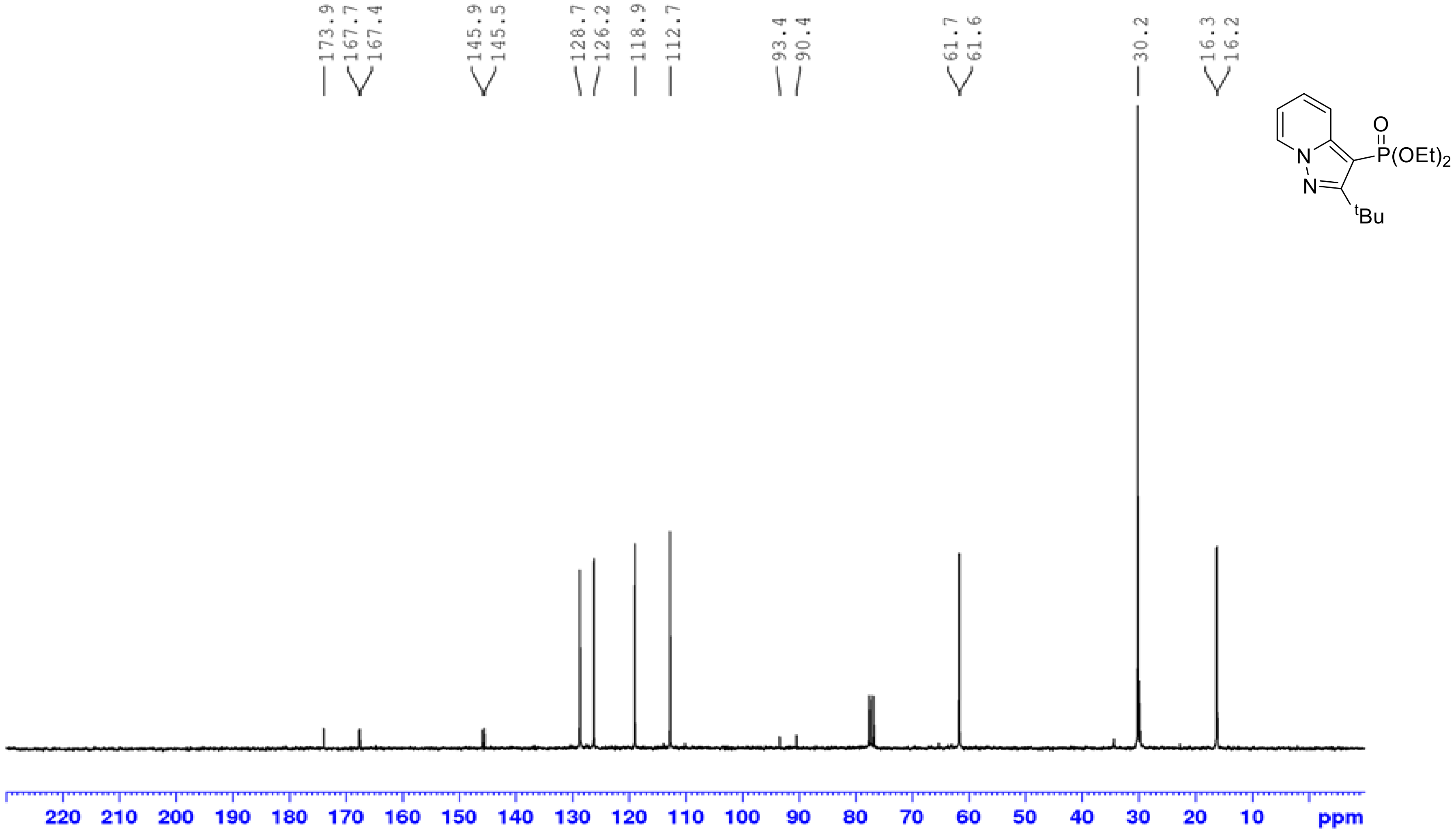

Diethyl (2-*tert*-butylpyrazolo[1,5-a]pyridin-3-yl)phosphonate (3h) ^{31}P NMR.

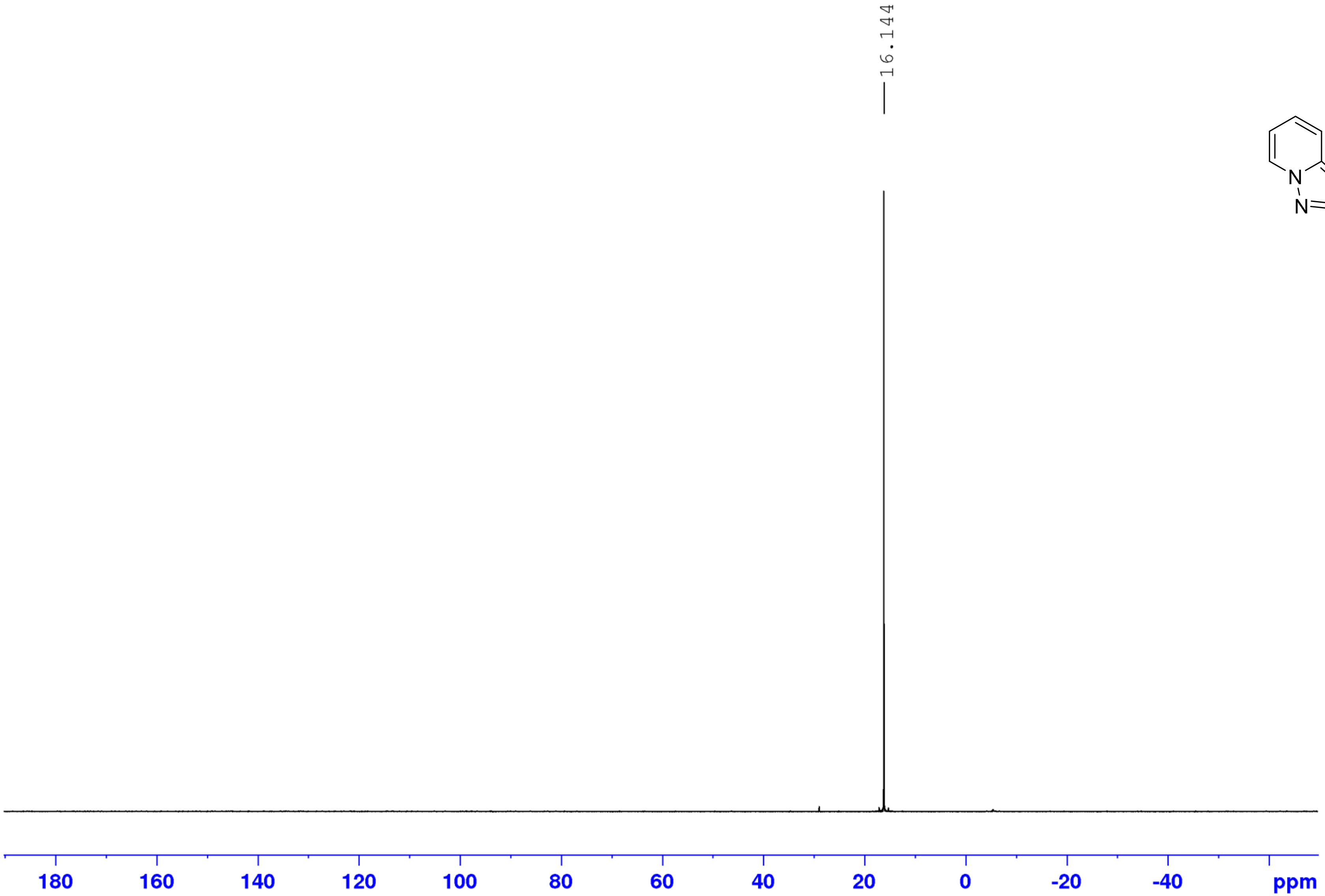

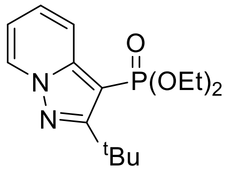

Diethyl (2-cyclopropylpyrazolo[1,5-a]pyridin-3-yl)phosphonate ^{1}H NMR(3i).

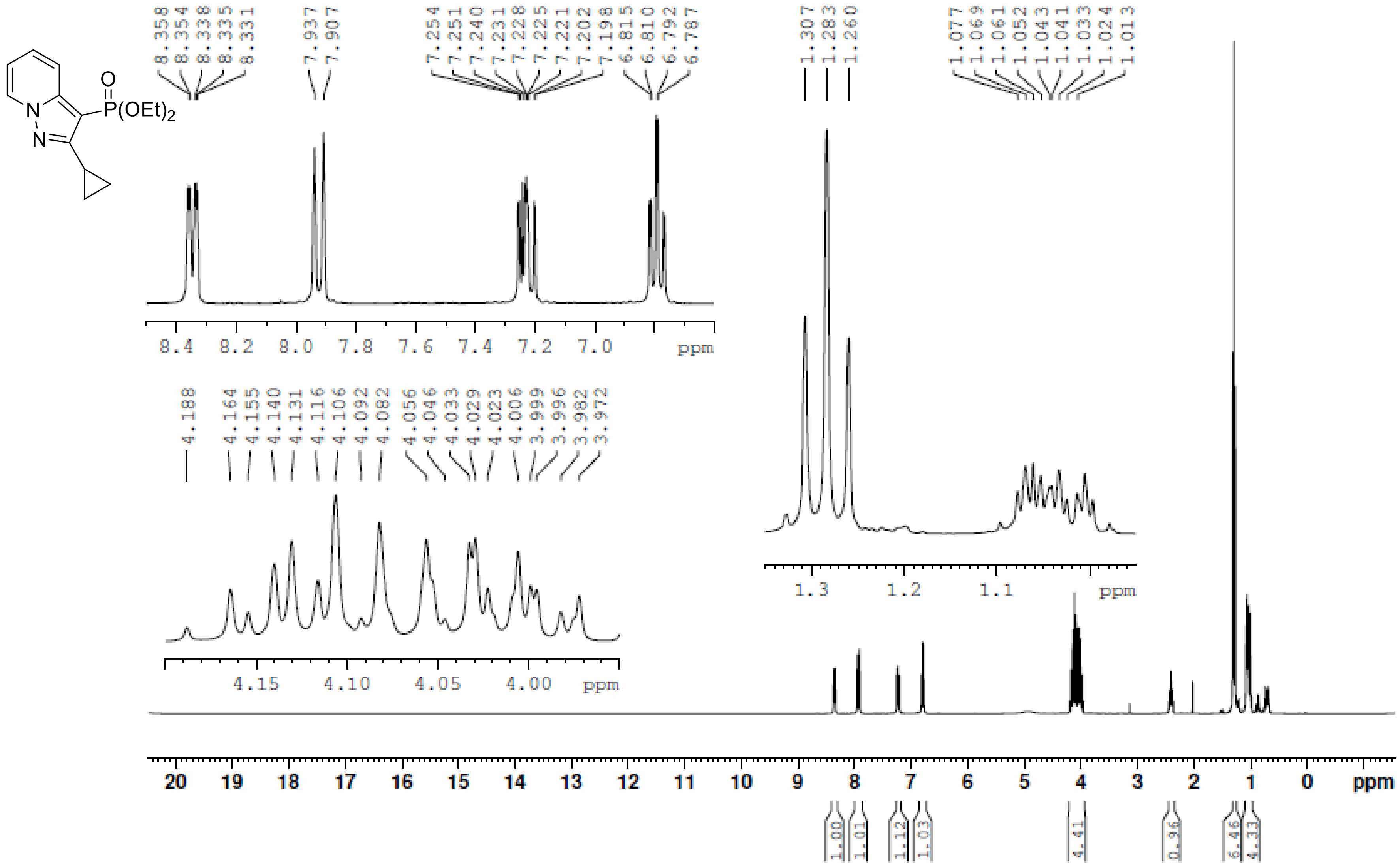

Diethyl (2-cyclopropylpyrazolo[1,5-a]pyridin-3-yl)phosphonate ^{13}C NMR(3i).

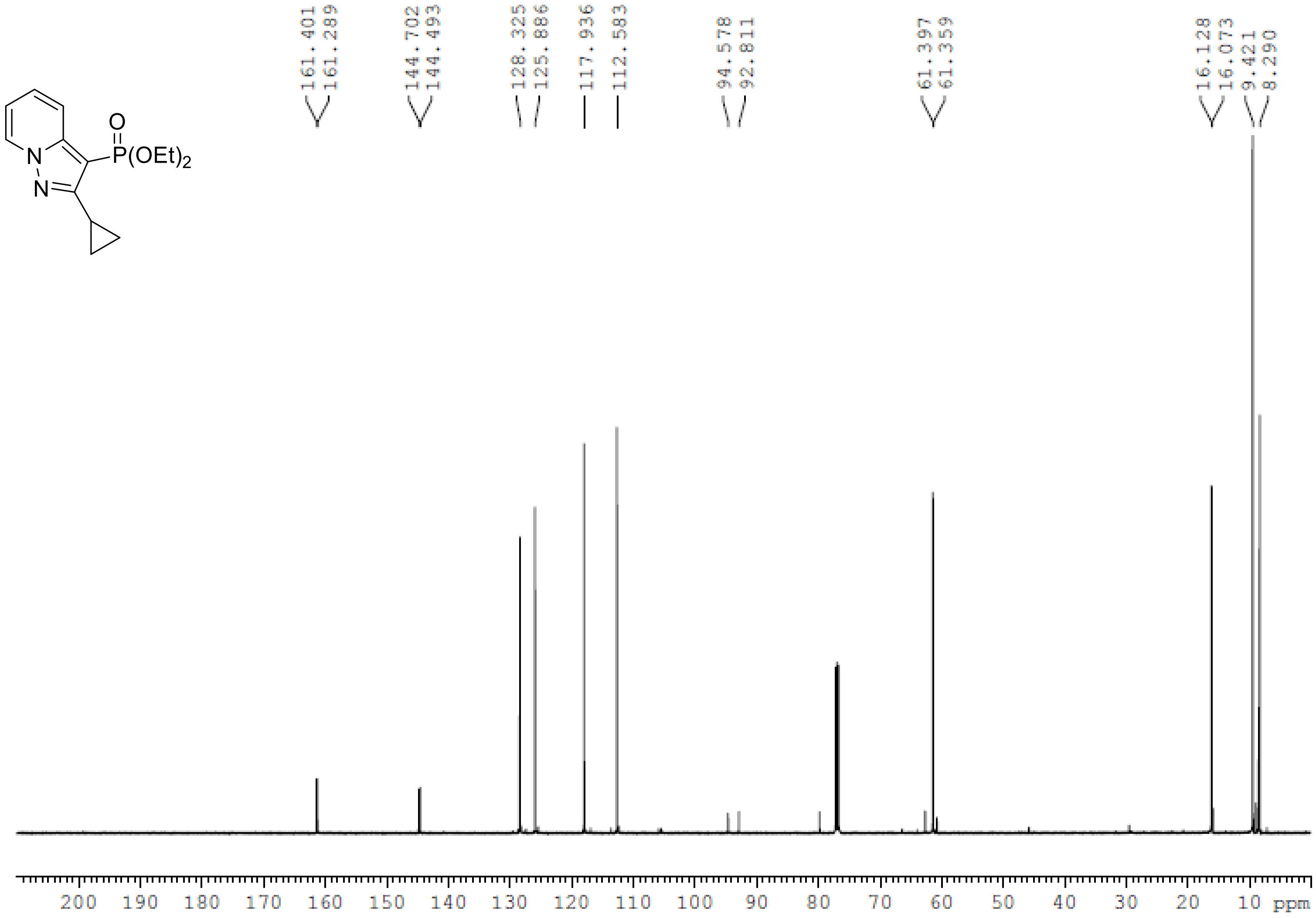

Diethyl (2-cyclopropylpyrazolo[1,5-a]pyridin-3-yl)phosphonate ^{31}P NMR(3i).

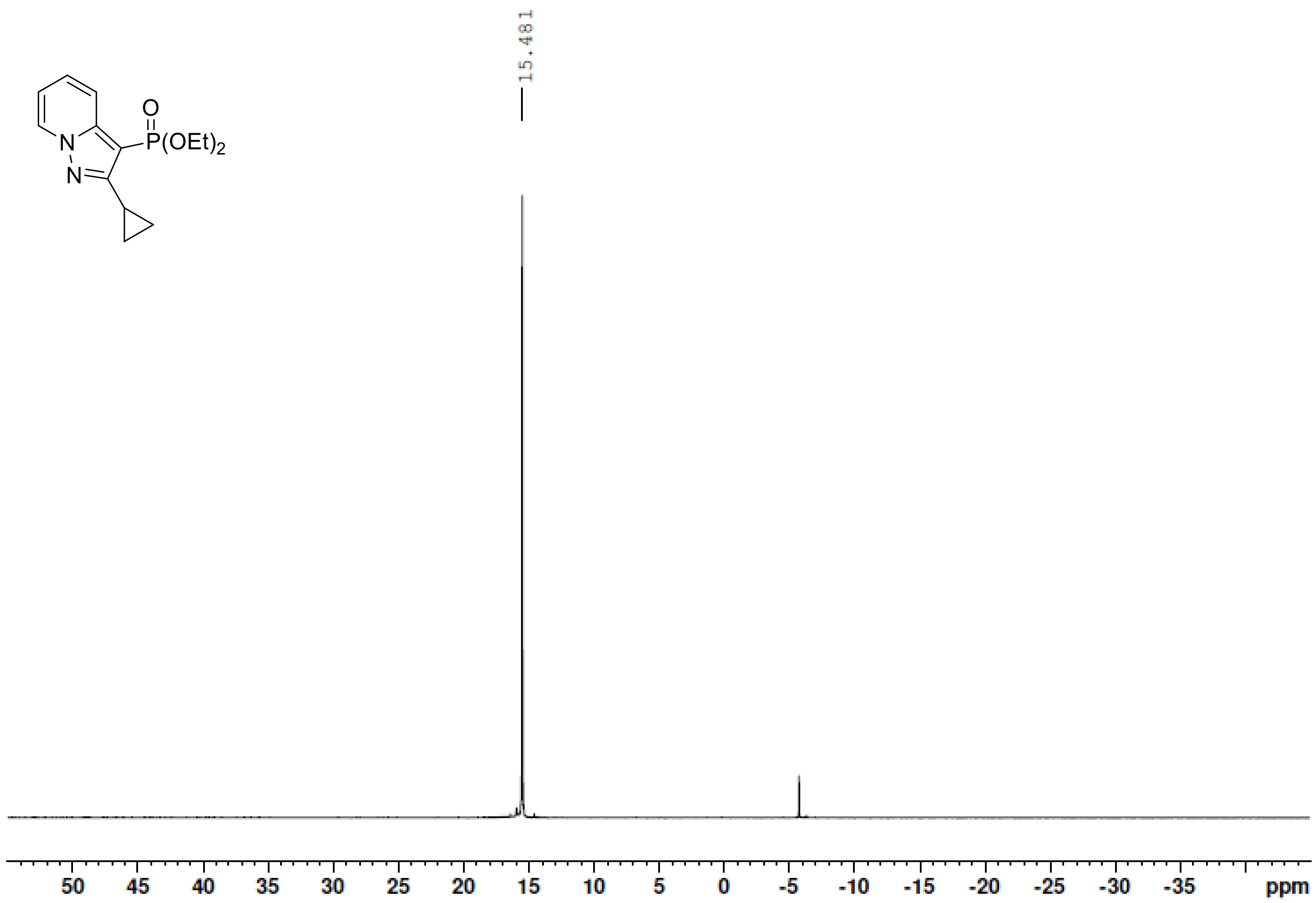

Diethyl (2-methyl[1,5-a]pyridin-3-yl)phosphonate ^{1}H NMR (3j).

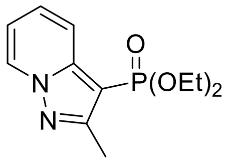

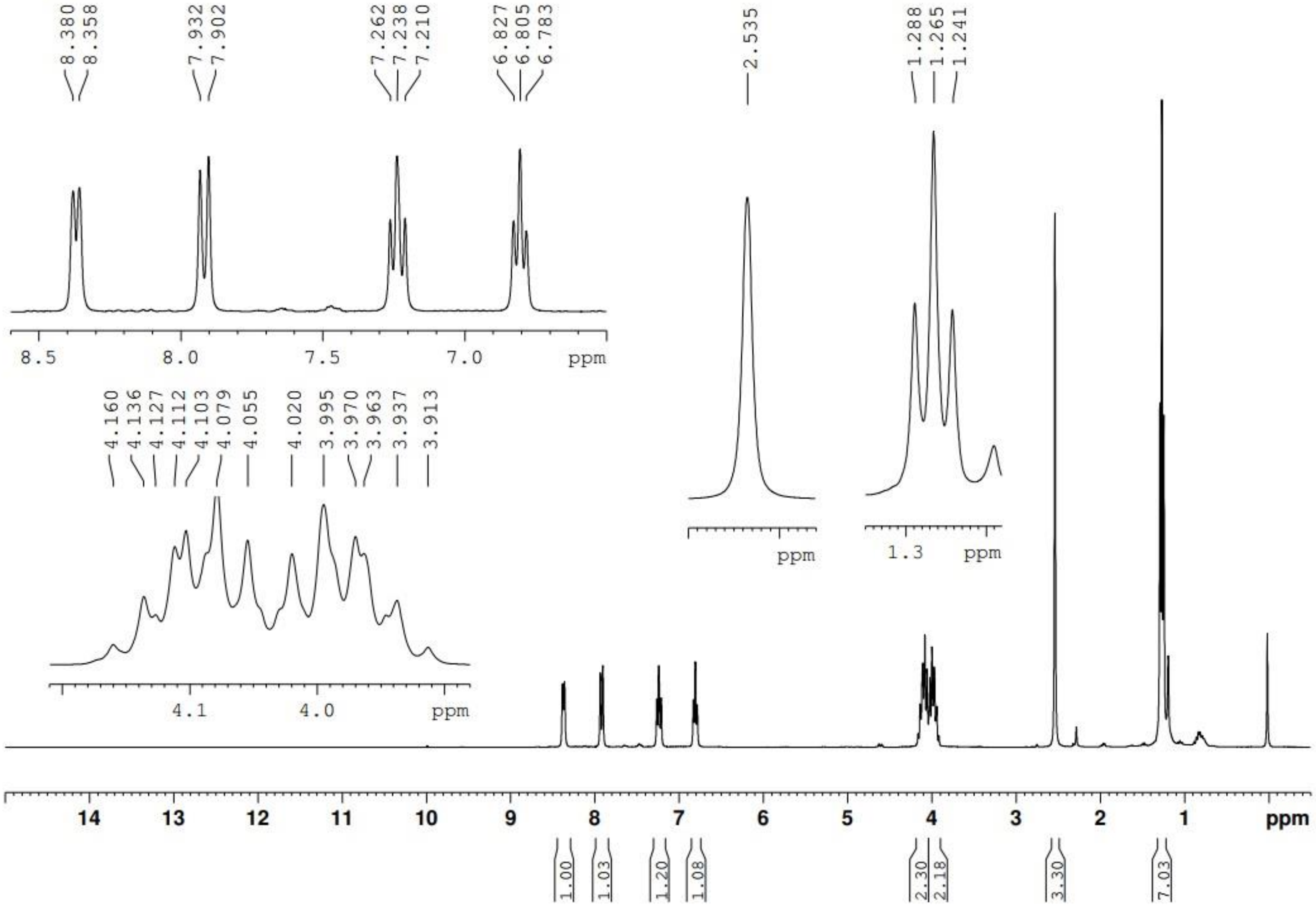

Diethyl (2-methyl[1,5-a]pyridin-3-yl)phosphonate ^{13}C NMR (3j).

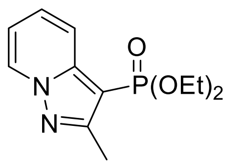

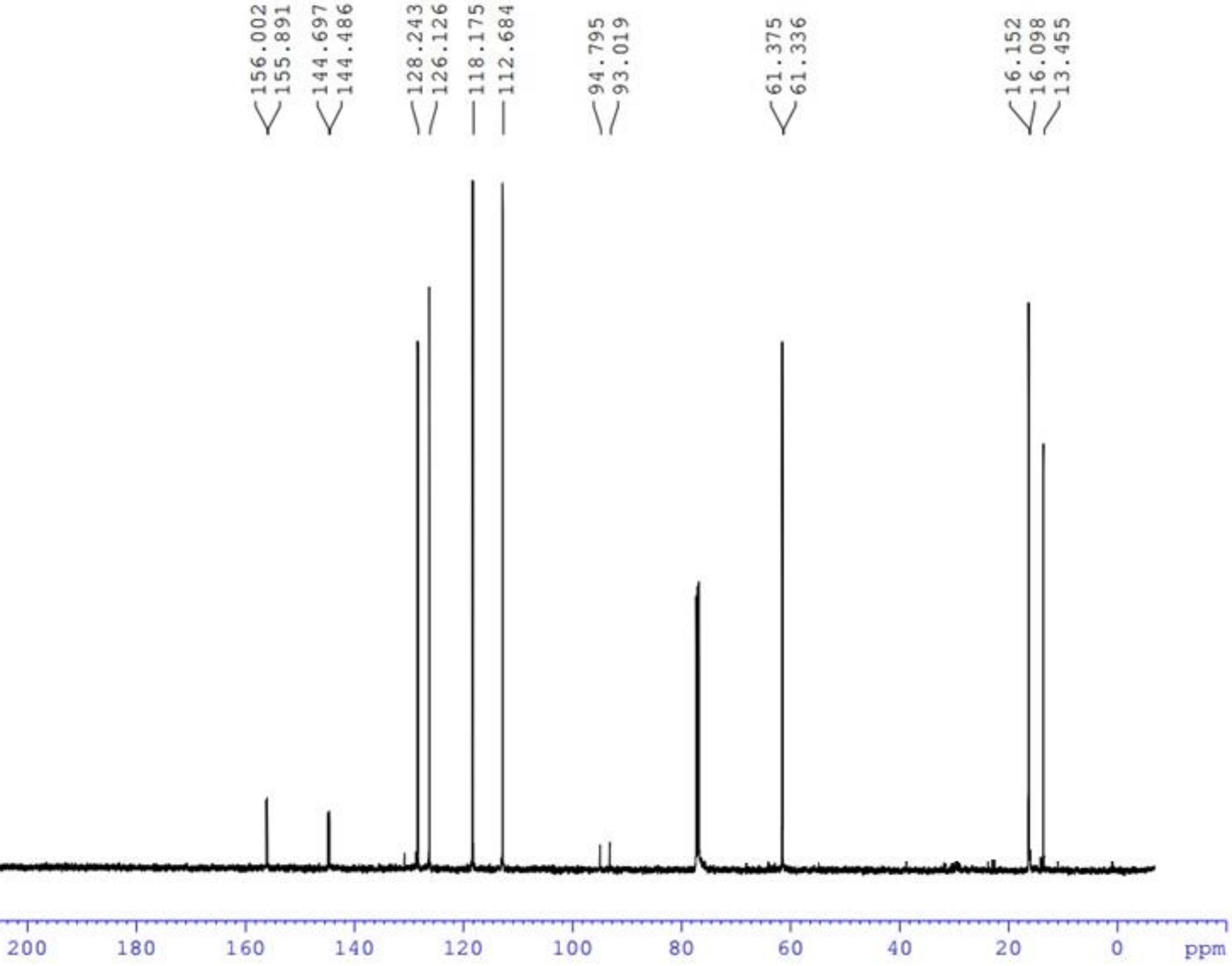

Diethyl (2-methyl[1,5-a]pyridin-3-yl)phosphonate ^{31}P NMR (3j).

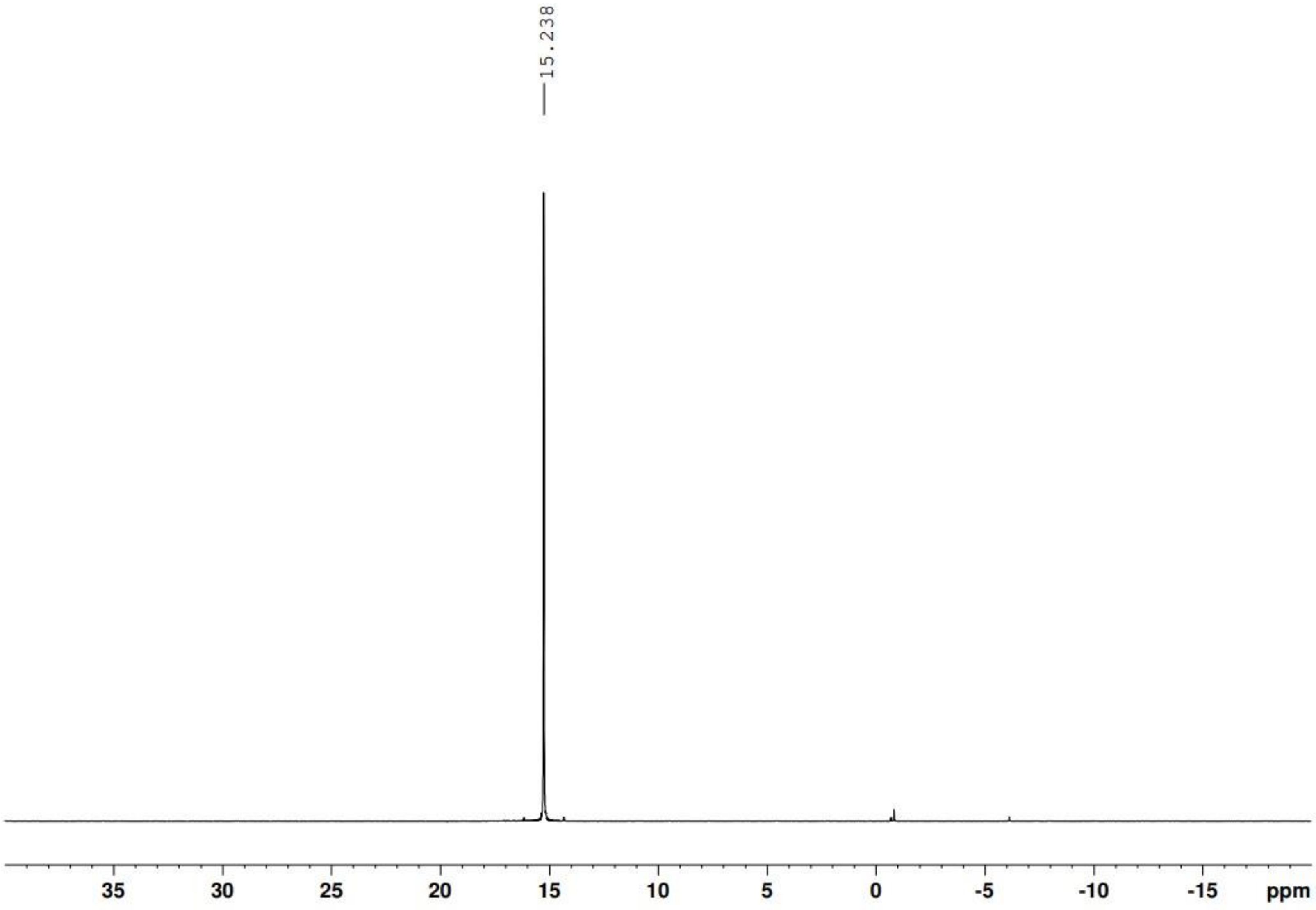

Diethyl (2-(2-hydroxypropan-2-yl)pyrazolo[1,5-a]pyridin-3-yl)phosphonate ^{1}H NMR (3k).

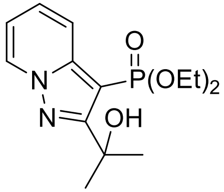

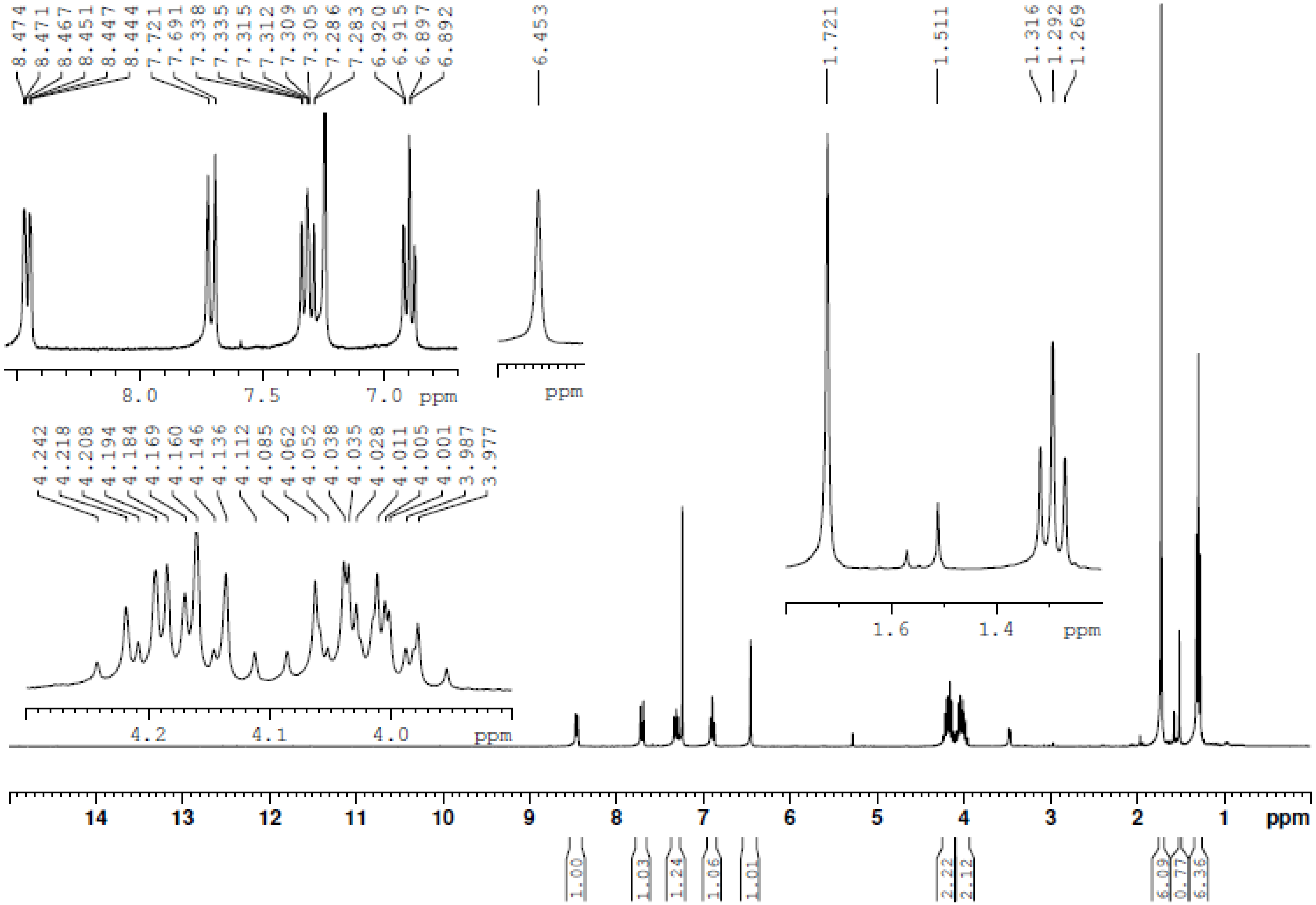

Diethyl (2-(2-hydroxypropan-2-yl)pyrazolo[1,5-a]pyridin-3-yl)phosphonate ^{13}C NMR (3k).

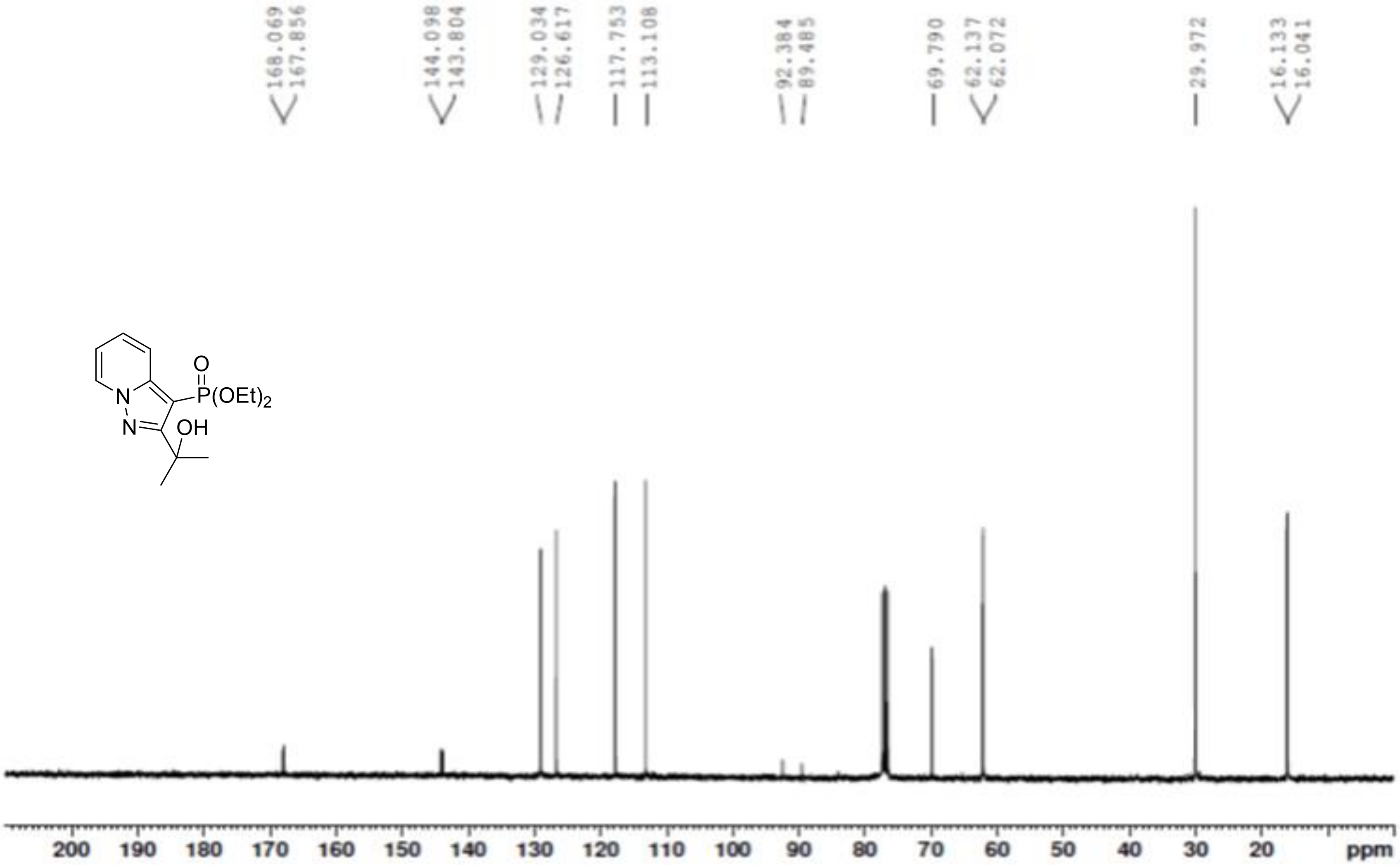

Diethyl (2-(2-hydroxypropan-2-yl)pyrazolo[1,5-a]pyridin-3-yl)phosphonate ^{31}P NMR (3k).

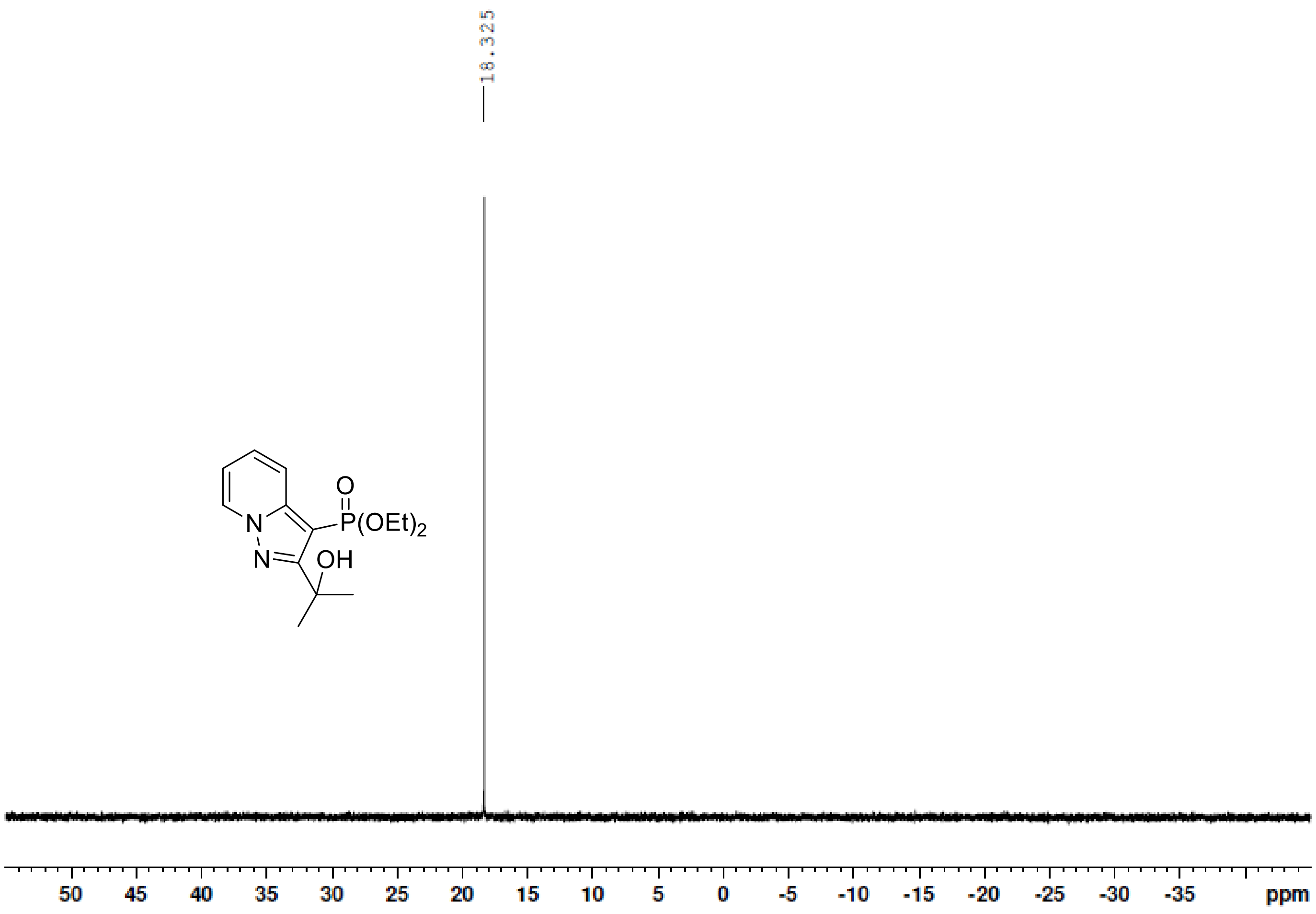

Diethyl (2-(1-hydroxycyclohexyl)pyrazolo[1,5-a]pyridin-3-yl)phosphonate ^{1}H NMR (3l).

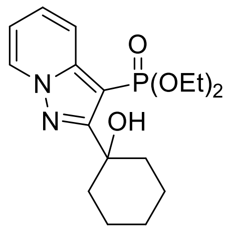

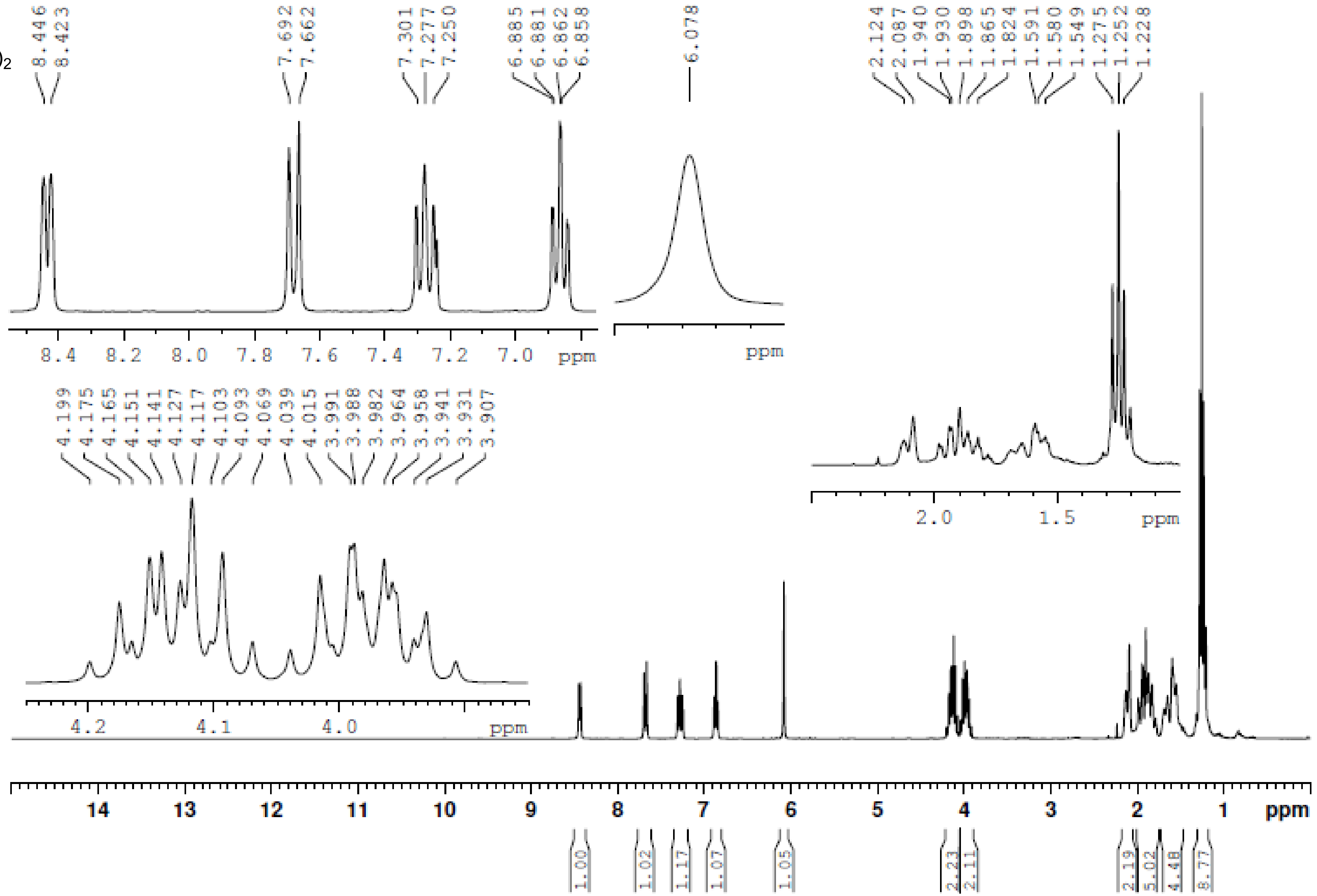

Diethyl (2-(1-hydroxycyclohexyl)pyrazolo[1,5-a]pyridin-3-yl)phosphonate ^{13}C NMR (3l).

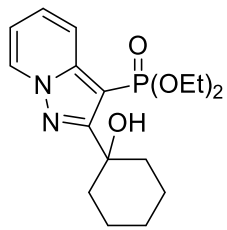

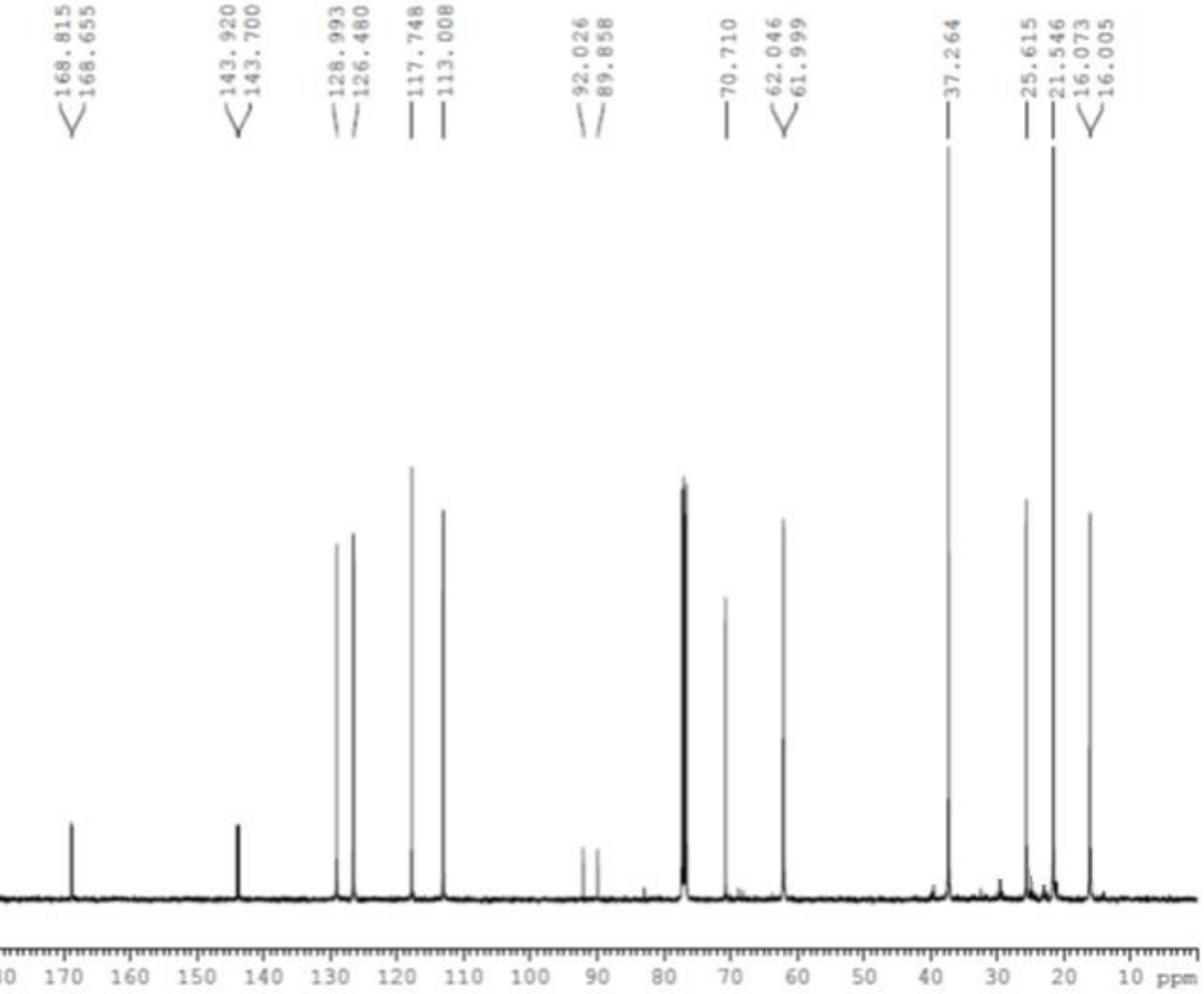

Diethyl (2-(1-hydroxycyclohexyl)pyrazolo[1,5-a]pyridin-3-yl)phosphonate ^{31}P NMR (3l).

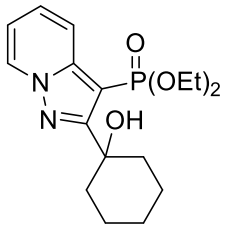

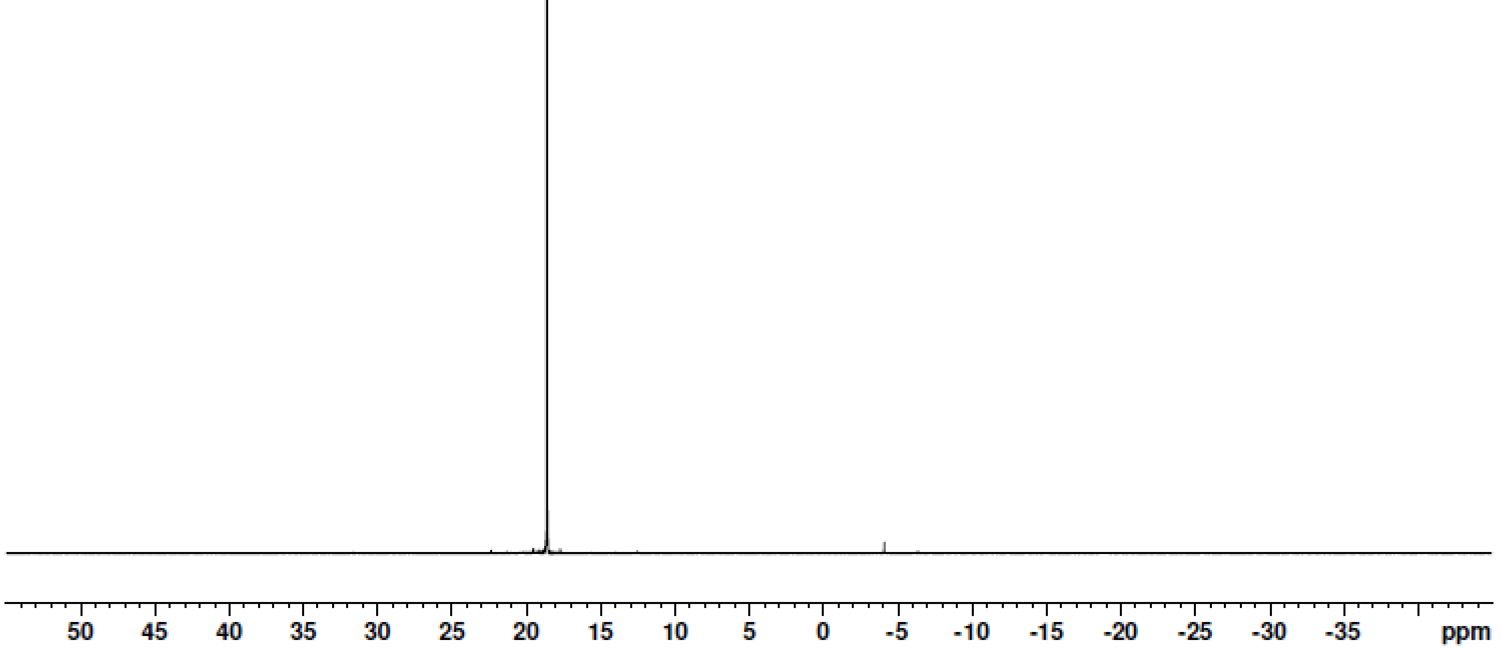

Tetraethyl pyrazolo[1,5-a]pyridine-2,3-diylbis(phosphonate) (5a) ^{1}H NMR.

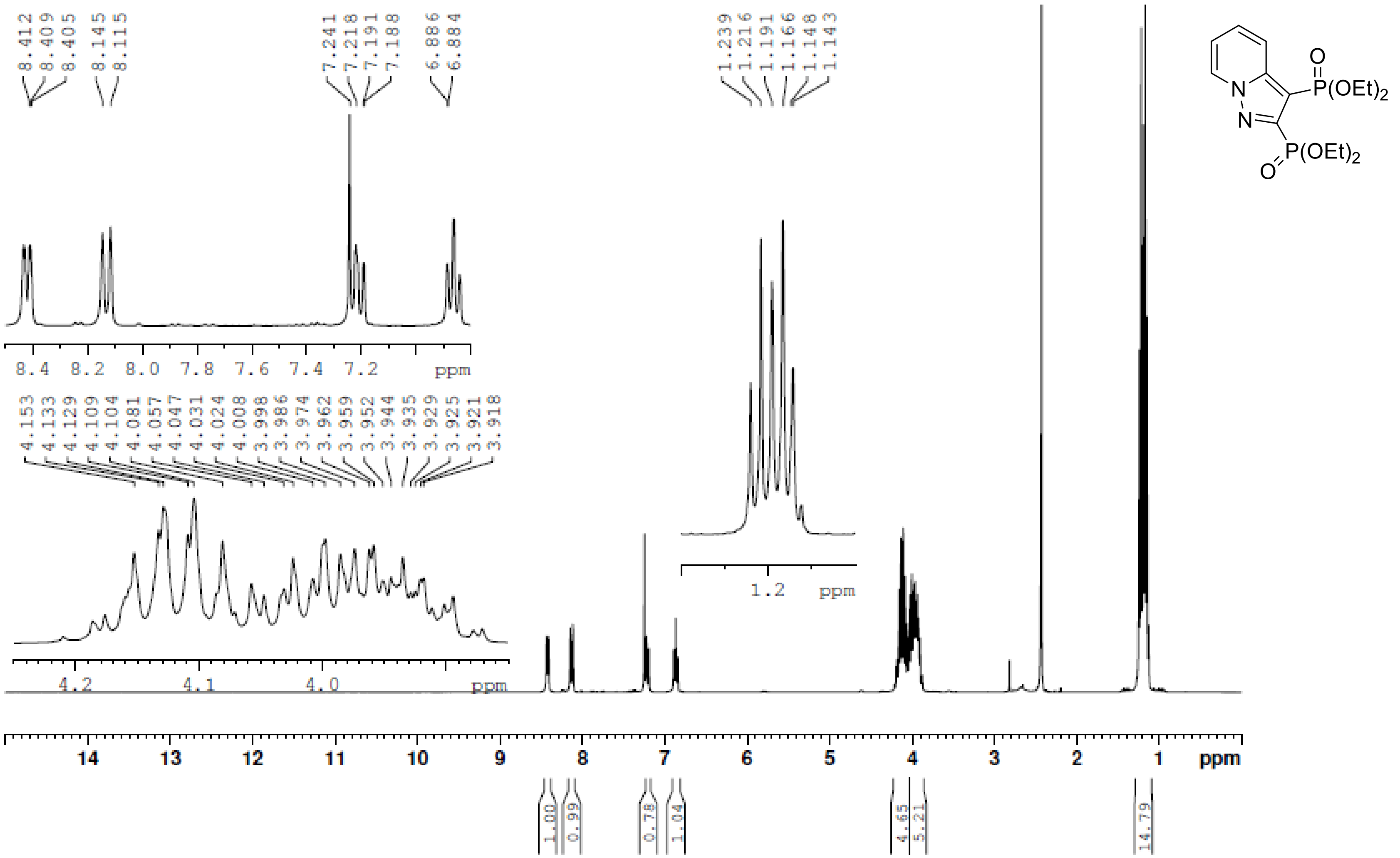

Tetraethyl pyrazolo[1,5-a]pyridine-2,3-diylbis(phosphonate) (5a) ^{13}C NMR

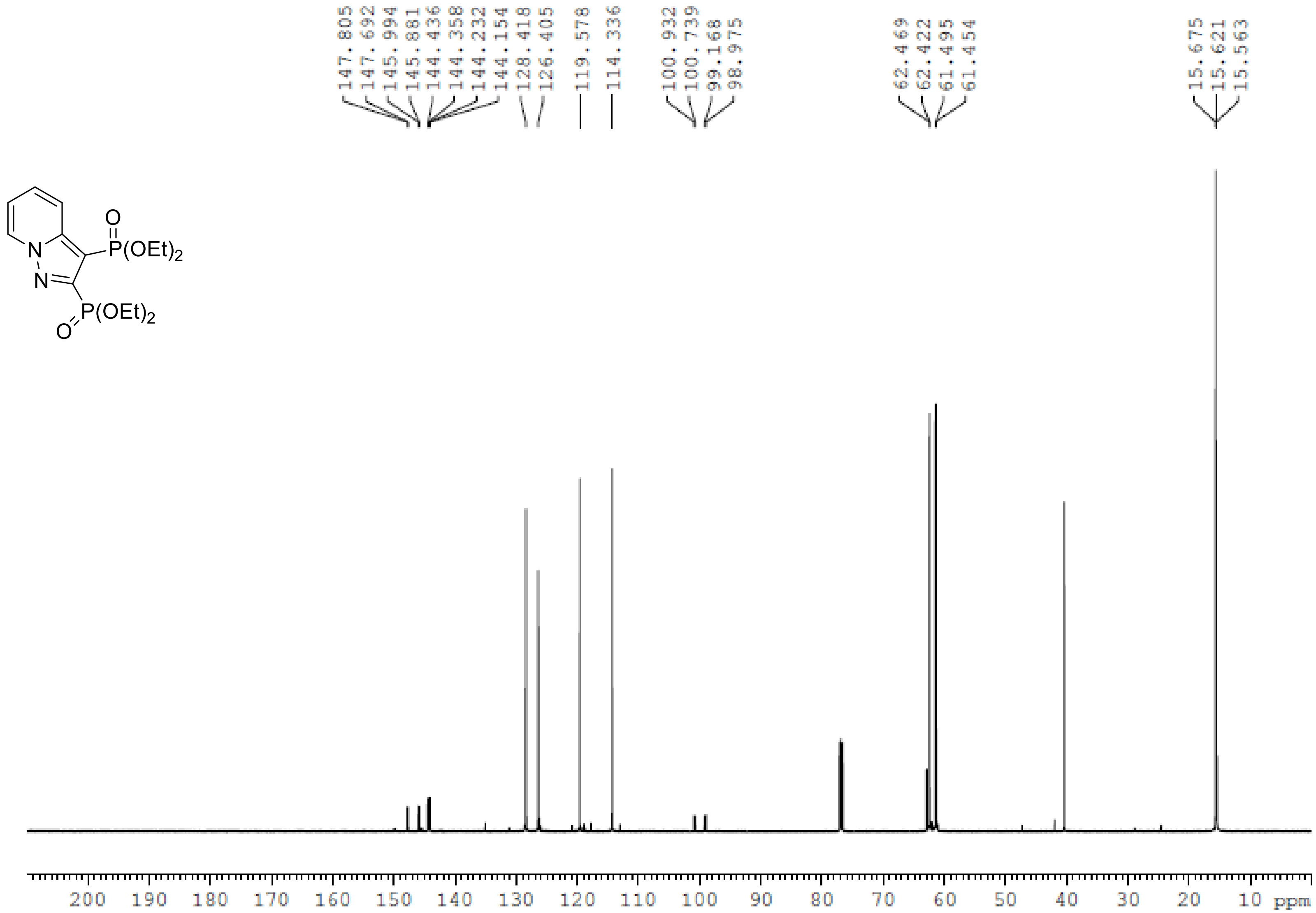

Tetraethyl pyrazolo[1,5-a]pyridine-2,3-diylbis(phosphonate) (5a) ^{31}P NMR.

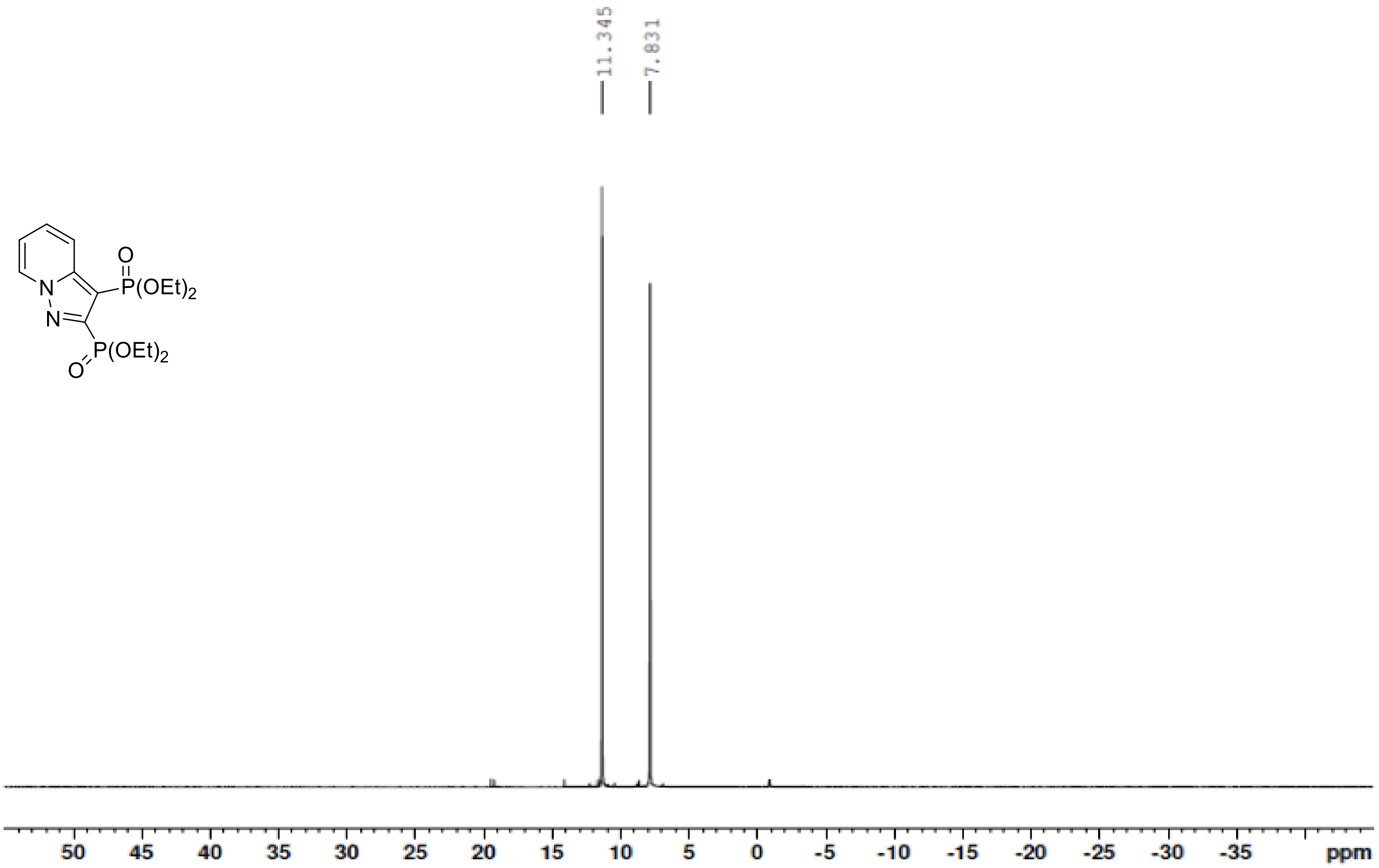

Tetraethyl (5-methylpyrazolo[1,5-a]pyridine-2,3-diyl)bis(phosphonate) (5b) ^{1}H NMR

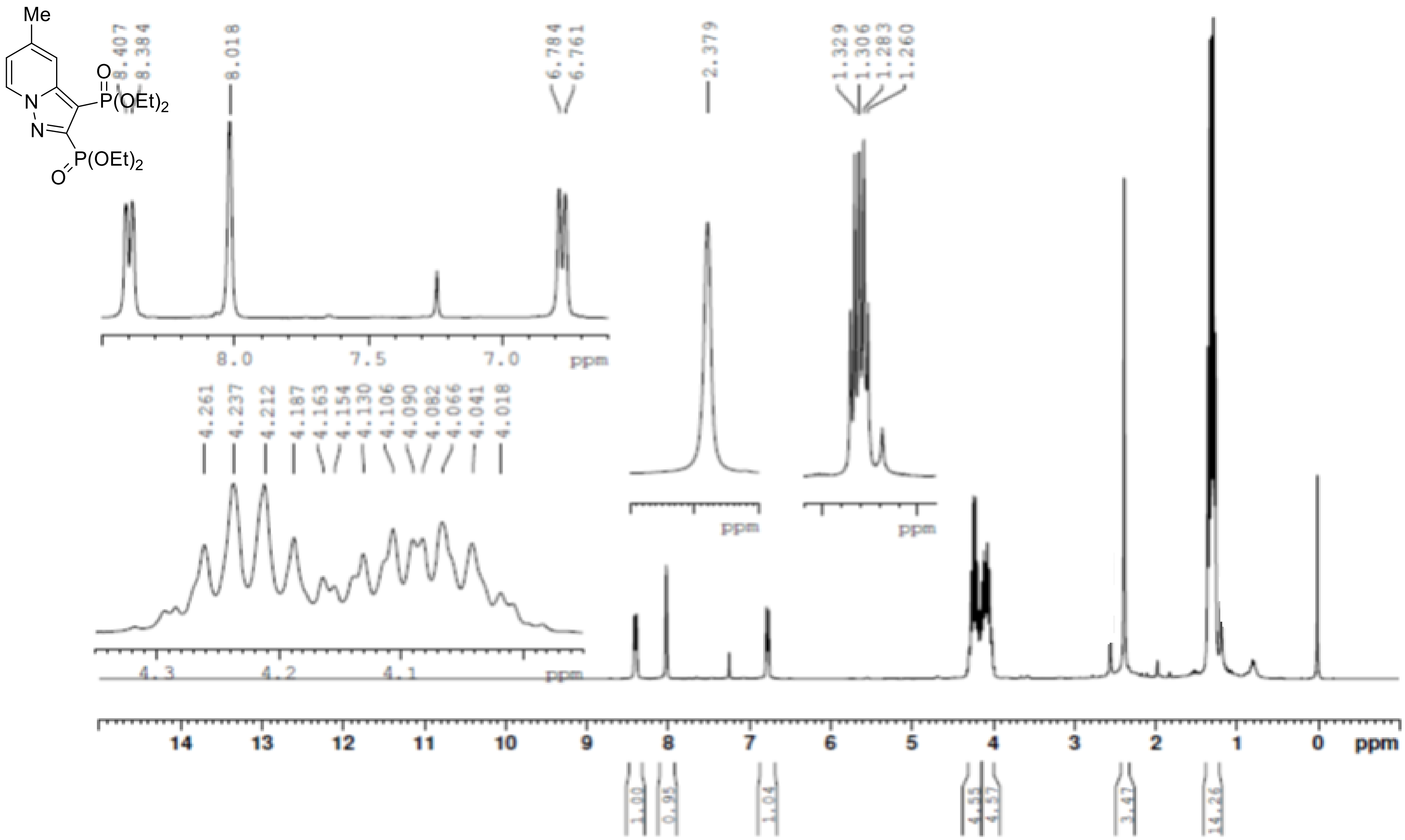

Tetraethyl (5-methylpyrazolo[1,5-a]pyridine-2,3-diyl)bis(phosphonate) (5b) ^{13}C NMR

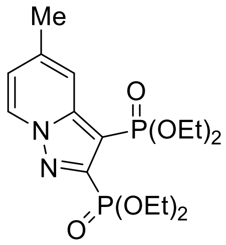

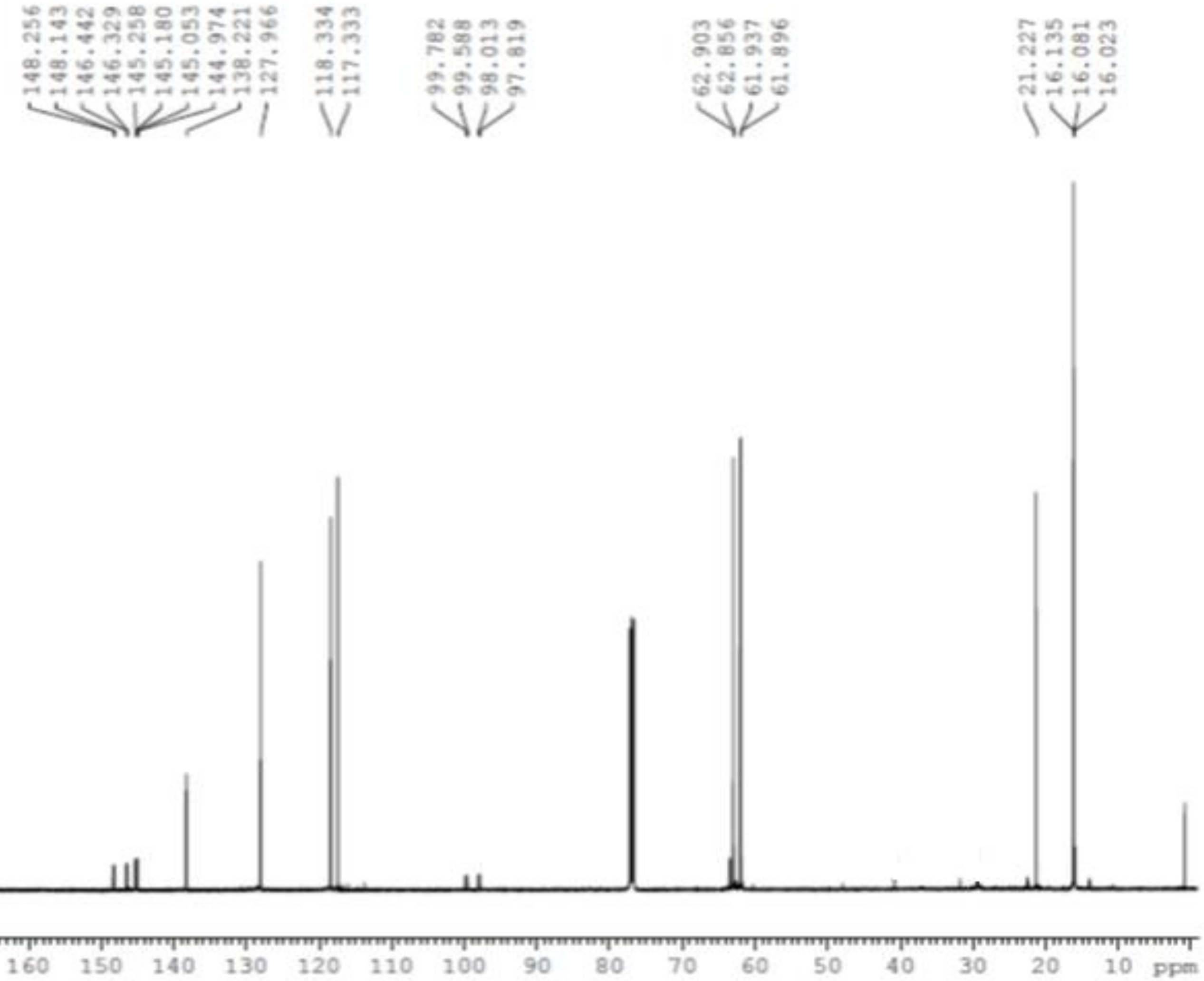

Tetraethyl (5-methylpyrazolo[1,5-a]pyridine-2,3-diyl)bis(phosphonate) (5b) ^{31}P NMR

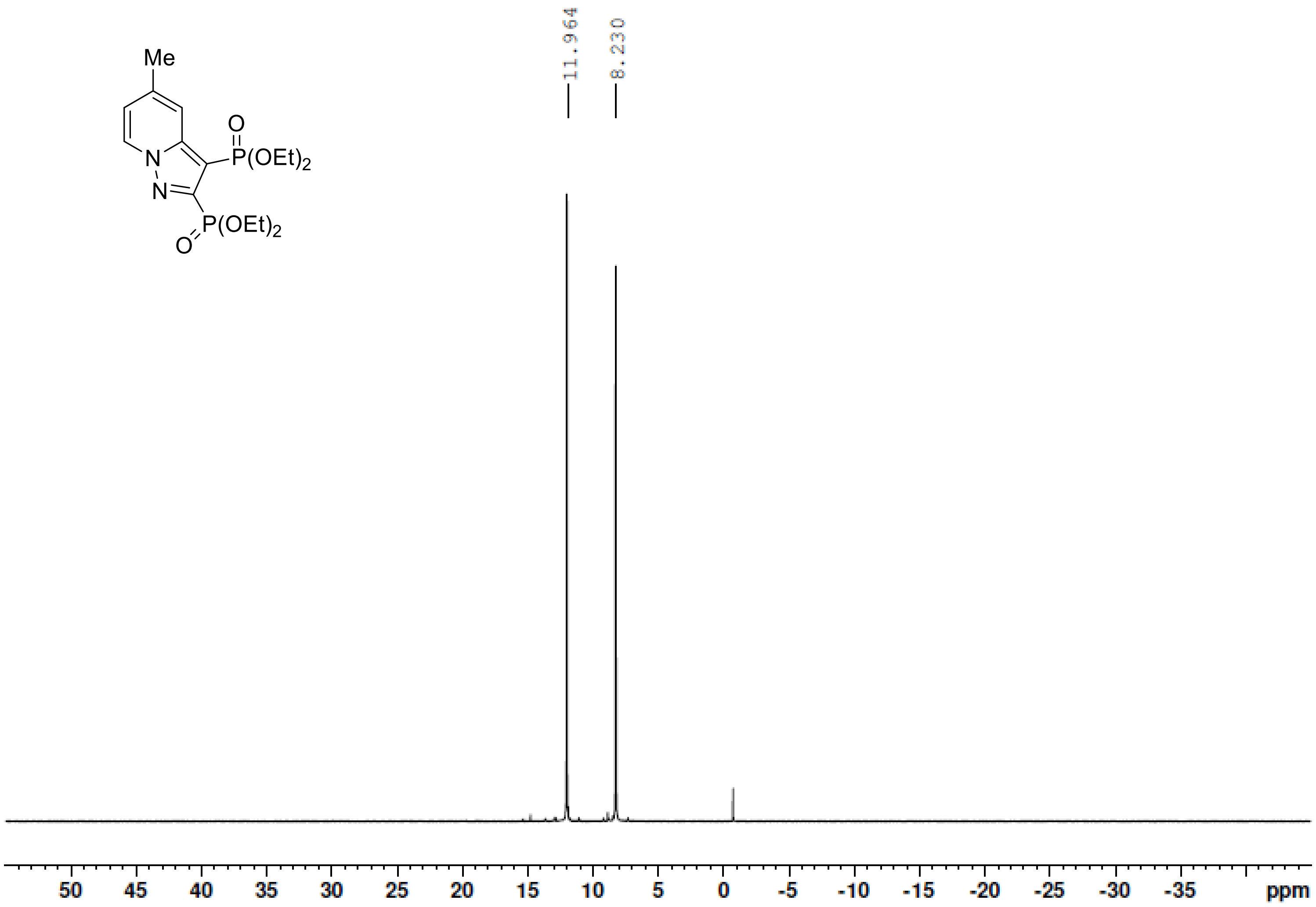

Tetraethyl (6-methylpyrazolo[1,5-*a*]pyridine-2,3-diyl)bis(phosphonate) and tetraethyl (4-methylpyrazolo[1,5-*a*]pyridine-2,3-diyl)bis(phosphonate) mixture (5c) ^{1}H NMR.

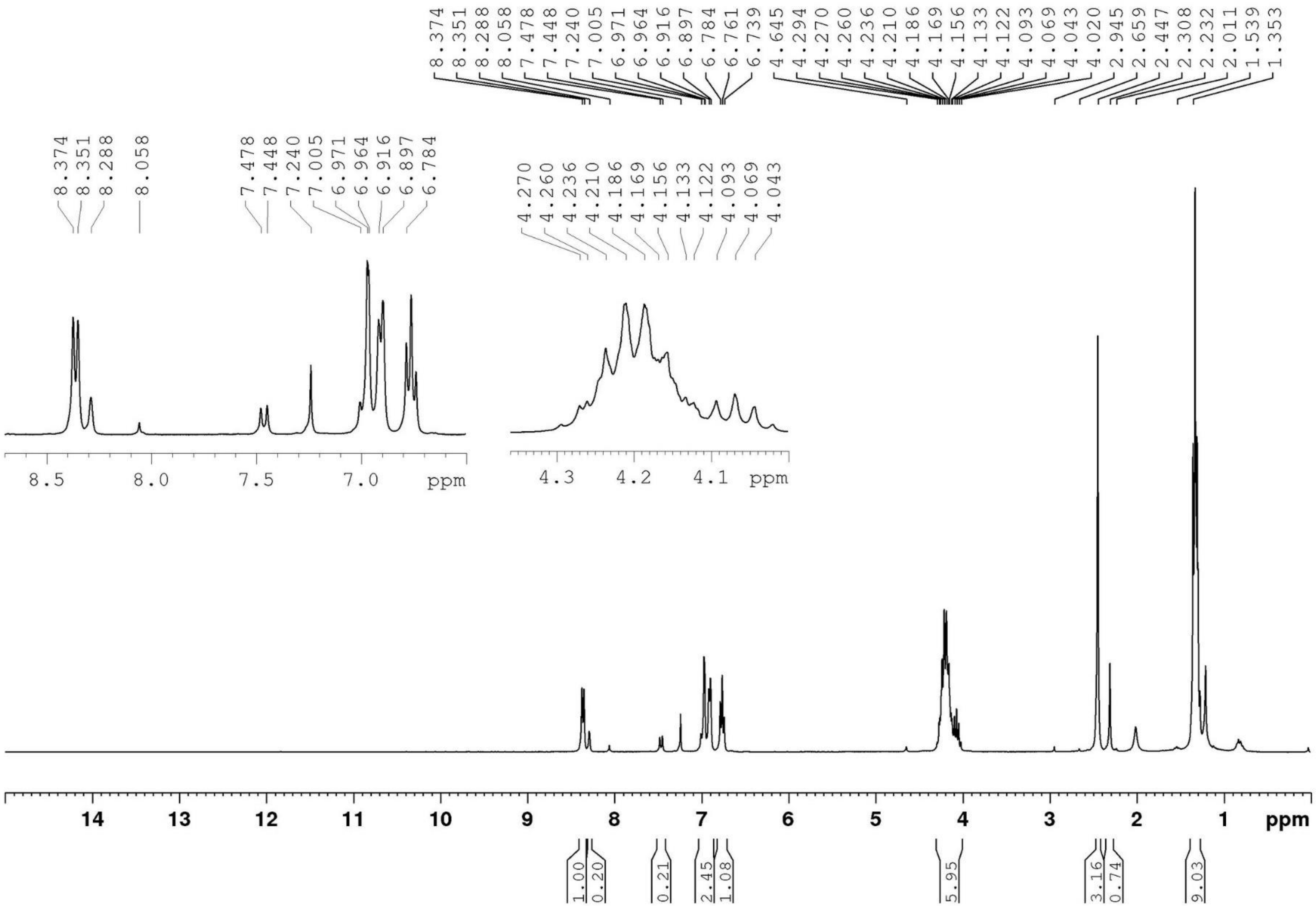

Tetraethyl (6-methylpyrazolo[1,5-*a*]pyridine-2,3-diyl)bis(phosphonate) and tetraethyl (4-methylpyrazolo[1,5-*a*]pyridine-2,3-diyl)bis(phosphonate) mixture (5c) ^{31}P NMR.

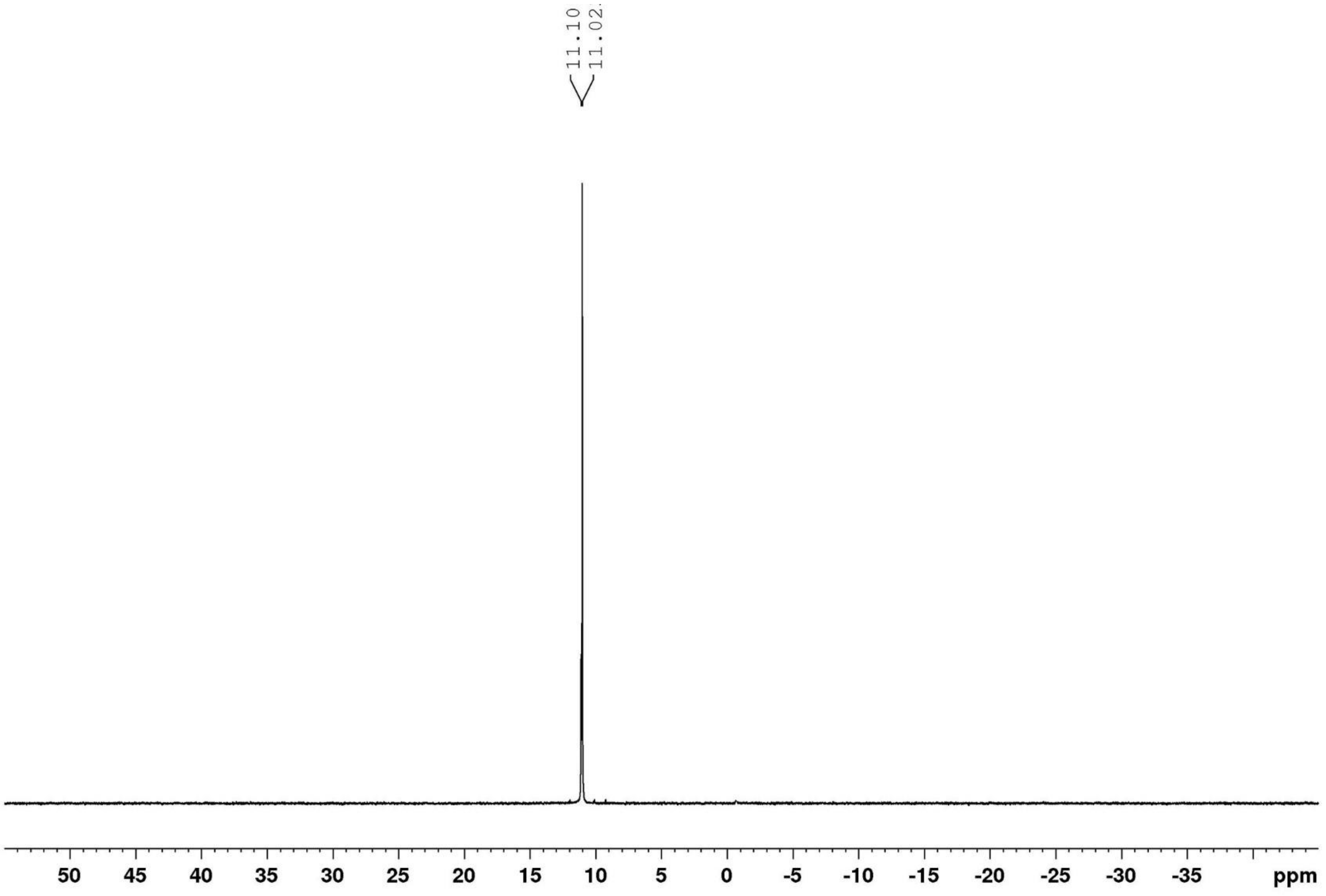

Ethyl 2,3-bis(diethoxyphosphoryl)pyrazolo[1,5-a]pyridine-5-carboxylate (5d) ^{1}H NMR

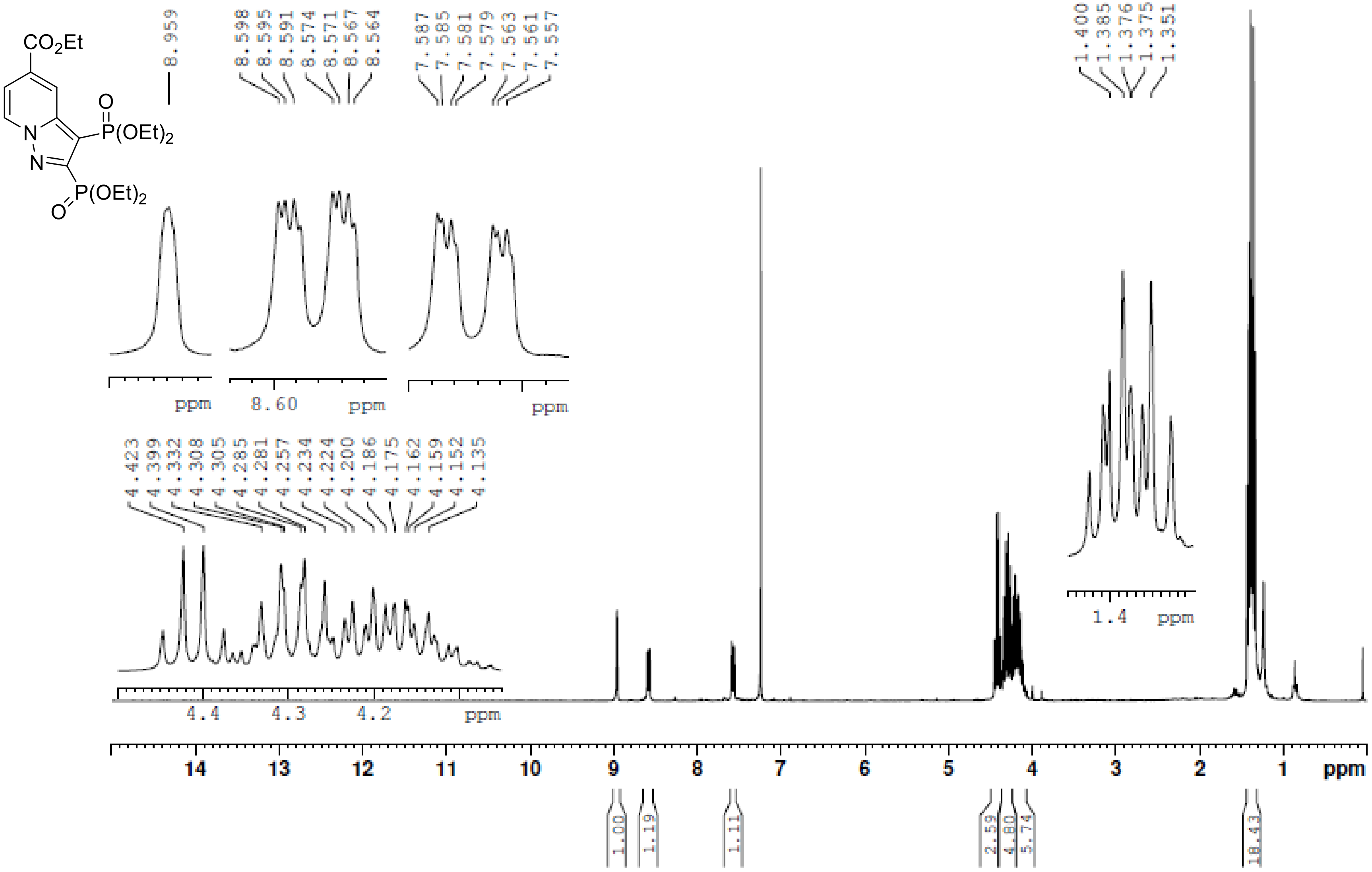

Ethyl 2,3-bis(diethoxyphosphoryl)pyrazolo[1,5-a]pyridine-5-carboxylate (5d) ^{13}C NMR

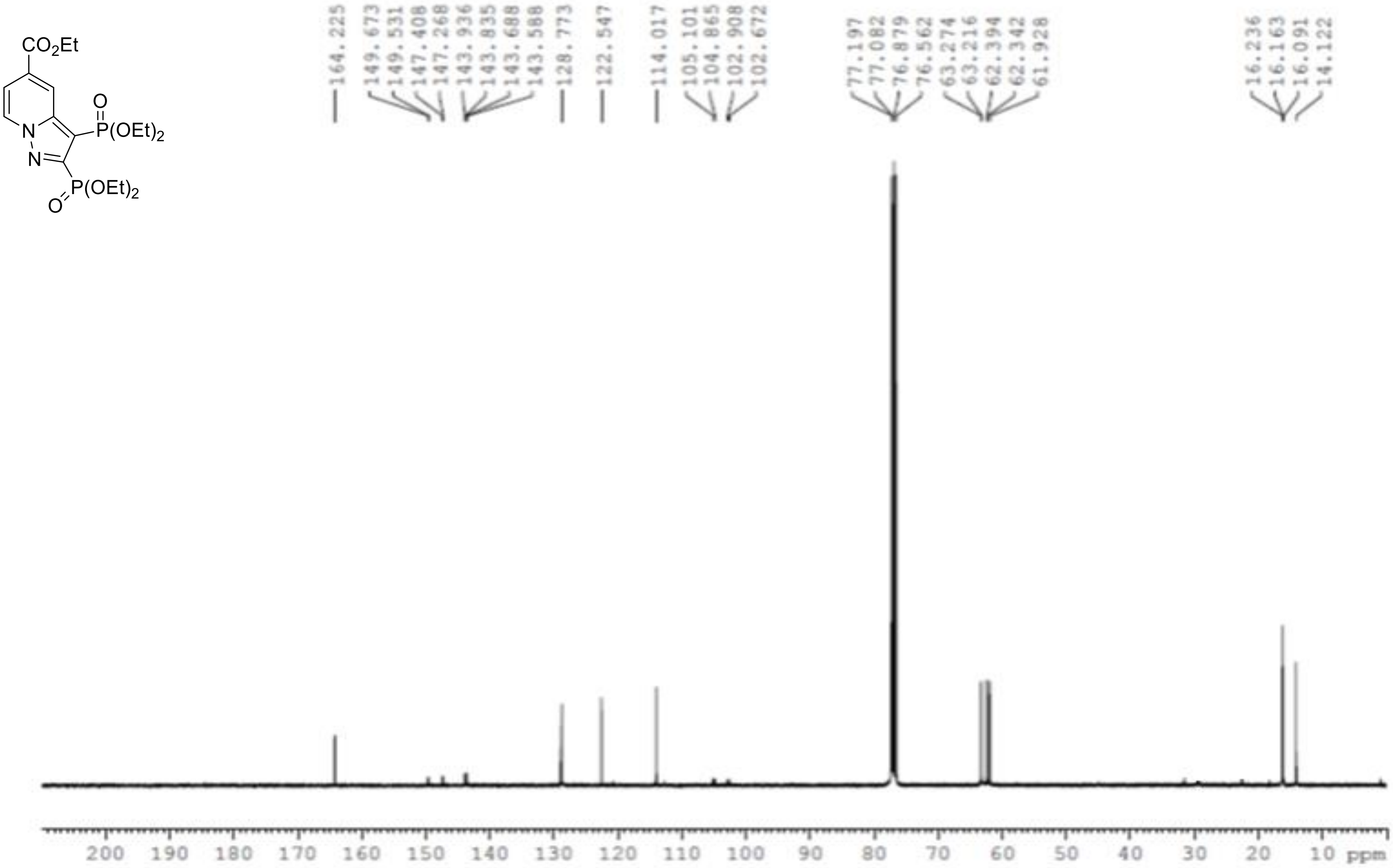

Ethyl 2,3-bis(diethoxyphosphoryl)pyrazolo[1,5-a]pyridine-5-carboxylate (5d) ^{31}P NMR

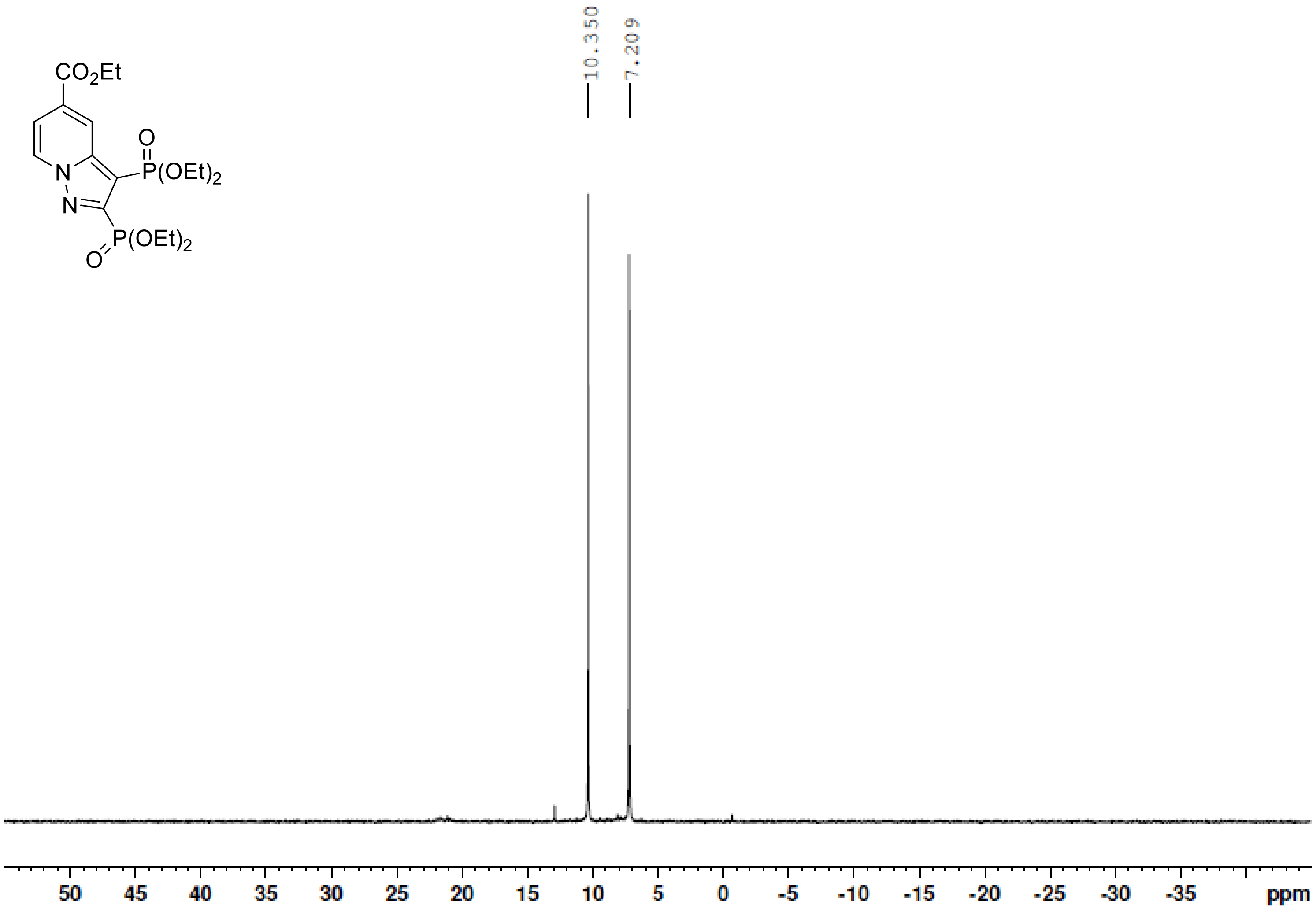

Tetraethyl (5-methoxypyrazolo[1,5-a]pyridine-2,3-diyl)bis(phosphonate) (5e) ^{1}H NMR.

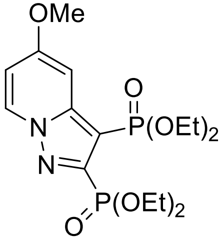

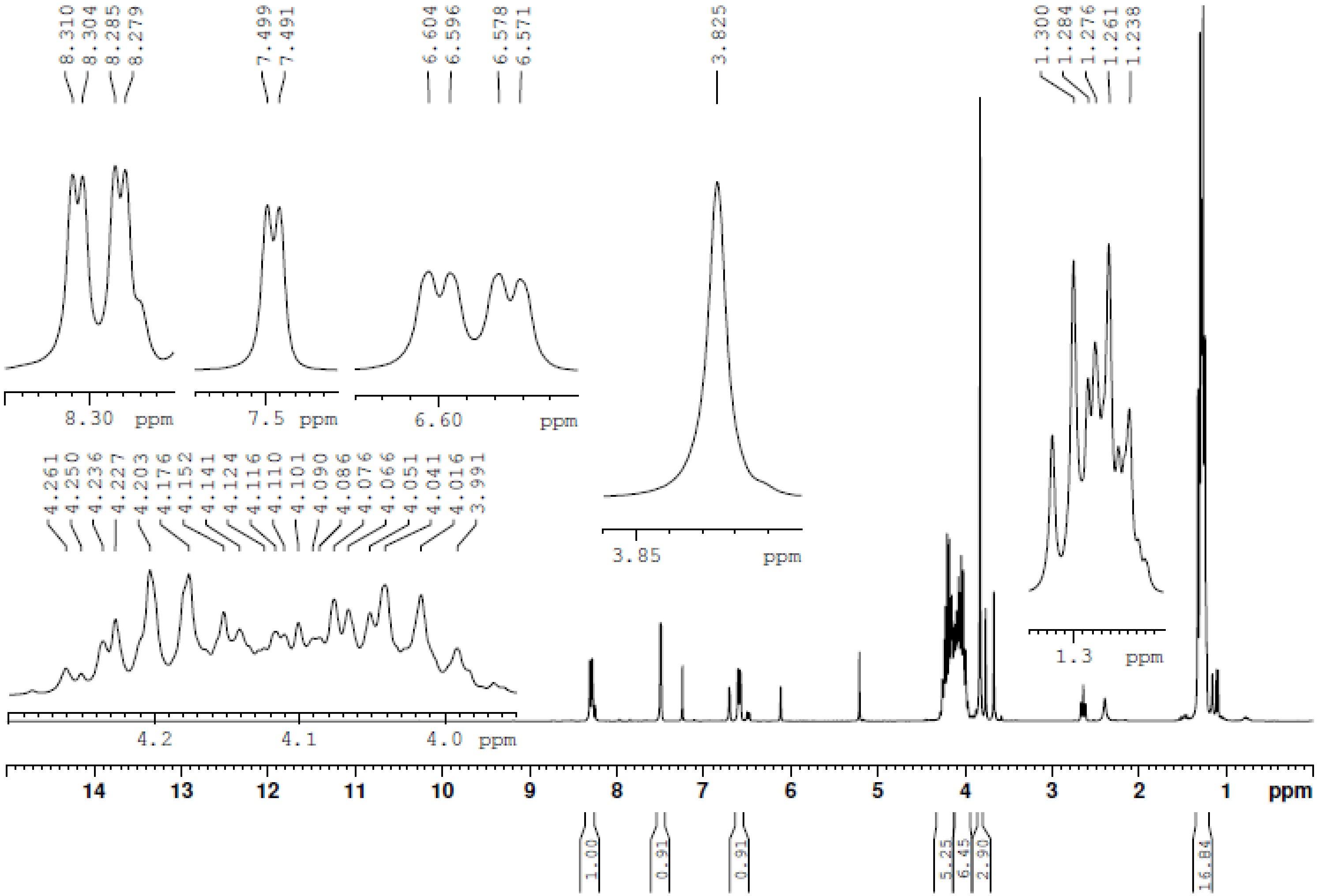

Tetraethyl (5-methoxypyrazolo[1,5-a]pyridine-2,3-diyl)bis(phosphonate) ^{31}P NMR

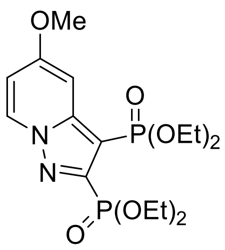

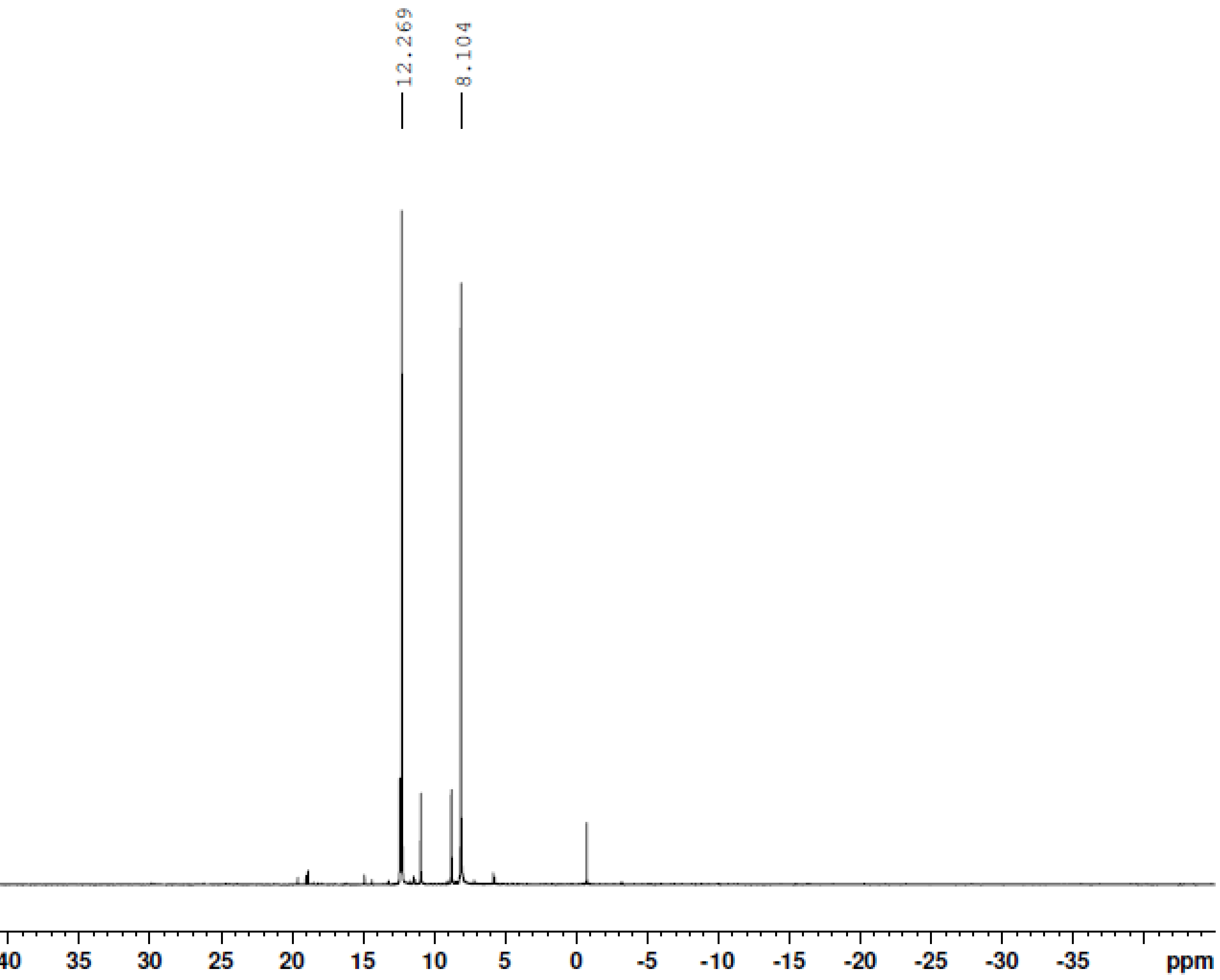

Tetraethyl 5-(pyridin-4-yl)pyrazolo[1,5-a]pyridine-2,3-diyldiphosphonate (5f) ^{1}H NMR.

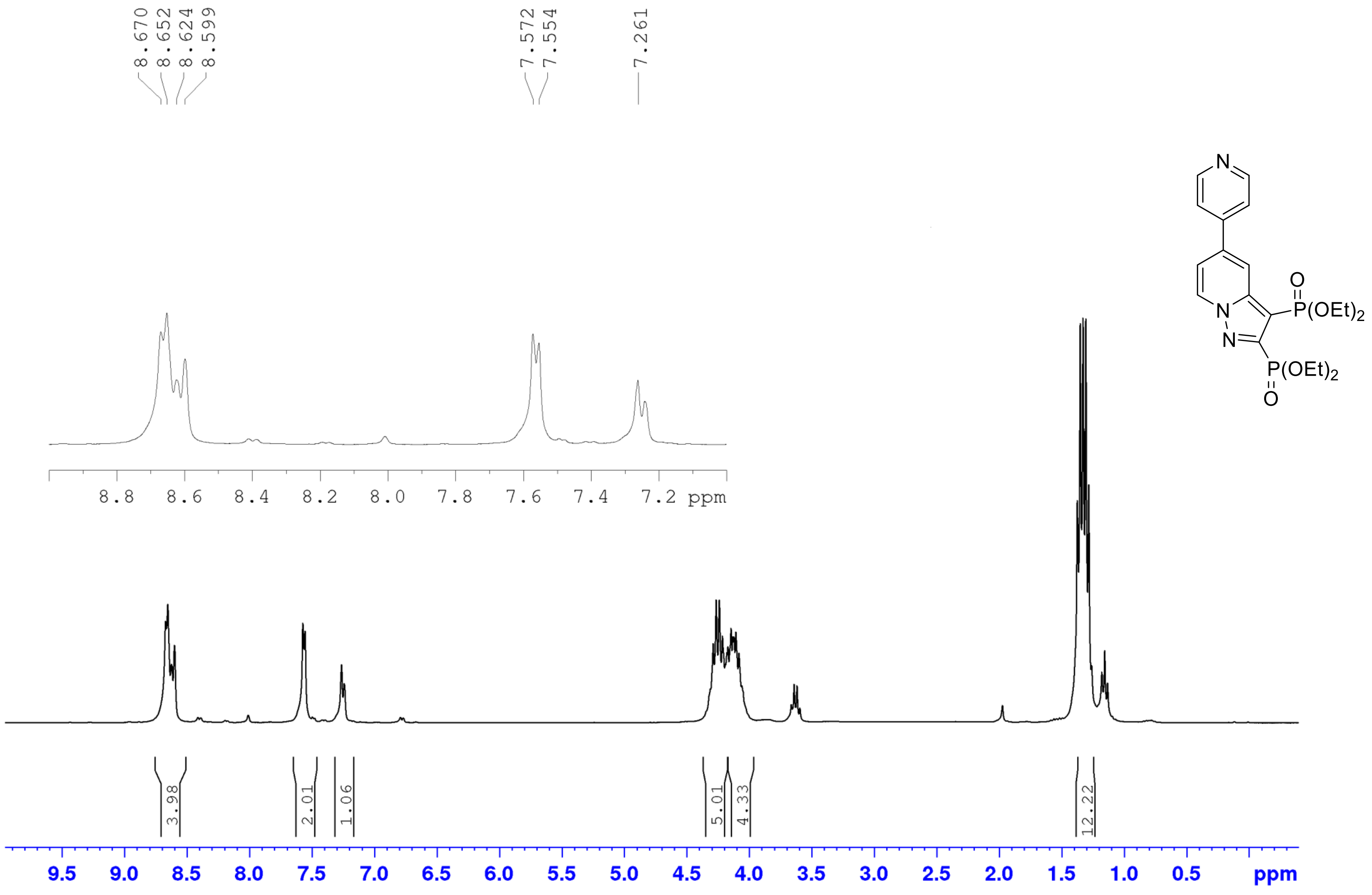

Tetraethyl 5-(pyridin-4-yl)pyrazolo[1,5-a]pyridine-2,3-diyldiphosphonate (5f) ^{13}C NMR.

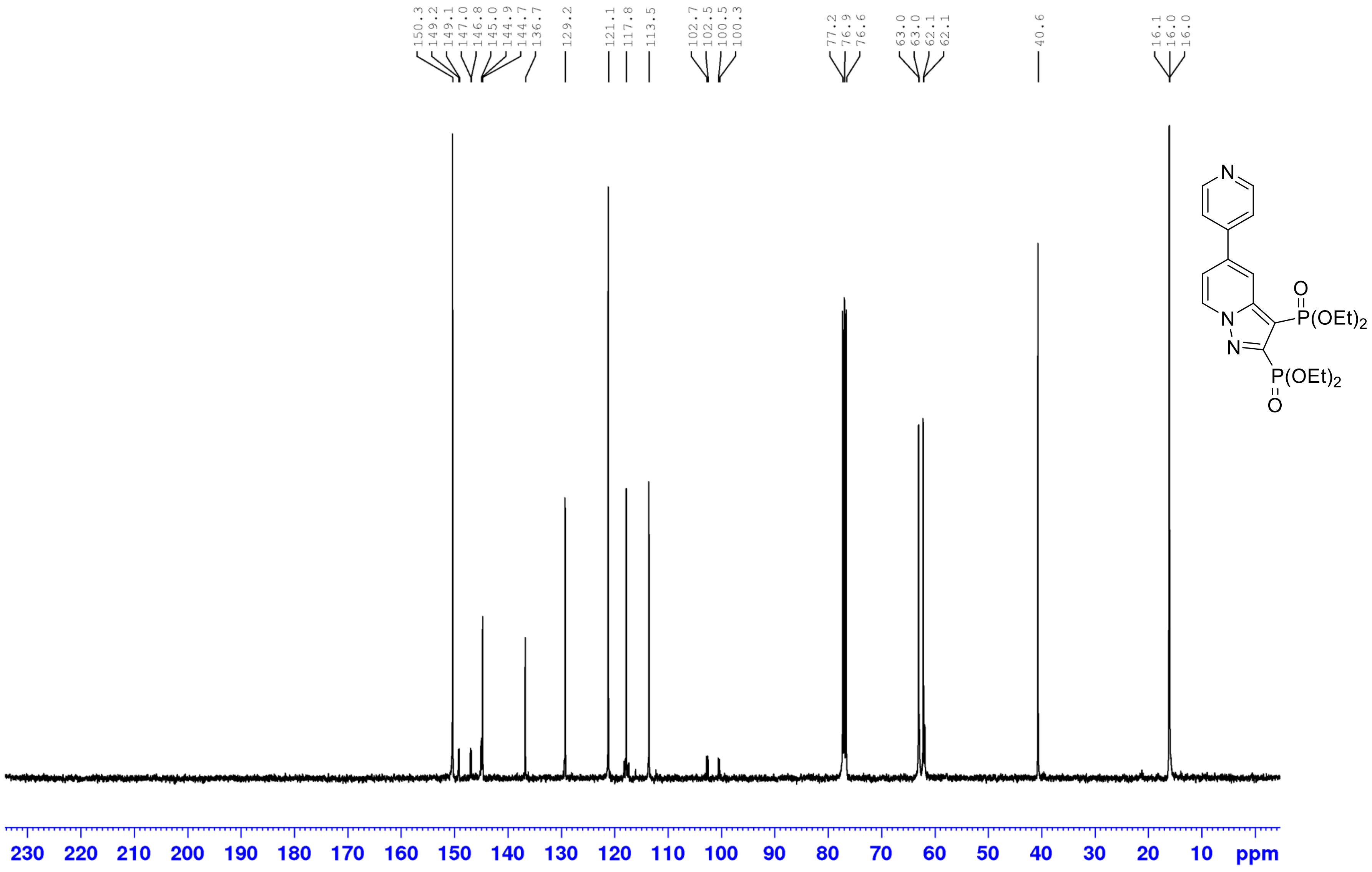

Tetraethyl 5-(pyridin-4-yl)pyrazolo[1,5-a]pyridine-2,3-diyldiphosphonate (5f) ^{31}P NMR.

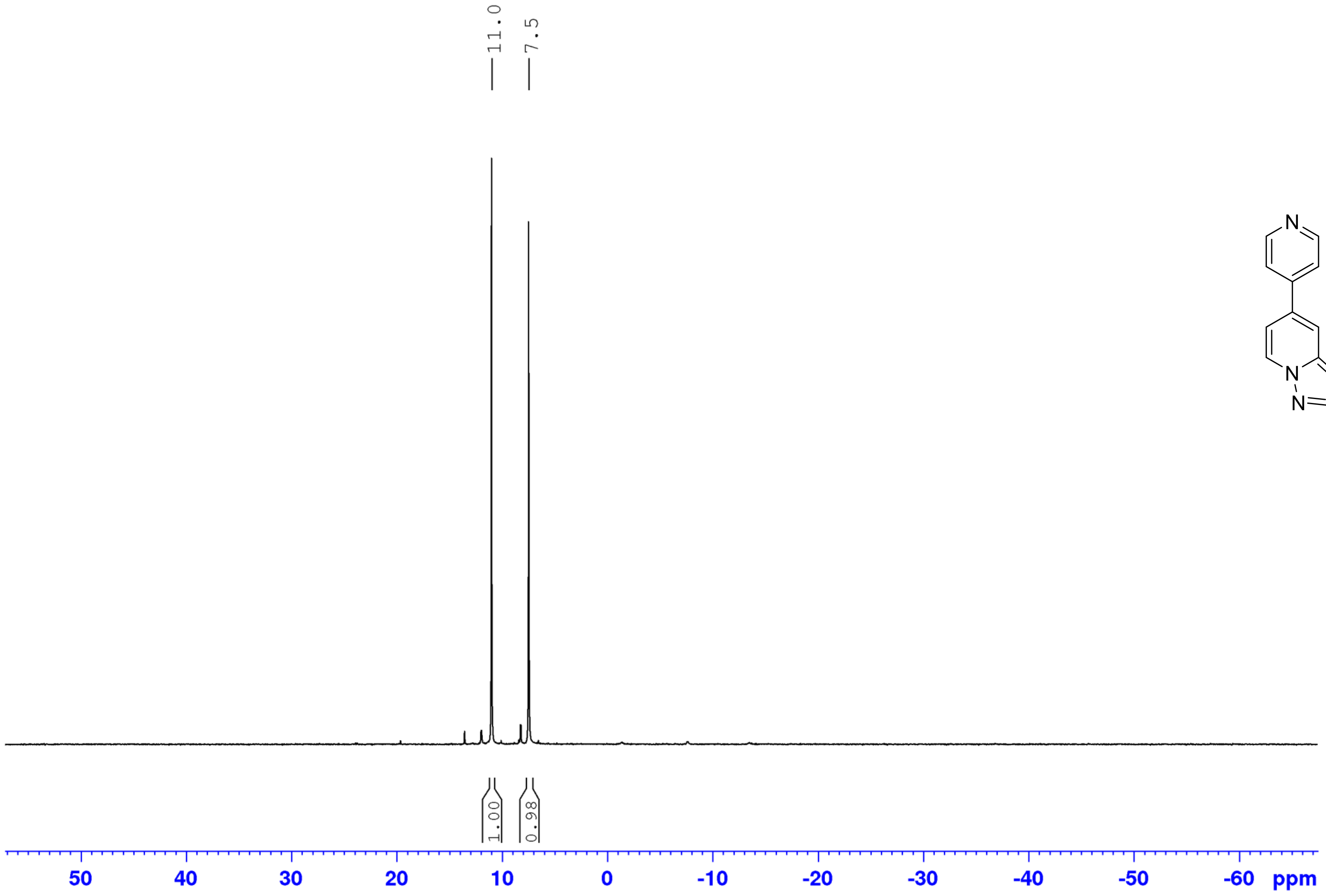

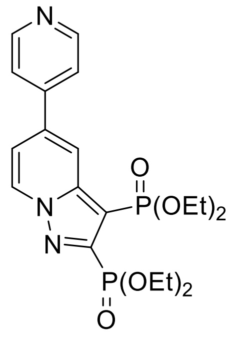

Tetraethyl pyrazolo[1,5-a]quinoline-2,3-diyldiphosphonate (5g) ^{1}H NMR

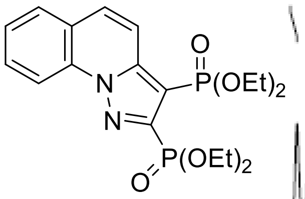

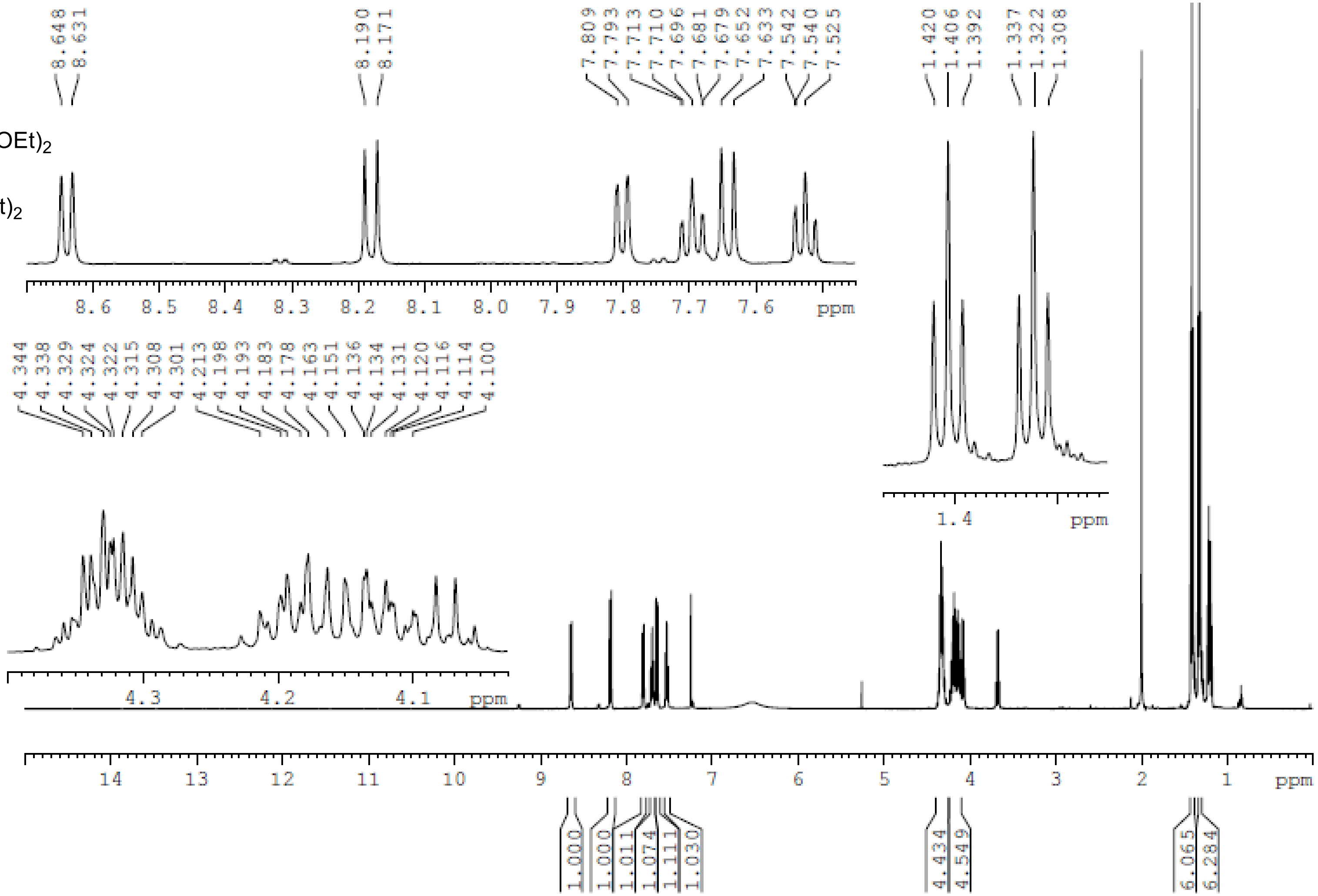

Tetraethyl pyrazolo[1,5-a]quinoline-2,3-diyldiphosphonate (5g) ^{13}C NMR

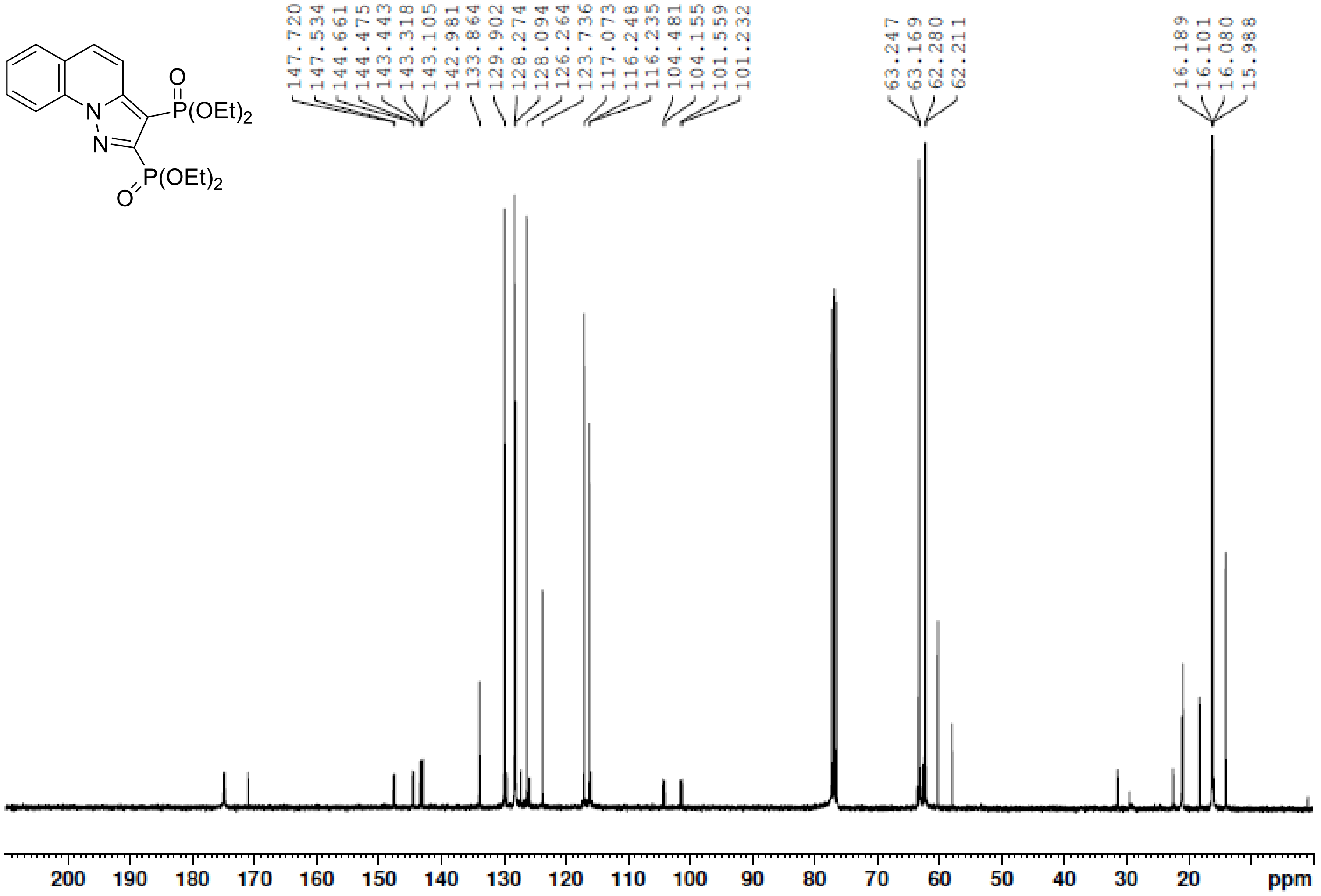

Tetraethyl pyrazolo[1,5-a]quinoline-2,3-diyldiphosphonate (5g) ^{31}P NMR

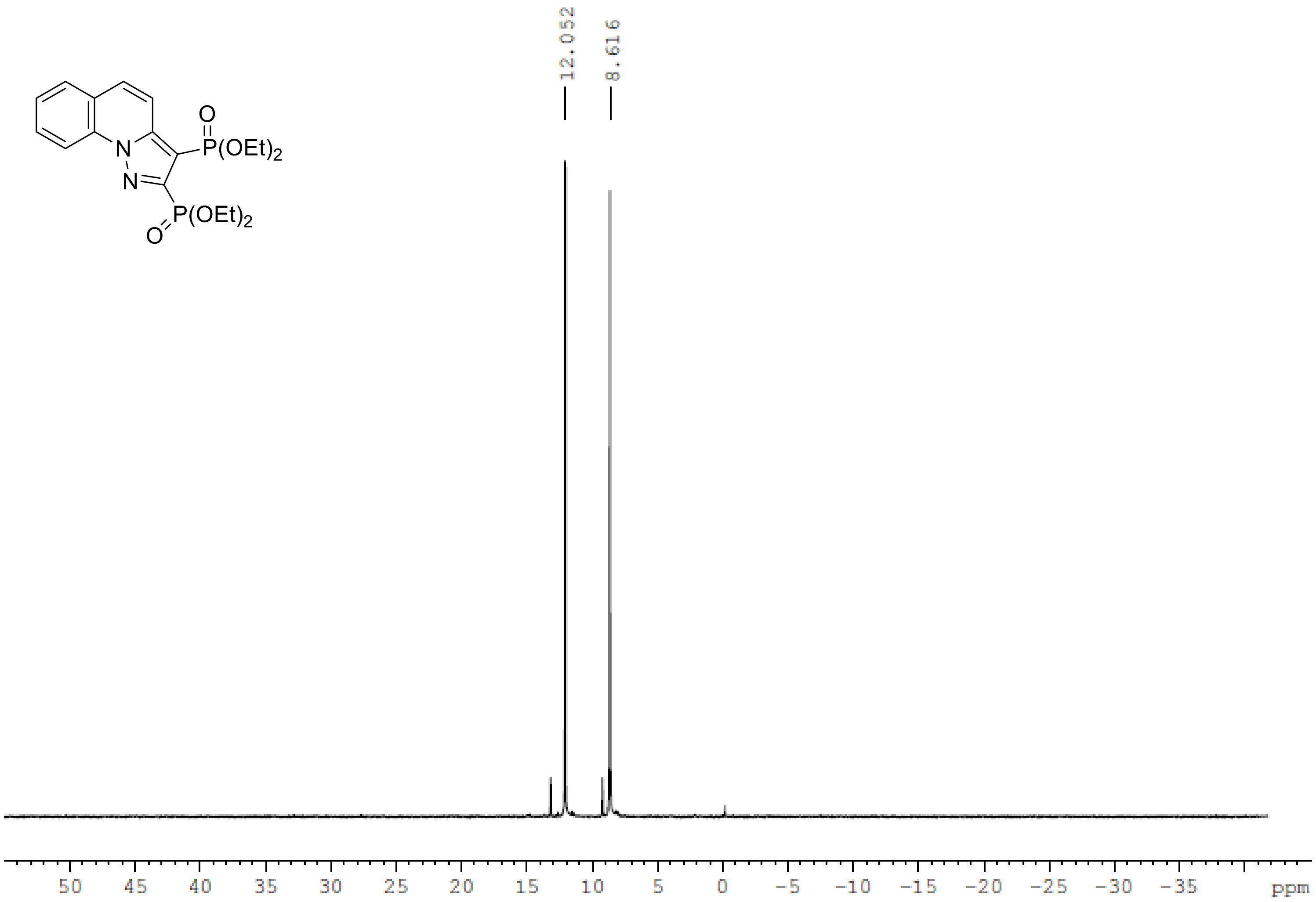

Tetraethyl (9-hydroxypyrazolo[1,5-a]quinoline-2,3-diyl)bis(phosphonate) (5h) ^{1}H NMR

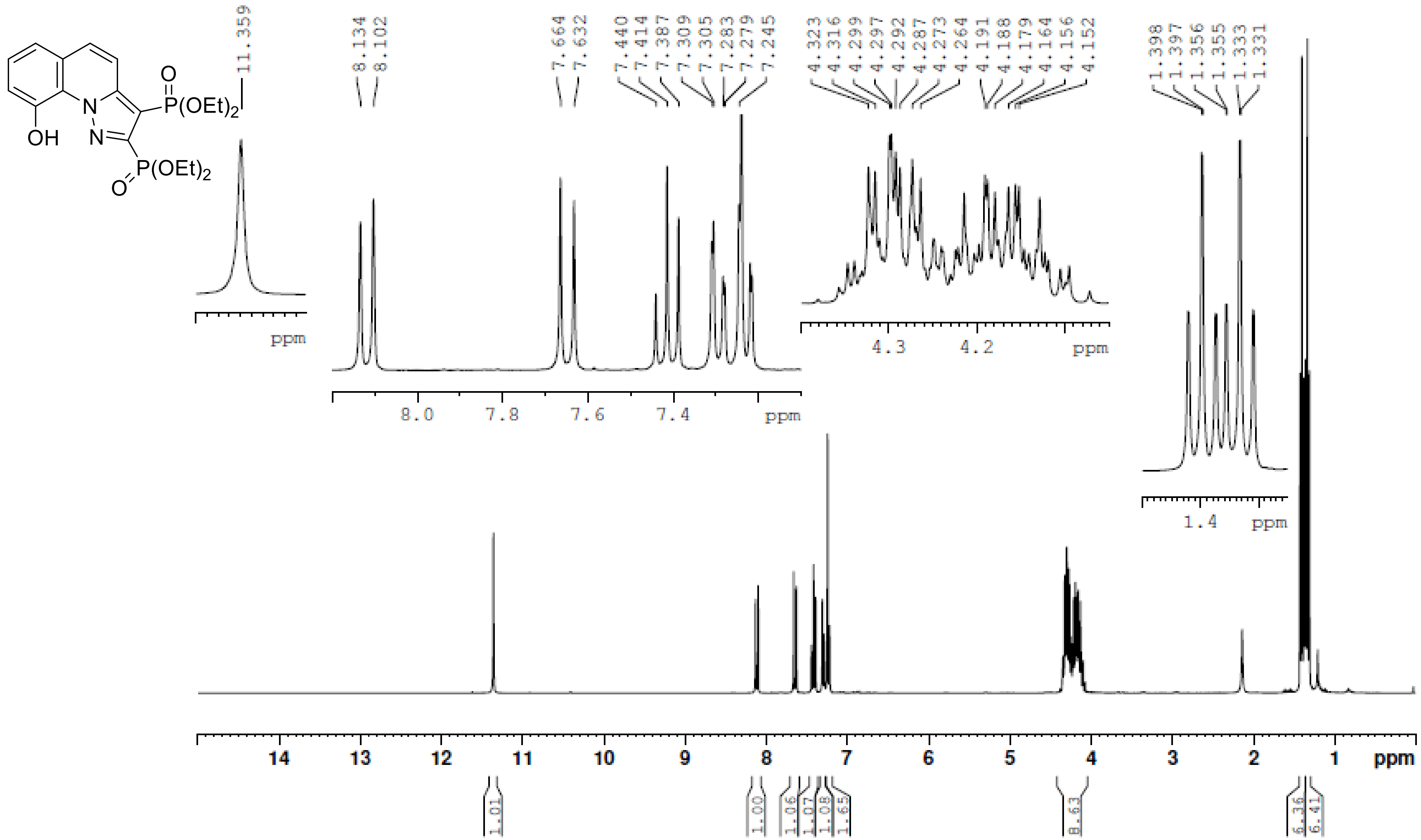

Tetraethyl (9-hydroxypyrazolo[1,5-a]quinoline-2,3-diyl)bis(phosphonate) (5h) ^{13}C NMR

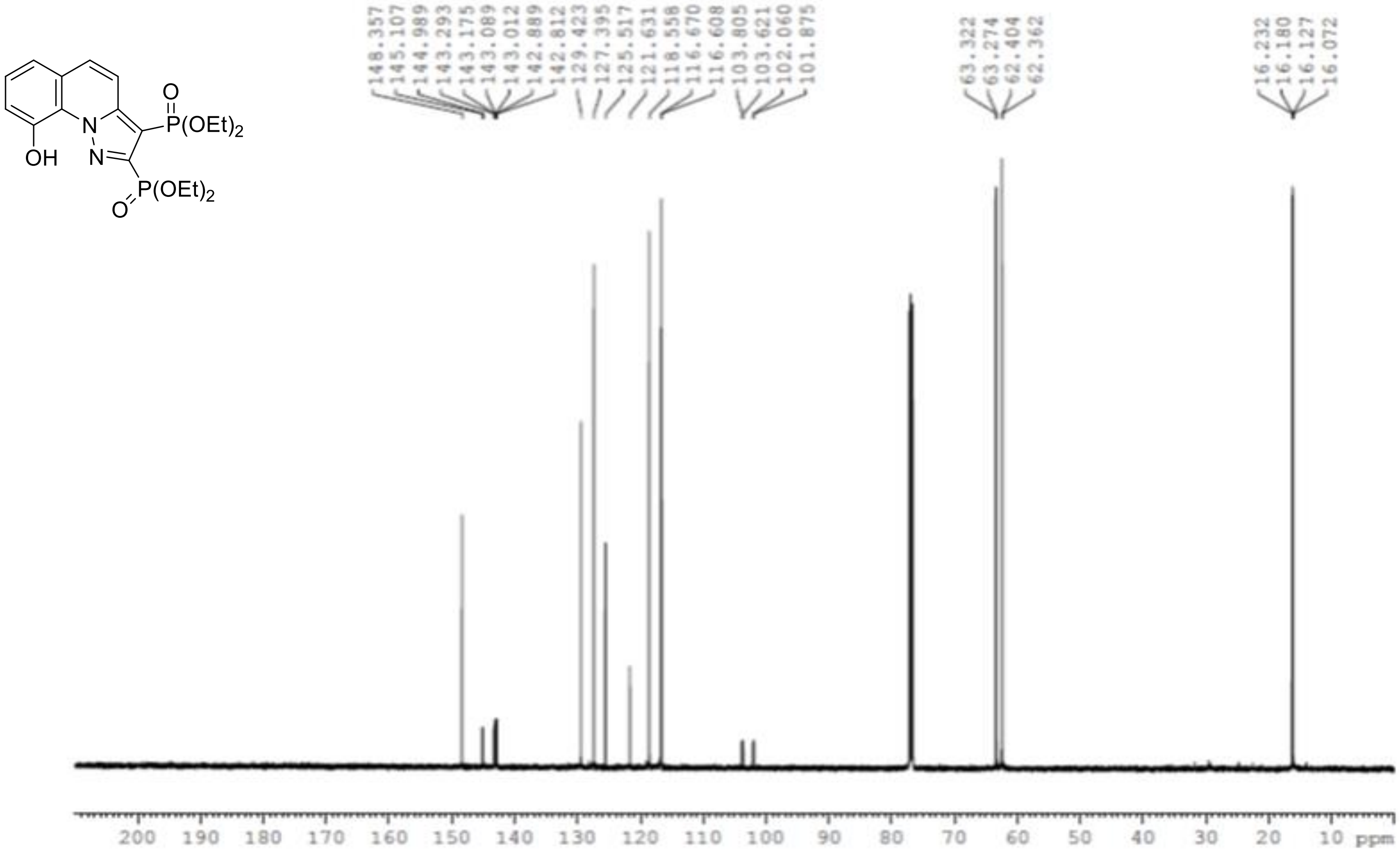

Tetraethyl (9-hydroxypyrazolo[1,5-a]quinoline-2,3-diyl)bis(phosphonate) (5h) ^{31}P NMR

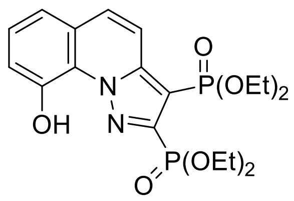

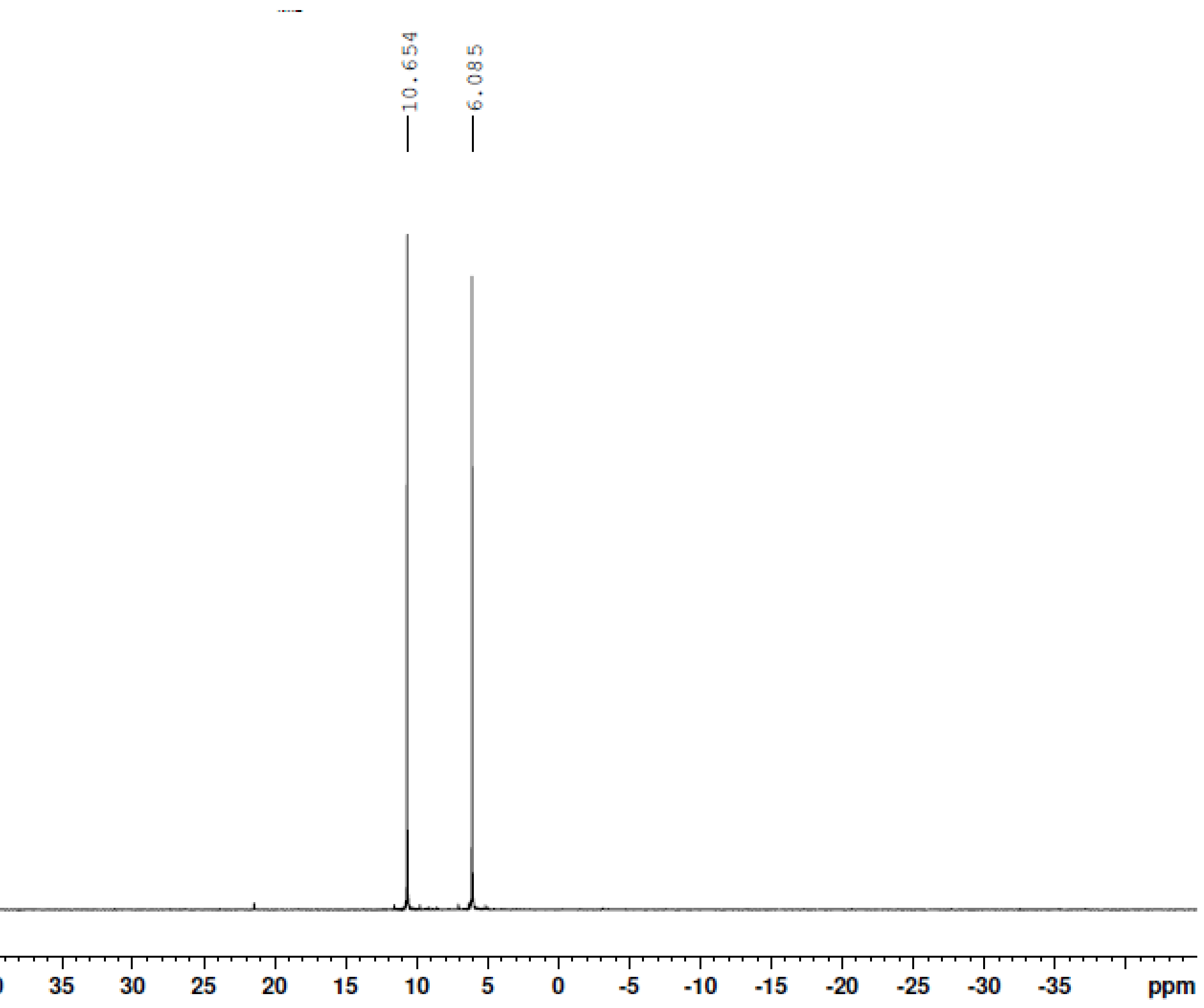

Tetraethyl pyrazolo[5,1-a]isoquinoline-1,2-diylbis(phosphonate) (5i) ^{1}H NMR

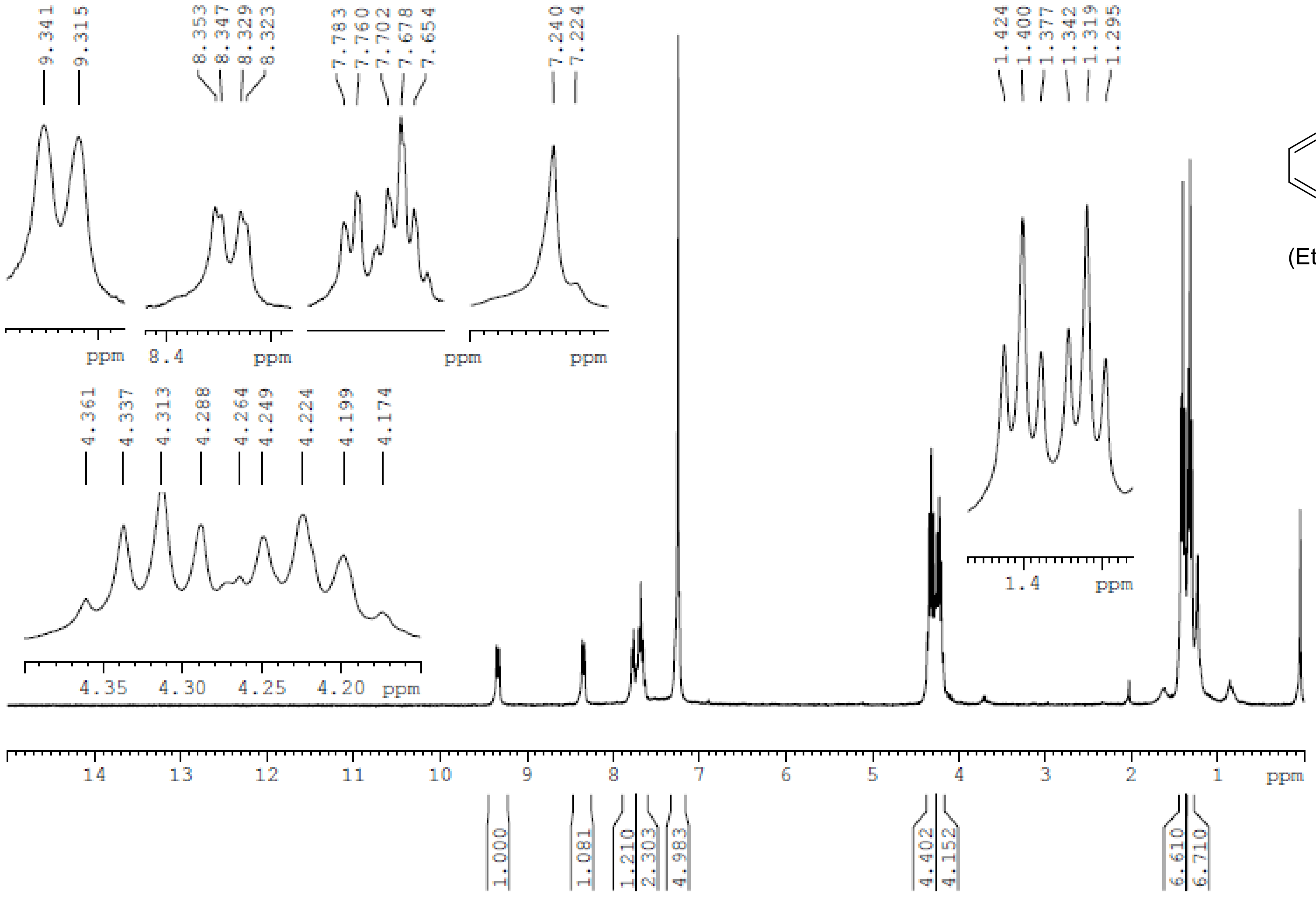

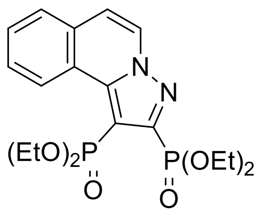

Tetraethyl pyrazolo[5,1-a]isoquinoline-1,2-diylbis(phosphonate) (5i) ^{13}C NMR

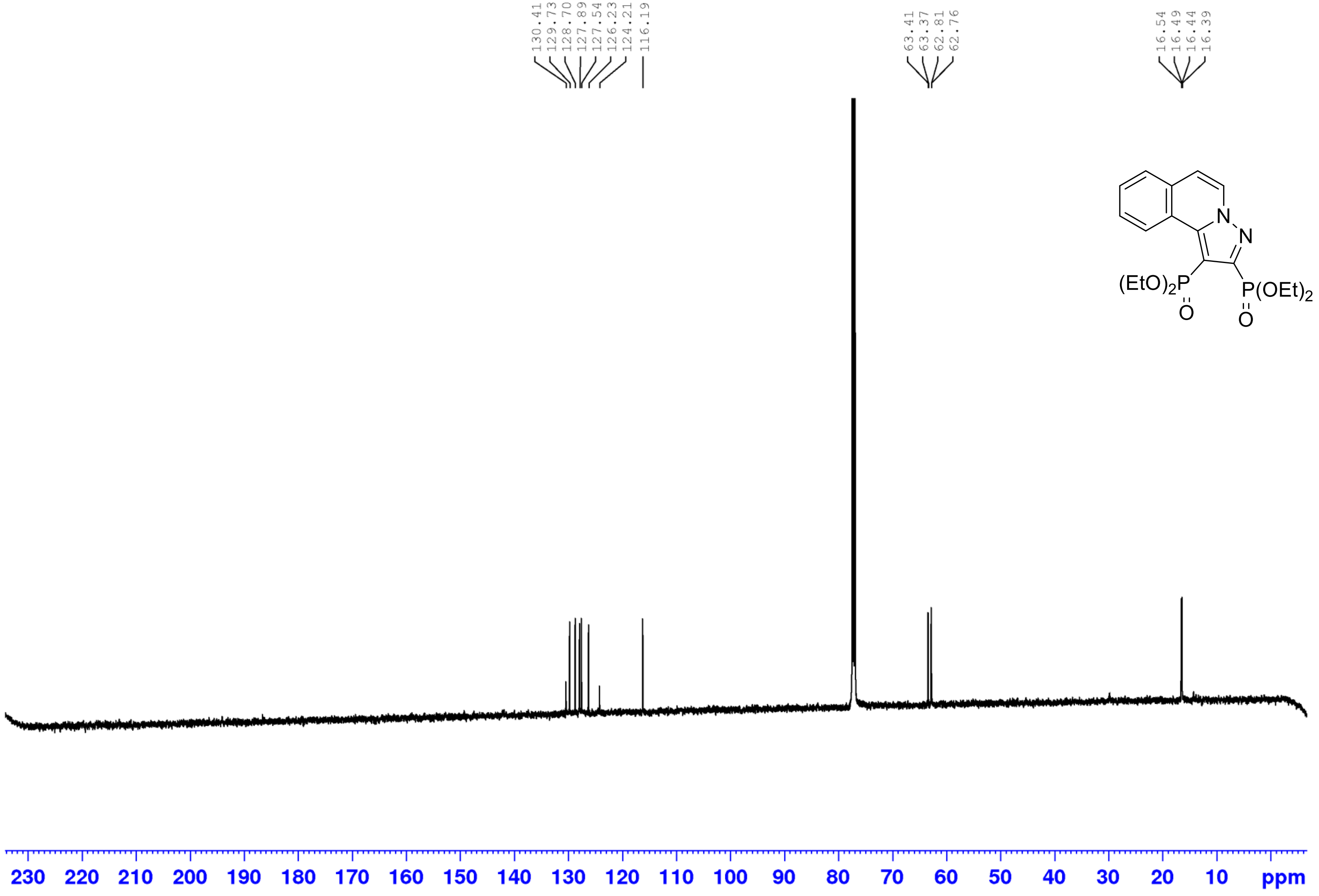

Tetraethyl pyrazolo[5,1-a]isoquinoline-1,2-diylbis(phosphonate) (5i) ^{31}P NMR

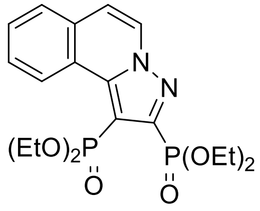

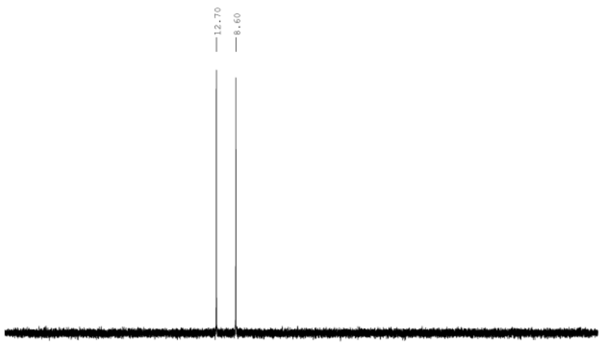

Tetraethyl pyrazolo[1,5-a][1,10]phenanthroline-2,3-diylbis(phosphonate) (5j) ^{1}H NMR

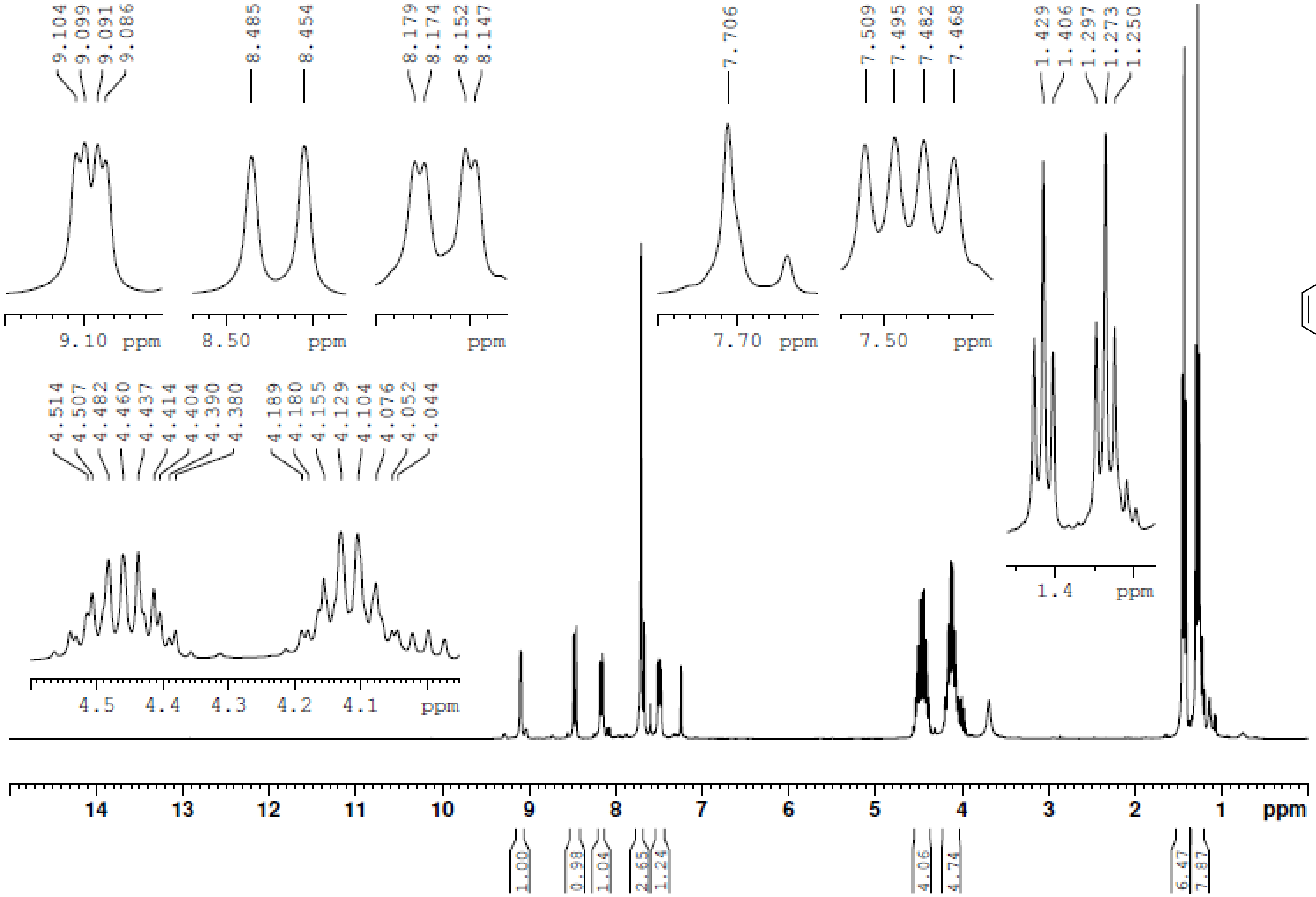

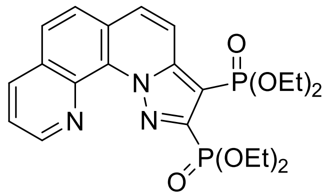

Tetraethyl pyrazolo[1,5-a][1,10]phenanthroline-2,3-diylbis(phosphonate) (5j) ^{13}C NMR

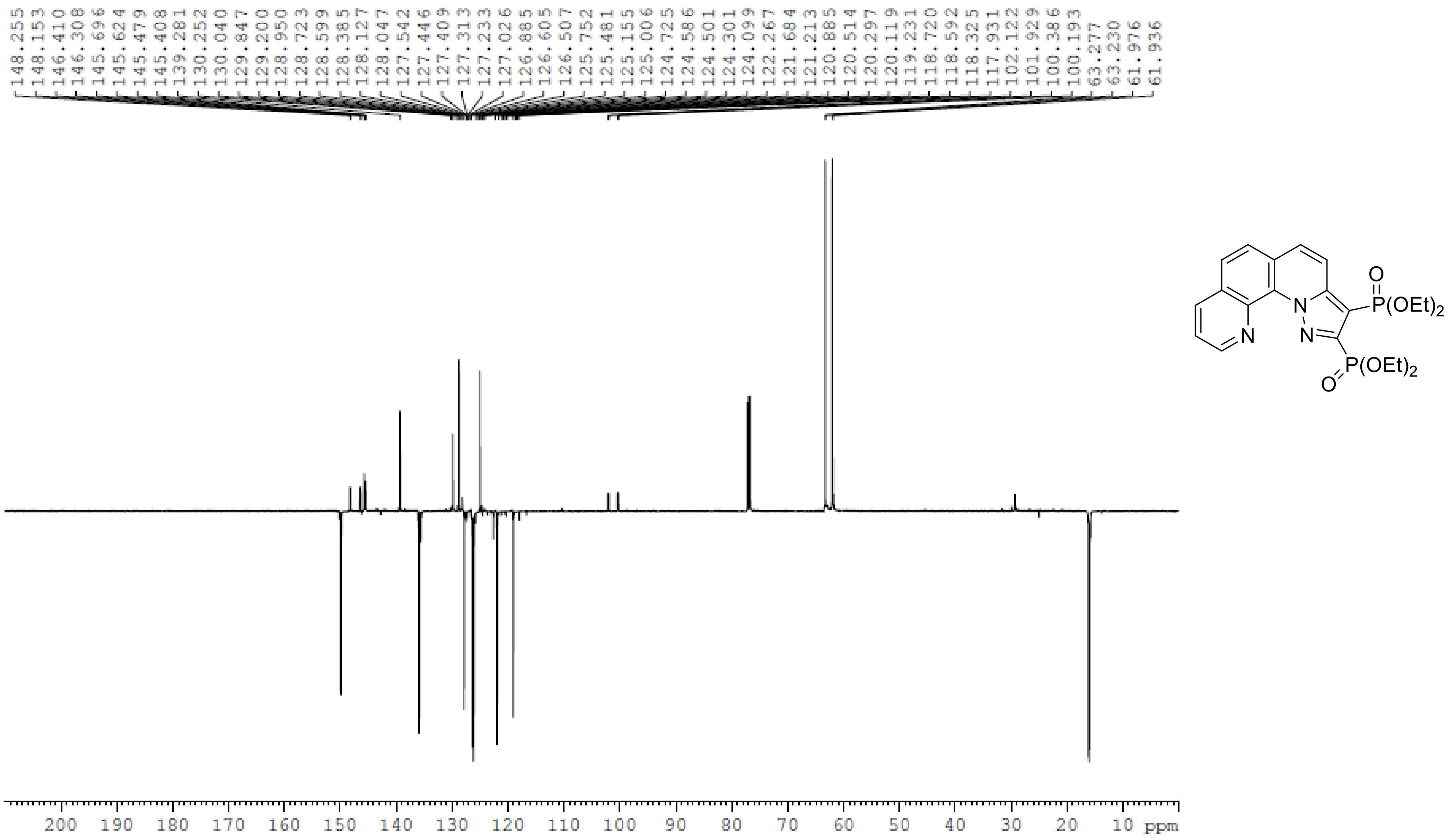

Tetraethyl pyrazolo[1,5-a][1,10]phenanthroline-2,3-diylbis(phosphonate) (5j) ^{31}P NMR

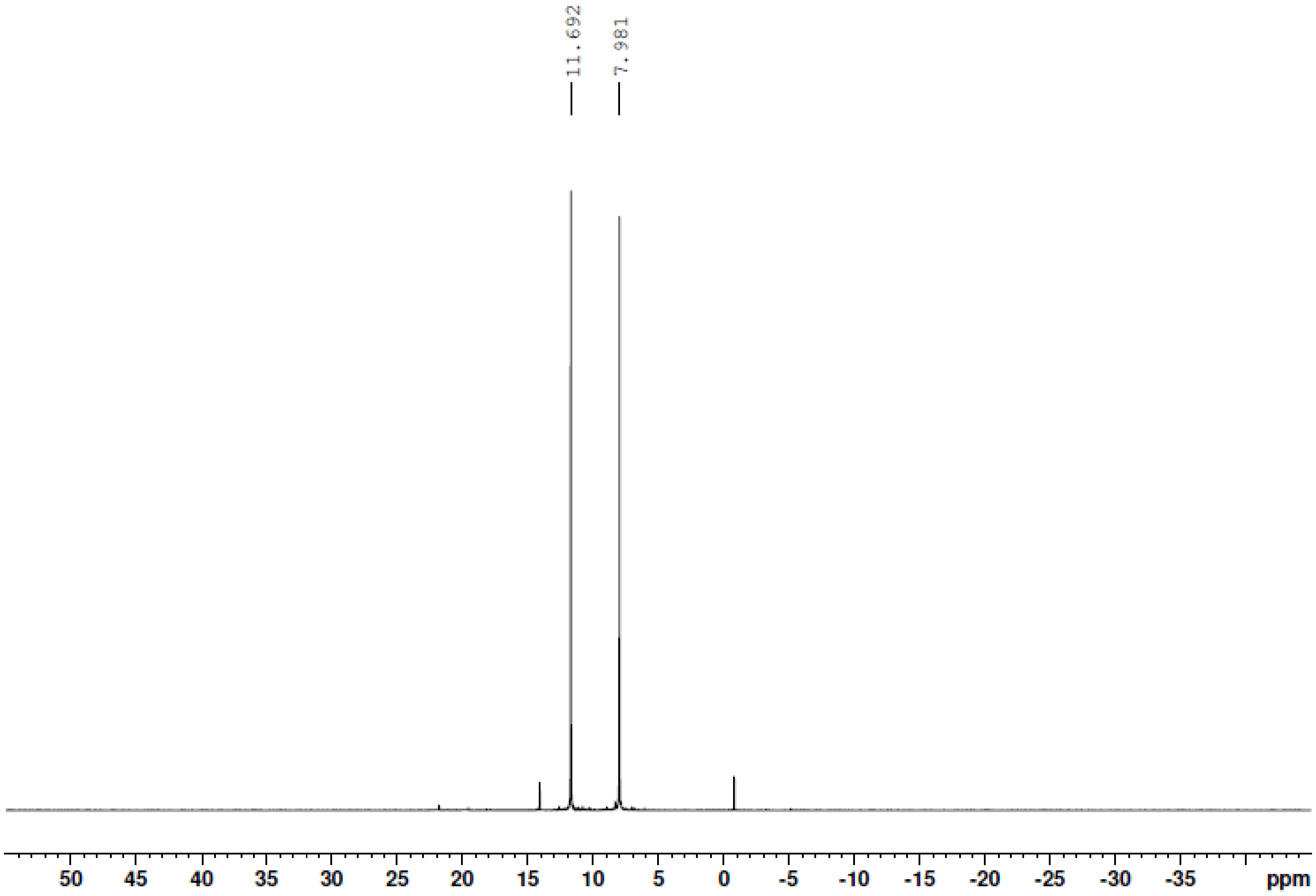

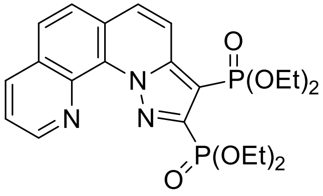

Diethyl (pyrazolo[1,5-a]pyridin-3-yl)phosphonate ^{1}H NMR (5k).

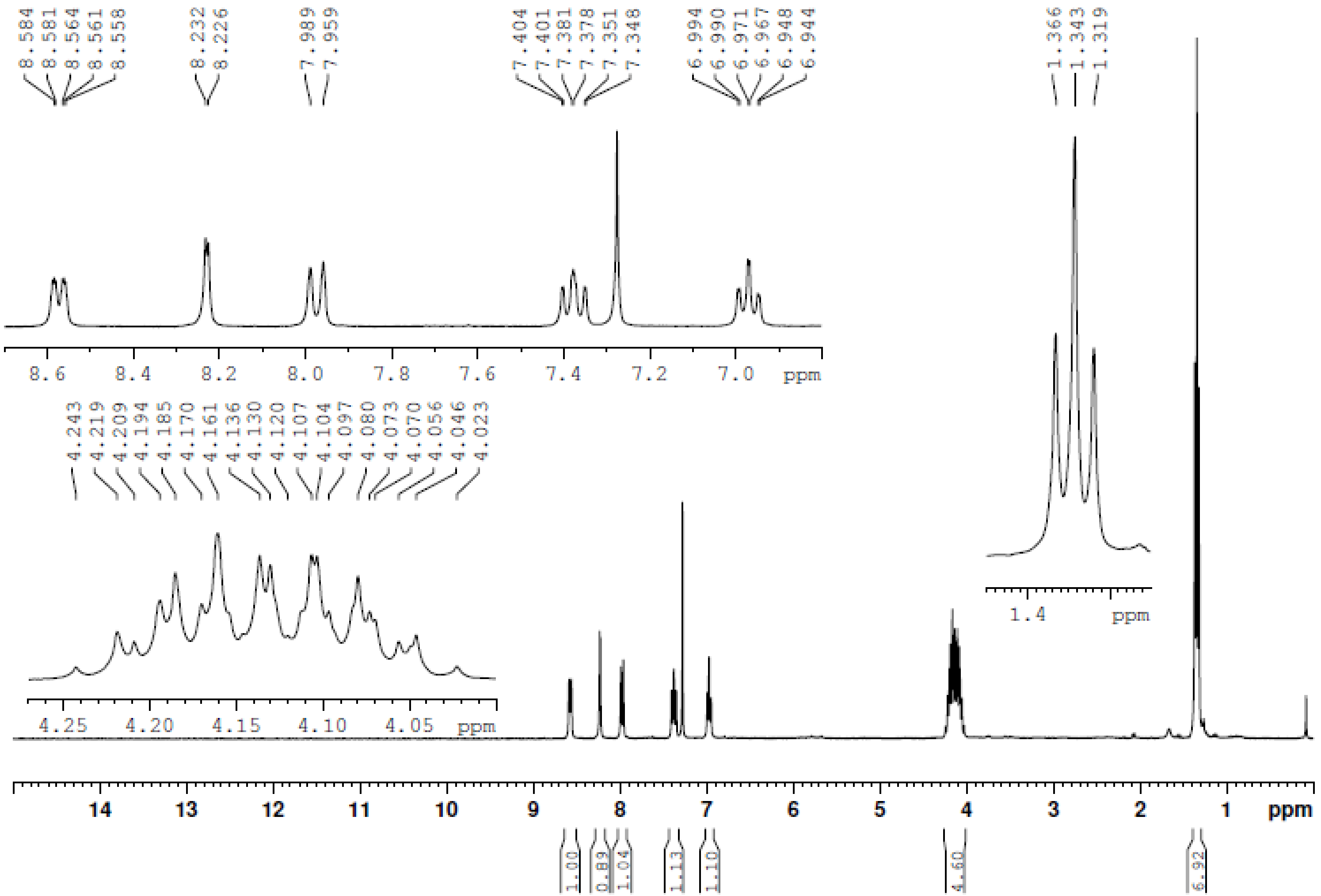

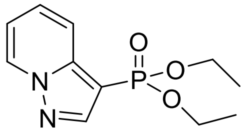

Diethyl (pyrazolo[1,5-a]pyridin-3-yl)phosphonate ^{13}C NMR (5k).

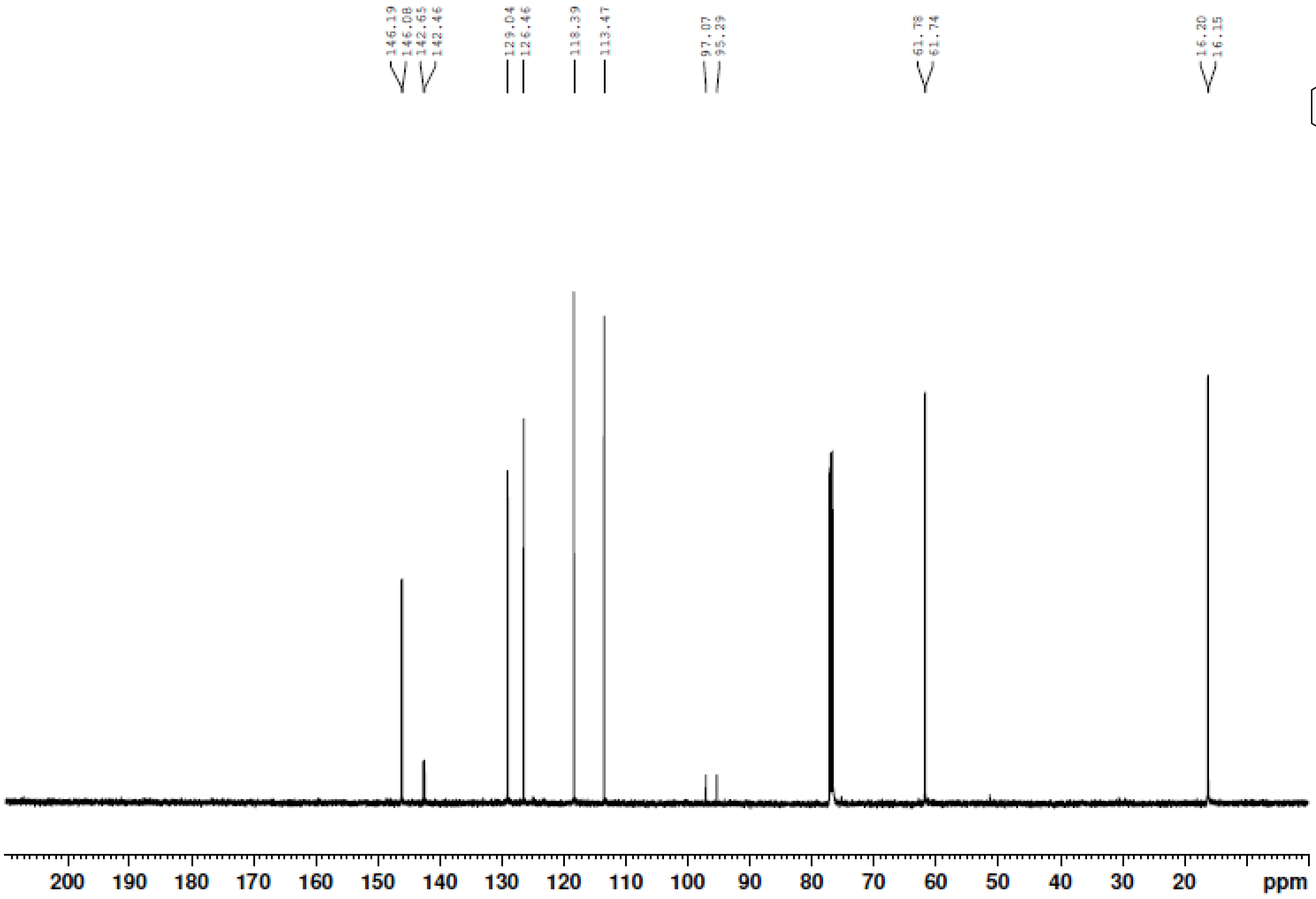

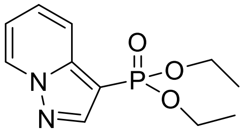

Diethyl (pyrazolo[1,5-a]pyridin-3-yl)phosphonate ^{31}P NMR (5k).

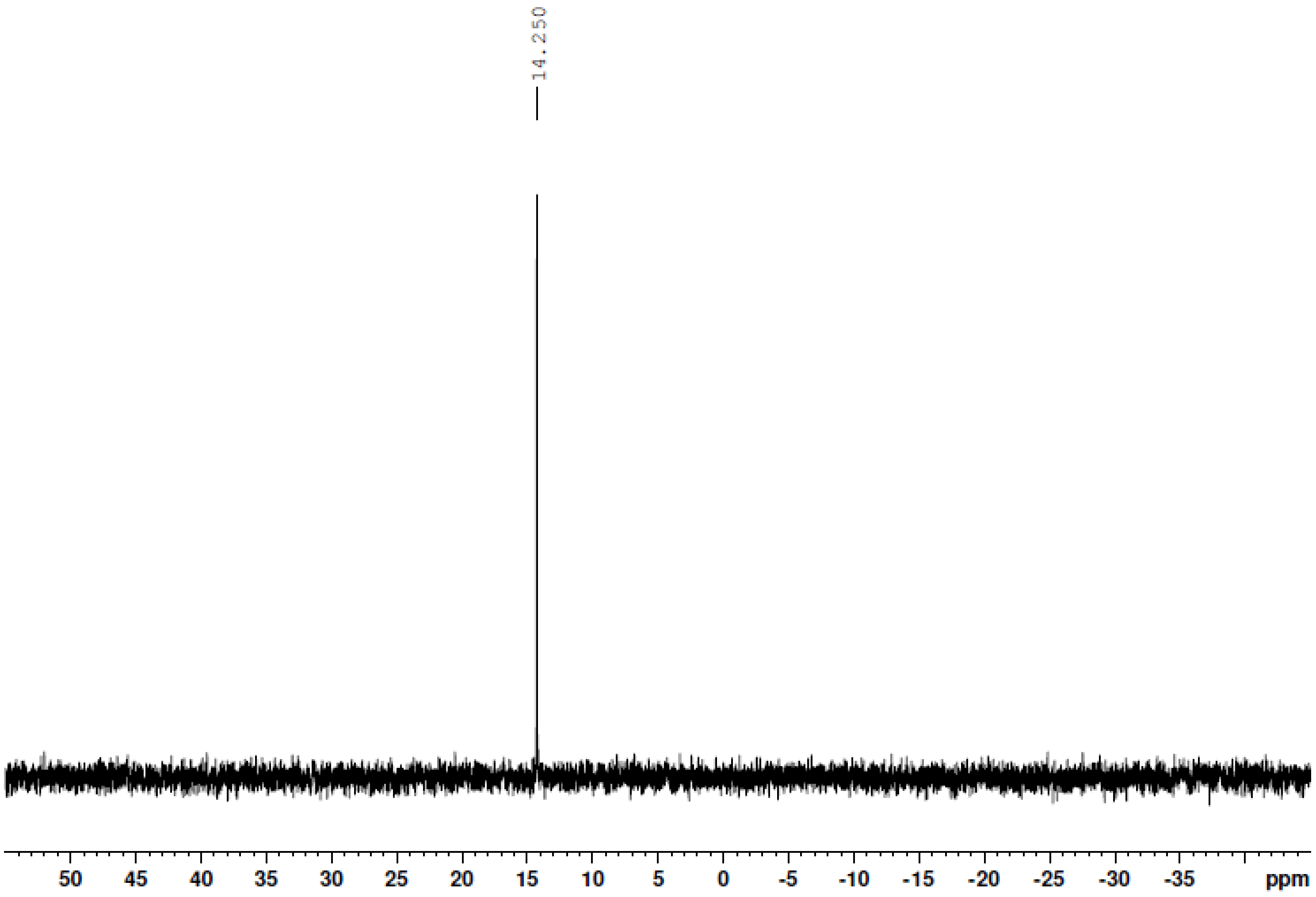

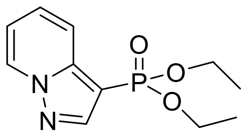

Diethyl (5-methylpyrazolo[1,5-a]pyridin-3-yl)phosphonate (5l) ^{1}H NMR.

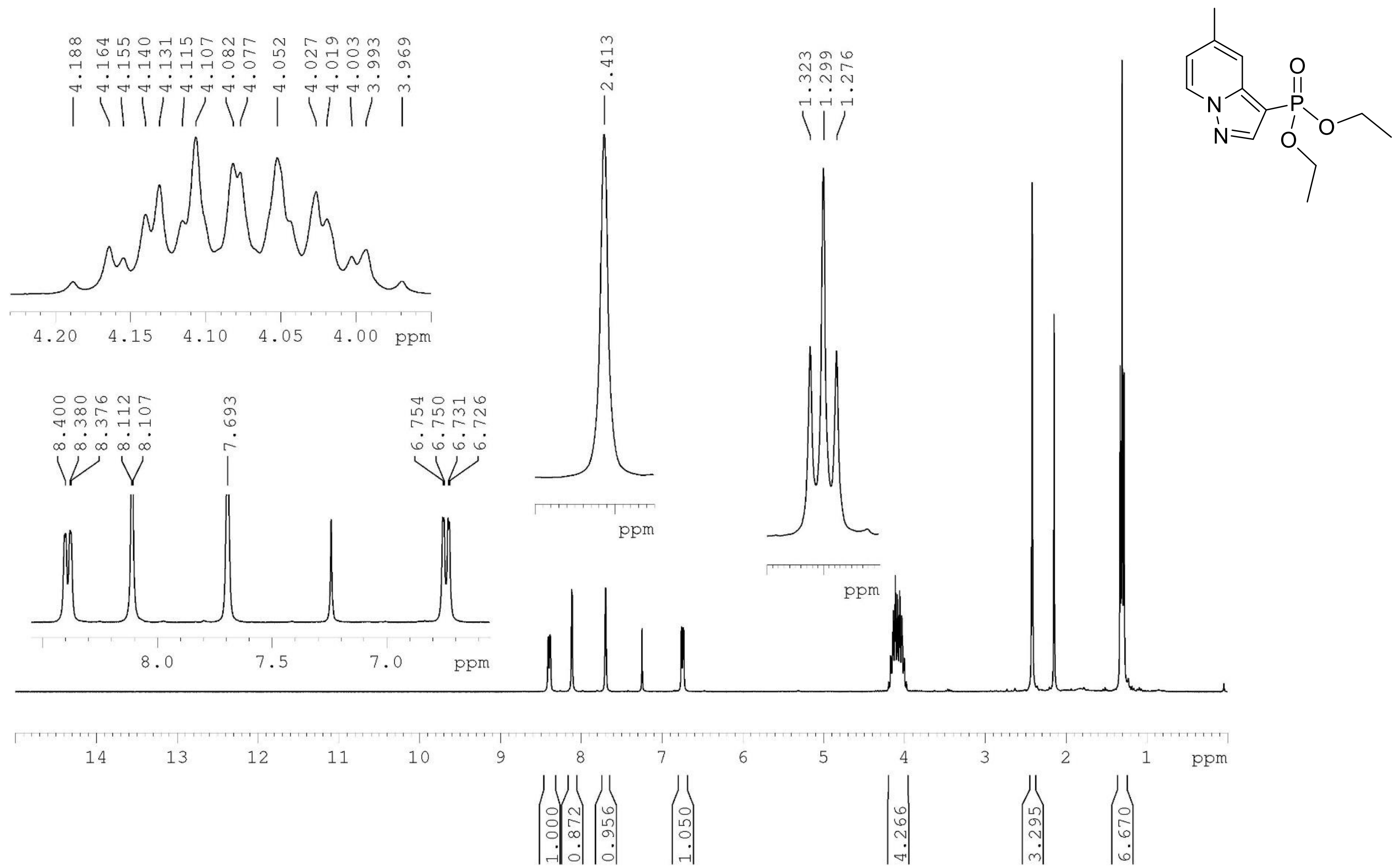

Diethyl (5-methylpyrazolo[1,5-a]pyridin-3-yl)phosphonate (5l) ^{13}C NMR.

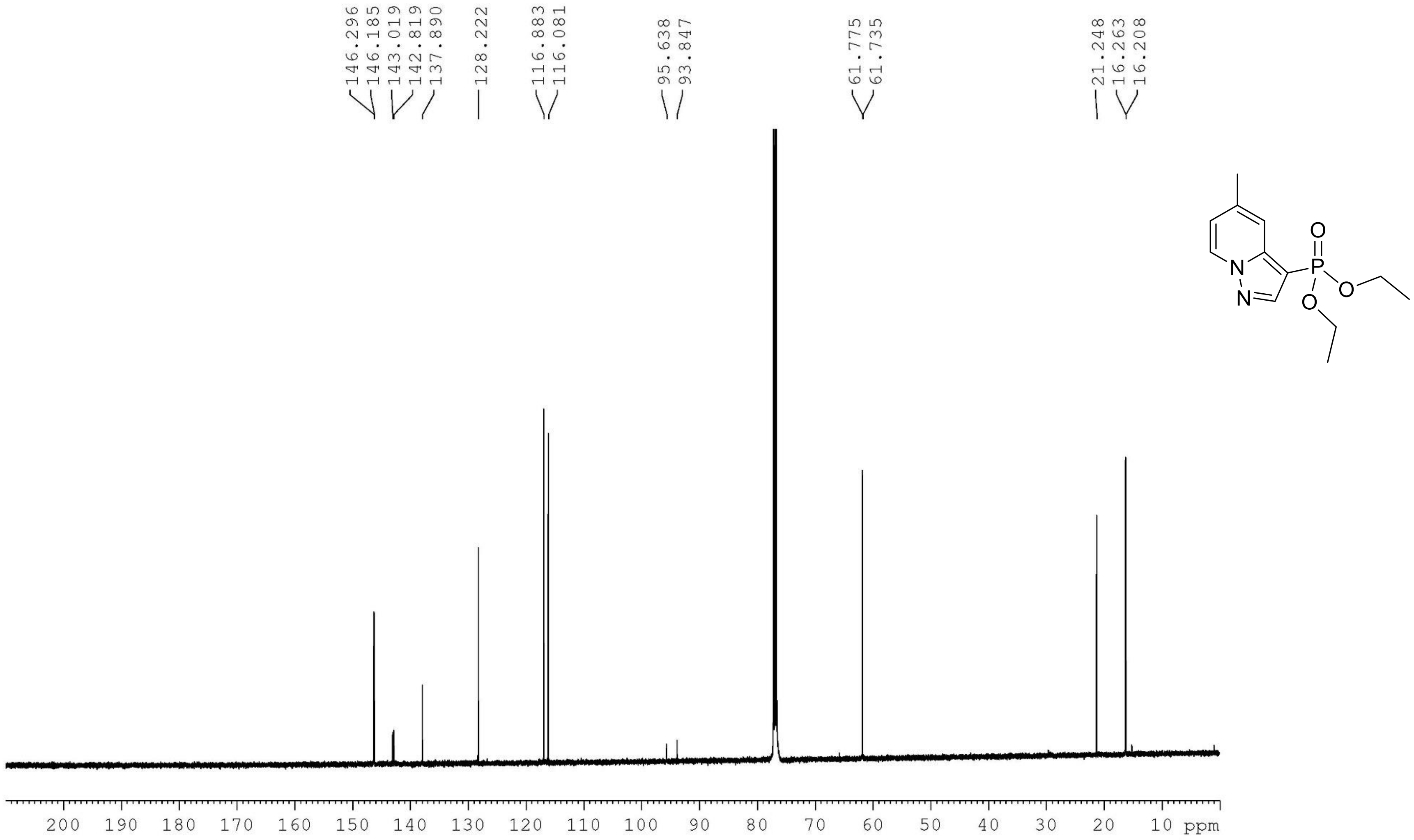

Diethyl (5-methylpyrazolo[1,5-a]pyridin-3-yl)phosphonate (5l) ^{31}P NMR.

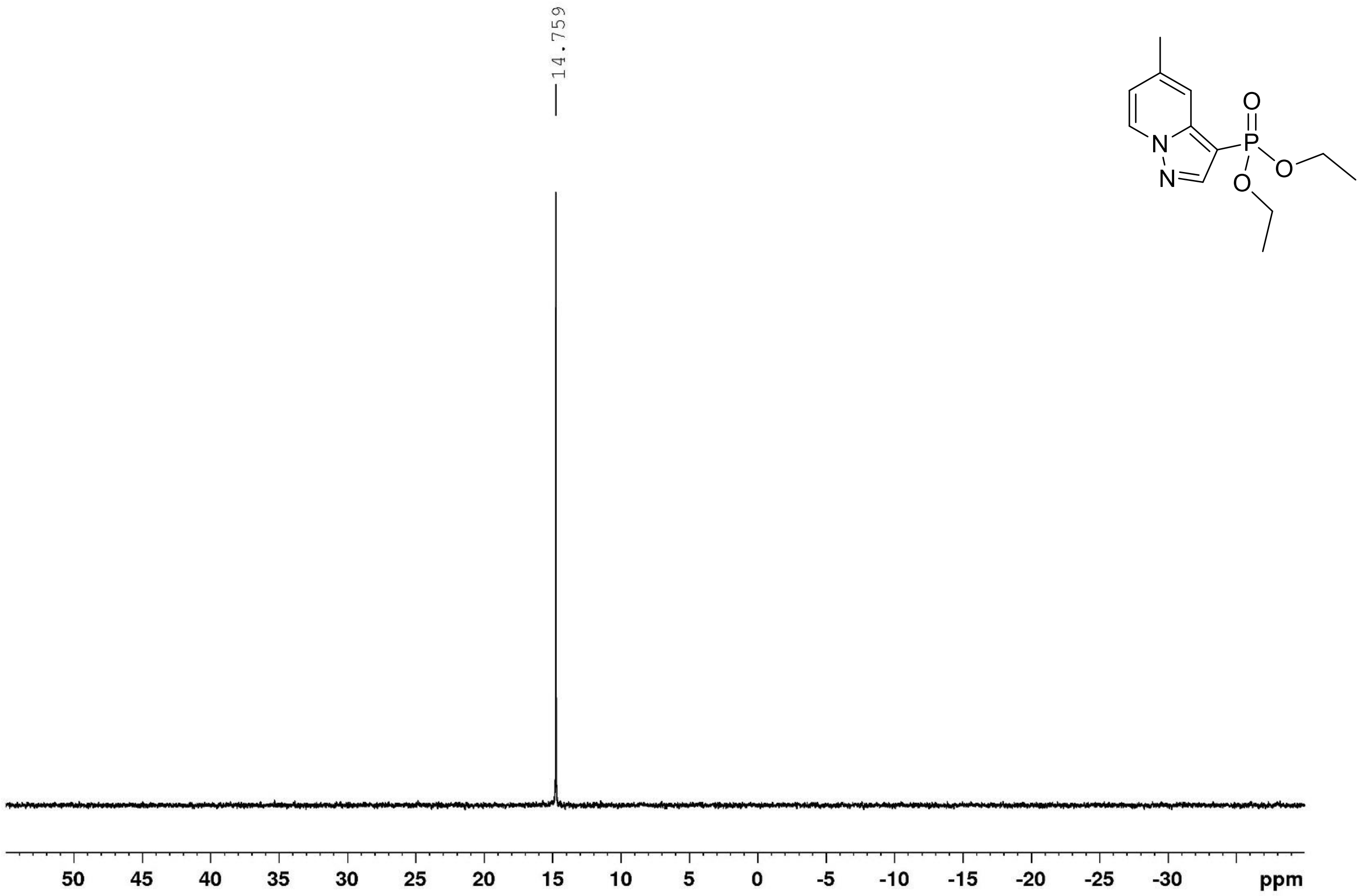

Diethyl (7-methylpyrazolo[1,5-a]pyridin-3-yl)phosphonate (5m) ^{1}H NMR.

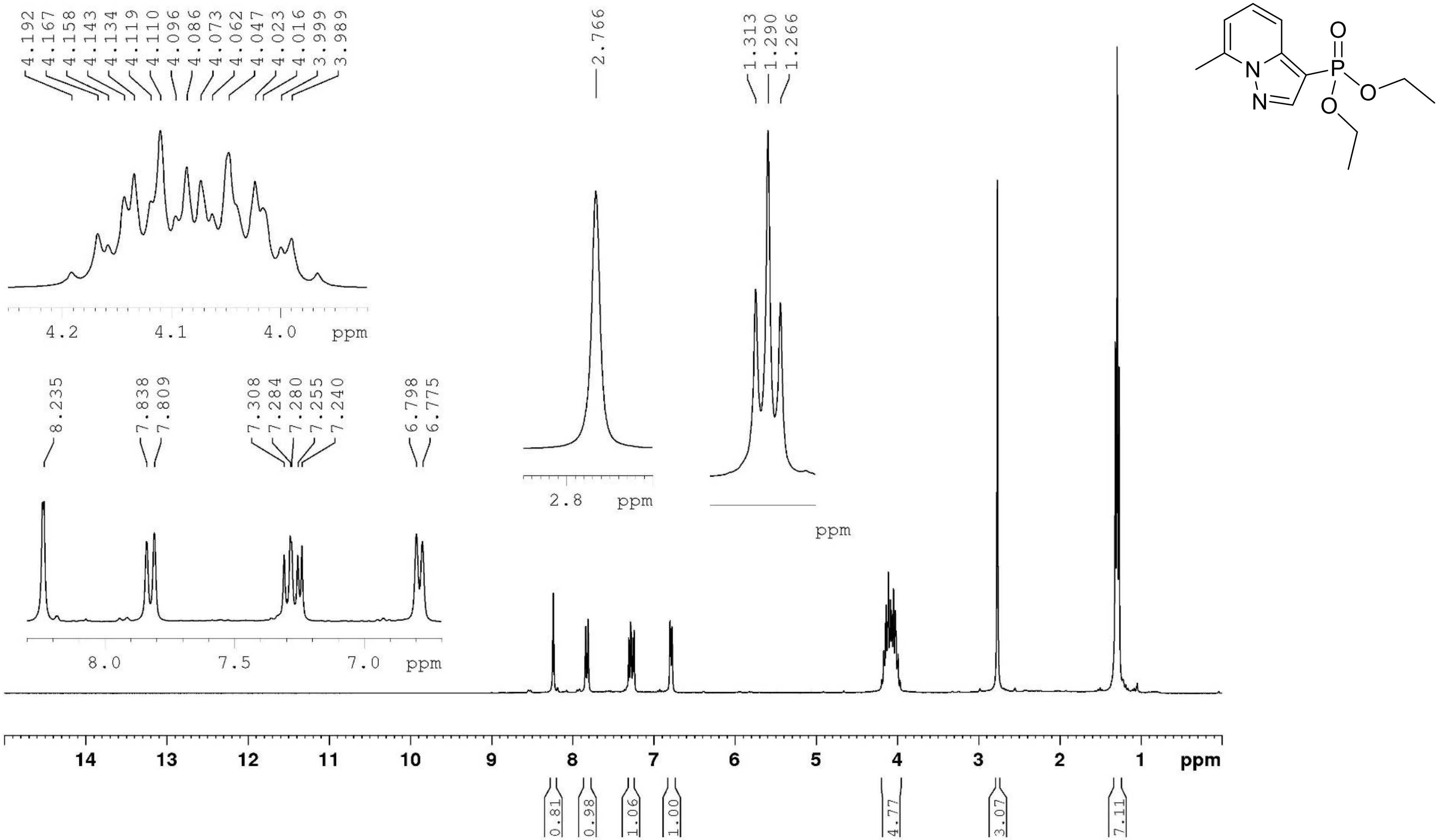

Diethyl (7-methylpyrazolo[1,5-a]pyridin-3-yl)phosphonate (5m) ^{13}C NMR.

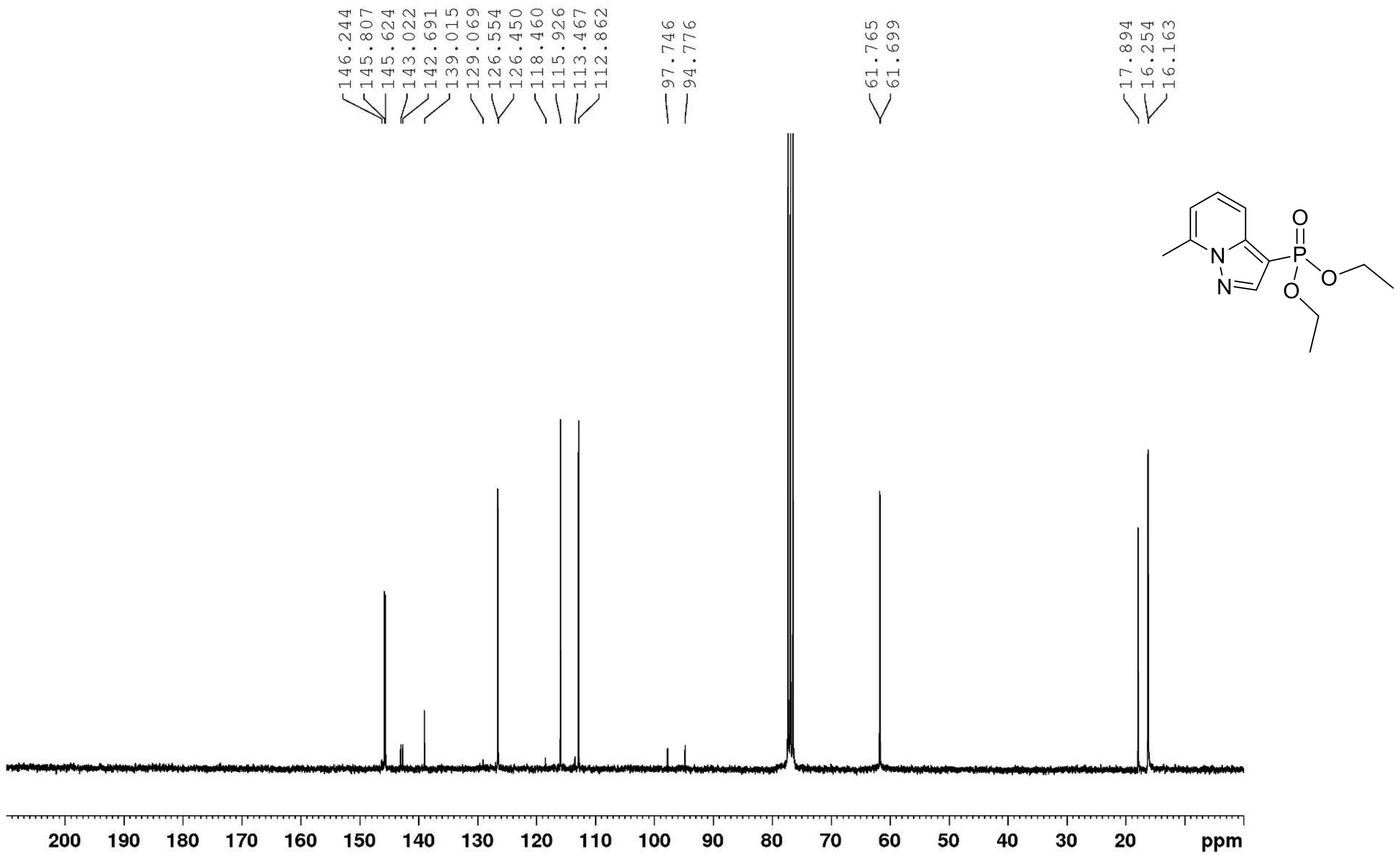

Diethyl (7-methylpyrazolo[1,5-a]pyridin-3-yl)phosphonate (5m) ^{31}P NMR.

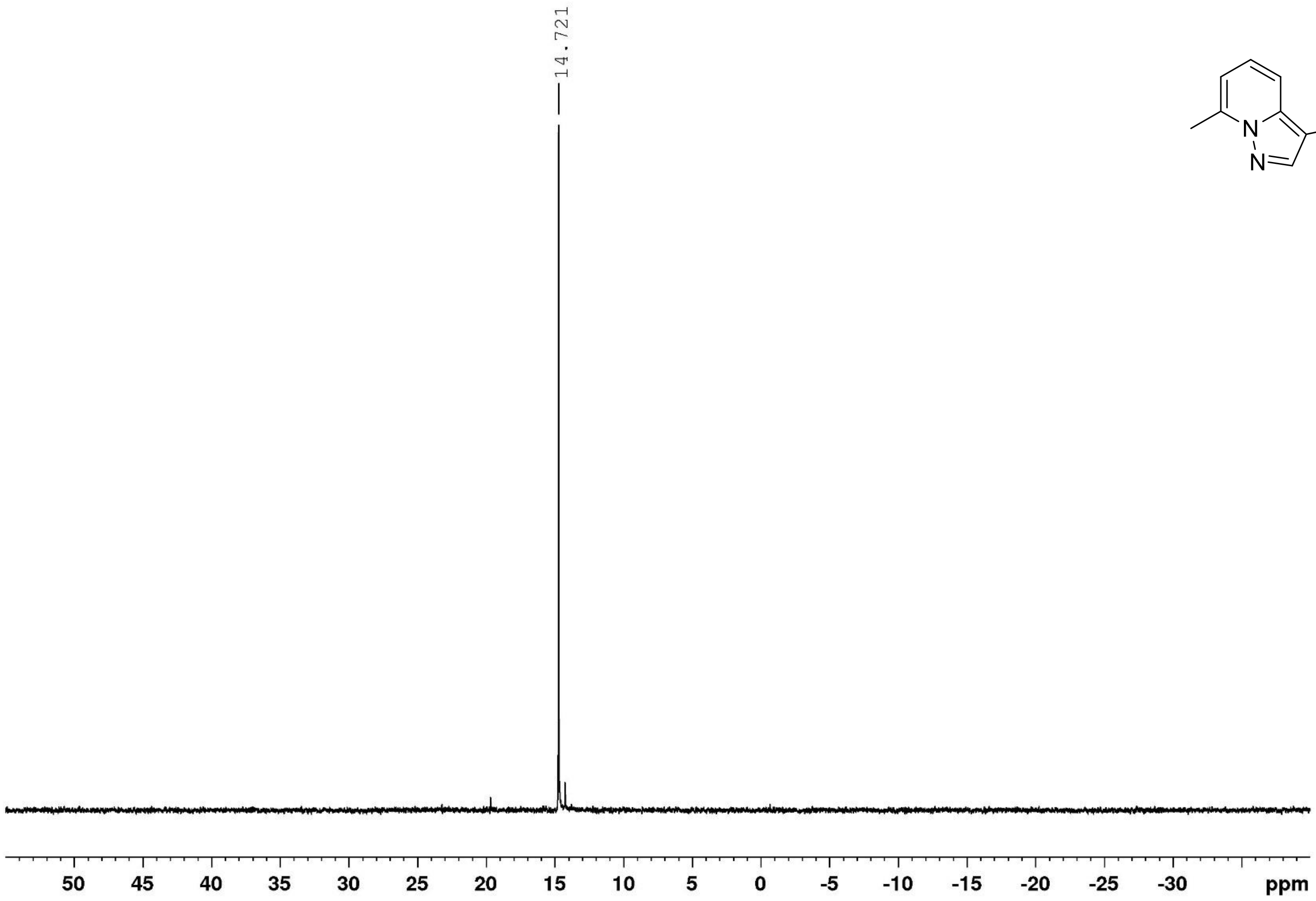

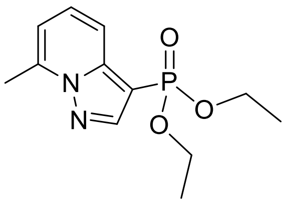

Diethyl (7-phenylpyrazolo[1,5-a]pyridin-3-yl)phosphonate (5n) ^{1}H NMR.

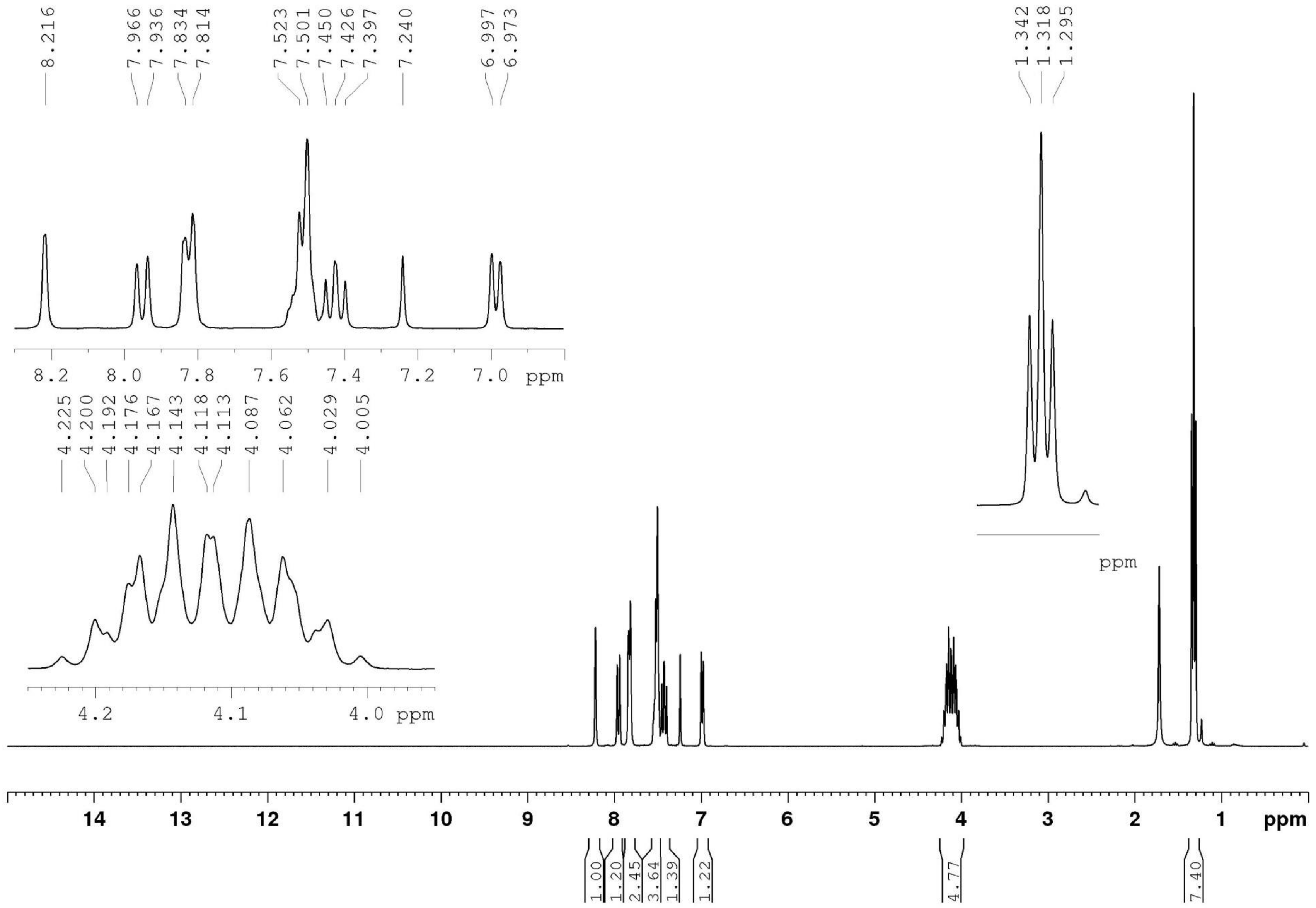

Diethyl (7-phenylpyrazolo[1,5-a]pyridin-3-yl)phosphonate (5n) ^{13}C NMR.

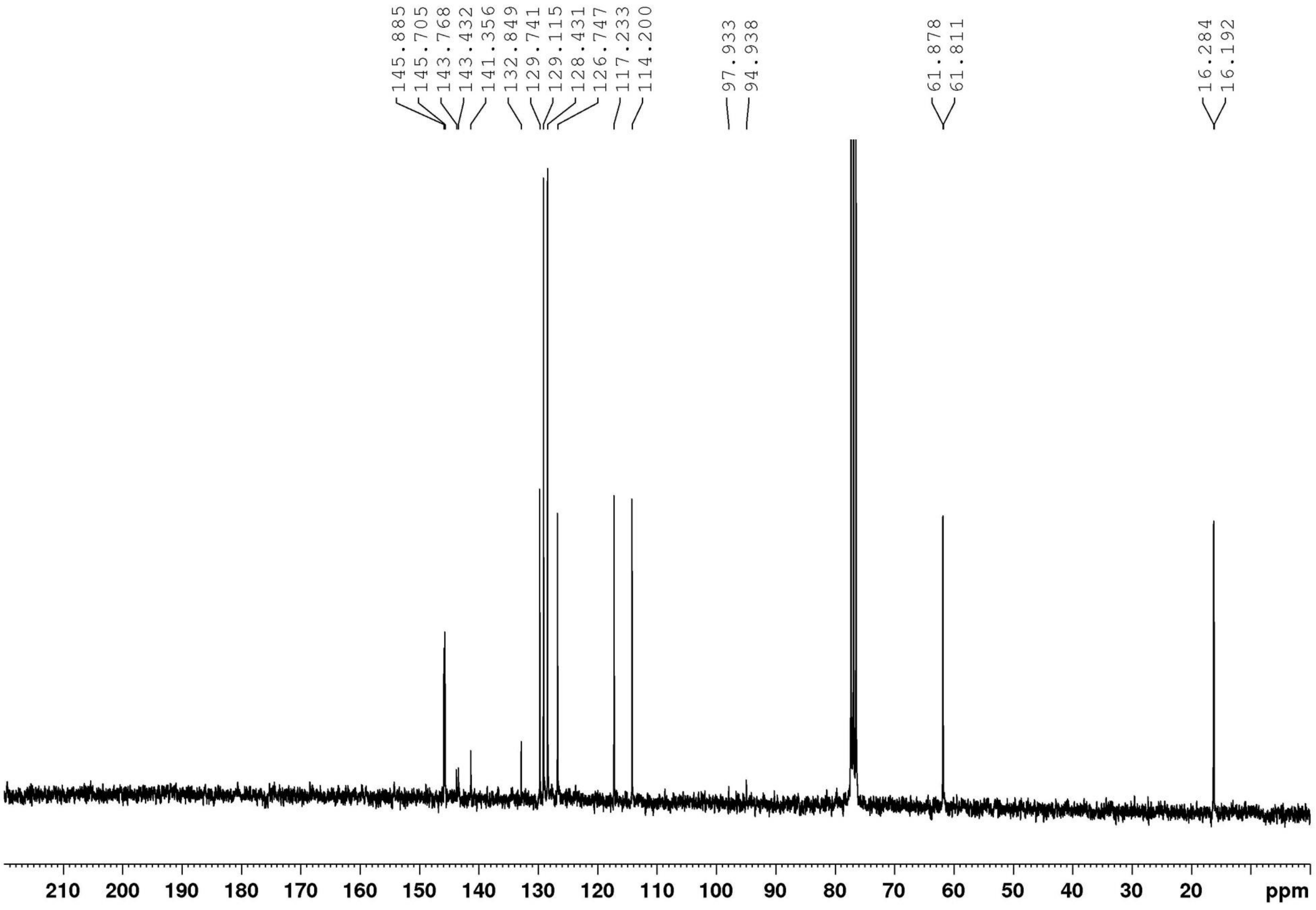

Diethyl (7-phenylpyrazolo[1,5-a]pyridin-3-yl)phosphonate (5n) ^{31}P NMR.

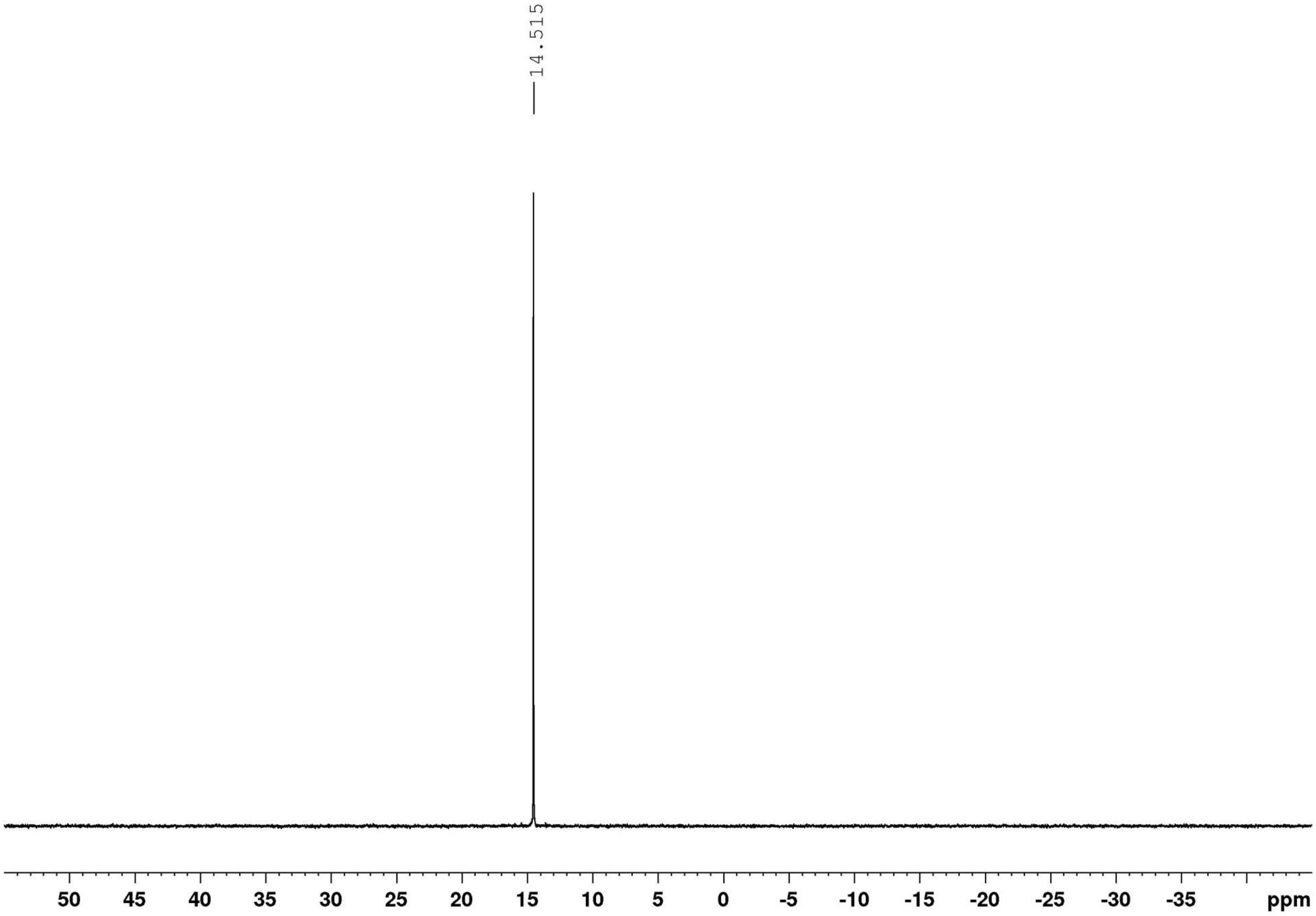

Methyl 3-dietoxyphosphorylpyrazolo[1,5-a]pyridine-5-carboxylate (5o) ^{1}H NMR.

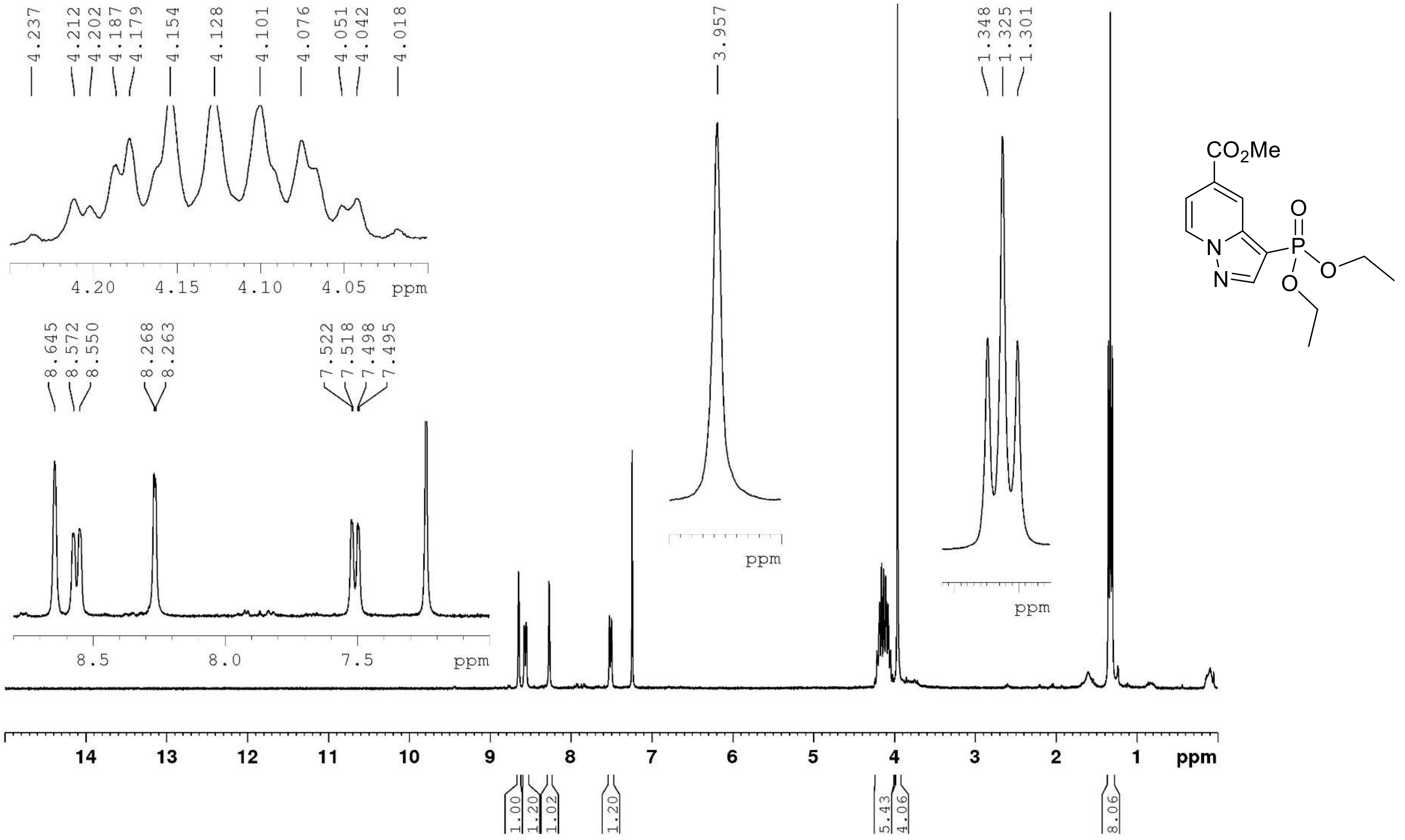

Methyl 3-dietoxyphosphorylpyrazolo[1,5-a]pyridine-5-carboxylate (5o) ^{13}C NMR.

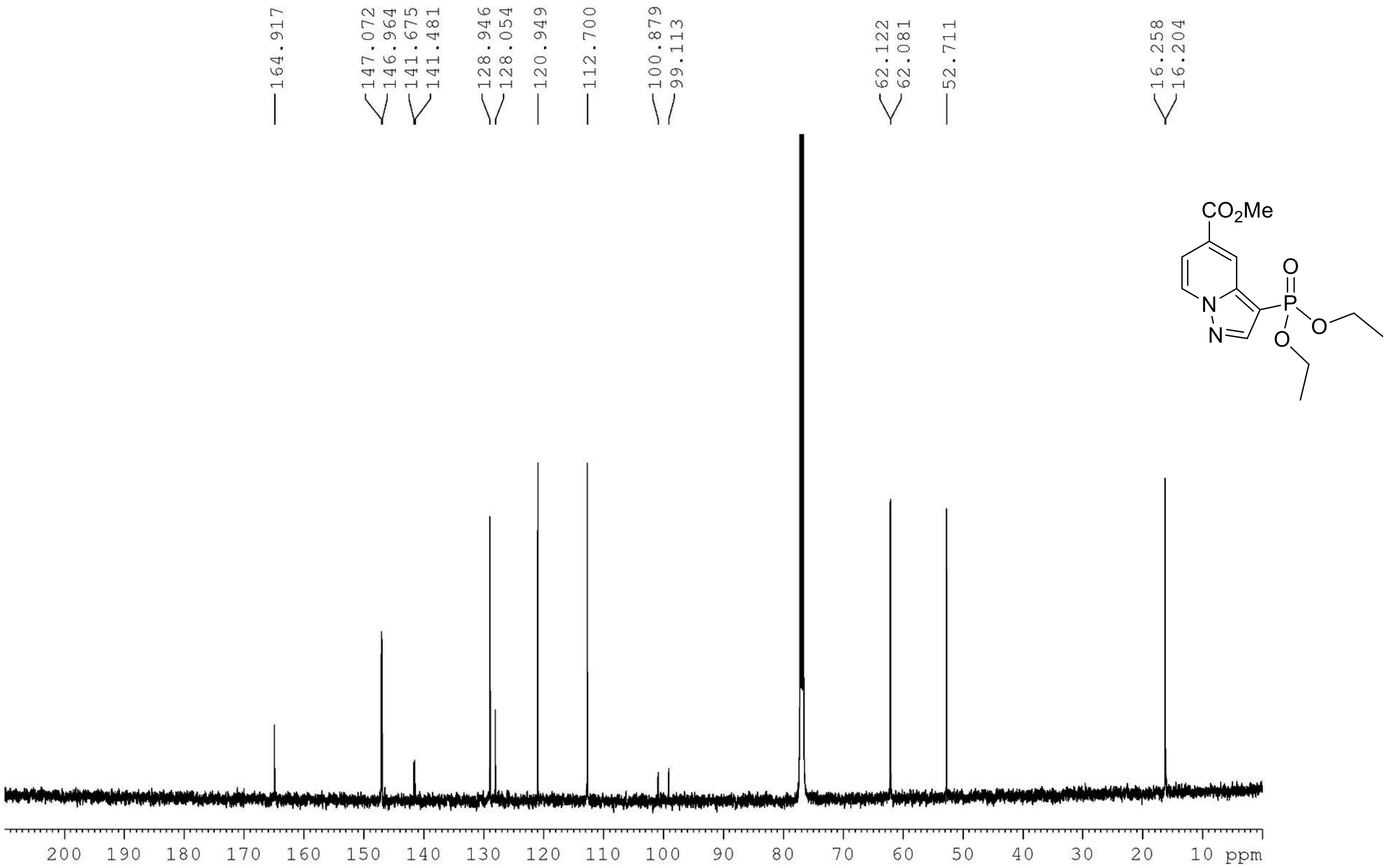

Methyl 3-dietoxyphosphorylpyrazolo[1,5-a]pyridine-5-carboxylate (5o) ^{31}P NMR.

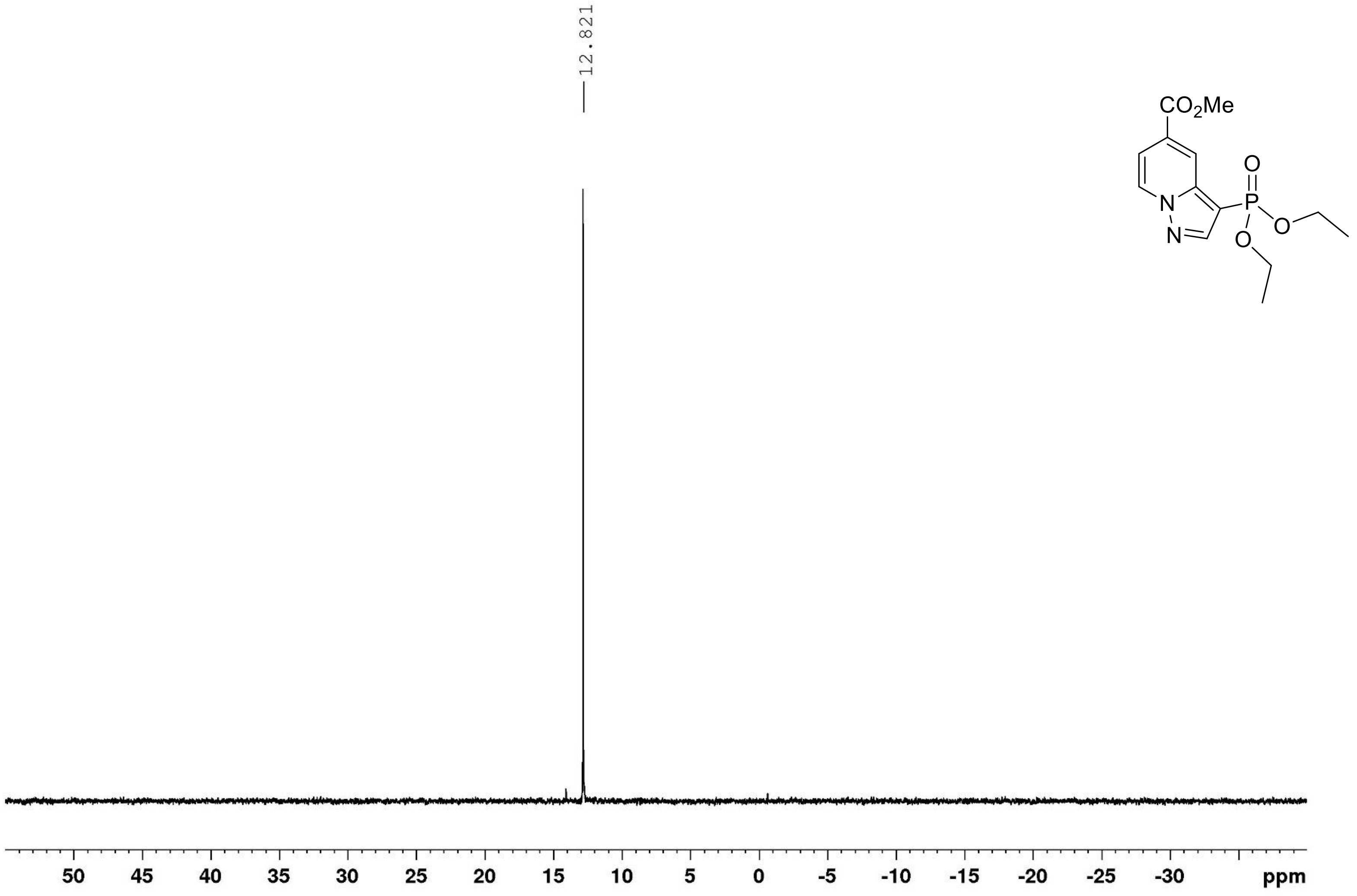

Diethyl (9-hydroxypyrazolo[1,5-a]quinoline-3-yl)phosphonate (5p) ^{1}H NMR.

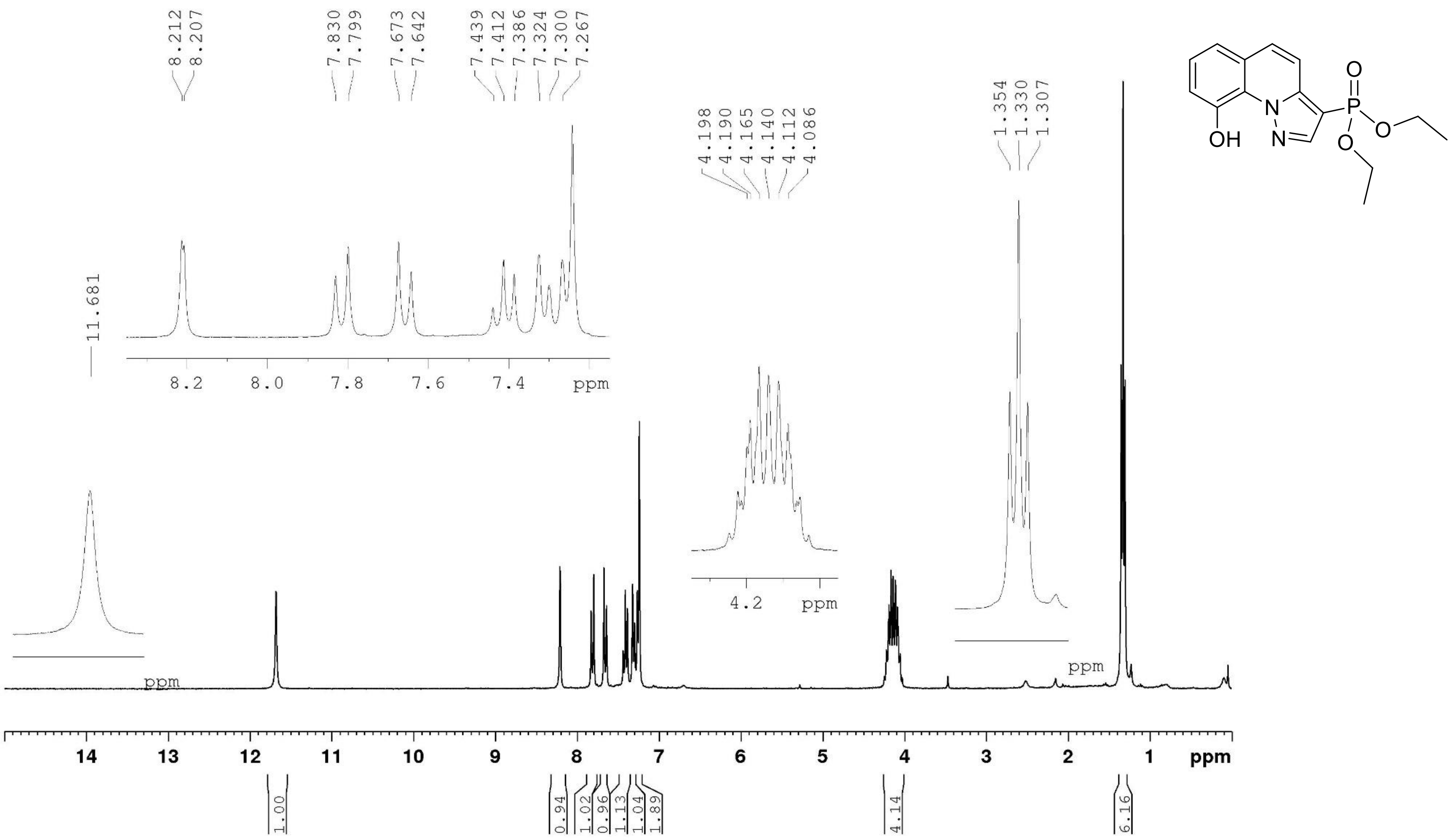

Diethyl (9-hydroxypyrazolo[1,5-a]quinoline-3-yl)phosphonate (5p) ^{13}C NMR.

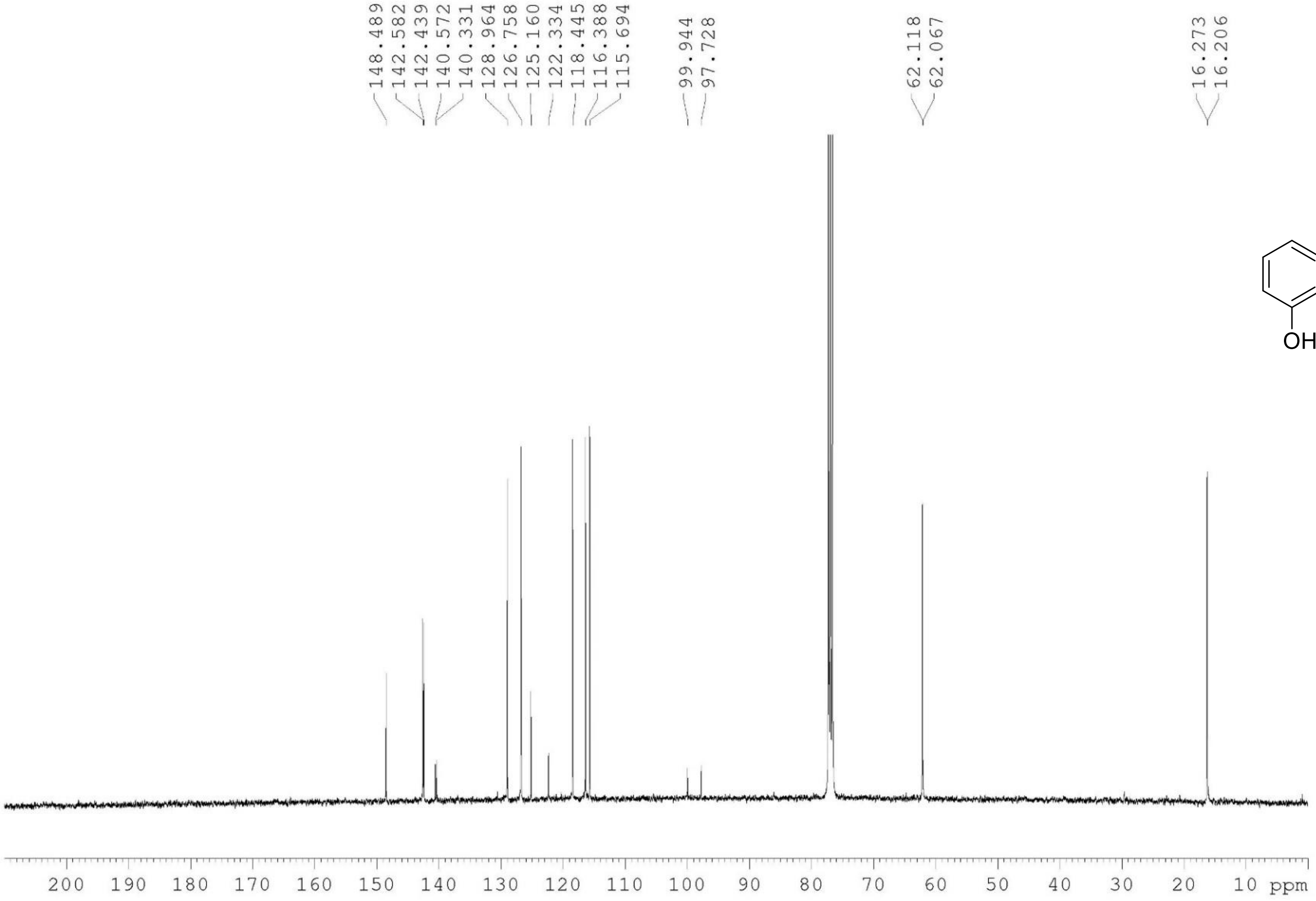

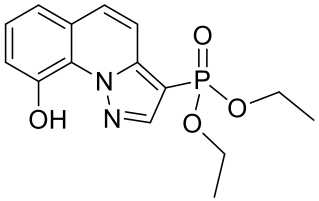

Diethyl (9-hydroxypyrazolo[1,5-a]quinoline-3-yl)phosphonate (5p) ^{31}P NMR.

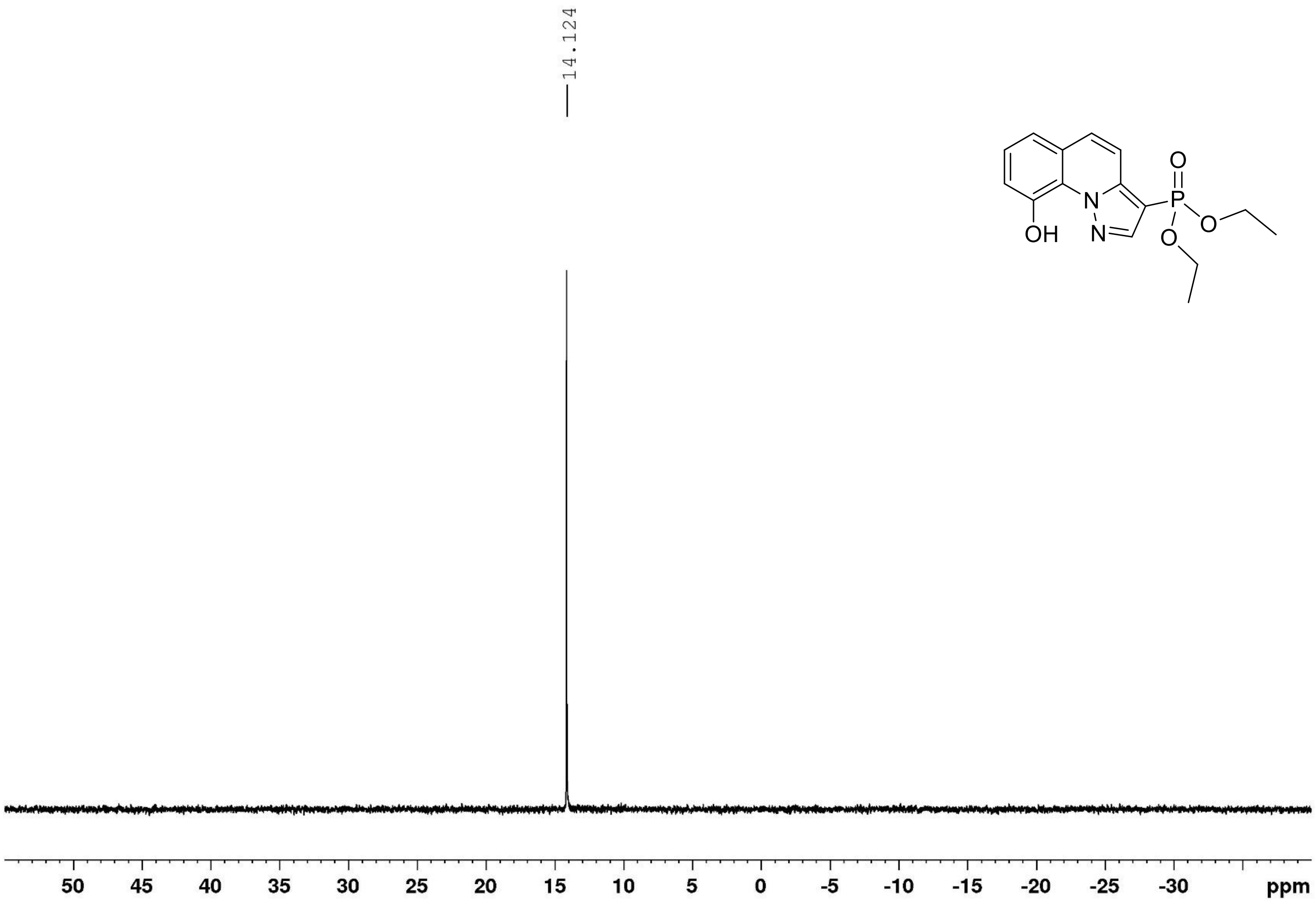

Diethyl (pyrazolo[1,5-a][1,10]phenantroline-3-yl)phosphonate (5q) ^{1}H NMR.

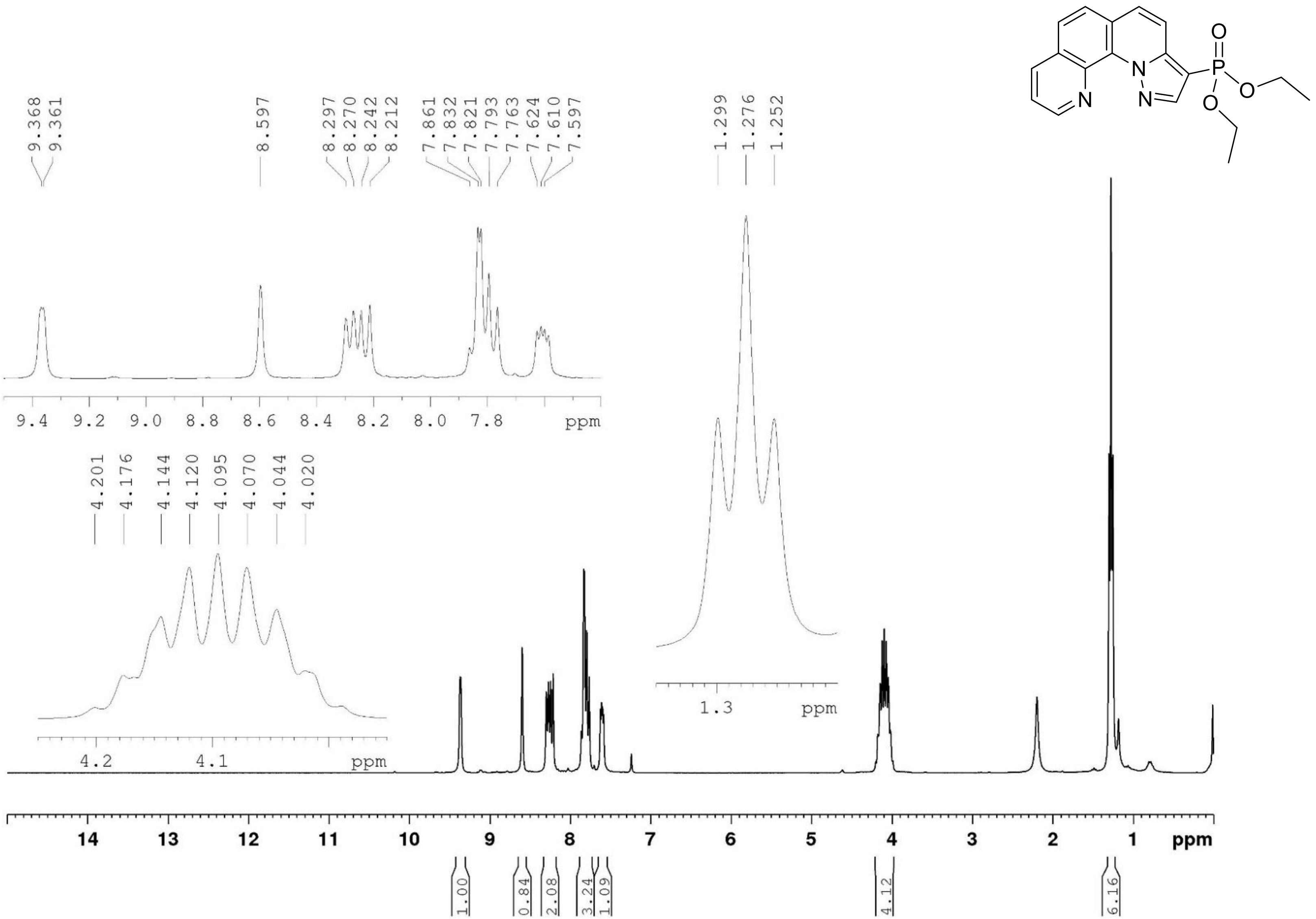

Diethyl (pyrazolo[1,5-a][1,10]phenantroline-3-yl)phosphonate (5q) ^{13}C NMR.

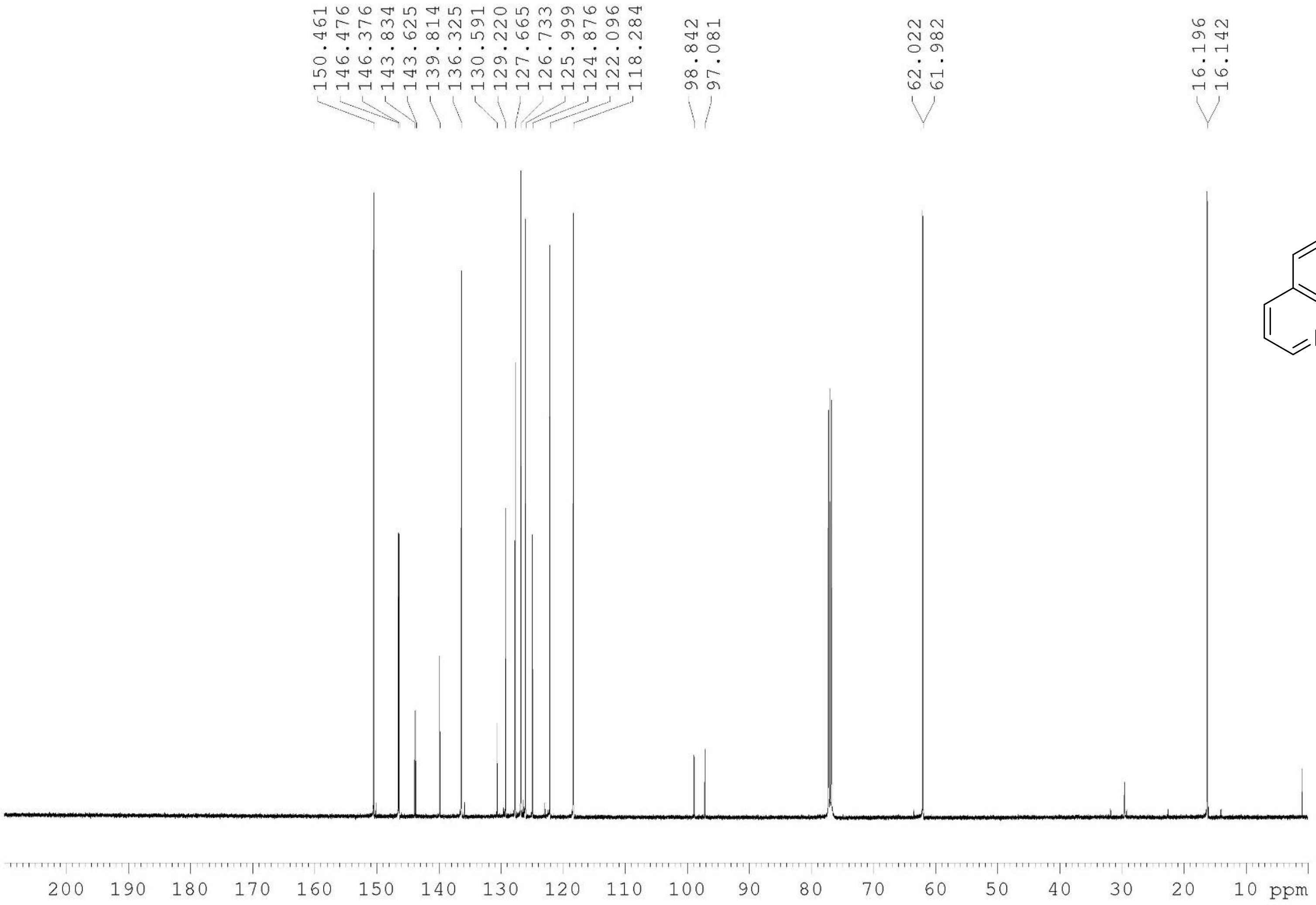

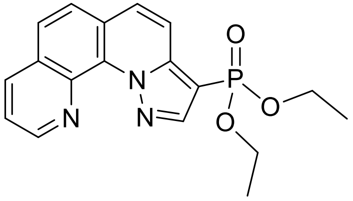

Diethyl (pyrazolo[1,5-a][1,10]phenantroline-3-yl)phosphonate (5q) ^{31}P NMR.

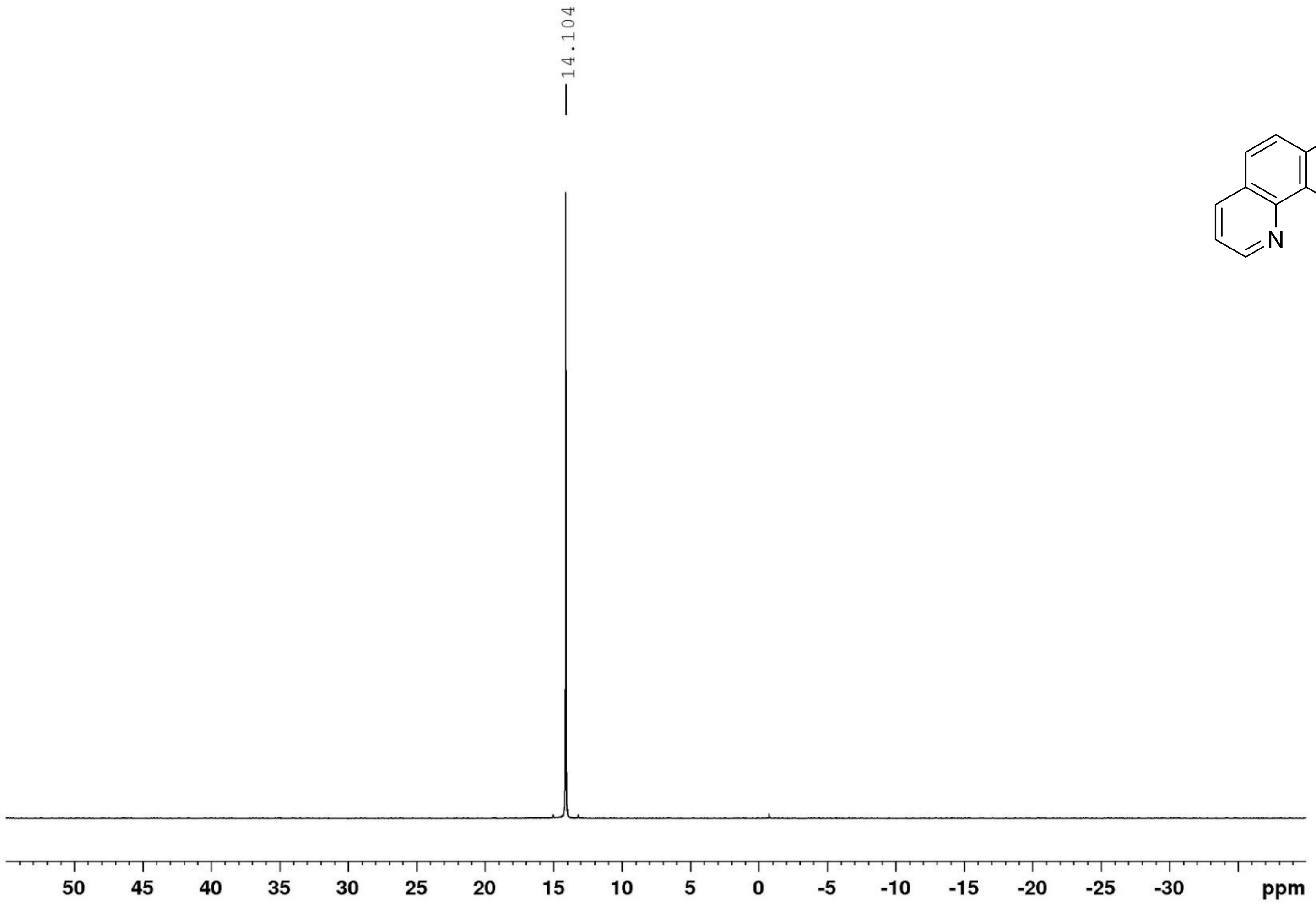

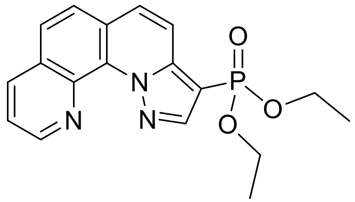

Diethyl (2-chloro[1,5-a]pyridin-3-yl)phosphonate ^{1}H NMR(5r).

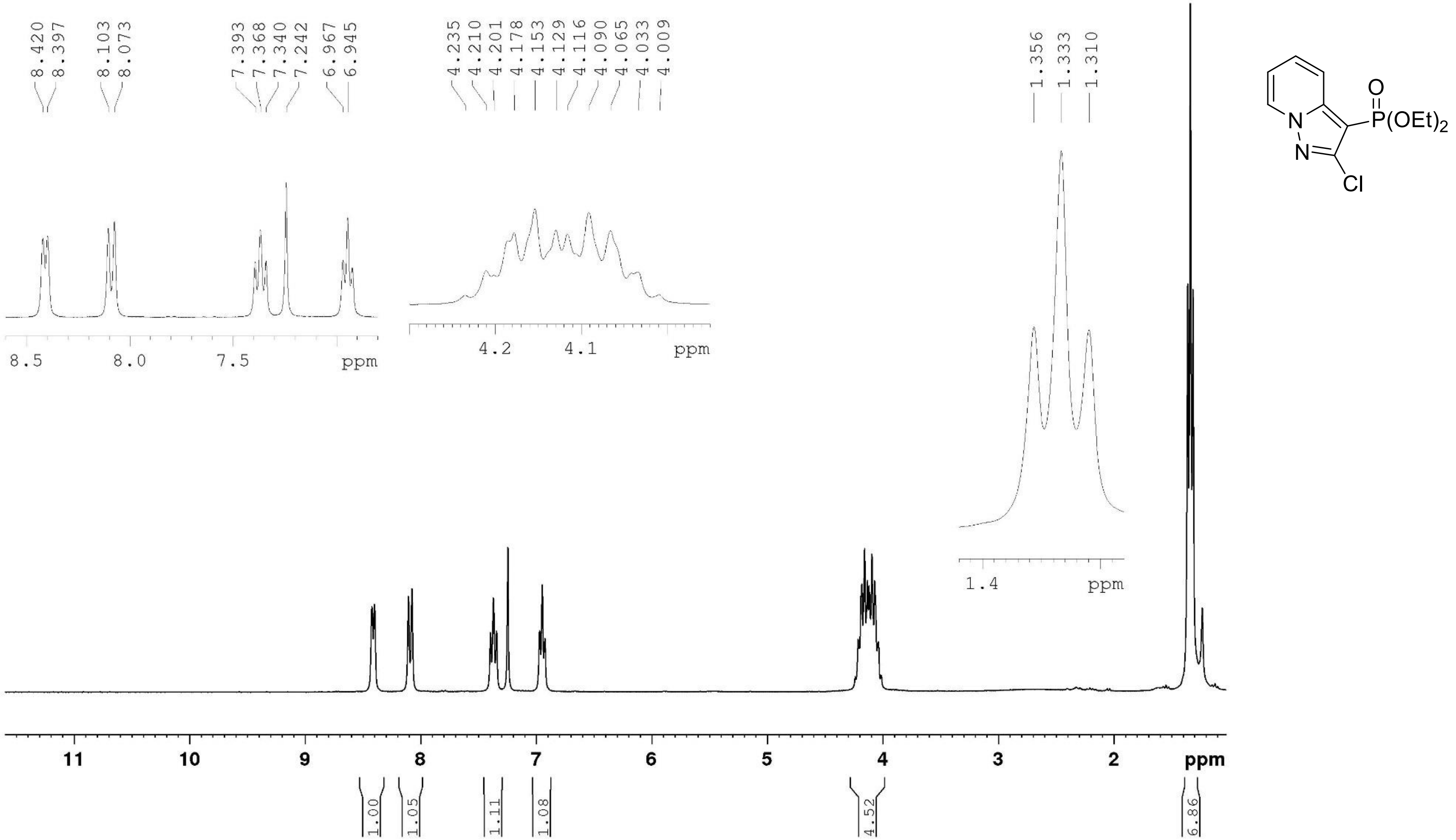

Diethyl (2-chloro[1,5-a]pyridin-3-yl)phosphonate ^{13}C NMR(5r).

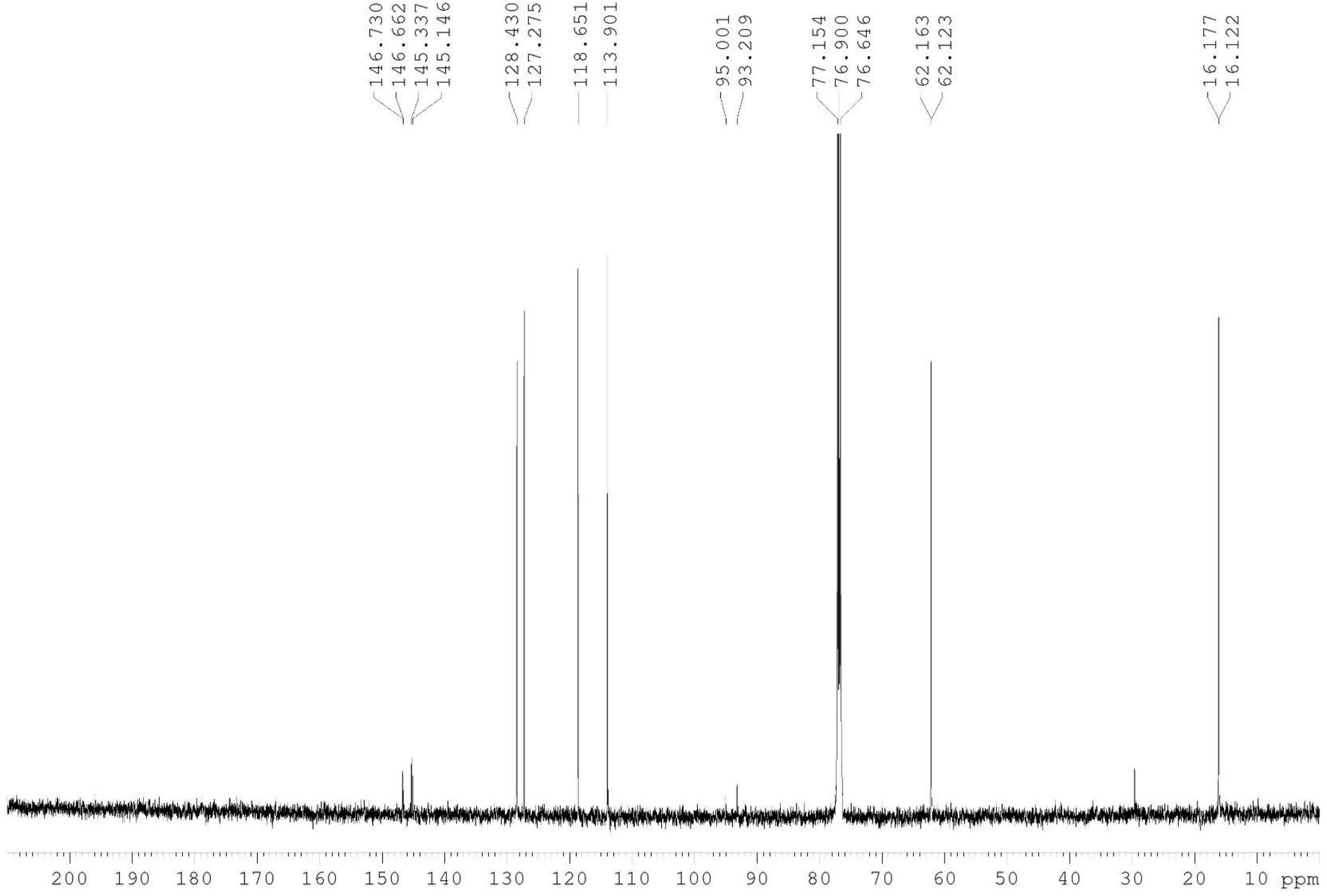
146.730
146.662
145.337
145.146
128.430
127.275
118.651
113.901
95.001
93.209
77.154
76.900
76.646
62.163
62.123
16.177
16.122
200 190 180 170 160 150 140 130 120 110 100 90 80 70 60 50 40 30 20 10 ppm

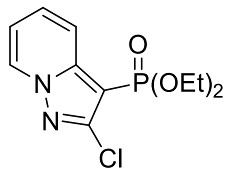
O
P(OEt)2
N
N
Cl

Diethyl (2-chloro[1,5-a]pyridin-3-yl)phosphonate ^{31}P NMR(5r).

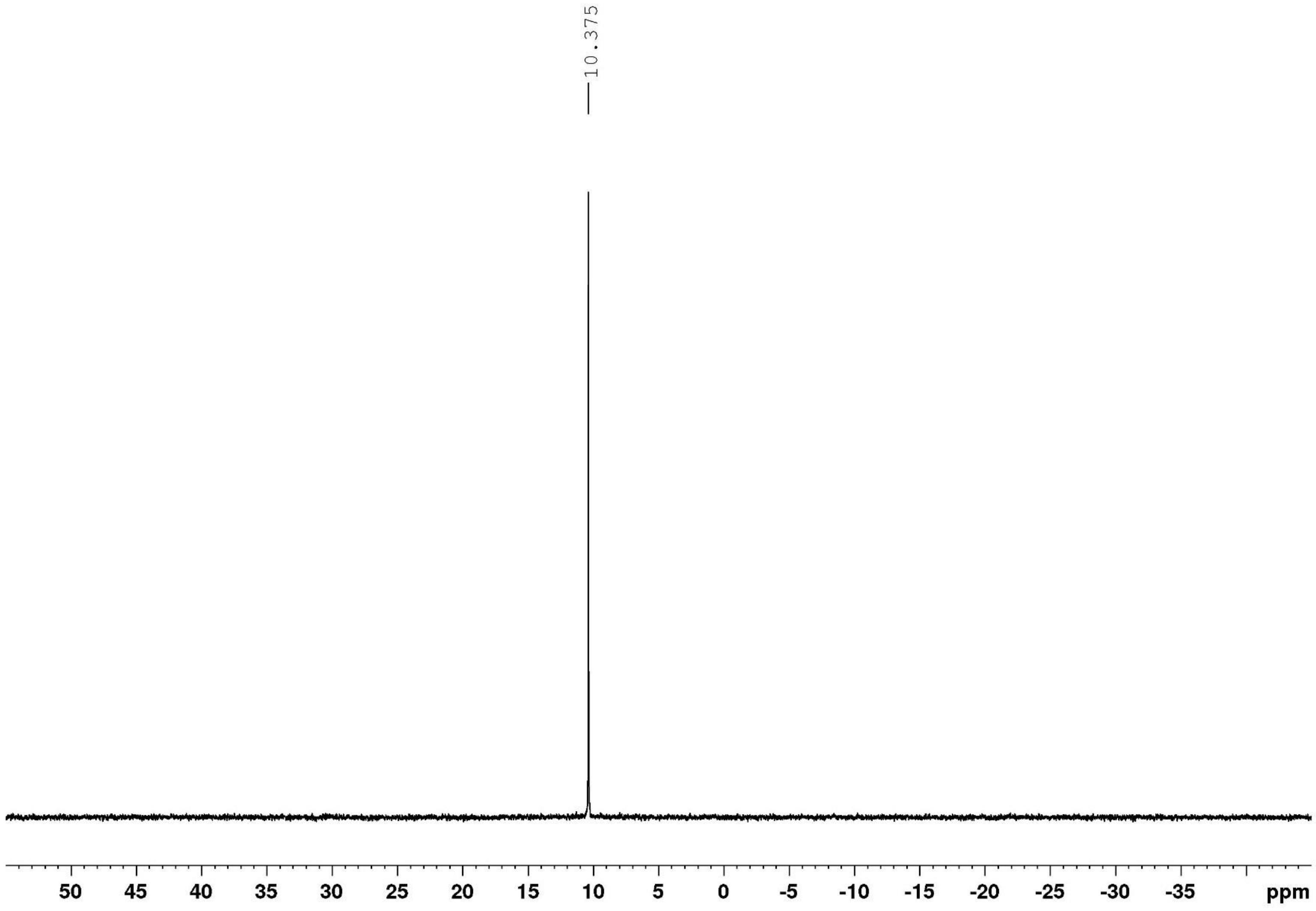

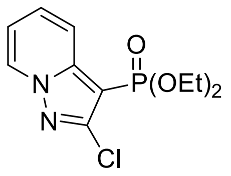

^{31}P NMR spectrum of reaction mixture for diethyl (2-bromo[1,5-a]pyridin-3-yl)phosphonate (5s) preparation.

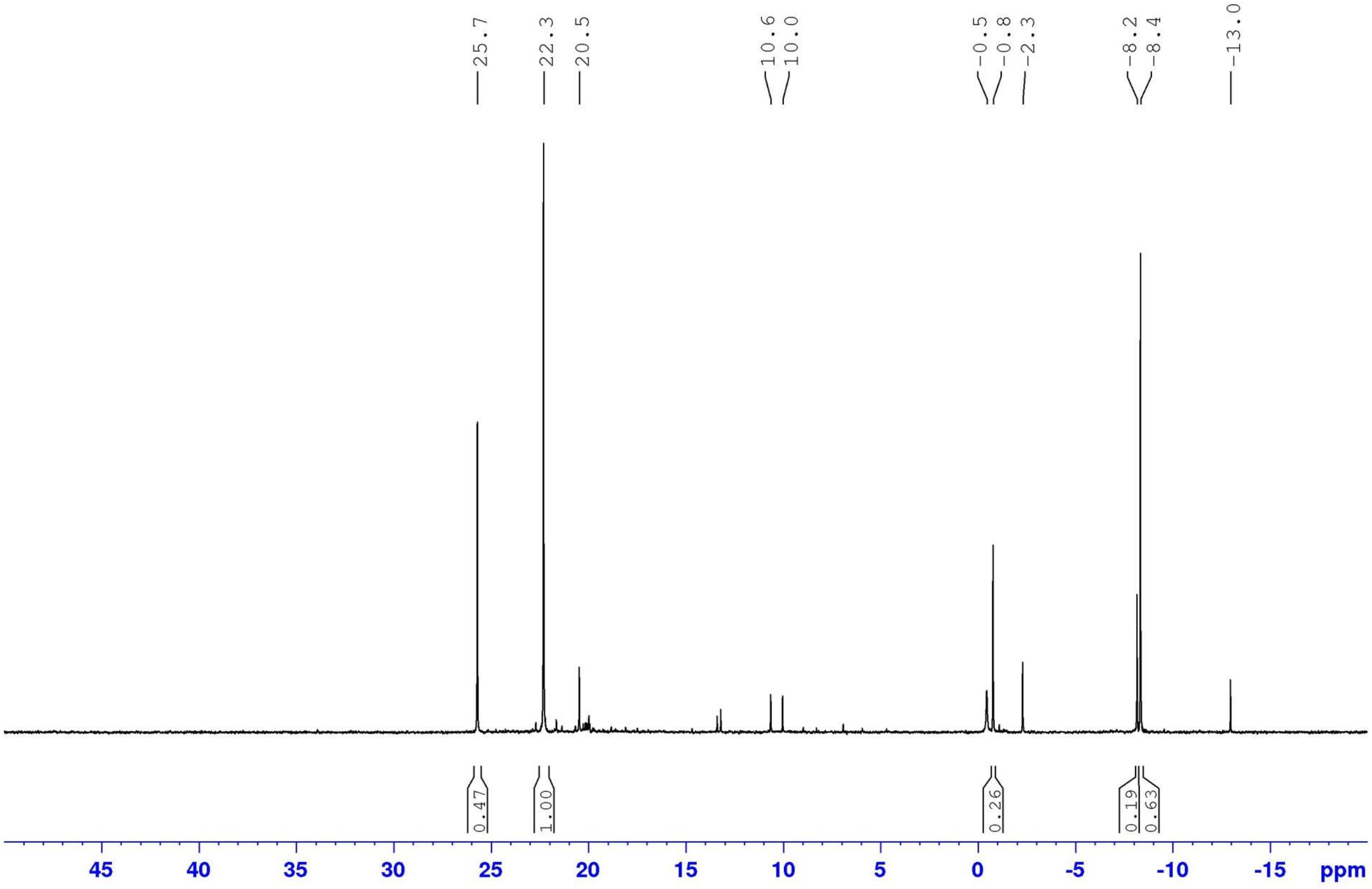

Diethyl (2-iodo[1,5-a]pyridin-3-yl)phosphonate ^{1}H NMR(5t).

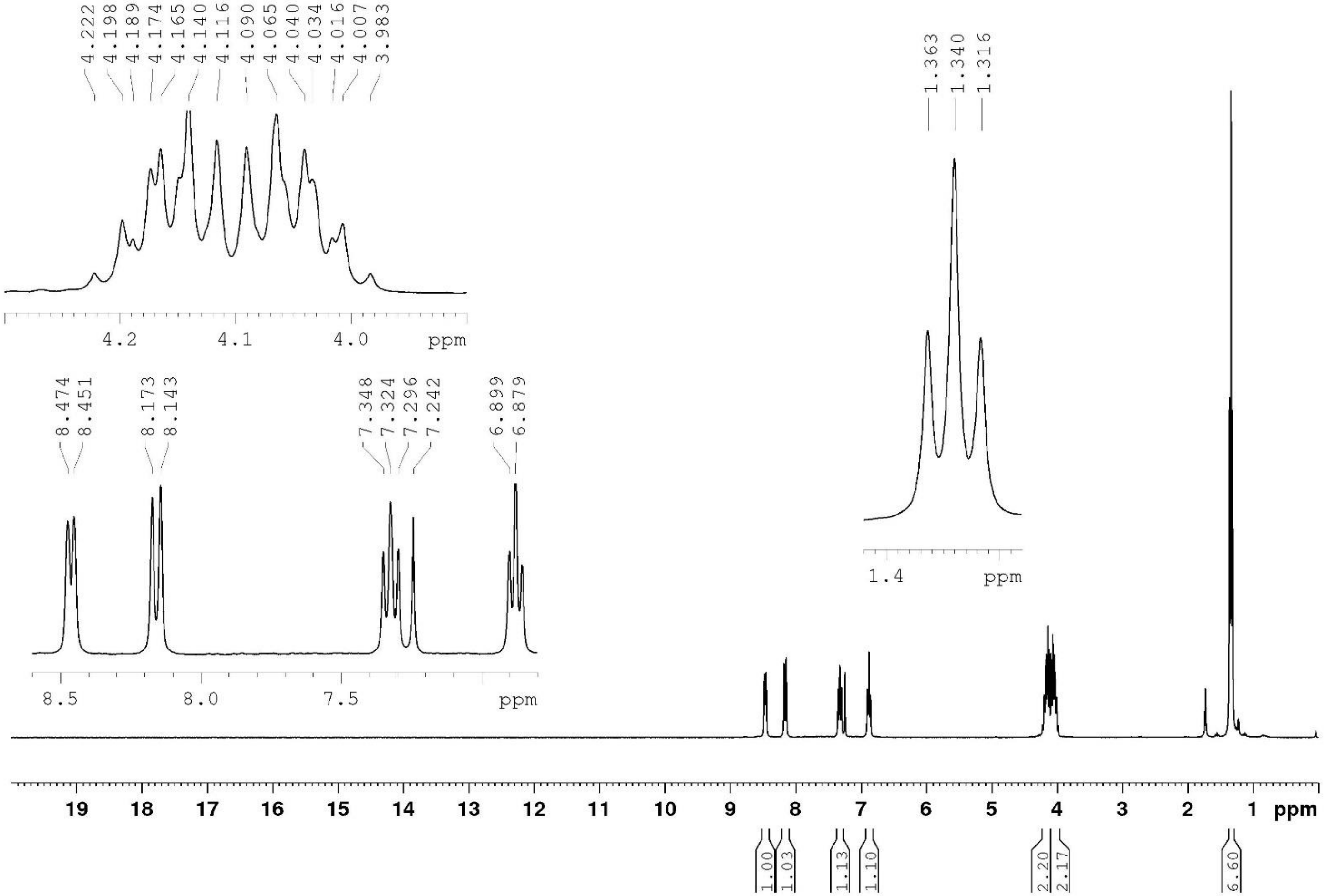

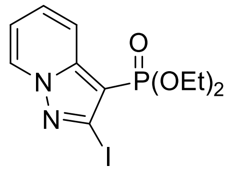

Diethyl (2-iodo[1,5-a]pyridin-3-yl)phosphonate ^{13}C NMR(5t).

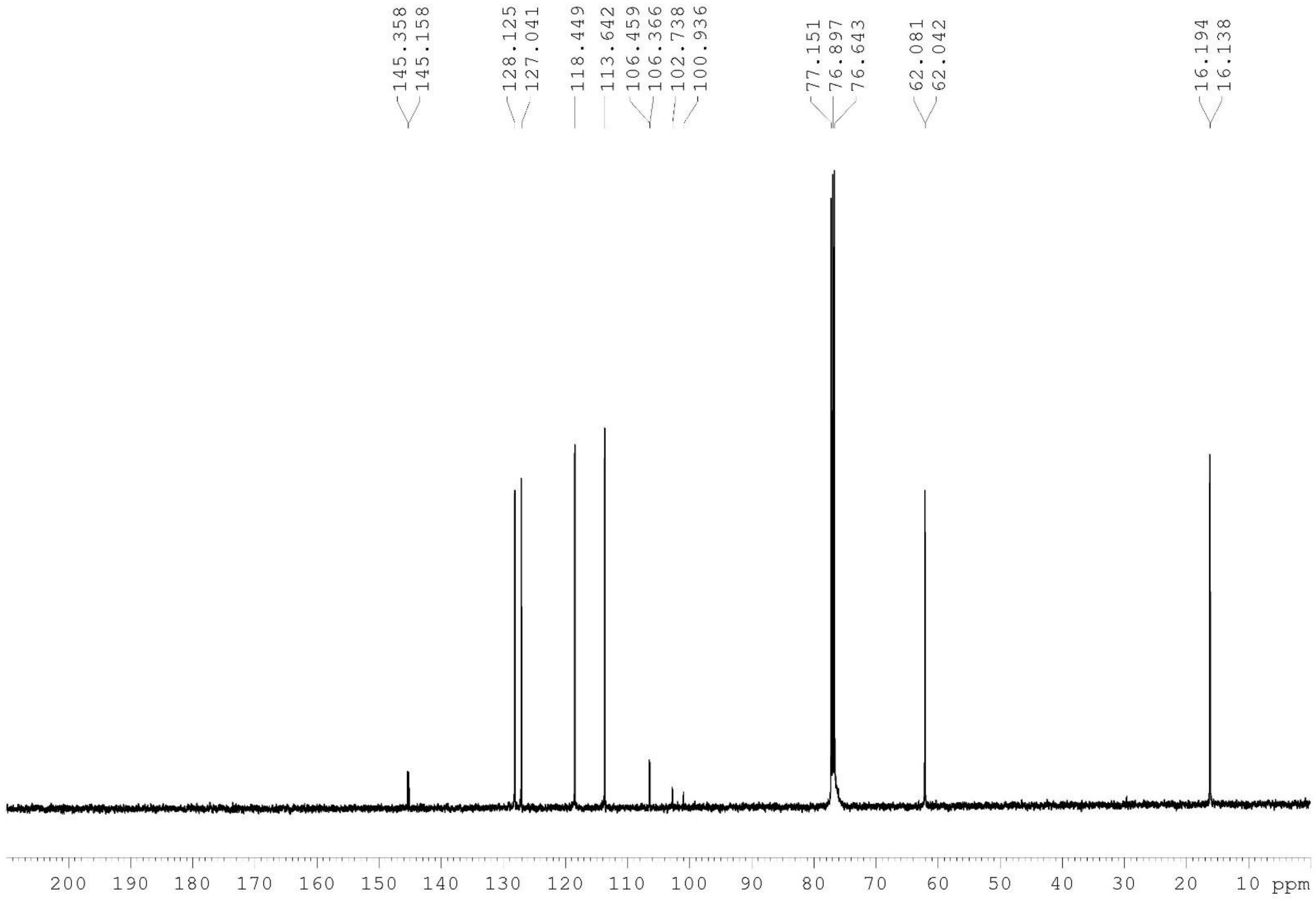

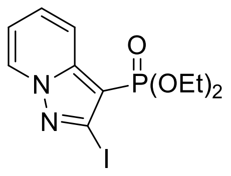

Diethyl (2-iodo[1,5-a]pyridin-3-yl)phosphonate ^{31}P NMR(5t).

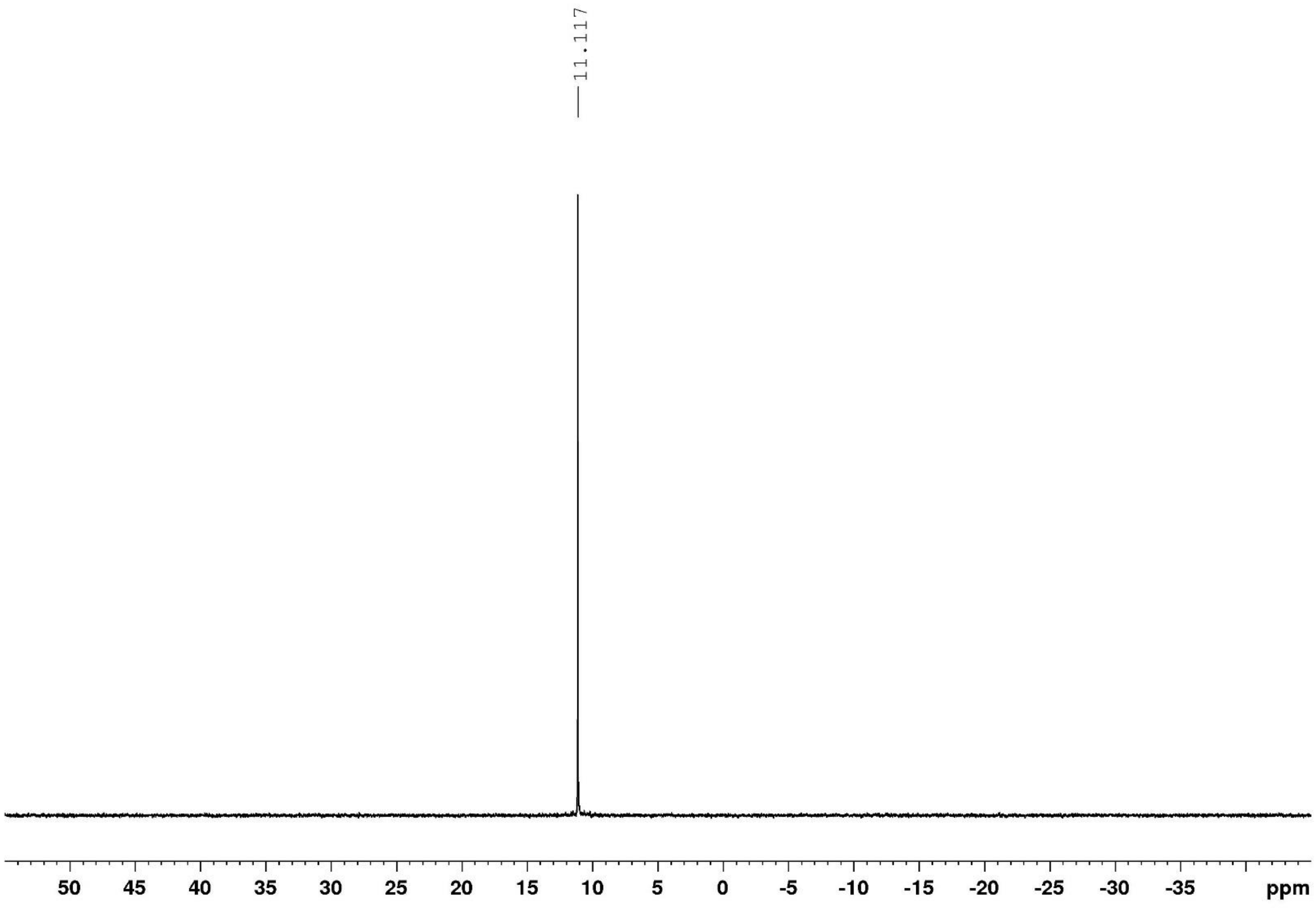

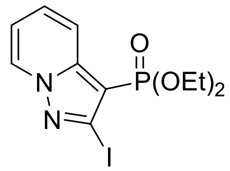

Diethyl (2-phenoxy[1,5-a]pyridin-3-yl)phosphonate ^{1}H NMR(5u).

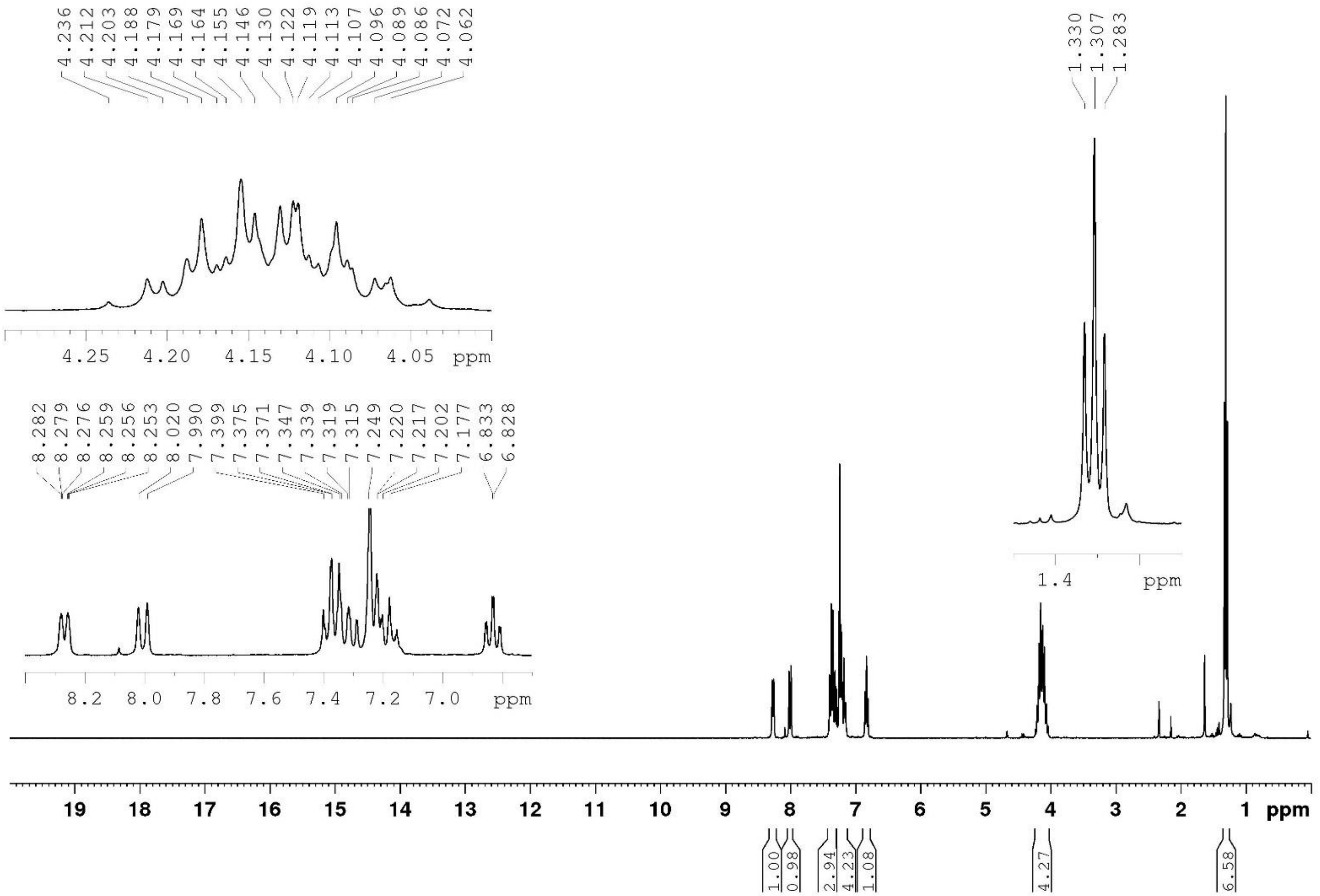

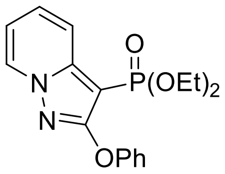

Diethyl (2-phenoxy[1,5-a]pyridin-3-yl)phosphonate ^{13}C NMR(5u).

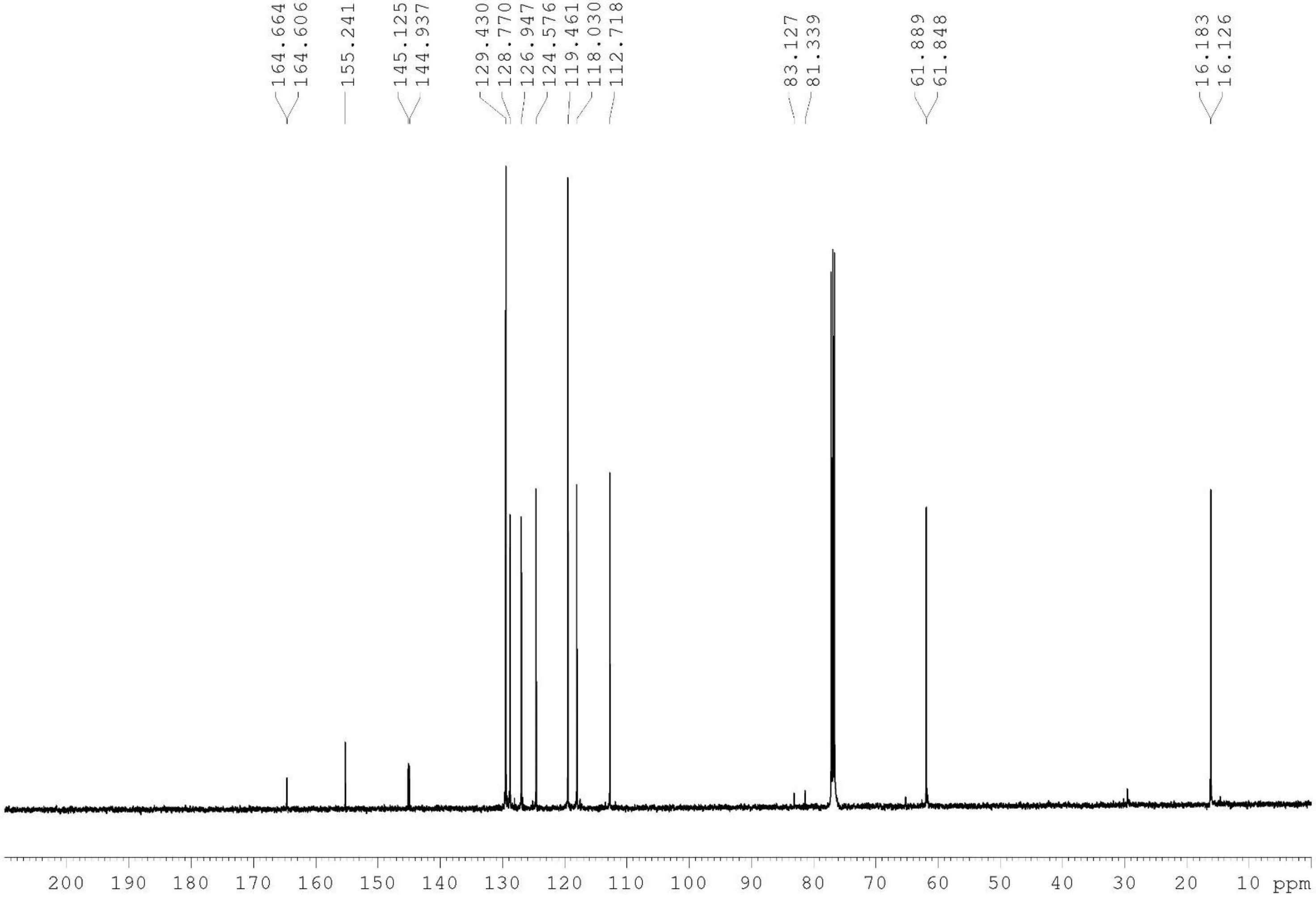

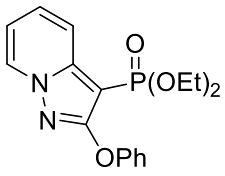

Diethyl (2-phenoxy[1,5-a]pyridin-3-yl)phosphonate ^{31}P NMR(5u).

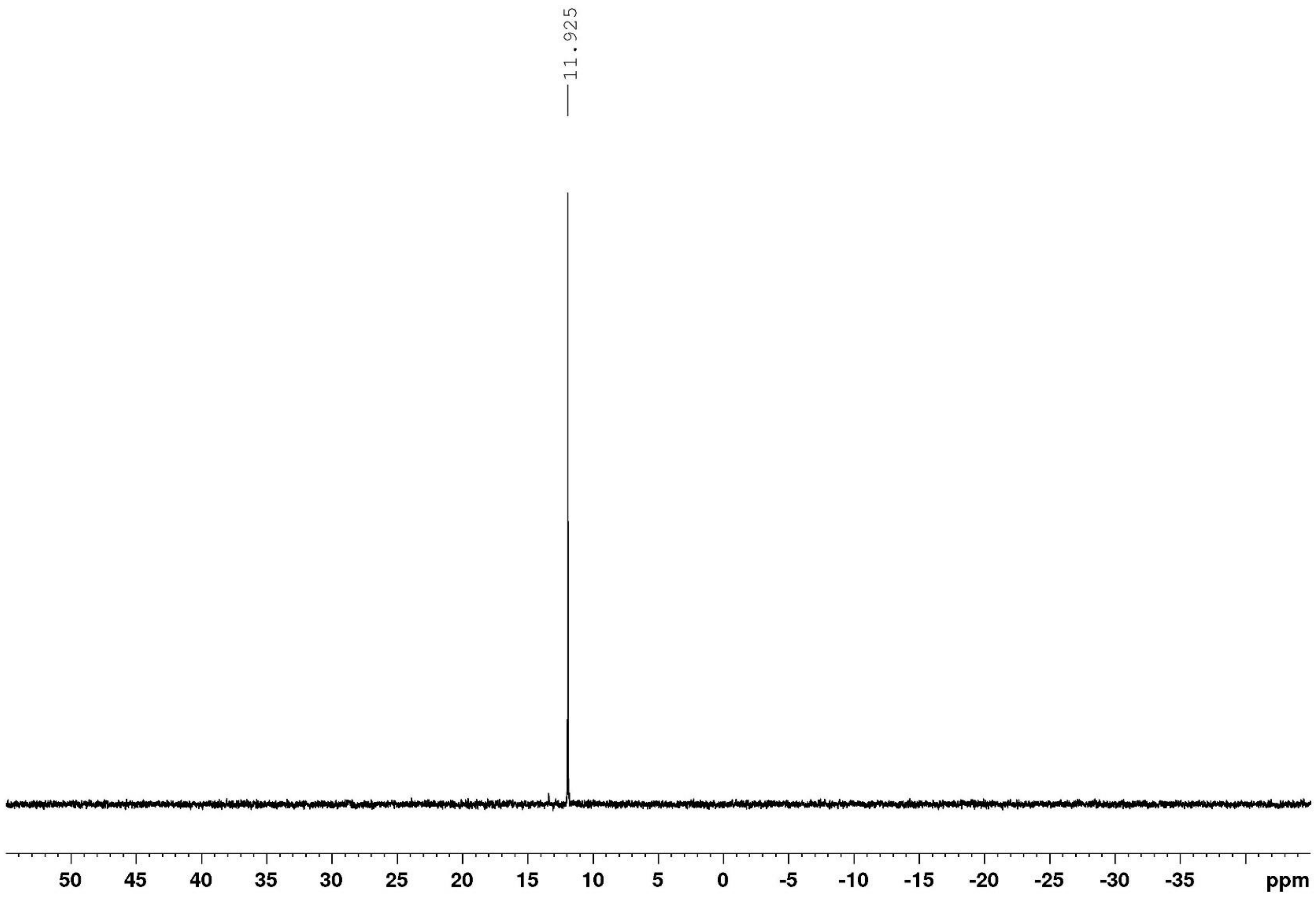

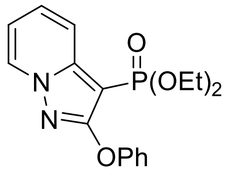

Supplement: Supplementary file 1 [file molecules-27-07913-s001.zip › molecules-2012525-supplementary.pdf]
